# Universality of Hair as a Nucleant: Exploring the Effects of Surface Chemistry and Topography

**DOI:** 10.1021/acs.cgd.3c01035

**Published:** 2023-11-11

**Authors:** Thomas H. Dunn, Sebastian. A. Skaanvik, Ian J. McPherson, Cedrick O’Shaughnessy, Xuefeng He, Alexander N. Kulak, Stuart Micklethwaite, Adriana Matamoros-Veloza, Ilaria Sandei, Liam Hunter, Thomas D. Turner, Johanna M. Galloway, Martin Rosenthal, Andrew J. Britton, Marc Walker, Mingdong Dong, Patrick R. Unwin, Fiona C. Meldrum

**Affiliations:** † School of Chemistry, University of Leeds, Woodhouse Lane, Leeds LS2 9JT, U.K.; ‡ Interdisciplinary Nanoscience Center (iNANO), Aarhus University, 8000 Aarhus C, Denmark; § Department of Chemistry, University of Warwick, Coventry CV4 7AL, U.K.; ∥ Department of Chemistry, Loughborough University, Loughborough LE11 3TU, U.K.; ⊥ Department of Chemistry, KU Leuven, Celestijnenlaan 200F, Box 2404, B-3001 Leuven, Belgium; # Dual-Belgian-Beamline (DUBBLE), European Synchrotron Radiation Facility (ESRF), 71 Avenue des Martyrs, CS40220, 38043 Grenoble Cedex 9, France; ∇ Bragg Centre for Materials Research, University of Leeds, Woodhouse Lane, Leeds LS2 9JT, U.K.; ○ Department of Physics, University of Warwick, Coventry CV4 7AL, U.K.

## Abstract

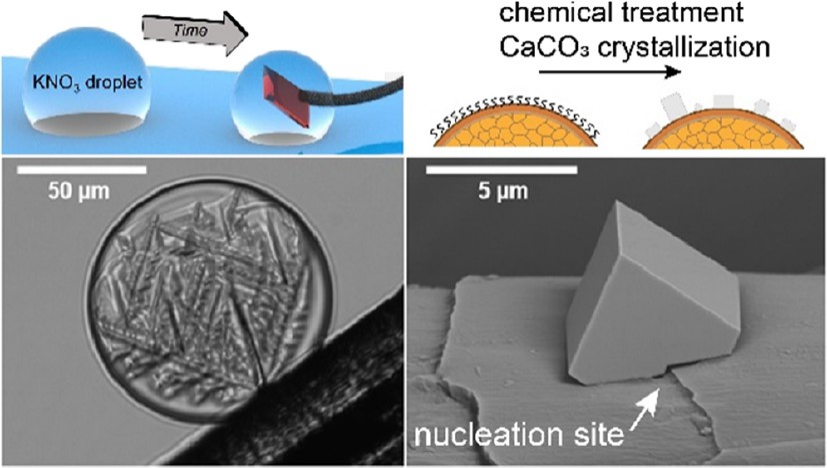

The ability to control crystal nucleation through the simple addition of a nucleating agent (nucleant) is desirable for a huge range of applications. However, effective nucleating agents are known for only a small number of systems, and many questions remain about the mechanisms by which they operate. Here, we explore the features that make an effective nucleant and demonstrate that the biological material hair—which naturally possesses a chemically and topographically complex surface structure—has excellent potential as an effective nucleating agent. Crystallization of poorly soluble compounds in the presence of hairs from a range of mammals shows that nucleation preferentially occurs at the cuticle step edges, while a novel microdroplet-based methodology was used to quantify the nucleating activities of different hairs. This showed that the activities of the hairs can be tuned over a wide range using chemical treatments. Analysis of the hair structure and composition using atomic force microscopy, scanning ion conductance microscopy, and X-ray photoelectron spectroscopy demonstrates that surface chemistry, surface topography, and surface charge all act in combination to create effective nucleation sites. This work therefore contributes to our understanding of heterogeneous nucleating agents and shows that surface topography as well as surface chemistry can be used in the design or selection of universal nucleating agents.

## Abstract 2

Identification of preferred nucleation sites and measurement of nucleation kinetics show that surface topography and surface chemistry combine to make hair an effective nucleant. This biological material is highly effective for both sparingly soluble and highly soluble inorganic compounds and its activity can be further tuned by chemical treatment.

## 1 Introduction

The ability to control crystal nucleation is key to a vast range of processes as diverse as the production of high-quality protein crystals, cloud seeding, the prevention of kidney stones and scale build-up in heating systems, the generation of pharmaceuticals with specific polymorphs, and control of gas hydrate formation.^1−4^ However, in contrast to the many strategies that exist for controlling crystal growth, control over nucleation remains highly challenging. One of the most attractive potential strategies is the addition of foreign nucleating agents (nucleants), which can determine nucleation rates, nucleation location, and properties of the crystalline product such as polymorph. However, practical experience shows that it can be highly challenging to identify effective nucleants—with the exception of seed crystals or compounds that possess an epitaxial match with the nucleating compound^5^—and many questions remain concerning the mechanisms by which they operate.

Studies to find effective nucleants have principally focused on ice and proteins,^6−9^ and relatively few have been identified even for widely used inorganic compounds such as calcium carbonate.^10^ An attractive strategy for increasing the efficiency of this search process is the discovery of “universal nucleants” that can direct the nucleation of multiple compounds.^11^ As potential candidates, materials of biological origin are of particular interest. Structures from a diverse range of organisms, including lichens, pollens, and fungi have been identified as excellent ice nucleants,^6^ and the ability of some bacteria to generate ice nucleating proteins has even been exploited in the production of commercial nucleants.^12−14^ Indeed, rabbit hair was used to generate the first artificial snowflakes ever made.^15^ Animal hair is also often used as a nucleant for protein crystals,^16−18^ and animal hair fragments have recently been incorporated in high-throughput crystallization assays to produce nuclei at lower supersaturations,^19,20^ leading to more controlled growth and higher quality crystals.

Here, we investigate the origins of the activity of hair as a nucleant and explore its potential to nucleate inorganic crystals, a class of compounds not considered previously. Hair also exhibits a complex surface topography that varies according to species and thus provides a unique opportunity to explore the combined roles of surface chemistry and topography on nucleation. While surface chemistry is invariably the primary factor considered when rationalizing the capacity of a surface to promote nucleation,^21−23^ there is growing evidence that surface topographical features including cracks and pits can also contribute significantly to its activity.^24−27^ Two contrasting systems are examined—CaCO_3_ and KNO_3_—where these exhibit common polymorphs^28,29^ but differ greatly in their solubilities and nucleation kinetics.^30,31^ Sparingly soluble CaCO_3_ is used to identify the nucleation sites on the hair, while a novel microdroplet methodology was developed to assess the nucleation kinetics of KNO_3_ in the presence of hair. Investigation of the effects of various chemical treatments then allowed us to correlate nucleating properties to surface chemistry, charge, and topography using X-ray photoelectron spectroscopy (XPS), atomic force microscopy (AFM), and scanning ion conductance microscopy (SICM). Our study demonstrates that hair is an effective nucleant for multiple compounds and increases our mechanistic understanding of its behavior and, in particular, the role of surface topography, which should ultimately enable us to identify and even design new nucleants.

## 2 Methods

### 2.1 Hair Source and Modifications

Dog hair was collected from a miniature Irish doodle, and human (head) hair was generously supplied by an individual with thick, straight, black hair. Mole, mink, squirrel, camel, and elk hairs were purchased in a selection box of fly fishing materials (Sportfish, Farlows Ltd.). Bat hairs were kindly supplied by the South Yorkshire Bat Group and the Northumberland Bat Group. The contribution of surface chemistry to the behavior of the hairs was investigated by employing three surface treatment strategies by immersion in: (i) ethanol, (ii) petroleum ether, and (iii) hydrogen peroxide before water rinsing (Section S1.1). Petroleum ether removes lipids from the surface, ethanol removes lipids and denatures proteins, and hydrogen peroxide oxidizes the proteins on the hair surface, altering its chemistry and surface charge.^32−35^

### 2.2 Calcium Carbonate Crystallization

The performance, distribution, and nature of nucleation sites present on a range of different hair types were determined by precipitating CaCO_3_ on their surfaces by immersion in a 2.5 mM equimolar solution of CaCl_2_ and Na_2_CO_3_ for 20 min, corresponding to a *C*/*C*_sat_ of 2.90. As a sparingly soluble compound, CaCO_3_ forms as small crystals, and their number density and locations with respect to the topography of the hair surface could be readily determined by scanning electron microscopy (SEM), and their polymorphs by Raman spectroscopy (Sections S1.1–1.3). Nucleation sites were investigated by placing crystal-coated hairs on the surface of an uncured resin and allowing it to cure before the hair was gently peeled from the resin surface, leaving an imprint of the hair with the undersides of the crystals exposed (Section S1.1). This enabled the relationship between the topographical features and the center of the crystals to be identified.

### 2.3 Crystallization of Potassium Nitrate in Microdroplets

A second method was employed to quantify the activities of the different hairs, where individual hairs were used to determine the concentration range in which nucleation was triggered within KNO_3_ microdroplets. Detailed information is found in Section S2.1. Briefly, the microdroplets of aqueous KNO_3_ (≈1 nL, 2.5 M) were deposited on hydrophobic glass slides under a layer of silicone oil using a nanopipette and Eppendorf InjectMan system, while the temperature was continuously recorded. Diffusion of water from the microdroplets to the silicone oil led to a gradual decrease in volume and an increase in the concentration of KNO_3_ within the microdroplets. The concentrations of the droplets could then be calculated from the droplet volumes at any point in time, which were determined using optical microscopy with automated image analysis (Sections S2.2 and S2.3). The behavior of hair as a nucleant was investigated by touching supersaturated droplets of different concentrations with the side of a hair (Movie S2) and recording whether a crystal was produced. Care was taken to ensure that only the region under investigation—the side of the hair—contacted each droplet.

### 2.4 Levitated Droplet Synchrotron Wide-Angle X-ray Scattering Measurements of KNO_3_ Crystallization

In situ wide-angle X-ray scattering (WAXS) measurements were collected at the European Synchrotron Radiation Facility (ESRF) to investigate phase transformations occurring within the microdroplets (Section S3). A 2 μL KNO_3_ droplet (0.5 M) was deposited into an acoustic levitator using a hydrophobic needle. A dog hair was grafted to Poly(tetrafluoroethylene) (PTFE) capillary tubing and placed in contact with a levitated droplet. The droplet evaporated under ambient conditions while time-resolved WAXS was simultaneously collected. The 2D WAXS patterns were background-corrected by using the first frame at the beamline using PyFAI software and averaged every 50 frames after preliminary analysis.

### 2.5 Characterization of the Surface Chemistry and Topography of Hair Samples

The topography, surface chemistry, and surface charge of the hair samples were carefully characterized by different techniques (Section S4). The topography of the hair samples could readily be determined with high resolution by AFM. The surface chemistry was determined by XPS, where survey scans give the elemental composition, providing information about the amount of lipids and proteins in the surface layer (5–10 nm). High-resolution sulfur 2p scans give chemical information about the protein oxidation state. The heterogeneity of the surface charge of the hairs immersed in aqueous electrolytes was determined by SICM. In such experiments, an ionic current is passed through a glass nanopipet that is used as a probe to scan the hair surface. The measured ionic current is proportional to the ionic conductance of the system, which is related to the surface charge through developed simulations. More information about the simulations is found in the Supporting Information (Sections S5 and S6).

## 3 Results

### 3.1 Calcium Carbonate Crystallization on Human and Dog Hair

**Figure 1:**
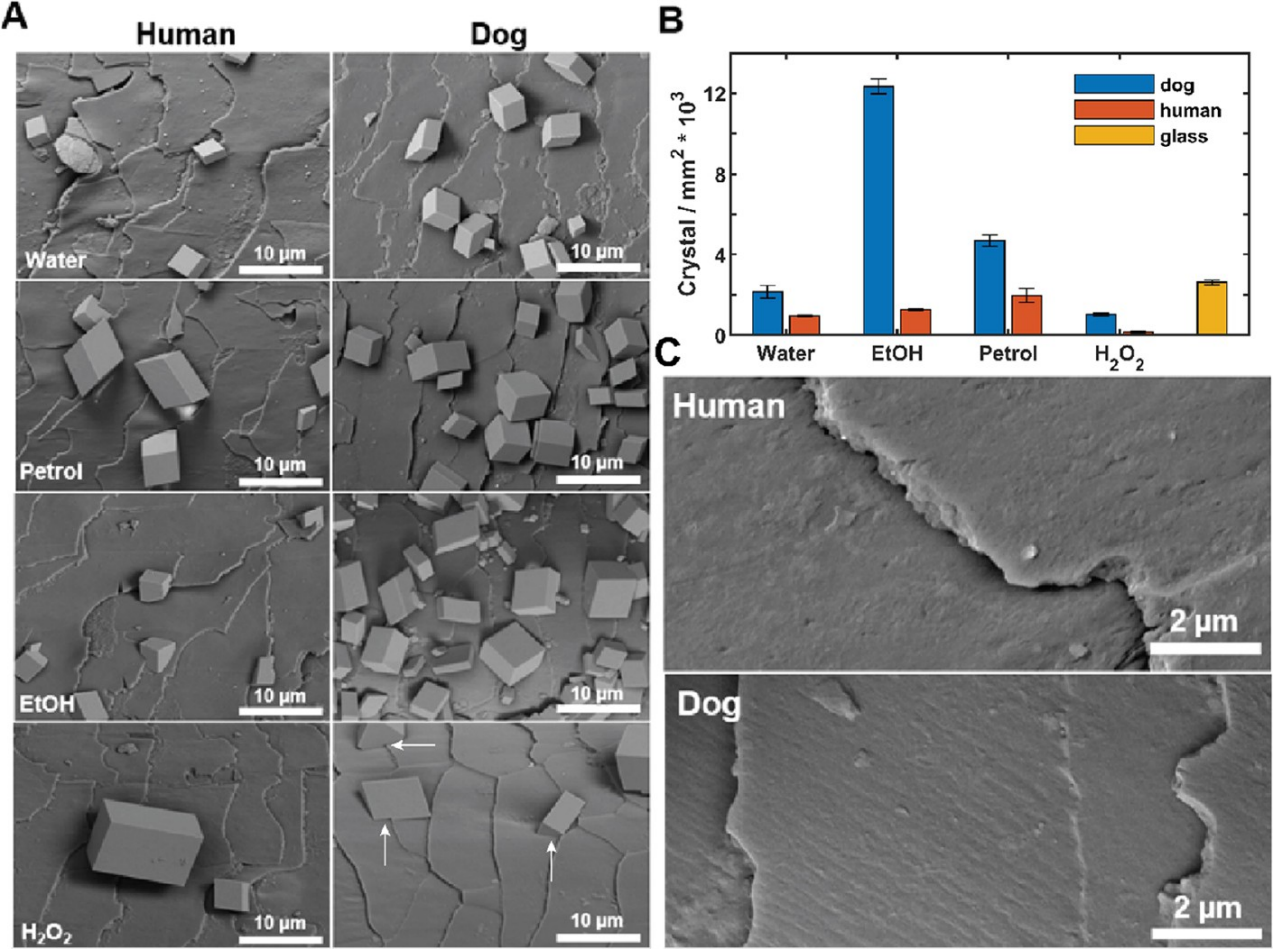
(A) SEM images of CaCO_3_ crystals grown on human and dog hair after chemical treatments. (B) Crystal density for dog and human hair under various chemical treatments to modify the surface structure. The error bars represent the standard deviation (*n* = 3). (C) High-magnification SEM images showing the cuticle morphologies of human and dog hair.

Initial studies focused on characterizing and using human and dog hair that had been thoroughly rinsed in water to nucleate CaCO_3_ crystals (Section S1.1). SEM showed that both of these hair types exhibited steplike cuticle edges (Figure 1a,c). The crystals were ≈5 μm in size in both cases and were rhombohedral in morphology (Figure 1a), and the polymorph was shown to be calcite (Section S1.2). Comparable crystals formed in control experiments in which a glass slide was used as a crystallization substrate. Evaluation of the number density of crystals showed that human hair (949 ± 81 crystals/mm^2^) performed worse than the pristine glass surface (2612 ± 268 crystals/mm^2^), and that dog hair (2146 ± 628 crystals/mm^2^) performed similarly as a nucleant to the glass. (Figure 1b and Section S1.3).

Surface treatment of the hairs had a dramatic effect on the number density of the crystals formed (Figure 1b). Ethanol washing increased the activity of dog hair (12,338 ± 740 crystals/mm^2^) by over five times but had little effect on human hair (1259 ± 91 crystals/mm^2^). Petroleum ether caused a modest increase in the number density (4683 ± 593 and 1959 ± 690 crystals/mm^2^ for dog and human hairs, respectively), while hydrogen peroxide significantly reduced the activity of both hair types (1012 ± 134 and 156 ± 78 crystals/mm^2^ for dog and human hairs, respectively). These results show that exposing the underlying protein-rich surface under nonoxidative conditions improves the nucleation density of the active dog hair. Moreover, a synergistic effect for ethanol treatment is seen where the removal of lipids and protein denaturing increases the availability/efficiency of the surface proteins for the nucleation of calcite. The images of the encrusted hairs suggest that the calcite crystals tended to form preferentially adjacent to the cuticle edges. This is most clear for the hydrogen peroxide-treated dog hair shown in Figure 1a (bottom left panel), where the small number of crystals present enables their locations to be identified.

**Figure 2:**
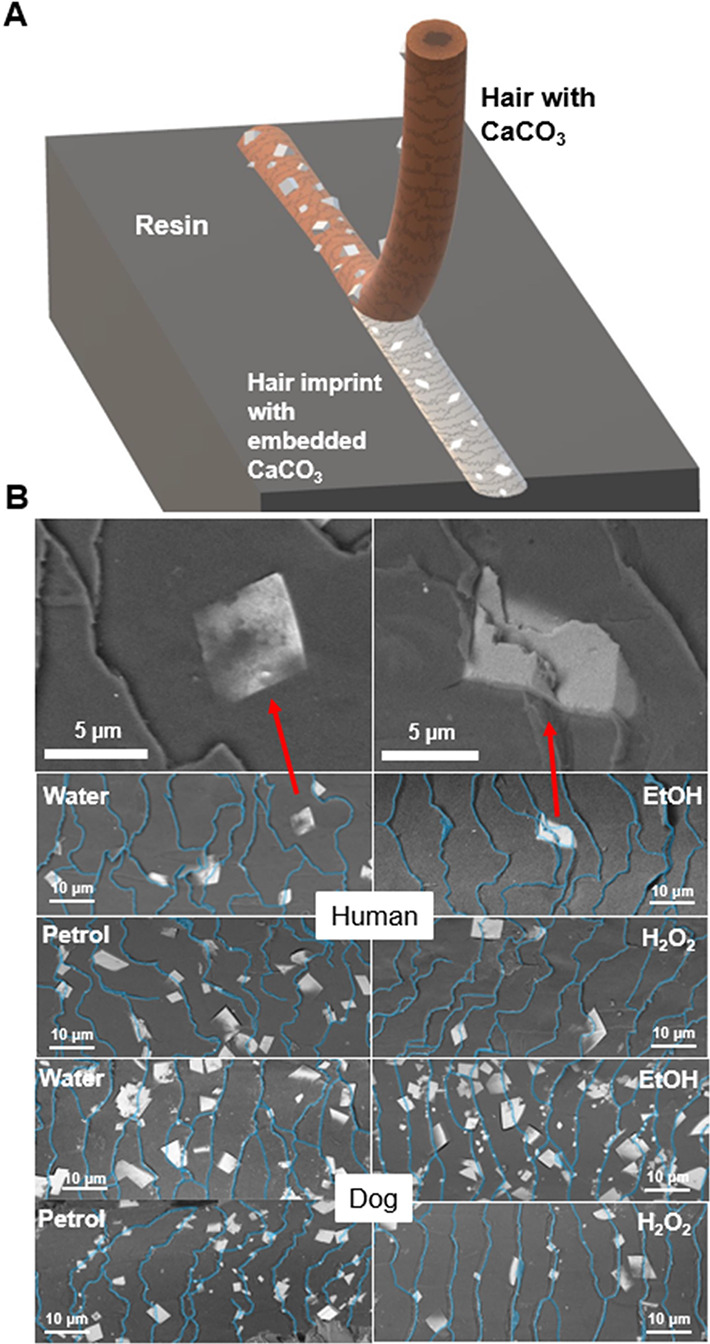
(A) Schematic demonstrating how a hair sample encrusted with CaCO_3_ crystals is placed on a wet resin and then cured. The hair is then gently peeled from the cured resin, leaving an imprint of the hair with the undersides of the crystals exposed. (B) SEM images showing these imprints for each hair treatment procedure. The cuticle edges have been marked in blue.

The nucleation sites were then further investigated by placing crystal-coated hairs on the surface of an uncured resin and allowing it to cure (Figure 2). The water and petroleum ether-treated hairs showed a slight enhancement of nucleation at the cuticle edges, while ethanol and peroxide treatments led to a significant increase in the proportion of crystals located at these sites. The cuticle edges give rise to a preferred environment for nucleation and can act as a nucleation site even when the rest of the cuticle scales are deactivated, resulting from the altered chemistry and topography at the cuticle edge.

### 3.2 Crystallization of Potassium Nitrate in Microdroplets

Precipitation of CaCO_3_ on the surfaces of hair offers a straightforward means of assessing their performances as nucleating agents and identifying preferred nucleation sites. Quantification of the activities of the different hair samples within a range of solution concentrations is more challenging, where previously reported methods have required the hair to be ground into fragments.^20^ The exposed hair surface resulting from grinding comprises a highly variable mixture of inner and outer regions, which is not representative of the native surface.^17,20^ We therefore developed a novel strategy in which individual intact hairs were used to trigger the nucleation of KNO_3_ crystals within supersaturated microdroplets. Arrays of microdroplets are ideally suited to nucleation studies since multiple experiments can be rapidly conducted while excluding the majority of heterogeneous contaminants.^36,37^ High supersaturations are achievable in contaminant-free microdroplets, enabling nucleation to be triggered at will by introducing a nucleant.^38,39^ We exploit this to compare the nucleant behavior over a wide concentration range.

#### 3.2.1 Nucleation in Microdroplets

**Figure 3:**
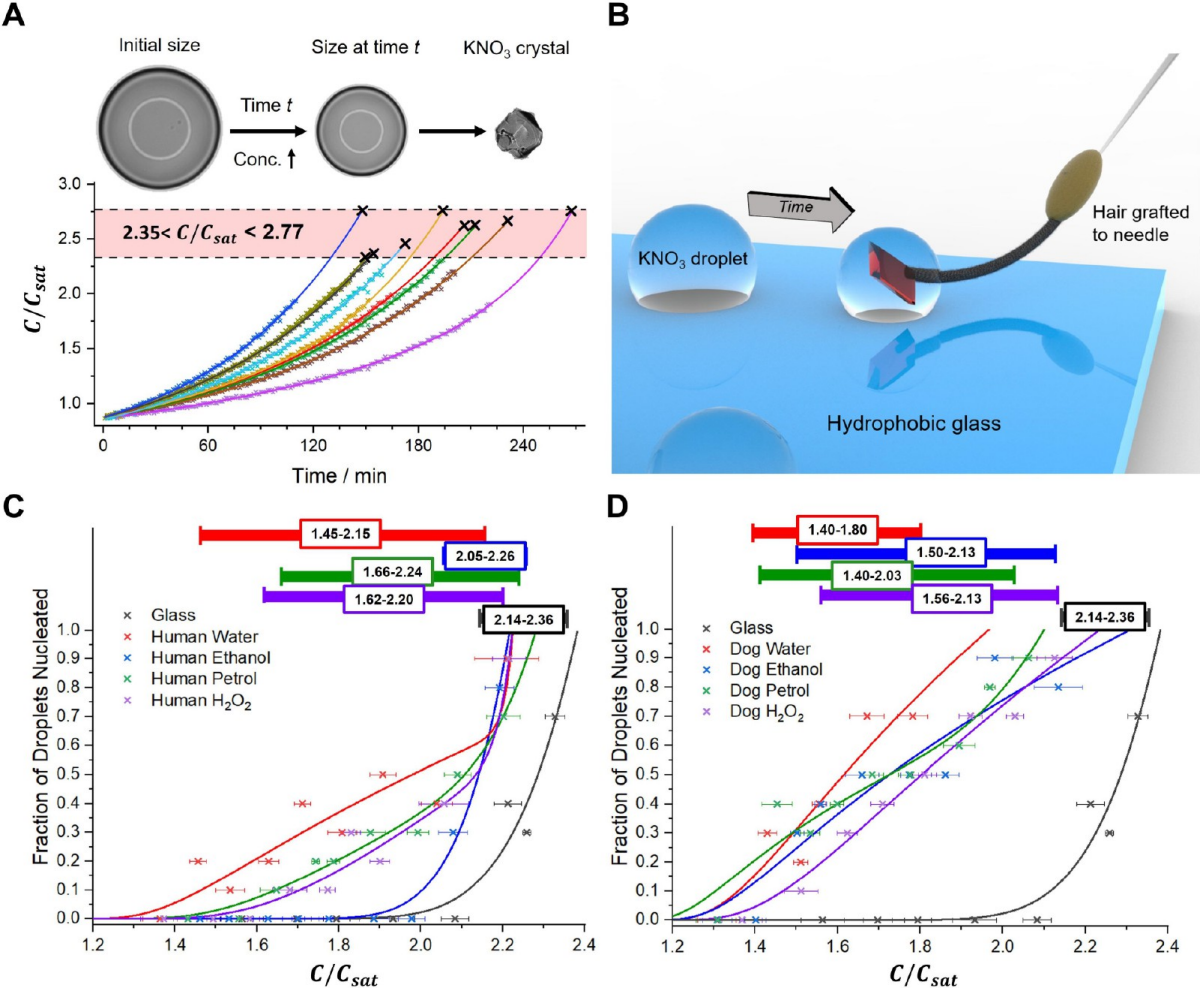
(A) Variation in *C*/*C*_sat_ of nine KNO_3_ microdroplets over time. The black crosses denote the points at which crystallization occurred. (B) Schematic showing how hair was used to nucleate KNO_3_ in the microdroplets. (C/D) Results from KNO_3_ droplet nucleation experiments for (C) human and (D) dog hairs. The bars at the top of the graphs show the ranges between the lowest *C*/*C*_sat_ (onset) nucleation event and the highest *C*/*C*_sat_ at which a droplet did not nucleate (end point). The error bars give the standard deviations of *C*/*C*_sat_.

Crystallization within a 3 × 3 array of KNO_3_ droplets occurred within 2–5 h in the absence of a nucleant, at a rate that was dependent on their position in the array (Figure 3a, Movie S1). The droplet in the center of the array always crystallized last, as it was surrounded by silicone oil with higher water concentration, due to the proximity to neighboring droplets. The droplets used were approximately 130 μm in diameter, which corresponds to a volume of ≈1 nL. Smaller droplets can be made to avoid heterogeneous contaminants to the point where homogeneous nucleation could be accessed in future induction studies.^40^

Human and dog hairs were used as nucleants, and their activities were compared with a glass nanorod as a control. The activities of nucleants were assessed by comparing the proportions of nucleation events that occurred within set ranges of *C*/*C*_sat_. *C*/*C*_sat_ is related to the supersaturation ratio but does not include ion activity coefficients, which are highly challenging to calculate or measure reliably in both supersaturated conditions and high ionic strengths. The glass failed to induce nucleation at *C*/*C*_sat_ > 2.14, but the fraction of nucleated droplets rose sharply above this point until every droplet crystallized at *C*/*C*_sat_ ≥ 2.36 (Figure 3c,d). Notably, all hairs induced nucleation at much lower concentrations. Dog hair consistently nucleated KNO_3_ at lower *C*/*C*_sat_ than human hair, where the onset *C*/*C*_sat_ was 1.40 for water-washed dog hair as compared with 1.45 for human hair, and the highest *C*/*C*_sat_ value for which nucleation failed to occur was 1.80 for dog, compared to 2.15 for human (Figure 3c,d).

This methodology was also used to evaluate how the surface chemistry of the hairs contributes to their activities, where the hairs were also tested after treatment with ethanol, petroleum ether, and hydrogen peroxide to alter the surface chemistry while maintaining the same topography. The efficacy of dog hair as a nucleant was relatively insensitive to surface treatment; the treated hairs exhibited a narrow range of onset *C*/*C*_sat_ (1.40–1.56), and the highest *C*/*C*_sat_ values at which nucleation failed to occur was between 1.80 and 2.13. Water-washed dog hairs were generally the most effective, followed by petroleum ether, ethanol, and then hydrogen peroxide-treated samples. Human hair showed a much wider range of onset *C*/*C*_sat_ (1.45–2.05), which was principally due to the significant increase in onset *C*/*C*_sat_ to 2.05 after ethanol treatment. The highest *C*/*C*_sat_ values at which nucleation failed to occur were similar for all treatments of human hair (2.14–2.26).

Results from the KNO_3_ crystallization experiments were more variable for hair samples than glass, which can be attributed to the higher variability in surface topography and chemistry along the length of each hair. As the droplet diameters were ≈130 μm, the typical spacing between hair cuticle edges is 5–15 μm, and between 10 and 20 cuticle edges were typically exposed to each droplet. In contrast, the relative simplicity of the glass surface is such that the microdroplets experience little variation in the surface topography and chemistry between different samples. Results from these experiments are shown in Figure 3c,d.

With the exception of the ethanol-treated human hair, the shapes of the fitted curves of the fraction of droplets nucleated versus *C*/*C*_sat_ were also quite different for hair when compared with the glass nanorod (Section S2.4). Those for hair generally exhibited a gradual increase in nucleation activity from just above the onset *C*/*C*_sat_, while glass exhibited a steep increase in activity over a narrower range of *C*/*C*_sat_ values. This is consistent with the hairs exhibiting a topographically and chemically complex surface on which a small number of unique nucleation sites gradually become active as supersaturation increases. Human hair behaved similarly but with a slight difference; a transition point exists at moderate *C*/*C*_sat_, where the fitting curves sharply rise (most prominently for water-washed human hair), suggesting that nucleation events shifted from being driven by rare, but highly effective nucleation sites, to less effective but more commonly expressed nucleation sites (Section S2.5).

#### 3.2.2 Potassium Nitrate Polymorphism

**Figure 4:**
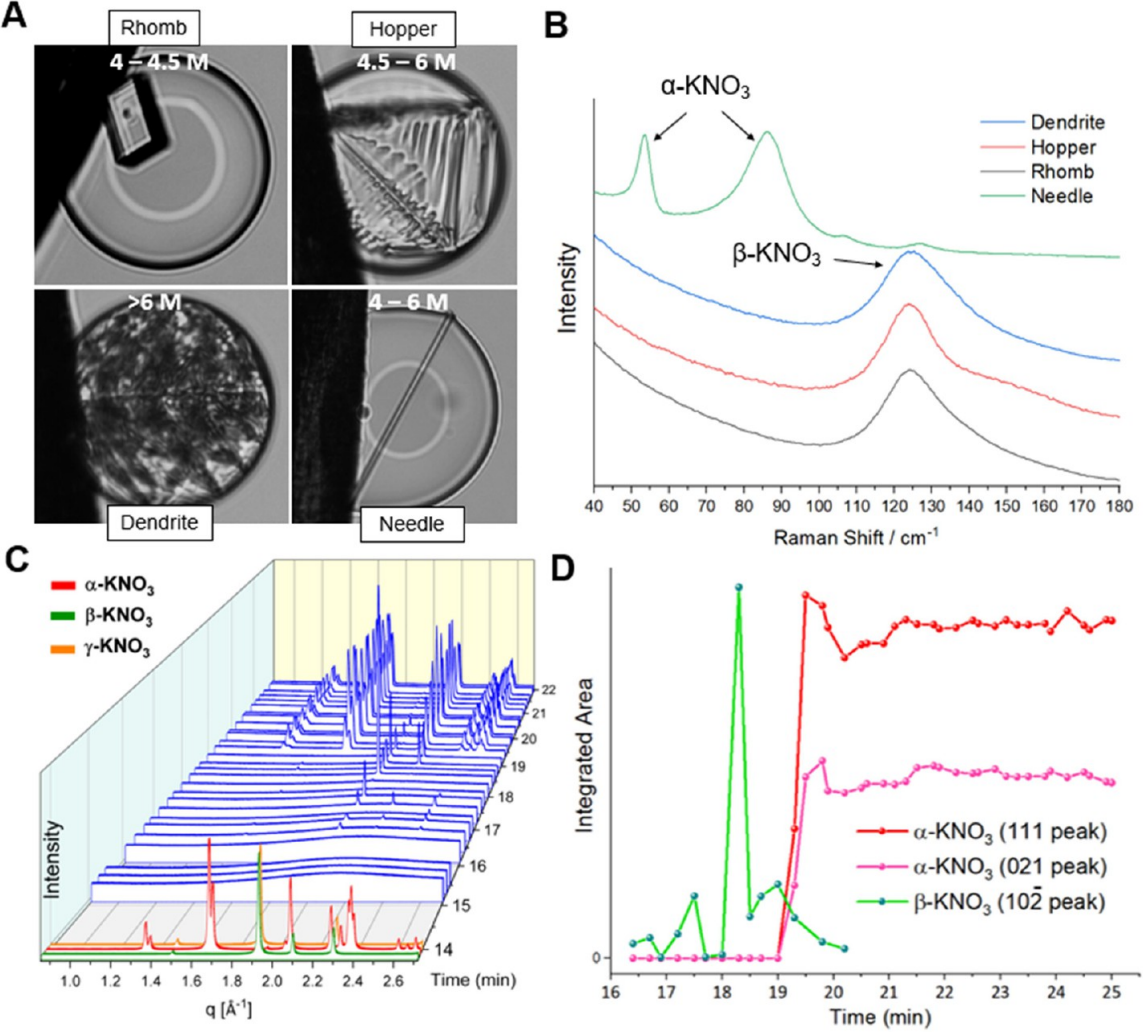
(A) Characteristic morphologies of KNO_3_ crystals and (B) their characterization by Raman spectroscopy. (C) Time-resolved WAXS patterns following evaporation of a levitated KNO_3_ droplet in the presence of a dog hair. (D) Integrated peak areas for α-KNO_3_ and β-KNO_3_ between 16.3 and 20.2 min.

KNO_3_ also exhibits interesting polymorphic behavior, allowing the polymorph selectivity by hair to be evaluated. KNO_3_ exhibits three major polymorphs, termed α (orthorhombic, Pncm), β (rhombohedral, *R*3*m*), and γ (rhombohedral, *R*3̅*m*). γ-KNO_3_ is reportedly only stable at above 90 °C,^41,42^ while α and β are commonly observed at ambient conditions. KNO_3_ saturates at ≈3.2 M with respect to α at room temperature, and at ≈4.0 M with respect to β.^36^ Virtually every crystal produced in the microdroplet experiments was β-KNO_3_, where the crystals were rhombohedral, hopper or dendritic in morphology depending on the concentration (Figure 4a,b). α-KNO_3_ only formed in ≈1% of droplets and was needle-like in form, making these crystals morphologically distinct from the β-phase.

Notably, β-KNO_3_ remained in the droplets when they were allowed to dry completely. This differs from experiments where large volumes of KNO_3_ are allowed to fully evaporate, in which α-KNO_3_ is the sole end product.^30,43^ In situ synchrotron WAXS analysis of KNO_3_ crystallization was carried out in levitated droplets to learn why the formation of α-KNO_3_ was suppressed in our silicon oil bound droplet-based experiments (Section S3).^40^ Droplets of KNO_3_ (2 μL, 0.5 M) were acoustically levitated in the synchrotron X-ray beam path, where they were contacted by a dog hair and allowed to evaporate while collecting data (Figure 4c,d). Three peaks at 1.9, 2.1, and 2.3 Å^–1^ corresponding to β-KNO_3_ formed after 16.3 min, and a set of peaks corresponding to α-KNO_3_ appeared after a further 3 min. The high-intensity (10^–2^) peak of the β-phase was still present when the α-KNO_3_ started to crystallize at 19.3 min, and as β-KNO_3_ is more soluble than α-KNO_3_, dissolution of the β-phase immediately followed α-phase formation. These data show that the metastable β-KNO_3_ phase forms first in both large and small droplets, but that its transformation to the thermodynamically stable α-phase is suppressed in small volumes, as is characteristic of crystallization in confinement.^44^

### 3.3 Surface Chemistry Analysis

**Figure 5:**
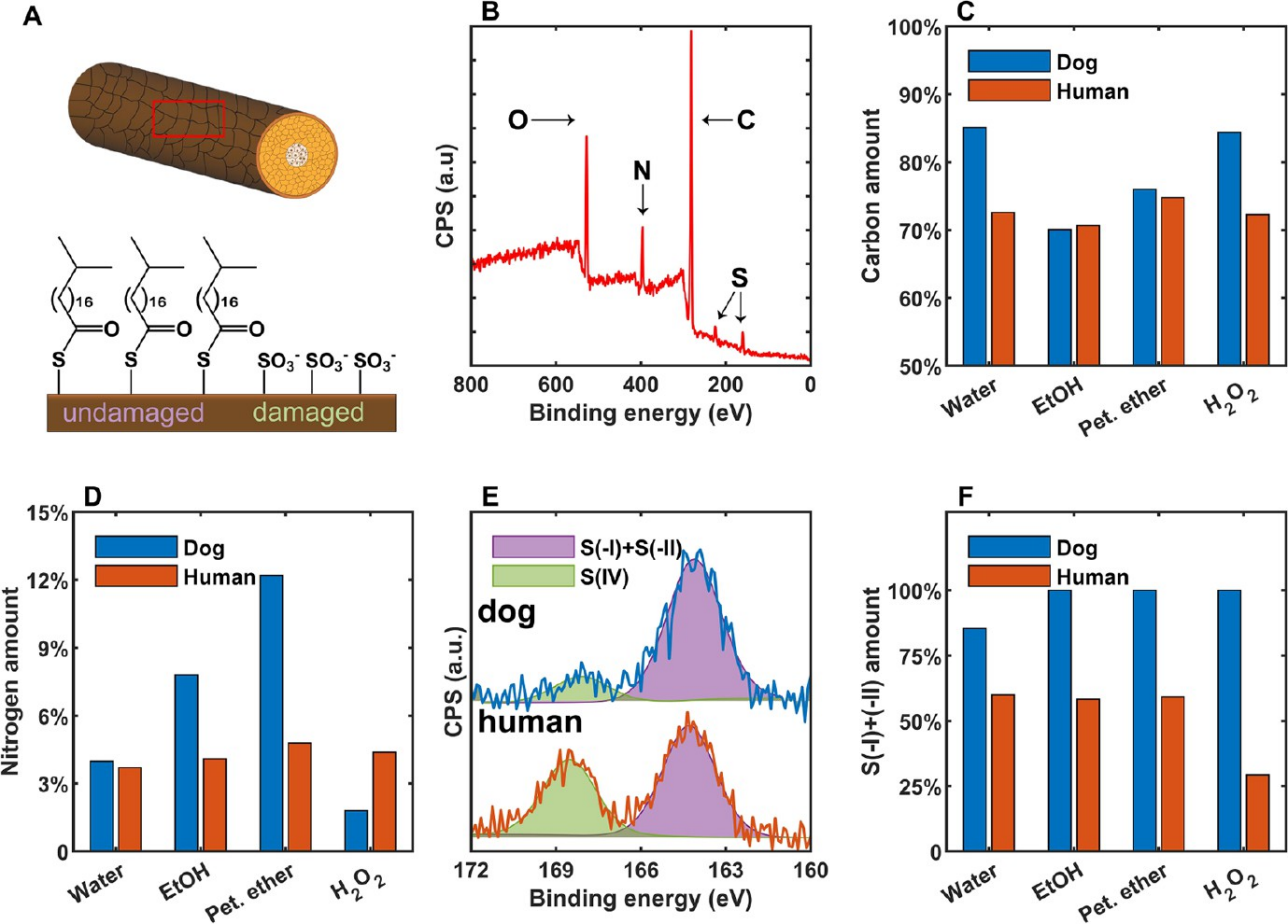
(A) Diagram of the lipid layer at the epicuticle. (B) XPS survey scan for water-washed dog hair. (C) Amount of carbon in the samples quantified from survey scans. (D) Amount of nitrogen in the samples. (E) High-resolution sulfur 2p scans for water-washed dog and human hairs. (F) Amount of S(-I) and S(-II) compared to the total sulfur amount in the samples.

The CaCO_3_ and KNO_3_ crystallization experiments revealed that the different surface modification strategies had marked effects on the nucleating properties of human and dog hairs. The outer surface of the hair is the cuticle layer, which consists of ≈75% highly cross-linked keratin and ≈25% lipids, of which 18-methyleicosanoic acid is the most abundant.^33^ Lipids are either free (unbound) or covalently attached to keratin through thioester linkages (bound). The outermost surface of the cuticle is known as the epicuticle (Figure 5a), an approximately 13 nm layer that contains a large portion of covalently bound lipids.^35^ These lipids readily undergo oxidation and cleavage as a result of excessive washing, mechanical damage, or UV-light exposure.^35,45^

XPS was used to characterize the surface chemistries of the differently treated human and dog hairs and to explore their relationship with the nucleating properties (Section S4.1). Survey scans showed that all hair samples only contained C, N, O, S, and small amounts of Si (contamination from adhesive tape) (Figure 5b, Section S4.2). The amount of carbon detected by XPS provides information about the quantity of lipids present on each hair surface, as the atomic percentage of carbon is 91% for lipids (predominantly 18-methyleicosanoic acid) and 61% for keratin.^46^ These numbers exclude H, as it cannot be detected by XPS directly. The water-washed dog hair exhibited a higher carbon signal than dog hairs treated with ethanol or petroleum ether (Figure 5c), confirming that unbound lipids had been removed by the latter treatments, while hydrogen peroxide treatment does not, at least fully, remove the unbound lipids. These treatments caused little change in the carbon signal of human hair, showing that human hair possessed fewer unbound lipids, likely due to regular washing.

The nitrogen content, in turn, provides information about the amount of protein on the hair surface (mostly keratin). Dog hair exhibits a stronger nitrogen signal when lipids are removed and thus contains a greater density of surface protein than the human hair (Figure 5d). This signal increased after treatment with petroleum ether or ethanol but remained unchanged for human hair, showing that the removal of lipids on the dog hairs exposes more proteins on the surface. Similarly, the nitrogen signal from dog hair decreased after treatment with hydrogen peroxide, which is indicative of loss of protein.^36^ The nitrogen content of the hair samples correlates reasonably well with the number densities of CaCO_3_ crystals formed on their surfaces, which suggests that exposed surface keratin plays a role in the ability to promote nucleation. Moreover, the increased amount of surface exposed proteins for petroleum ether-treated samples compared to that of ethanol-treated ones also supports the synergistic effect observed earlier, where protein denaturation further promotes the nucleation of CaCO_3_.

The degree of keratin oxidation was evaluated from the sulfur 2p signal (Figure 5e, Section S4.3), where sulfur can exist in multiple oxidation states in biological systems.^37^ Thioester (oxidation state −2) and disulfide (−1) groups exhibit overlapping peaks centered at 164.4 eV, whereas sulfonate (+4) groups exhibit a peak at 168.6 eV.^34^ The ratio of the areas of these separate peaks can, therefore, be used to gauge the degree of keratine oxidation (Figure 5f). Water-washed human hair contained substantial numbers of sulfonate groups on the surface, showing that the surface proteins of our as-acquired hair sample were significantly oxidized. These sulfonate groups remained after treatment with ethanol and petroleum ether, and the sample was almost completely oxidized following hydrogen peroxide treatment_._^34^ Very little oxidation was detected on as-acquired or chemically treated dog hair, including samples that had been treated with petroleum ether to remove unbound lipids and then hydrogen peroxide (Section S4.4).^28^ This suggests that dog hair possesses an inherent resistance to oxidation by hydrogen peroxide and that the loss of nucleation efficacy after hydrogen peroxide treatment was not simply related to the protein oxidation state as evaluated from the sulfur moieties, but rather to the removal of proteins from the surface. This could be related to differences in the amino acid composition.

### 3.4 Topography and Surface Charge Mapping

**Figure 6:**
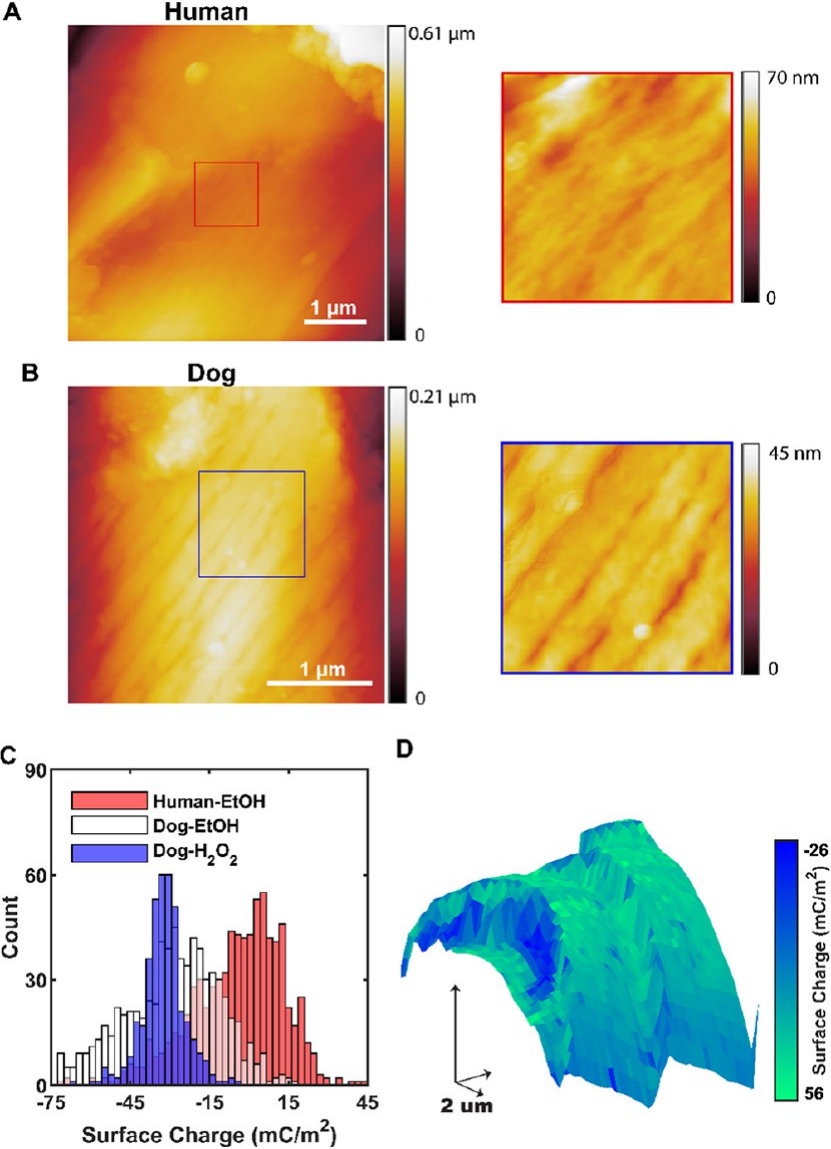
(A/B) AFM topography images of EtOH-treated human and dog hairs, respectively. (C) Surface charge distribution from SICM surface charge mapping experiments. (D) Surface charge/topography map for ethanol-treated dog hair.

Previous reports of enhanced crystal nucleation in cracks, scratches, and crevices^47^ suggest that the surface topography of the hairs may also contribute to their behavior. Ethanol-treated dog and human hairs were selected for study, as there is a large difference in their activities as nucleants. The nanoscale surface topographies of these samples were evaluated using AFM, and clear differences were observed (Section S4.1). The keratin fibrils were aligned and prominent on dog hair, while human hair had a more disordered surface (Figure 6a,b). The macroscale topographies of hair samples are clear from SEM imaging and generally comprise overlapping scales (cuticles) that are 0.5 μm thick, giving rise to distinct topographical steps at their boundaries.

A difference in surface charge at the cuticle edges could also contribute to the activities of these sites as this can produce high local ion concentrations and increased nucleation rates.^48^ Potential-pulse SICM was performed to map the surface charge of ethanol-treated hairs and hydrogen peroxide-treated dog hairs in aqueous solution,^32,49^ which involved using a nanopipette probe to compare the ionic current in bulk solution with that close to the surface, where the ion flow is modulated by the surface charge (Section S5). Comparison with numerical modeling of the ion current then provides surface charge measurements with high spatial resolution (Figure 6c, Section S6).

The modal average magnitude of hair surface charges followed the order human-ethanol < dog-ethanol < dog-hydrogen peroxide (most negative charge density). As no sulfonates were found on dog hairs treated with hydrogen peroxide, this suggests that the lipid removal and/or loss of protein exposes more negatively charged keratin. That ethanol-treated dog hair is more negatively charged than ethanol-treated human hair can be attributed to the higher density of keratin, as shown by XPS. Ethanol-treated dog hair had the broadest distribution of surface charges of the three samples, however, meaning that certain areas of the hair existed that were highly negatively charged. A high spatial resolution surface charge/topography map of an ethanol-treated dog hair (Figure 6d) shows that the edges of some cuticles were substantially more negatively charged than the rest of the hair, implying that these regions also possessed unique surface chemistry.

### 3.5 Broadening the Scope of the Study

**Figure 7:**
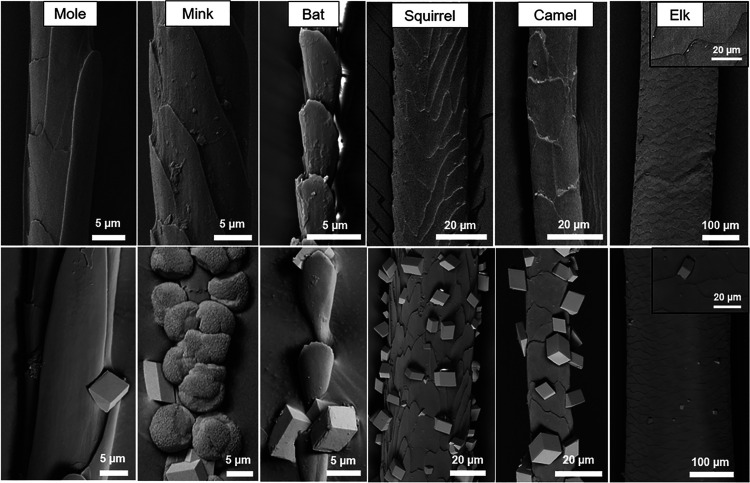
SEM images of (top row) various animal hairs, showing the variety of shape, size, and cuticle structure, and (bottom row) the hairs following immersion in the CaCO_3_ crystallization solution.

Finally, the generality of using hair as an effective nucleant was further explored by investigating the abilities of different types of animal hairs to nucleate CaCO_3_ and of a given hair type to nucleate different minerals. A range of hair samples from different animals were selected for their contrasting morphologies, and their behavior as nucleants for CaCO_3_ was investigated (Figure 7, Section S1.1). The hairs tested varied significantly in their activities. Few crystals nucleated on mole, bat, and elk hairs, while squirrel and camel hairs showed activities similar to those of human and dog hairs. In all cases, the crystals were preferentially located at the edges of the cuticles, where this effect was particularly clear for the mole, bat, and elk hairs due to the scarcity of the crystals. It is striking that crystals formed in comparable locations, i.e., at the cuticle edge, despite the very different sizes and structures of these hairs. The most unusual result, however, came from crystallization of mink hair. While rhombohedral calcite crystals formed on all the other hairs investigated, vaterite was the principal polymorph on mink hair (Section S1.2).

**Figure 8:**
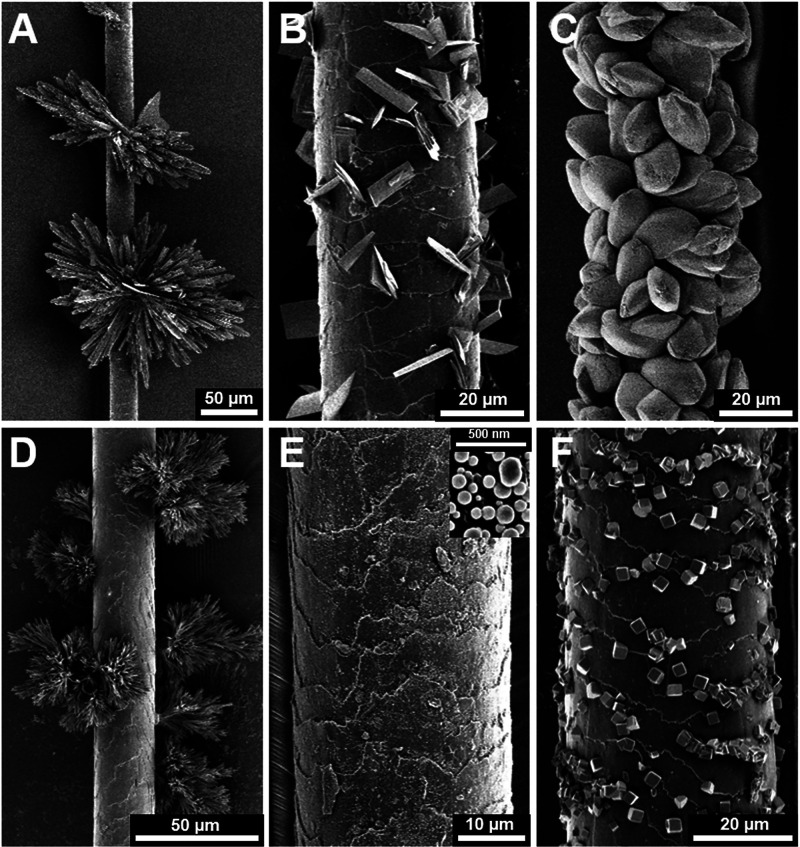
SEM images showing results of crystalli*z*ing (A) CaSO_4_, (B) BaSO_4_, (C) SrSO_4_, (D) BaCO_3_, (E) CuCO_3_, and (F) CaF_2_ on ethanol-treated dog hair.

Dog hair treated with ethanol was an effective nucleant for CaCO_3_ and was therefore explored for its ability to promote the nucleation of other compounds. CaSO_4_, BaSO_4_, SrSO_4_, BaCO_3_, CuCO_3_, and CaF_2_ were selected for the study as they comprise different anions and cations and vary widely in solubility (Figure 8, Section S1.1). Crystals grew on all hair samples, and for SrSO_4_, the hairs were completely encrusted. A preference of the crystals to grow on the cuticle edges was particularly clear for CaF_2_ and CuCO_3_. These data further demonstrate the potential of hair to act as an effective nucleating agent.

## 4 Discussion

Heterogeneous nucleation is a complex process. Most of our current understanding of the behavior of nucleants has come from studies of ice and proteins. In the case of ice, activity is often rationalized by identifying a structural relationship between the nucleating agent and ice, and it is generally accepted that a close lattice match between the two can significantly enhance nucleation rates.^7^ However, this does not guarantee good nucleating ability, as many crystals with good lattice match to ice such as BaF_2_ are ineffective nucleants,^50^ while crystalline steroids can effectively nucleate ice despite lacking a clear structural relationship.^51,52^ The structuring and dynamics of interfacial water and its hydrogen bonding arrangement prior to nucleus formation may play a crucial role^7,53,54^ and is defined by a multitude of factors including temperature, surface charge, chemistry, structure, topography, and polarizability.

For protein nucleation, the surface chemistry of the nucleant can have multiple effects including reducing interfacial energies, as well as adsorbing and aligning molecules from the solution, thereby causing a local increase in supersaturation.^55^ Surface porosity has also been found to enhance protein crystallization by immobilizing and facilitating the accumulation of protein molecules and producing favorable conditions for crystallization.^9,27,56−58^

Although surface chemistry has attracted the lion’s share of attention, there is growing evidence of the role of surface topography in enhancing nucleation. Most surfaces contain features such as scratches and pits, and classical nucleation theory predicts that nucleation is enhanced in concave nanoscale topographical features, provided that the nucleus-substrate contact angle is sufficiently low^59,60^ Surface topography is therefore expected to have the greatest effect on nucleation from vapor,^61−63^ the least on nucleation from a melt,^60^ and nucleation from solution provides an intermediary case.^64−66^ Both surface topography and surface chemistry should therefore be considered when selecting or designing a universal nucleant.

Hair therefore provides an excellent candidate where the edges of the cuticle scales create the type of concave geometries expected to enhance nucleation, and the surface chemistry can readily be modified using simple treatments. Its potential has been demonstrated for proteins^67,68^ and ice,^15^ and our experiments show that it also enhances the nucleation of a variety of soluble and sparingly soluble minerals. Notably, the efficacy is dependent on the hair type, where dog hair is more effective than human hair, and large variations are observed between hair from different mammals. The activity of hair as a nucleant could also be tuned to a wide degree by using surface treatments.

Our experiments provided insight into the origins of this behavior. Considering first the role of surface chemistry, there were significant differences between the surface chemistries of as-acquired human and dog hairs, where dog hair possessed surface lipids and no oxidized sulfur and more surface protein when the unbound lipids are removed. In contrast, the human hair had lost nearly all of its free lipids prior to acquisition, and the sulfur in the surface proteins was significantly oxidized, which can be attributed to regular treatment with shampoos. Treatments that removed lipids (ethanol and petroleum ether) increased the ability of hair to promote CaCO_3_ nucleation—by over four times in the case of ethanol-treated dog hair—but had little effect on KNO_3_. The exception is the ethanol treatment of human hair, which dramatically reduced the ability to nucleate KNO_3_. Hydrogen peroxide treatment, which oxidizes proteins, deactivated both human and dog hairs toward CaCO_3_ nucleation but made little difference to KNO_3_. These results suggest that the protein surface is more active than lipids in nucleating CaCO_3_, and that activity is increased with some denaturing/loss of crystallinity of the protein. This is lost with oxidative damage.

The experiments also indicate that the surface topographies of the hairs contribute to their activities as nucleants, where an investigation of the contrasting CaCO_3_ and KNO_3_ systems provided key information about the nucleation sites. Exfoliation of hairs encrusted with CaCO_3_ crystals showed that the cuticle edges were the key nucleation sites. The dog hair expressed a slightly higher density of cuticle edges than a human hair, which may partly contribute to its higher activity. The KNO_3_ experiments, in contrast, evaluate the concentration ranges at which nucleation is triggered by a hair. This occurs at rare, active sites at low *C*/*C*_sat_, and the active sites increase in number with increasing *C*/*C*_sat,_ and the barrier to nucleation is reduced.

Such preferential nucleation at a few highly active sites is reminiscent of observations of ice nucleation within water droplets on polished feldspar and quartz surfaces,^69^ where high-speed imaging of ice nucleation sites over multiple freeze/thaw cycles showed that nucleation occurred at a tiny handful of surface pits. While it is possible for microcline feldspar to exhibit rare, high-energy faces that possess an epitaxial match to the basal plane of ice, no such correspondence is possible for rose quartz, suggesting that the surface topography dominates its ability to enhance ice nucleation. The topography of the nucleation sites therefore appears to dominate their behavior on the quartz.

We can speculate as to why the cuticle edges are the most effective nucleation sites. Considering geometric effects first, the SEM images show that there is a gap between the overlapping cuticles at the cuticle edges, which could potentially promote nucleation if the dimensions of the confined regions approach the nanoscale. Theoretic consideration of nucleation in convex features shows that if the surface is attractive to a nucleus, the nucleation barrier is significantly reduced as compared with nucleation on a planar surface,^70,71^ and indeed evidence is scattered through the literature to support this.^5,72^

The cuticle edges may also exhibit domains with different chemistries and surface charges to the rest of the surface. The charge on a surface can play a major role in nucleation by increasing the adsorption of ions/molecules from the solution, thereby locally increasing concentration.^73^ Our SICM measurements show that ethanol-treated dog hair possesses a highly nonuniform distribution of charges as compared to ethanol-treated human hair and hydrogen peroxide-treated dog hair and that some cuticle edges are significantly more negatively charged than the rest of the hair surface. The latter may have corresponded to areas where slight exfoliation exposed the inner cuticle. The endocuticle region of the inner cuticle has been shown to express the highly active calcium binding protein S100A3,^74−76^ which contains an extremely high proportion of cysteine residues, making it especially active toward calcium binding.^77^ However, the fact that hair is an effective nucleant for a wide range of compounds suggests that it is not the dominant factor in its behavior.

Given the complexity of the surface of a hair, it is not possible to fully rationalize many of the differences seen in the behavior of the samples investigated. However, our results clearly show that surface chemistry, charge, and topography can act together to enhance crystal nucleation. Further, subtle variations in these properties can lead to significant changes in the ability of a hair to promote nucleation such that sites that can promote nucleation at low supersaturations are rare. This is also consistent with simulations that demonstrate a strong dependence of crystal nucleation rates on the geometry and dimensions of topographical features.^78−80^ As a substrate that naturally exhibits a wide range of surface topographies and chemistries, hair therefore offers an excellent candidate for a universal nucleant.

## 5 Conclusions

The identification of universal crystal nucleants is a long-standing challenge,^11^ where studies focusing on ice and proteins have demonstrated a wide range of mechanisms by which they can operate, emphasizing the complexity of the problem. Inorganic compounds, by comparison, have received relatively attention, where as far as we are aware only montmorillonite,^81^ bioactive glasses, and NX Illite^10^ have yet been reported to be active for CaCO_3_. We have explored hair as a potential universal nucleant and have shown that it can be highly effective for both sparingly and highly soluble inorganic compounds. Its activity depends strongly on the hair source and the nature of the protein state, which can be further modified by chemical treatment. Investigation of the nucleation behavior shows that nucleation occurs preferentially at the cuticle edges, which was especially clear for CaCO_3_, CuCO_3_, and CaF_2_. Combined AFM, XPS, and SICM surface charge mapping suggests that surface chemistry, charge, and topography combine to increase nucleation rates at these locations. With the marked variations in the structure of hair from different animals—which have evolved for purposes as varied as protection from solar UV,^82^ cuts, and grazes, to facilitate perspiration and regulation of body temperature, and reduce friction^83^—hair provides an extensive natural resource of nucleants that exhibit a variety of surface topography and chemistry. Looking beyond hair, this work highlights the role of surface topography as well as surface chemistry in the activity of nucleants, which will ultimately enable us to select or possibly even design active nucleants for target compounds.

## Supporting Information Available

Materials and methods; additional analysis of samples; and surface charge simulation report describing the SICM simulations, including simulation geometry, physical parameters used, and physics applied (PDF)
Shrinking of and subsequent crystallization in KNO_3_ droplets in the absence of a nucleant (MP4)
Behavior of hair as a nucleant toward supersaturated KNO_3_ droplets (MP4)

## Abbreviations

AFM: atomic force microscopy
CNT: classical nucleation theory
SICM: scanning ion conductance microscopy
SEM: scanning electron microscopy
WAXS: wide-angle X-ray scattering
XPS: X-ray photoelectron spectroscopy

## References

[ref1] Braatz R. D. Advanced Control of Crystallization Processes. Annu. Rev. Control 2002, 26, 87–99. 10.1016/S1367-5788(02)80016-5.

[ref2] Chayen N. E.; Saridakis E. Protein Crystallization: From Purified Protein to Diffraction-Quality Crystal. Nat. Methods 2008, 5, 147–153. 10.1038/nmeth.f.203. 18235435

[ref3] Sivaguru M.; Saw J. J.; Wilson E. M.; Lieske J. C.; Krambeck A. E.; Williams J. C.; Romero M. F.; Fouke K. W.; Curtis M. W.; Kear-Scott J. L. Human Kidney Stones: A Natural Record of Universal Biomineralization. Nat. Rev. Urol. 2021, 18, 404–432. 10.1038/s41585-021-00469-x. 34031587

[ref4] Kashchiev D.; Firoozabadi A. Driving Force for Crystallization of Gas Hydrates. J. Cryst. Growth 2002, 241, 220–230. 10.1016/S0022-0248(02)01134-X.

[ref5] Bonafede S. J.; Ward M. D. Selective Nucleation and Growth of an Organic Polymorph by Ledge-Directed Epitaxy on a Molecular Crystal Substrate. J. Am. Chem. Soc. 1995, 117, 7853–7861. 10.1021/ja00135a001.

[ref6] Murray B. J.; O’Sullivan D.; Atkinson J. D.; Webb M. E. Ice Nucleation by Particles Immersed in Supercooled Cloud Droplets. Chem. Soc. Rev. 2012, 41, 6519–6554. 10.1039/c2cs35200a. 22932664

[ref7] Marcolli C.; Nagare B.; Welti A.; Lohmann U. Ice Nucleation Efficiency of AgI: Review and New Insights. Atmos. Chem. Phys. 2016, 16, 8915–8937. 10.5194/acp-16-8915-2016.

[ref8] Saridakis E.; Khurshid S.; Govada L.; Phan Q.; Hawkins D.; Crichlow G. V.; Lolis E.; Reddy S. M.; Chayen N. E. Protein Crystallization Facilitated by Molecularly Imprinted Polymers. Proc. Natl. Acad. Sci. U. S. A. 2011, 108, 11081–11086. 10.1073/pnas.1016539108. 21690356 PMC3131372

[ref9] Khurshid S.; Saridakis E.; Govada L.; Chayen N. E. Porous Nucleating Agents for Protein Crystallization. Nat. Protoc. 2014, 9, 1621–1633. 10.1038/nprot.2014.109. 24922271

[ref10] Levenstein M. A.; Anduix-Canto C.; Kim Y. Y.; Holden M. A.; Nino C. G.; Green D. C.; Foster S. E.; Kulak A. N.; Govada L.; Chayen N. E.; Day S.; Tang C. C.; Weinhausen B.; Burghammer M.; Kapur N.; Meldrum F. C. Droplet Microfluidics XRD Identifies Effective Nucleating Agents for Calcium Carbonate. Adv. Funct. Mater. 2019, 29, 1808172 10.1002/adfm.201808172.

[ref11] Saridakis E.; Chayen N. E. Towards a “universal” Nucleant for Protein Crystallization. Trends Biotechnol. 2009, 27, 99–106. 10.1016/j.tibtech.2008.10.008. 19110330

[ref12] Möhler O.; Georgakopoulos D. G.; Morris C. E.; Benz S.; Ebert V.; Hunsmann S.; Saathoff H.; Schnaiter M.; Wagner R. Heterogeneous Ice Nucleation Activity of Bacteria: New Laboratory Experiments at Simulated Cloud Conditions. Biogeosciences 2008, 5, 1425–1435. 10.5194/bg-5-1425-2008.

[ref13] Voets I. K. From Ice-Binding Proteins to Bio-Inspired Antifreeze Materials. Soft Matter 2017, 13, 4808–4823. 10.1039/C6SM02867E. 28657626 PMC5708349

[ref14] Bar Dolev M.; Braslavsky I.; Davies P. L. Ice-Binding Proteins and Their Function. Annu. Rev. Biochem. 2016, 85, 515–542. 10.1146/annurev-biochem-060815-014546. 27145844

[ref15] Ukichiro, Nakaya. Snow Crystals, Natural and Artificial; Harvard University Press, 1954.

[ref16] D’Arcy A.; Mac Sweeney A.; Haber A. Using Natural Seeding Material to Generate Nucleation in Protein Crystallization Experiments. Acta. Crystallogr., Sect. D: Biol. Crystallogr. 2003, 59, 1343–1346. 10.1107/S0907444903009430. 12832806

[ref17] Thakur A. S.; Robin G.; Guncar G.; Saunders N. F. W.; Newman J.; Martin J. L.; Kobe B. Improved Success of Sparse Matrix Protein Crystallization Screening with Heterogeneous Nucleating Agents. PLoS One 2007, 2, 1091 10.1371/journal.pone.0001091. PMC2034409 17971854

[ref18] Leung C. J.; Nall B. T.; Brayer G. D. CRYSTALLIZATION OF YEAST ISO-2-CYTOCHROME-C USING A NOVEL HAIR SEEDING TECHNIQUE. J. Mol. Biol. 1989, 206, 783–785. 10.1016/0022-2836(89)90585-8. 2544732

[ref19] D’Arcy A.; Mac Sweeney A.; Haber A. Modified Microbatch and Seeding in Protein Crystallization Experiments. J. Synchrotron Radiat. 2004, 11, 24–26. 10.1107/s0909049503023926. 14646125

[ref20] Nederlof I.; Hosseini R.; Georgieva D.; Luo J.; Li D.; Abrahams J. P. A Straightforward and Robust Method for Introducing Human Hair as a Nucleant into High Throughput Crystallization Trials. Cryst. Growth Des. 2011, 11, 1170–1176. 10.1021/cg101374r.

[ref21] Aizenberg J.; Black A. J.; Whitesides G. M. Control of Crystal Nucleation by Patterned Self-Assembled Monolayers. Nature 1999, 398, 495–498. 10.1038/19047.

[ref22] Küther J.; Seshadri R.; Knoll W.; Tremel W. Templated Growth of Calcite, Vaterite and Aragonite Crystals on Self-Assembled Monolayers of Substituted Alkylthiols on Gold. J. Mater. Chem. 1998, 8, 641–650. 10.1039/a705859d.

[ref23] Artusio F.; Fumagalli F.; Valsesia A.; Ceccone G.; Pisano R. Role of Self-Assembled Surface Functionalization on Nucleation Kinetics and Oriented Crystallization of a Small-Molecule Drug: Batch and Thin-Film Growth of Aspirin as a Case Study. ACS Appl. Mater. Interfaces 2021, 13, 15847–15856. 10.1021/acsami.1c00460. 33759495 PMC8041258

[ref24] Holbrough J. L.; Campbell J. M.; Meldrum F. C.; Christenson H. K. Topographical Control of Crystal Nucleation. Cryst. Growth Des. 2012, 12, 750–755. 10.1021/cg201084j.

[ref25] Holden M. A.; Whale T. F.; Tarn M. D.; O’Sullivan D.; Walshaw R. D.; Murray B. J.; Meldrum F. C.; Christenson H. K. High-Speed Imaging of Ice Nucleation in Water Proves the Existence of Active Sites. Sci. Adv. 2019, 5, 1–11. 10.1126/sciadv.aav4316. PMC6358314 30746490

[ref26] Whale T. F.; Holden M. A.; Kulak A. N.; Kim Y. Y.; Meldrum F. C.; Christenson H. K.; Murray B. J. The Role of Phase Separation and Related Topography in the Exceptional Ice-Nucleating Ability of Alkali Feldspars. Phys. Chem. Chem. Phys. 2017, 19, 31186–31193. 10.1039/C7CP04898J. 29139499 PMC11970471

[ref27] Chayen N. E.; Saridakis E.; El-Bahar R.; Nemirovsky Y. Porous Silicon: An Effective Nucleation-Inducing Material for Protein Crystallization. J. Mol. Biol. 2001, 312, 591–595. 10.1006/jmbi.2001.4995. 11575916

[ref28] Meldrum F. C.; Cölfen H. Controlling Mineral Morphologies and Structures in Biological and Synthetic Systems. Chem. Rev. 2008, 108, 4332–4432. 10.1021/cr8002856. 19006397

[ref29] Kracek F. C. The Polymorphism of Potassium Nitrate. J. Phys. Chem. 1930, 34, 225–247. 10.1021/j150308a001.

[ref30] Linnikov O. D.; Rodina I. V.; Grigorov I. G.; Polyakov E. V. Kinetics and Mechanism of Spontaneous Crystallization of Potassium Nitrate from Its Supersaturated Aqueous Potassium Nitrate from Its Supersaturated Aqueous Solutions. Cryst. Struct. Theory Appl. 2013, 02, 16–27. 10.4236/csta.2013.21003.

[ref31] Reddy M. M.; Nancollas G. H. The Crystallization of Calcium Carbonate. IV. The Effect of Magnesium, Strontium and Sulfate Ions. J. Cryst. Growth 1976, 35, 33–38. 10.1016/0022-0248(76)90240-2.

[ref32] Maddar F. M.; Perry D.; Brooks R.; Page A.; Unwin P. R. Nanoscale Surface Charge Visualization of Human Hair. Anal. Chem. 2019, 91, 4632–4639. 10.1021/acs.analchem.8b05977. 30807113

[ref33] Gershbein L. L.; Baburao K. Multiple Discriminant Analysis of Fatty Acids from Male Scalp Hair Lipids. Fette, Seifen, Anstrichm. 1984, 86, 121–128. 10.1002/lipi.19840860310.

[ref34] Okamoto M.; Ishikawa K.; Tanji N.; Aoyagi S. Investigation of the Damage on the Outermost Hair Surface Using ToF-SIMS and XPS. Surf. Interface Anal. 2012, 44, 736–739. 10.1002/sia.3878.

[ref35] Robbins C. R. Chemical and Physical Behavior of Human Hair V5; Springer Science & Business Media, 2012.

[ref36] Laval P.; Giroux C.; Leng J.; Salmon J. B. Microfluidic Screening of Potassium Nitrate Polymorphism. J. Cryst. Growth 2008, 310, 3121–3124. 10.1016/j.jcrysgro.2008.03.009.

[ref37] Cedeno R.; Grossier R.; Candoni N.; Levernier N.; Flood A.; Veesler S. CNT Effective Interfacial Energy and Pre-Exponential Kinetic Factor from Measured NaCl Crystal Nucleation Time Distributions in Contracting Microdroplets, 2023, (Preprint) arXiv:2301.11088 submitted: Jan 2023. 10.1063/5.0143704 37191406

[ref38] Grossier R.; Hammadi Z.; Morin R.; Veesler S. Predictive Nucleation of Crystals in Small Volumes and Its Consequences. Phys. Rev. Lett. 2011, 107, 025504 10.1103/PhysRevLett.107.025504. 21797619

[ref39] Hammadi Z.; Candoni N.; Grossier R.; Ildefonso M.; Morin R.; Veesler S. Small-Volume Nucleation. C. R. Phys. 2013, 14, 192–198. 10.1016/j.crhy.2012.12.004.

[ref40] Selzer D.; Tüllmann N.; Kiselev A.; Leisner T.; Kind M. Investigation of Crystal Nucleation of Highly Supersaturated Aqueous KNO3 Solution from Single Levitated Droplet Experiments. Cryst. Growth Des. 2018, 18, 4896–4905. 10.1021/acs.cgd.7b01778.

[ref41] Jurado-Lasso F.; Jurado-Lasso N.; Ortiz J.; Jurado J. F. Thermal Dielectric and Raman Studies on the KNO3 Compound High-Temperature Region. Dyna 2016, 83, 244–249. 10.15446/dyna.v83n198.54693.

[ref42] Nimmo J. K.; Lucas B. W. The Crystal Structures of γ- and β-KNO 3 and the α ← γ ← β Phase Transformations. Acta Crystallogr., Sect. B: Struct. Crystallogr. Cryst. Chem. 1976, 32, 1968–1971. 10.1107/S0567740876006894.

[ref43] Linnikov O. D.; Grigorov I. G.; Rodina I. V.; Polyakov E. V. Mechanism of Potassium Nitrate Crystal Intergrowth during Spontaneous Crystallization from Supersaturated Aqueous Solutions. Dokl. Phys. Chem. 2011, 439, 135–138. 10.1134/S0012501611070049.

[ref44] Freney E. J.; Garvie L. A. J.; Groy T. L.; Buseck P. R. Growth and Single-Crystal Refinement of Phase-III Potassium Nitrate, KNO 3. Acta Crystallogr., Sect. B: Struct. Sci. 2009, 65, 659–663. 10.1107/S0108768109041019. 19923693

[ref45] Robbins C. R.; Bahl M. K. Analysis of Hair by Electron Spectroscopy for Chemical Analysis. J. Soc. Cosmet. Chem. 1984, 35, 379–390.

[ref46] Sinha P.; Yadav A.; Tyagi A.; Paik P.; Yokoi H.; Naskar A. K.; Kuila T.; Kar K. K. Keratin-Derived Functional Carbon with Superior Charge Storage and Transport for High-Performance Supercapacitors. Carbon 2020, 168, 419–438. 10.1016/j.carbon.2020.07.007.

[ref47] Grosfils P.; Lutsko J. F. Impact of Surface Roughness on Crystal Nucleation. Crystals 2021, 11, 4 10.3390/cryst11010004.

[ref48] Finney A. R.; McPherson I. J.; Unwin P. R.; Salvalaglio M. Electrochemistry, Ion Adsorption and Dynamics in the Double Layer: A Study of NaCl(Aq) on Graphite. Chem. Sci. 2021, 12, 11166–11180. 10.1039/D1SC02289J. 34522314 PMC8386640

[ref49] Page A.; Perry D.; Young P.; Mitchell D.; Frenguelli B. G.; Unwin P. R. Fast Nanoscale Surface Charge Mapping with Pulsed-Potential Scanning Ion Conductance Microscopy. Anal. Chem. 2016, 88, 10854–10859. 10.1021/acs.analchem.6b03744. 27774792

[ref50] Conrad P.; Ewing G. E.; Karlinsey R. L.; Sadtchenko V. Ice Nucleation on BaF2(111). J. Chem. Phys. 2005, 122, 11 10.1063/1.1844393. 15740398

[ref51] Head R. B. Steroids as Ice Nucleators. Nature 1961, 191, 1058–1059. 10.1038/1911058a0. 13712571

[ref52] Sosso G. C.; Whale T. F.; Holden M. A.; Pedevilla P.; Murray B. J.; Michaelides A. Unravelling the Origins of Ice Nucleation on Organic Crystals. Chem. Sci. 2018, 9, 8077–8088. 10.1039/C8SC02753F. 30542556 PMC6238755

[ref53] Fukuta N.; Mason B. J. Epitaxial Growth of Ice on Organic Crystals. J. Phys. Chem. Solids 1963, 24, 715–718. 10.1016/0022-3697(63)90217-8.

[ref54] Atkinson J. D.; Murray B. J.; Woodhouse M. T.; Whale T. F.; Baustian K. J.; Carslaw K. S.; Dobbie S.; O’Sullivan D.; Malkin T. L. The Importance of Feldspar for Ice Nucleation by Mineral Dust in Mixed-Phase Clouds. Nature 2013, 498, 355–358. 10.1038/nature12278. 23760484

[ref55] Tsekova D. S.; Williams D. R.; Heng J. Y. Y. Effect of Surface Chemistry of Novel Templates on Crystallization of Proteins. Chem. Eng. Sci. 2012, 77, 201–206. 10.1016/j.ces.2012.01.049.

[ref56] Nanev C. N.; Saridakis E.; Chayen N. E. Protein Crystal Nucleation in Pores. Sci. Rep. 2017, 7, 35821 10.1038/srep35821. 28091515 PMC5238398

[ref57] Nanev C.; Govada L.; Chayen N. E. Theoretical and Experimental Investigation of Protein Crystal Nucleation in Pores and Crevices. IUCrJ. 2021, 8, 270–280. 10.1107/S2052252521000269. 33708403 PMC7924239

[ref58] Chayen N. E.; Saridakis E.; Sear R. P. Experiment and Theory for Heterogeneous Nucleation of Protein Crystals in a Porous Medium. Proc. Natl. Acad. Sci. U. S. A. 2006, 103, 597–601. 10.1073/pnas.0504860102. 16407115 PMC1334630

[ref59] Turnbull D. Kinetics of Heterogeneous Nucleation. J. Chem. Phys. 1950, 18, 198–203. 10.1063/1.1747588.

[ref60] Campbell J. M.; Meldrum F. C.; Christenson H. K. Is Ice Nucleation from Supercooled Water Insensitive to Surface Roughness?. J. Phys. Chem. C 2015, 119, 1164–1169. 10.1021/jp5113729.

[ref61] Campbell J. M.; Meldrum F. C.; Christenson H. K. Characterization of Preferred Crystal Nucleation Sites on Mica Surfaces. Cryst. Growth Des. 2013, 13, 1915–1925. 10.1021/cg301715n.

[ref62] Holbrough J. L.; Campbell J. M.; Meldrum F. C.; Christenson H. K. Topographical Control of Crystal Nucleation. Cryst. Growth Des. 2012, 12, 750–755. 10.1021/cg201084j.

[ref63] Kovács T.; Meldrum F. C.; Christenson H. K. Crystal Nucleation without Supersaturation. J. Phys. Chem. Lett. 2012, 3, 1602–1606. 10.1021/jz300450g. 26285715

[ref64] Asanithi P. Surface Porosity and Roughness of Micrographite Film for Nucleation of Hydroxyapatite. J. Biomed. Mater. Res., Part A 2014, 102, 2590–2599. 10.1002/jbm.a.34930. 24038761

[ref65] Wu H.; Yao X.; Gui Y.; Hao H.; Yu L. Surface Enhancement of Crystal Nucleation in Amorphous Acetaminophen. Cryst. Growth Des. 2022, 22, 5598–5606. 10.1021/acs.cgd.2c00695.

[ref66] Delmas T.; Roberts M. M.; Heng J. Y. Y. Nucleation and Crystallization of Lysozyme: Role of Substrate Surface Chemistry and Topography. J. Adhes. Sci. Technol. 2011, 25, 357–366. 10.1163/016942410X525614.

[ref67] Georgieva D. G.; Kuil M. E.; Oosterkamp T. H.; Zandbergen H. W.; Abrahams J. P. Heterogeneous Nucleation of Three-Dimensional Protein Nanocrystals. Acta Crystallogr., Sect. D: Biol. Crystallogr. 2007, 63, 564–570. 10.1107/S0907444907007810. 17452781

[ref68] D’Arcy A.; Mac Sweeney A.; Haber A. Using Natural Seeding Material to Generate Nucleation in Protein Crystallization Experiments. Acta Crystallogr., Sect. D: Biol. Crystallogr. 2003, 59, 1343–1346. 10.1107/S0907444903009430. 12832806

[ref69] Holden M. A.; Whale T. F.; Tarn M. D.; O’Sullivan D.; Walshaw R. D.; Murray B. J.; Meldrum F. C.; Christenson H. K. High-Speed Imaging of Ice Nucleation in Water Proves the Existence of Active Sites. Sci. Adv. 2019, 5, 1–11. 10.1126/sciadv.aav4316. PMC6358314 30746490

[ref70] Sholl C. A.; Fletcher N. H. Decoration Criteria for Surface Steps. Acta Metall. 1970, 18, 1083–1086. 10.1016/0001-6160(70)90006-4.

[ref71] Chakraverty B. K.; Pound G. M. Heterogeneous Nucleation at Macroscopic Steps. Acta Metall. 1964, 12, 851–860. 10.1016/0001-6160(64)90143-9.

[ref72] Campbell J. M.; Meldrum F. C.; Christenson H. K. Characterization of Preferred Crystal Nucleation Sites on Mica Surfaces. Cryst. Growth Des. 2013, 13, 1915–1925. 10.1021/cg301715n.

[ref73] Smeets P. J. M.; Cho K. R.; Kempen R. G. E.; Sommerdijk N.; De Yoreo J. J. Calcium Carbonate Nucleation Driven by Ion Binding in a Biomimetic Matrix Revealed by in Situ Electron Microscopy. Nat. Mater. 2015, 14, 394–399. 10.1038/nmat4193. 25622001

[ref74] Rogers G. E. Known and Unknown Features of Hair Cuticle Structure: A Brief Review. Cosmetics 2019, 6, 32 10.3390/cosmetics6020032.

[ref75] Kizawa K.; Uchiwa H.; Murakami U. Highly-Expressed S100A3, a Calcium-Binding Protein, in Human Hair Cuticle. Biochim. Biophys. Acta, Mol. Cell Res. 1996, 1312, 94–98. 10.1016/0167-4889(96)00023-7. 8672544

[ref76] Kizawa K.; Troxler H.; Kleinert P.; Inoue T.; Toyoda M.; Morohashi M.; Heizmann C. W. Characterization of the Cysteine-Rich Calcium-Binding S100A3 Protein from Human Hair Cuticles. Biochem. Biophys. Res. Commun. 2002, 299, 857–862. 10.1016/S0006-291X(02)02744-4. 12470658

[ref77] Smart K. E.; Kilburn M.; Schroeder M.; Martin B. G. H.; Hawes C.; Marsh J. M.; Grovenor C. R. M. Copper and Calcium Uptake in Colored Hair. J. Cosmet. Sci. 2009, 60, 337–345. 10.1111/j.1468-2494.2010.00549_3.x. 19586601

[ref78] Page A. J.; Sear R. P. Crystallization Controlled by the Geometry of a Surface. J. Am. Chem. Soc. 2009, 131, 17550–17551. 10.1021/ja9085512. 19911797

[ref79] Page A. J.; Sear R. P. Heterogeneous Nucleation in and out of Pores. Phys. Rev. Lett. 2006, 97, 065701 10.1103/PhysRevLett.97.065701. 17026175

[ref80] Bi Y.; Cao B.; Li T. Enhanced Heterogeneous Ice Nucleation by Special Surface Geometry. Nat. Commun. 2017, 8, 15372 10.1038/ncomms15372. 28513603 PMC5442314

[ref81] Kralj D.; Vdovic N. The Influence of Some Naturally Occurring Minerals on the Precipitation of Calcium Carbonate Polymorphs. Water Res. 2000, 34, 179–184. 10.1016/S0043-1354(99)00110-4.

[ref82] De Gálvez M. V.; Aguilera J.; Bernabõ J. L.; Sánchez-Roldán C.; Herrera-Ceballos E. Human Hair as a Natural Sun Protection Agent: A Quantitative Study. Photochem. Photobiol. 2015, 91, 966–970. 10.1111/php.12433. 25682789

[ref83] Chernova O. F.; Zherebtsova O. V. A Comparative SEM Study of the Guard Hair Architecture in Subterranean Moles (Talpidae, Soricomorpha), Golden Moles (Chrysochloridae, Afrosoricidae), and Silvery Mole-Rats (Bathyergidae, Rodentia). Zool. Anz. 2022, 301, 59–75. 10.1016/j.jcz.2022.09.005.

[ref84] Dunn T. H.; Skaanvik S. A.; McPherson I. J.; O’Shaughnessy C.; He X.; Kulak A. N.; Micklethwaite S.; Matamoros-Veloza A.; Sandei I.; Hunter L.; Turner T. D.; Galloway J. M.; Rosenthal M.; Britton A. J.; Walker M.; Dong M.; Unwin P. R.; Meldrum F. C. Dataset for The Universality of Hair as a Nucleant: Exploring the Effects of Surface Chemistry and Topography. 2023, https://doi.org/10.5518/1382. 10.1021/acs.cgd.3c01035 PMC10704409 38076525

