# Supplementary material for: Universality of Hair as a Nucleant: Exploring the Effects of Surface Chemistry and Topography
Source: Cryst Growth Des. 2023 Nov 11;23(12):8978–90. doi: 10.1021/acs.cgd.3c01035 (PMC10704409; doi:10.1021/acs.cgd.3c01035)
Supplement: Supplementary file 1 — cg3c01035_si_001.pdf [file cg3c01035_si_001.pdf]

The Universality of Hair as a Nucleant: Exploring the Effects of Surface Chemistry and Topography

Thomas H. Dunn, Sebastian. A. Skaanvik, Ian J. McPherson, Cedrick O'Shaughnessy, Xuefeng He, Alexander N. Kulak, Stuart Micklethwaite, Adriana Matamoros-Veloza, Ilaria Sandei, Liam Hunter, Thomas D. Turner, Johanna M. Galloway, Martin Rosenthal, Andrew J. Britton, Marc Walker, Mingdong Dong, Patrick R. Unwin, Fiona C. Meldrum

1. $CaCO_3$ crystallization experiments

1.1. Experimental

Chemical treatment of hair samples Hair samples were cut into 1.5 cm sections before treatment. For either water washing, ethanol or petrol treatment, samples were immersed in either Milli-Q water, absolute ethanol (99.9%, VWR Chemicals) or petroleum ether (40-60 °C, Sigma-Aldrich) for 15 minutes, then the liquid was decanted away and replaced. This was repeated for 3 washing cycles. For ethanol and petrol treatment, the hair sections were allowed to evaporate to dryness and then immersed in water to give the samples time to wet before use in an experiment. For hydrogen peroxide treatment, hair samples were firstly immersed in water for 15 minutes to improve wettability, and then immersed in hydrogen peroxide (30 wt. %) for 30 minutes. The samples were then subjected to 3 water washing cycles, and left in water until used in experiments.

Fabrication of hydrophobic glass substrates Borosilicate glass slides (Scientific Glass Laboratories Ltd) were washed with isopropyl alcohol (99.9%, Sigma-Aldrich) and air dried, then placed in piranha solution (a 3:1 mixture of H_2SO_4 (>95%, Fisher Scientific) and hydrogen peroxide (>30% w/v, Fisher Scientific)) for 2 hours (Caution! Piranha solution is a strong oxidizing agent, which reacts violently

with most organic materials and must be handled with extreme care). The slides were washed with copious amounts of deionised water, followed by isopropyl alcohol. They were then treated with Aquapel (PGW Auto Glass) for 1 minute. The slide was removed, washed with isopropyl alcohol, gently polished with a lint-free wipe followed by a final washing step with isopropyl alcohol and air drying.

Crystallization of sparingly soluble minerals Borosilicate glass slides (Scientific Glass Laboratories Ltd) were treated with piranha solution and washed by the same procedure outlined in the previous section. Glass slides and treated hair samples were immersed in a 2.5 mL of a primary solution, to which 2.5 mL of a secondary solution was added. Details of these solutions and the length of time for which crystallization was allowed to proceed are shown in **Error! Reference source not found.**. After crystallization was complete, hairs were washed gently with water and isopropyl alcohol, then air dried.

Mineral	Solution 1	Solution 2	Crystallization time
$CaCO_3$	$CaCl_2$ (5 mM)	Na_2CO_3 (5 mM)	20 minutes
$CaSO_4$	$CaCl_2$ (600 mM)	Na_2SO_4 (600 mM)	5 minutes
$BaSO_4$	$BaCl_2$ (0.5 mM)	Na_2SO_4 (0.5 mM)	2 hours
$SrSO_4$	$SrCl_2$ (50 mM)	Na_2SO_4 (50 mM)	5 minutes
CaF_2	$CaCl_2$ (6 mM)	NaF (12 mM)	30 minutes
$BaCO_3$	$BaCl_2$ (2 mM)	Na_2CO_3 (2 mM)	1 hour
$CuCO_3$	$CuCl_2$ (10 mM)	Na_2CO_3 (10 mM)	1 hour

Table S1. List of solution concentrations and crystallization times used.

Methodology for resin exfoliation of hairs Demotec 70 conductive carbon resin (Agar Scientific) was mixed and allowed to partially cure. Hairs encrusted with $CaCO_3$ crystals were placed on top, and the

resin was left for 20 minutes to cure completely, after which the hairs were gently pulled away from the surface.

Raman spectroscopy of hair samples Raman spectra were collected from crystals embedded in hairs using a Horiba LabRAM HR Evolution Raman microscope with a 50 W 532 nm (green) laser passing through an edge filter. A hole size of 50 µm and laser powers of 1-10% were used, and the grating was 1800 grooves/mm, and 5 s per scan and the data were averaged over 2 passes.

1.2. Characterization of $CaCO_3$ by Raman spectroscopy

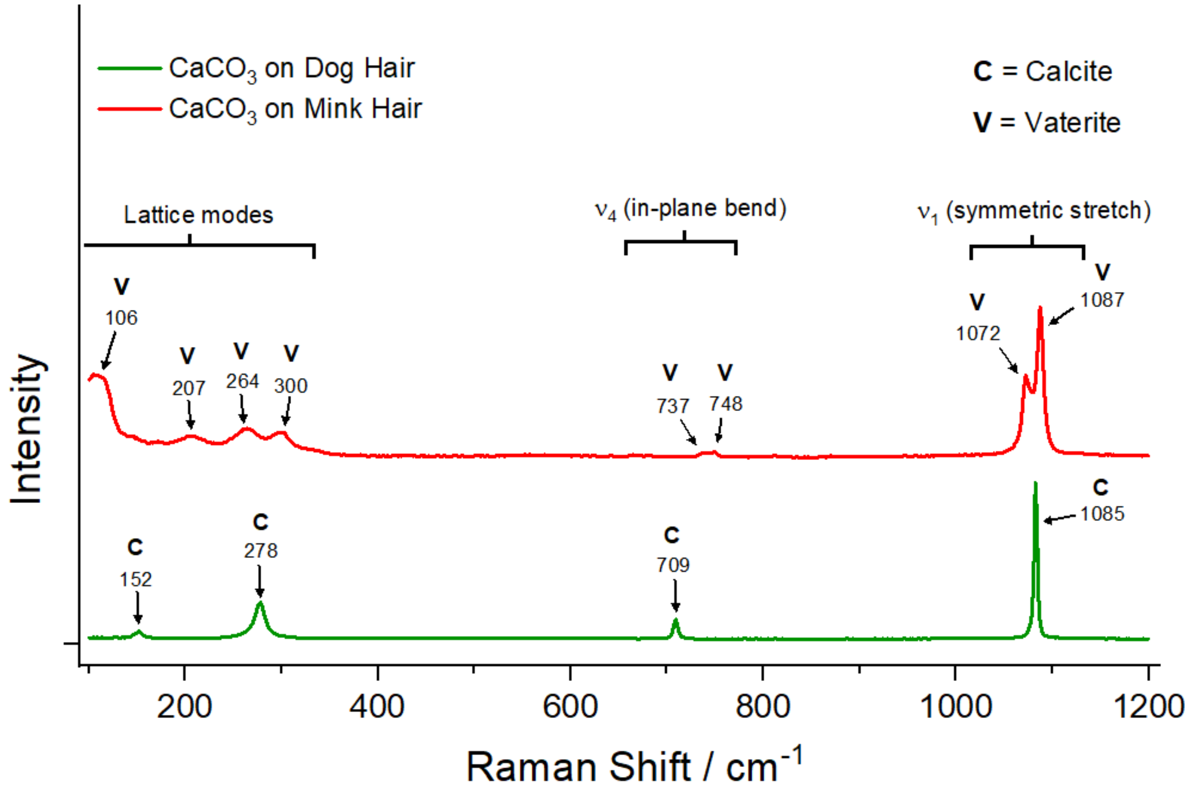

Figure S1. Raman spectrum showing representative scans of crystals encrusted on dog hair (calcite) and mink hair (vaterite)

1.3. Calculation of number density on glass substrates and hair samples

For glass substrates, optical microscopy images shown in Figure S2 were converted to binary images using ImageJ, and the 'Analyze Particles' tool was used to calculate the number of particles, which was divided by the area of view. Values from three different images were used to calculate the final value and standard deviation. For hair samples, the crystal number density was calculated by Equation S1, assuming a cylindrical hair geometry:

$$Number\ density = \frac{N_{crystals}}{\pi r l} \qquad \textit{Eq S1}$$

where *r* is the radius (half of width) and *l* is the length of the hair.

1.3.1 Glass substrates

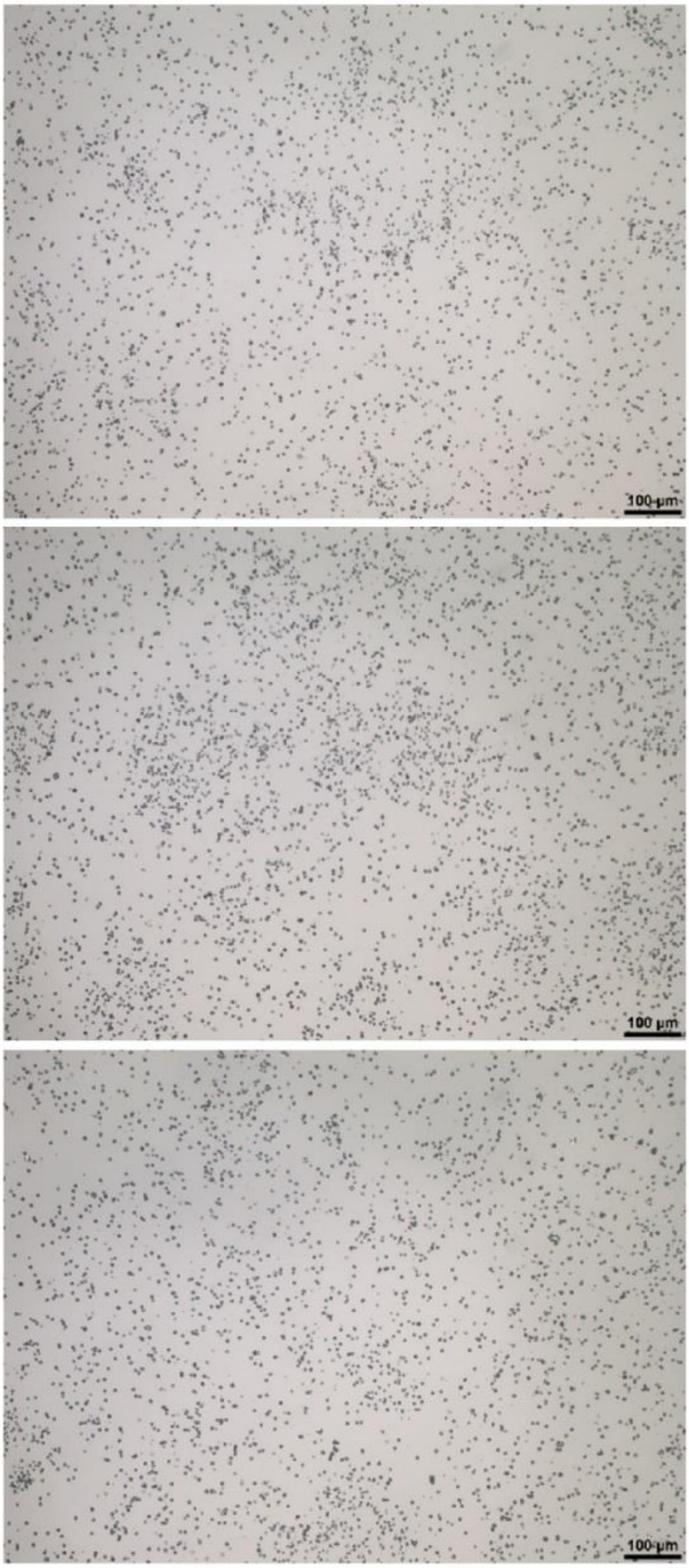

Figure S2. Optical micrographs of $CaCO_3$ on glass used to calculate $CaCO_3$ nucleation densities.

1.3.2 Dog hair – water washed

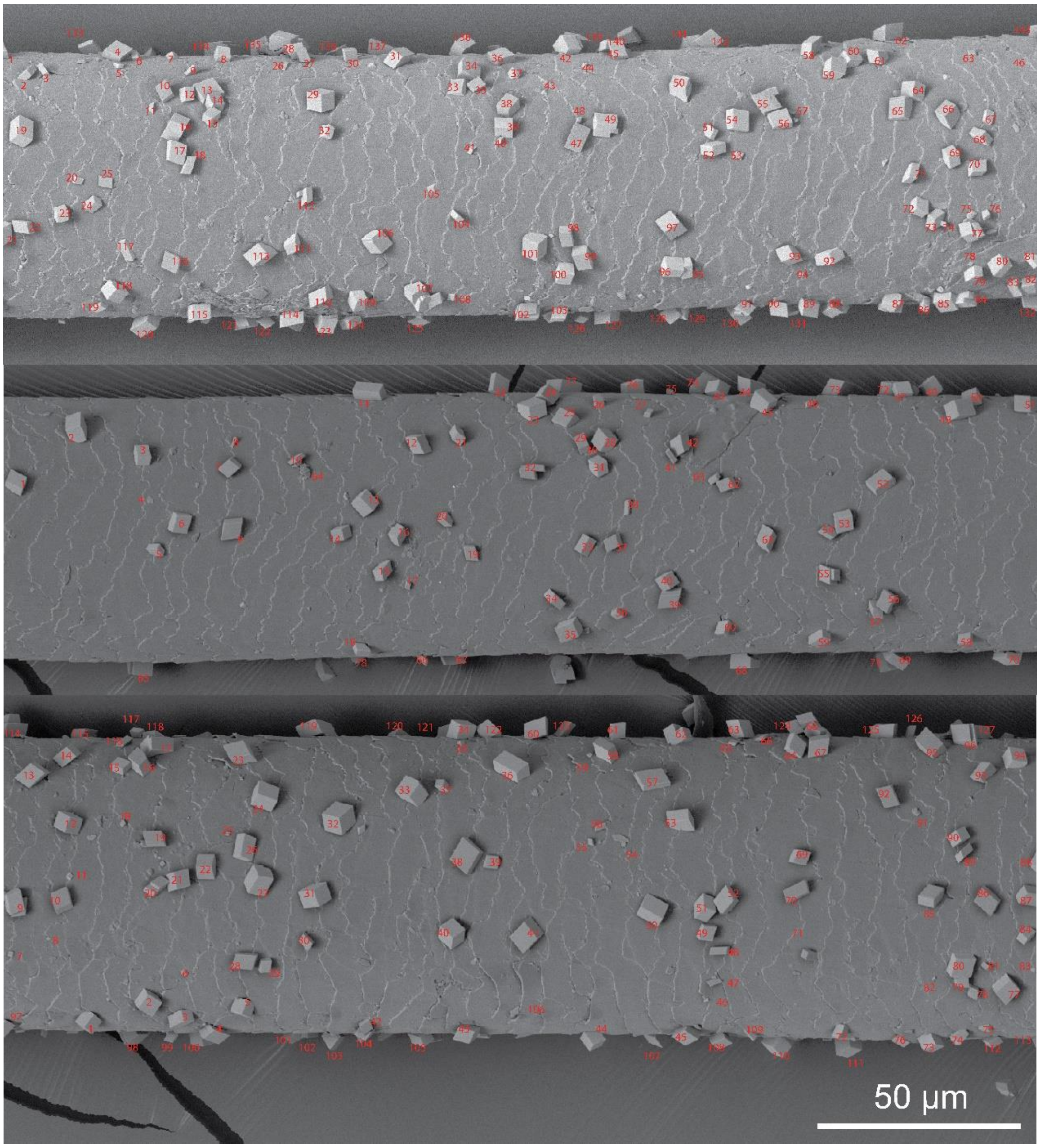

Figure S3. Three replicates of crystal number density measurements for water washed dog hair.

1.3.3 Dog hair – petroleum ether treated

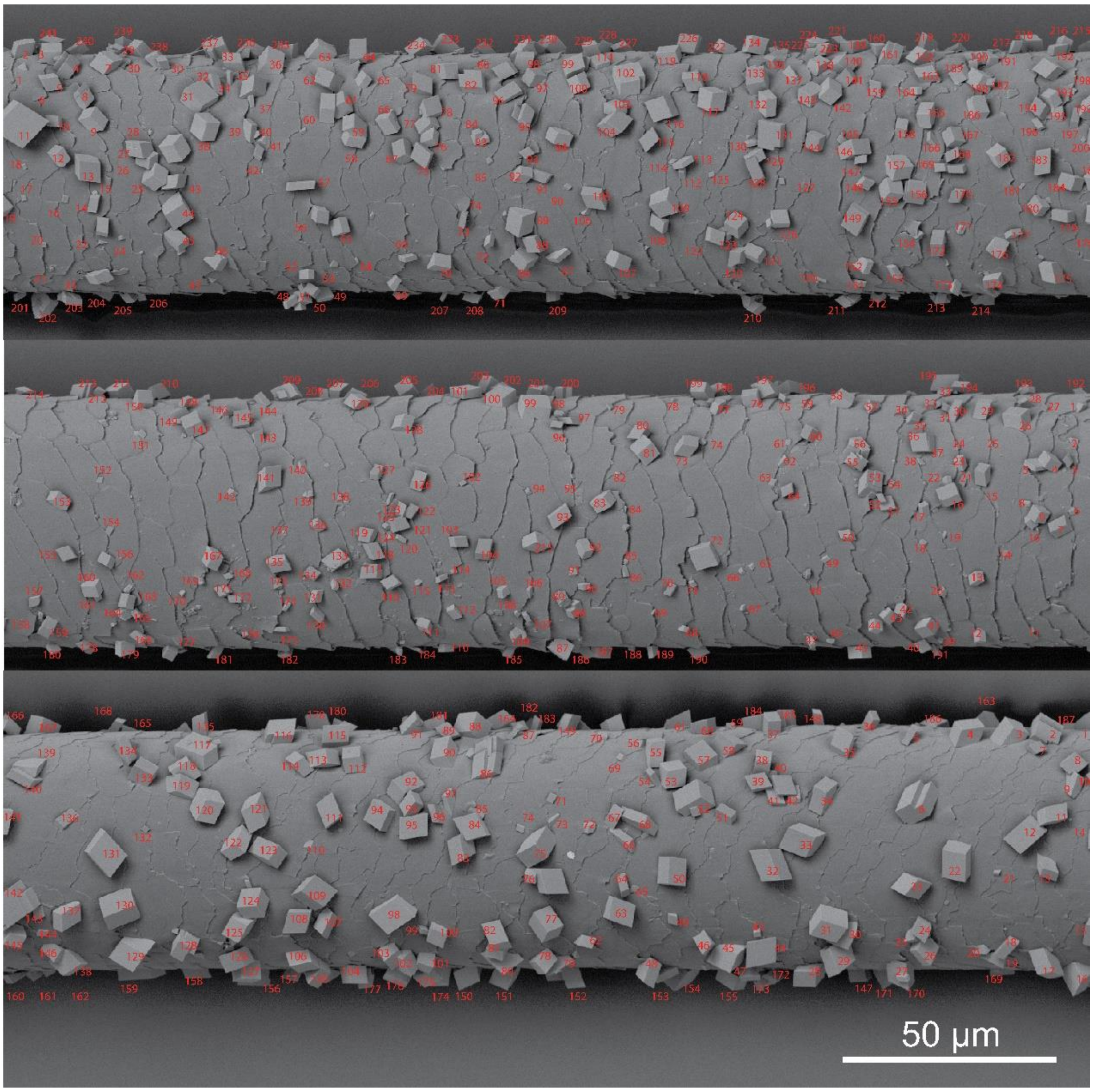

Figure S4. Three replicates of crystal number density measurements for petroleum ether treated dog hair.

1.3.4 Dog hair – ethanol treated

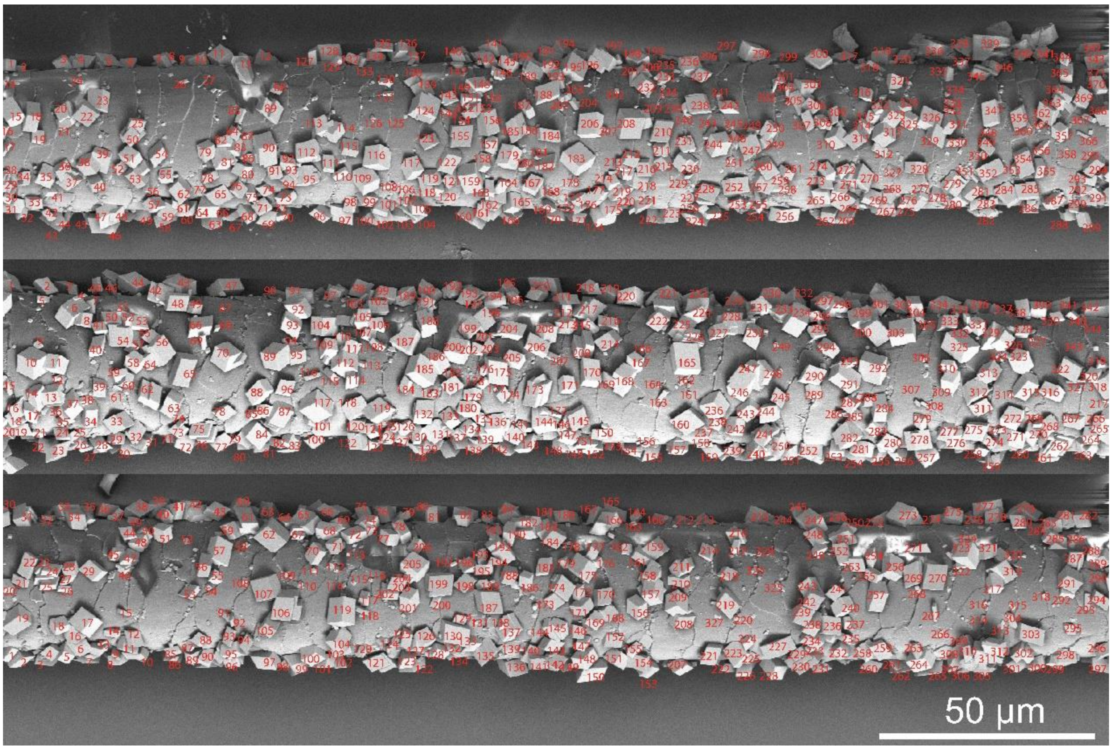

Figure S5. Three replicates of crystal number density measurements for ethanol treated dog hair.

1.3.5 Dog hair – hydrogen peroxide treated

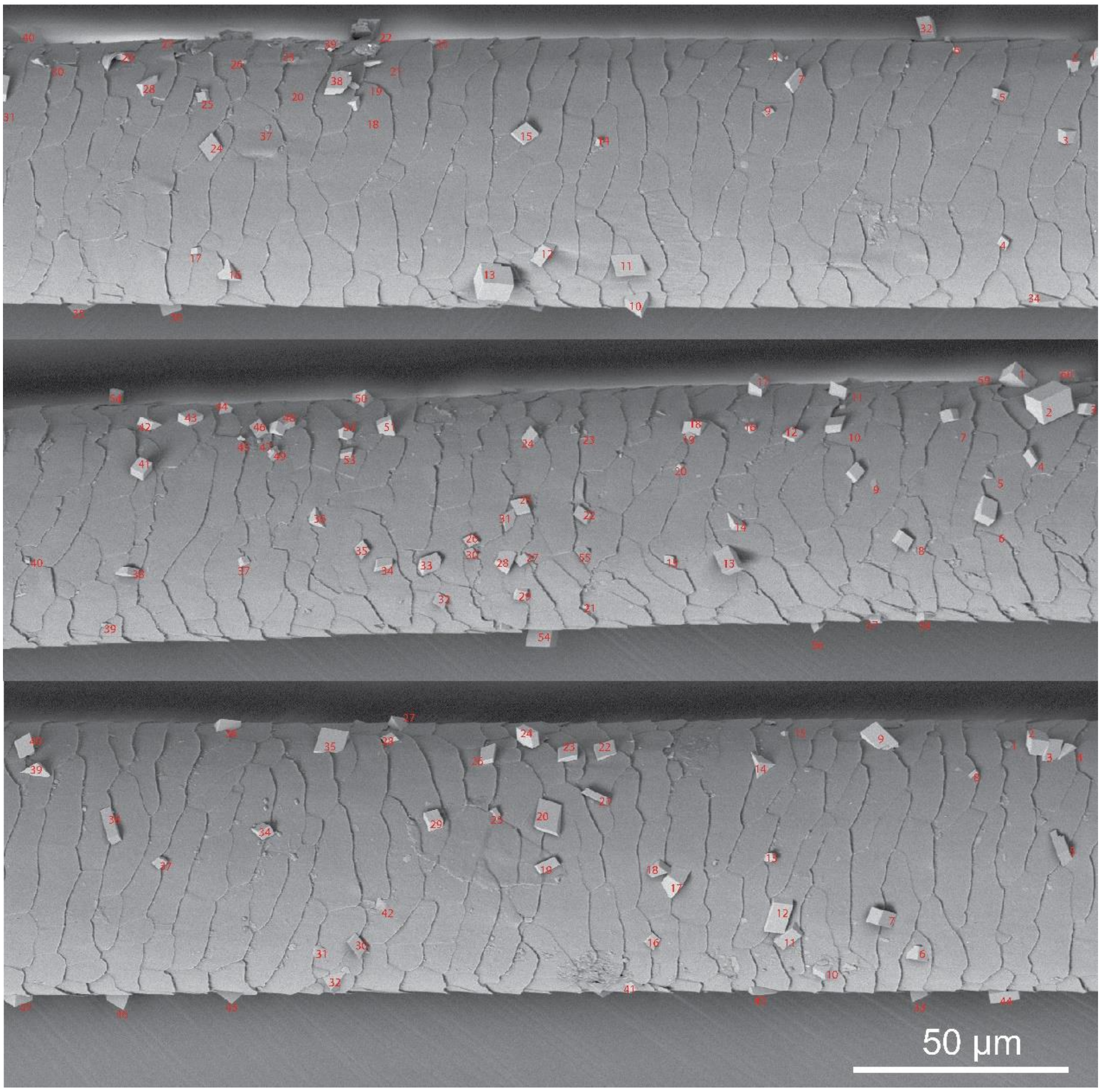

Figure S6. Three replicates of crystal number density measurements for H_2O_2 treated dog hair.

1.3.6 Human hair – water washed

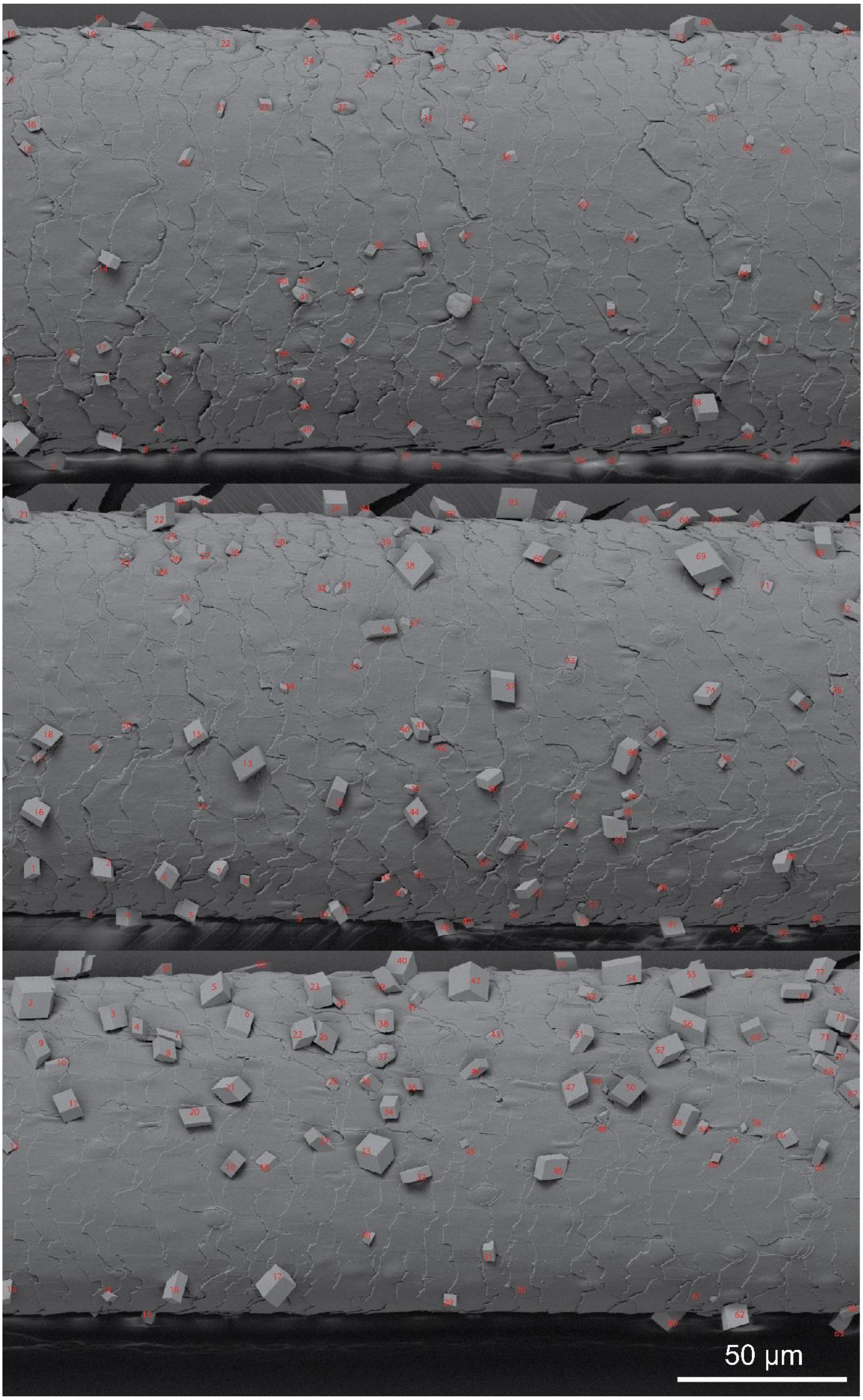

Figure S7. Three replicates of crystal number density measurements for water washed human hair.

1.3.7 Human hair – petroleum ether treated

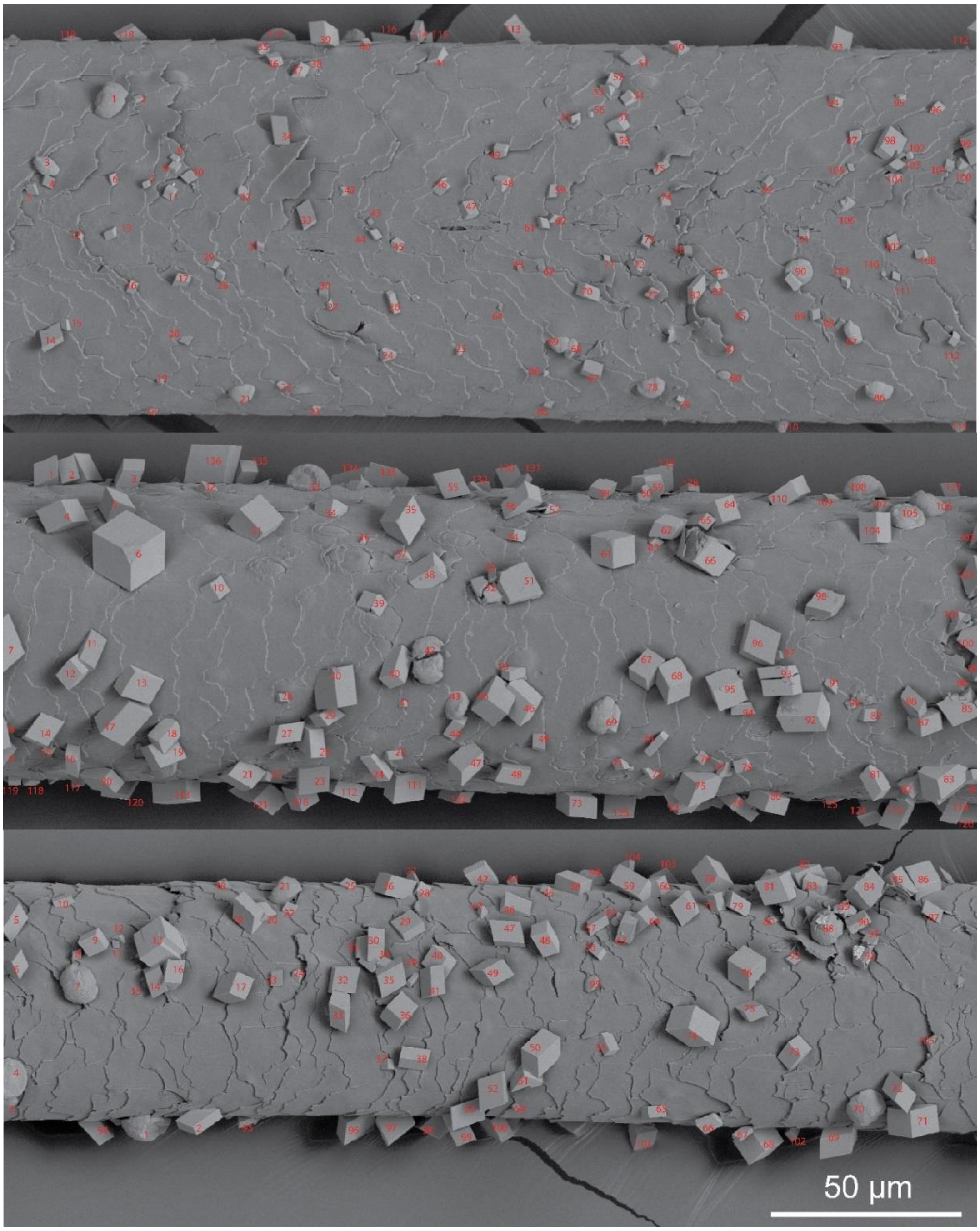

Figure S8. Three replicates of crystal number density measurements for petroleum ether treated human hair.

1.3.8 Human hair – ethanol treated

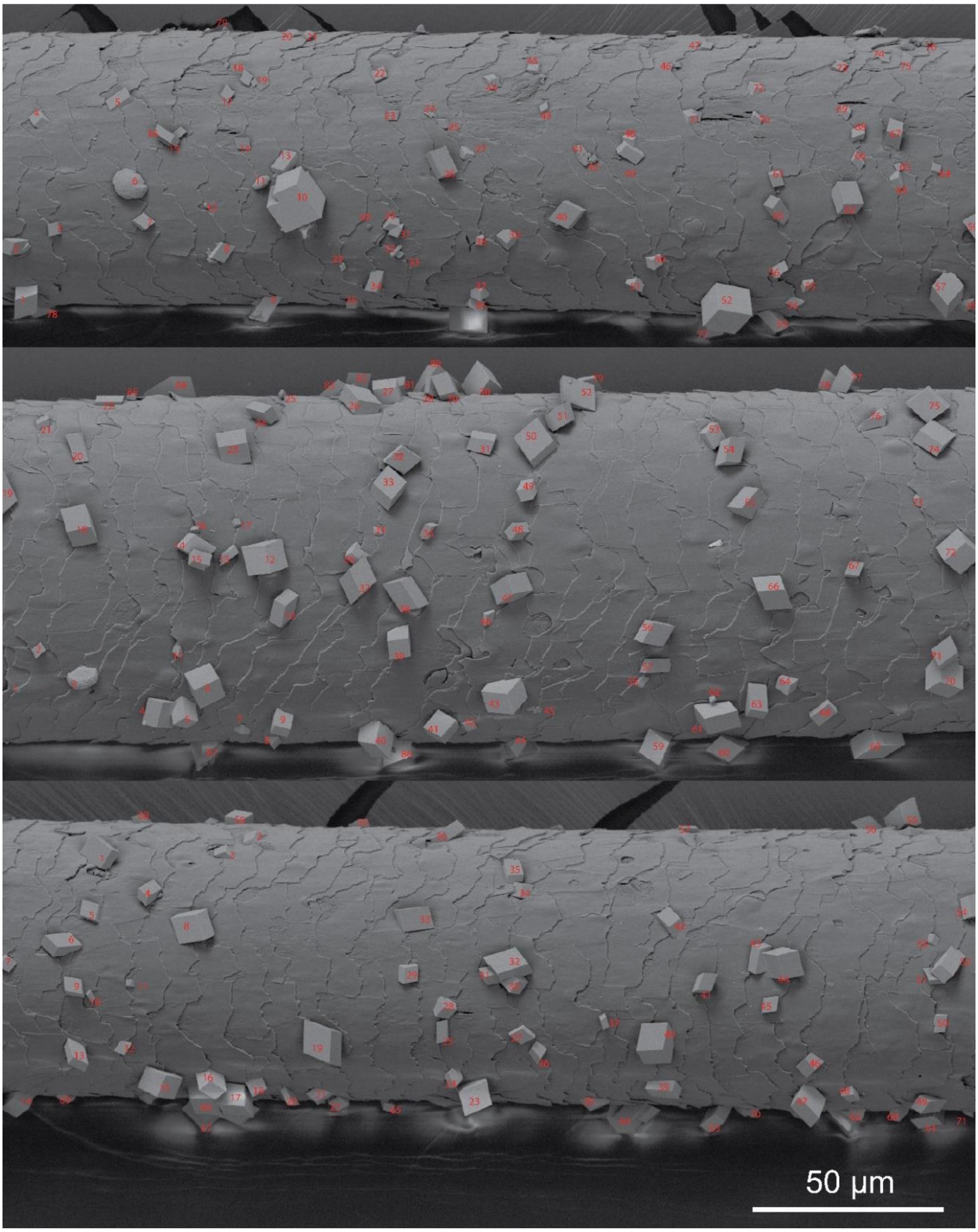

Figure S9. Three replicates of crystal number density measurements for ethanol treated human hair.

1.3.9 Human hair – hydrogen peroxide treated

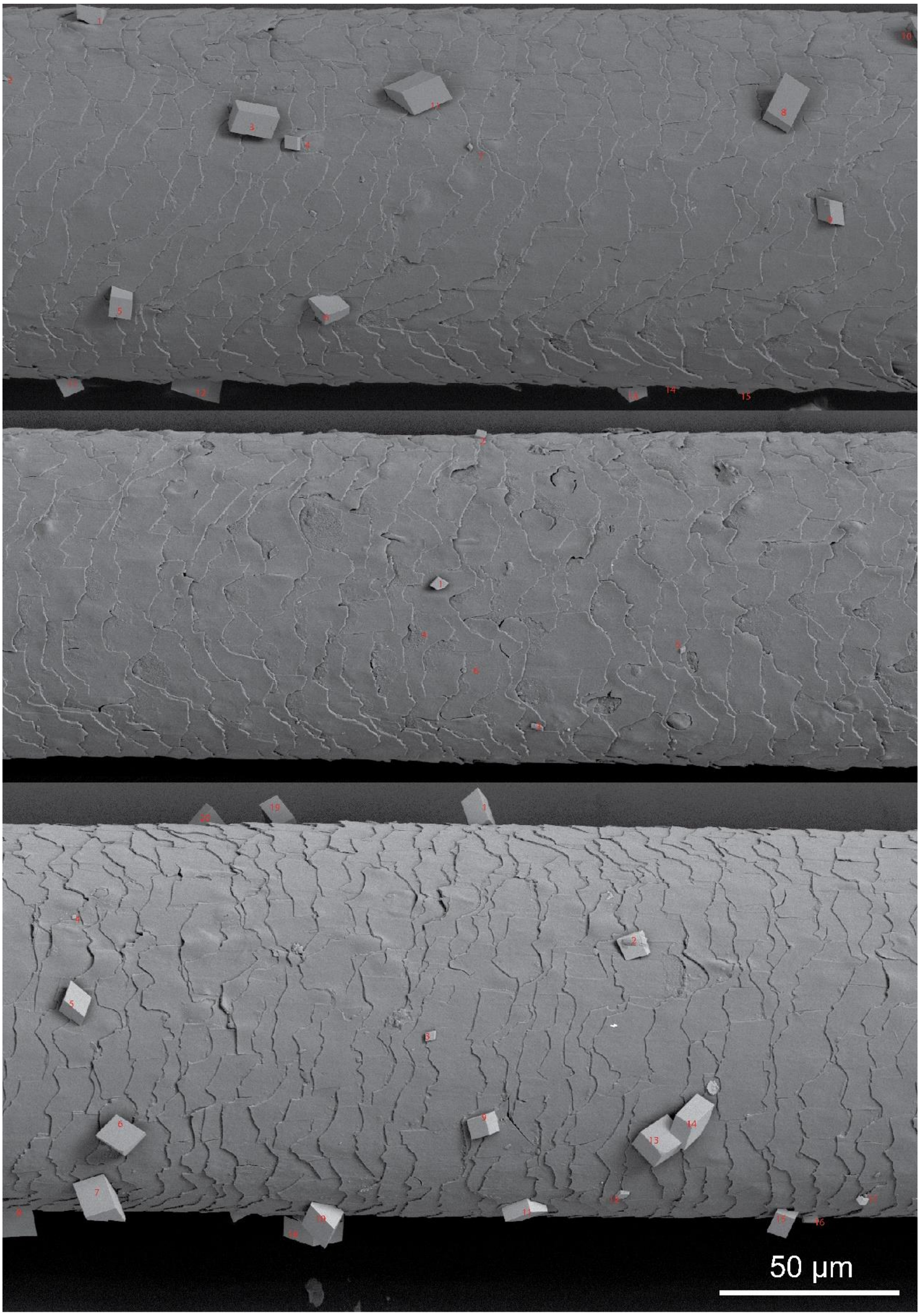

Figure S10. Three replicates of crystal number density measurements for hydrogen peroxide treated human hair.

2. KNO_3 droplet nucleation experiments

2.1. Experimental

Fabrication and treatment of nanopipettes Borosilicate glass capillaries (WPI GBF100-50-7.5) were loaded into a Sutter Model P-2000 micropipette puller. The programme was set using the following parameters to produce nanopipettes with tip diameters of 420 nm: HEAT=350, FIL=3, VEL=40, DEL=220, PULL = 0 then HEAT=350, FIL=3, VEL=40, DEL=180, PULL=80.

Fabrication of pulled glass nanorods Borosilicate glass rods (WPI GR100-4) were loaded into a Sutter Model P-2000 micropipette puller. The programme was set using the following parameters: HEAT=550, FIL=4, VEL=38, DEL=175, PULL=30, then HEAT=500, FIL=4, VEL=36, DEL=125, PULL=70. The pulled glass rods were then pushed gently through a lint-free wipe to produce tips with end diameters of 20-50 µm. Rods were washed with isopropyl alcohol, air dried and then plasma treated using a Diener Atto Plasma Cleaner set to 200W for 2 minutes. Glass tips were used within 1 hour of plasma treatment. Nanopipettes were dipped in Aquapel (PGW Auto Glass) to increase their hydrophobicity.

Experimental setup for KNO_3 droplet nucleation experiments A hydrophobic glass substrate was placed on a Zeiss Axio Observer 7 Material inverted light microscope, and approximately 2 mL of silicone oil was placed at the centre. A nanopipette was loaded with 2.5M KNO_3 (99%, Sigma-Aldrich) solution using Eppendorf Microloader tips and a micropipette, and this was connected to an Eppendorf InjectMan system. The loaded nanopipette was lowered through the oil phase into contact with the hydrophobic glass substrate. The supply pressure and injection time of the FemtoJet unit was adjusted to produce droplets of 100-130 µm in diameter. 3x3 arrays of droplets were deposited on the substrate, and over the course of 4 hours, counter-diffusion of water from the droplet into the oil phase increased the concentration of the droplets.

The hair and glass rod nucleants were mounted on a custom holder for a Sutter MPC-385 micromanipulator and submerged in water to improve their wettability. After the droplets had been left to concentrate between 2 and 5 hours, a nucleant was introduced to the droplet. Each nucleant contacted the droplet for 10 seconds before it was retracted, and each nucleant was only used to nucleate a maximum of 9 droplets with a water washing step between each droplet to avoid seed crystal retention. Video acquisition allowed tracking of droplet volumes from their initial maximum and substrate contact radii until the frame before they were contacted by a nucleant. A macro written for ImageJ enabled the outer radii to be extracted from the raw files and convert the values into a concentration (Section S2.2 and S2.3). The temperature of the system was also recorded using a three-wire PicoTech PT-104 temperature logger at the time of nucleant contact.

2.2. Calculation of droplet supersaturation ratios

All droplets exhibited a contact angle on the hydrophobic glass substrate of >90°, meaning that both an outer and inner/contact radius could be observed by optical microscopy and extracted automatically using image analysis (Figure S2 and S3). Droplets were deposited in a 3x3 array such that their outer radii were 65 ± 5 µm. The volume of a spherical cap could be calculated from the inner and outer radii (Figure S1) of the droplets by:

$$V_{cap} = \frac{\pi}{3} r_{outer}^3 (2 + \cos\theta)(1 - \cos\theta)^2 \qquad \textit{Eq S2}$$

where

$$\theta = \left(\sin^{-1}\left(\frac{r_{inner}}{r_{outer}}\right)\right). \qquad \textit{Eq S3}$$

Therefore, the droplet volume could be calculated from Equation S4:

$$V_{droplet} = V_{sphere} - V_{cap} = \frac{4\pi r_{outer}^3}{3} - \frac{\pi r_{outer}^3}{3}\left(2 + \cos\left(\sin^{-1}\left(\frac{r_{inner}}{r_{outer}}\right)\right)\right)\left(1 - \cos\left(\sin^{-1}\left(\frac{r_{inner}}{r_{outer}}\right)\right)\right)^2.$$

Eq S4

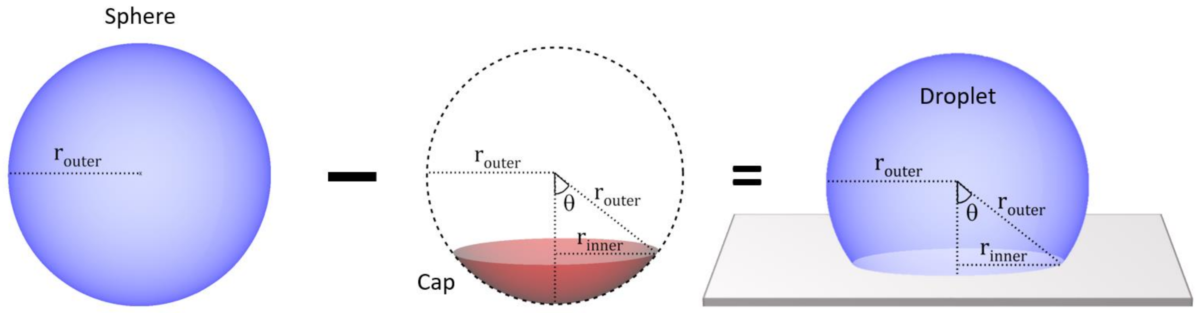

Figure S11. Geometric representation of the droplet showing how its volume could be calculated from the inner and outer radii observed by optical microscopy.

By taking an image of the droplets just after deposition when the concentration was 2.5 M and an image at time *t*, the concentrations could be calculated from the volumes at these stages from **Equation S5**:

$$C_t = C_0\left(\frac{V_0}{V_t}\right). \qquad \textit{Eq S5}$$

Simultaneous monitoring of temperature, which varied slightly, enabled the use of the following equation[1] to calculate and plot C/C_{sat} from initial concentration *C* and temperature *T*:

$$\frac{C}{C_{sat}} = \frac{101.1C}{132.17 + 2.52T(\ln T)} \qquad \textit{Eq S6}$$

2.3. Image analysis method for measuring inner and outer radii of shrinking droplets

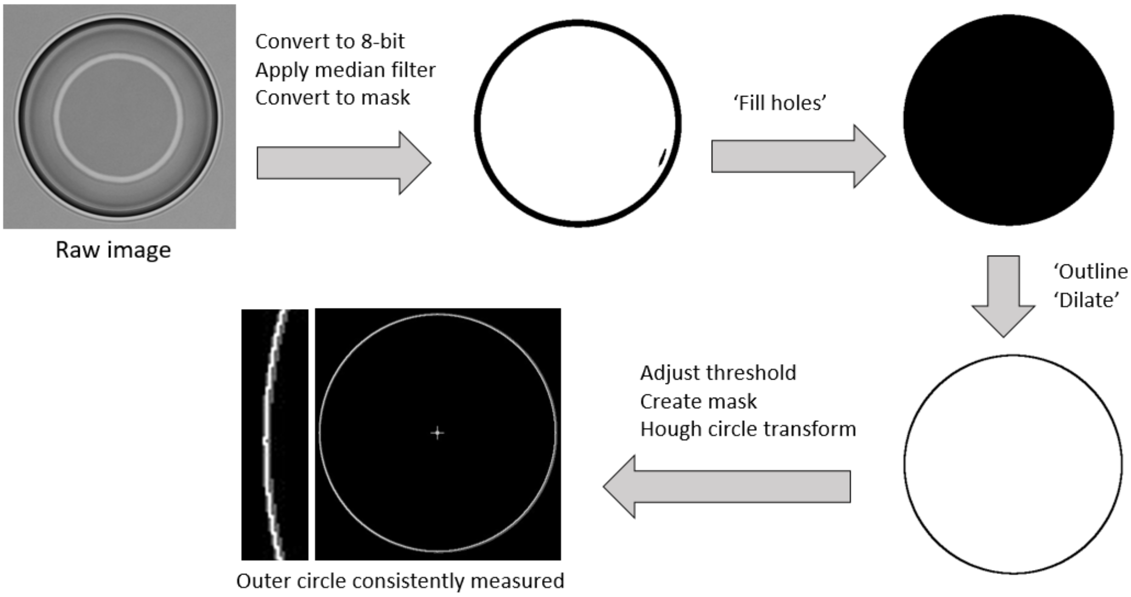

Figure S12. Methodology for image analysis using ImageJ to measure droplet outer diameters.

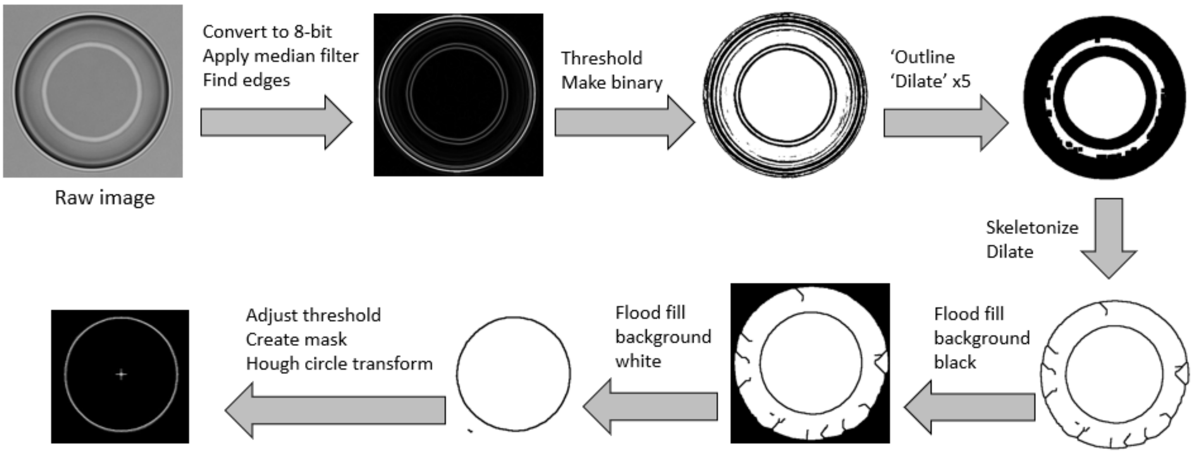

Figure S13. Methodology for image analysis using ImageJ to measure droplet inner diameters.

2.4. KNO_3 nucleation data processing and curve fitting

2.4.1. Data plotting

The results from these experiments needed to be treated carefully to avoid creating artificial trends in the data, and common ways to plot similar data needed to be reconsidered due to the nature of introducing a heterogeneous nucleant[2]. The x values (C/C_{sat}) followed a roughly Gaussian distribution, but these values were essentially experimental input variables rather than output variables, since the droplets were manually touched with a nucleant. This means that the gradient of a cumulative fraction of nucleated droplets plotted against C/C_{sat} would be highly sensitive to skewing based on uneven sampling of concentrations. This problem is illustrated in Figure S14a, where fictional nucleation data from a nucleant is collected and plotted twice, the only differences being the sampling width of the two experiments.

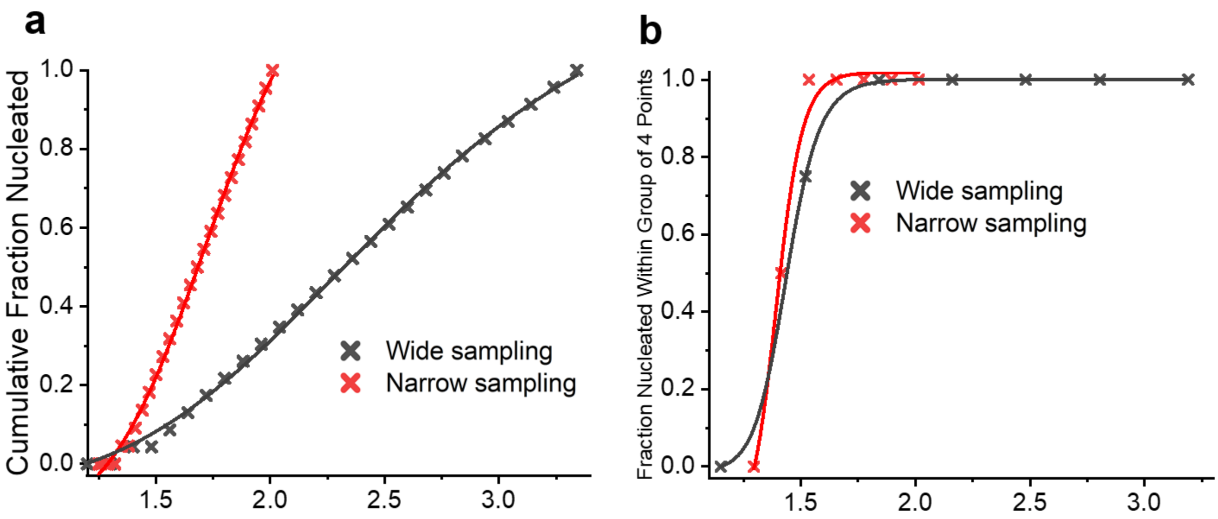

Figure S14. (a) Plot of cumulative fraction nucleated against C/C_{sat}, showing potential data skewing by uneven data sampling even when nucleant behaviour is near identical. (b) Demonstration of how data grouping was used to minimise the effect of data skewing by uneven data sampling.

To solve this problem, data points were instead grouped into sets of 10, in which C/C_{sat} was averaged (with standard deviations). Within each group, the fraction of crystallised droplets was calculated and plotted against the average C/C_{sat}. As shown in

Figure **S14**b, the data processing was able to greatly minimise any issues caused by uneven sampling widths.

2.5. Curve fitting

The y values of the graphs can be thought to be roughly proportional to the rate of nucleation. Ideally, heterogeneous nucleation rates should be a summation of the kinetics of nucleation at each possible surface site, shown by Equation S7.

$$Rate = \sum_{i=1}^{n} A_{\mathrm{i}} S e^{\frac{B_{\mathrm{i}}}{ln^2 S}} \qquad \textit{Eq S7}$$

Curve fitting was performed by iterating plot parameters to minimise chi squared, which means that using an equation that approximates Equation S7 would over fit the data. Instead, we fitted the data to a binomial nucleation rate equation, which allowed for the possibility that two different 'types' of surface site could dominate the nucleation processes at different supersaturation S_r ranges. C/C_{sat} is related to supersaturation S, but does not include the ion activity coefficients, which are challenging to calculate or measure at high ionic strengths and supersaturated conditions.

$$Rate = A_1 S e^{\frac{B_1}{ln^2 S}} + A_2 S e^{\frac{B_2}{ln^2 S}} \qquad \textit{Eq S8}$$

Here, A_1 and A_2 not only encompass the kinetic prefactors for nucleation at particular nucleation sites, but also include a number between 0 and 1 which gives the probability of such a site being accessible to the droplet. Extracting values for the distribution of active sites is not possible, because we cannot directly calculate the kinetic prefactor, but the curves were only intended to guide the eye. B_1 and B_2 are exponential terms related to energy barrier height, and define how steeply the gradient of the fitting curve will rise, but again, these quantities cannot be extracted from the values. Every data point group where 100% of droplets nucleated was masked and not included in the curve fitting, to make the curve resemble nucleation rate which would not be sigmoidal in shape. To replace these data points, a star symbol was used to indicate the highest value of C/C_{sat} at which a droplet did not nucleate. These stars were not included in the curve fitting.

3. KNO_3 Polymorphism

3.1. Experimental

Levitated droplet synchrotron wide-angle X-ray scattering (WAXS) measurements of KNO_3 Crystallization. *In situ* WAXS measurements were collected on BM26 at the European Synchrotron Radiation Facility (ESRF) using a monochromatic x-ray beam at 12.93keV (0.959Å). 2D scattering intensities were collected using a Dectris Pilatus 300 K-W. α-Al_2O_3 was used to calibrate the q-range between 1.0 $Å^{-1}$ and 2.5 $Å^{-1}$. A 2 μL KNO_3 droplet (0.5 M) was deposited into an acoustic levitator (Tec5, 100 kHz, Germany) using a hydrophobic needle. A dog hair was grafted to PTFE capillary tubing, and allowed to contact the levitated droplet. The droplet evaporated under ambient conditions whilst time-resolved WAXS was simultaneously collected at a rate of 100 ms/frame. The 2D WAXS patterns were background-corrected using the first frame at the beamline using PyFAI software and averaged every 50 frames after preliminary analysis.

4. X-ray photoelectron spectroscopy (XPS) and atomic force microscopy (AFM) characterization of the hair samples

4.1. Experimental

AFM measurements. AFM measurements were carried out in tapping mode under ambient conditions using a Bruker Innova instrument (Bruker, USA). Bruker RFESP-75 probes were used (k = 3 N/m, f_0 = 75 kHz).

Ultra-high vacuum XPS. XPS spectra were collected on a Kratos Axis Ultra DLD spectrometer at the University of Warwick (base pressure less than 1 × 10-10 mbar,) equipped with a monochromated Al Kα X-ray source (1486.69 eV, 120W, and analysis area of 0.300 mm × 0.700 mm). Hairs were mounted to the stage in tight bundles using adhesive carbon tape. Survey spectra were collected using an analyser pass energy of 160 eV (1 eV increments and 500 ms dwell time). High resolution spectra were

collected using an analyser pass energy of 20 eV (0.1 eV increments). All samples were charge compensated by a low-energy electron gun. The spectrometer work function and binding energy scale were calibrated using the Fermi edge and 3d5/2 peak recorded from a polycrystalline Ag sample.

Environmental XPS. Environmental XPS spectra were collected with a SPECS EnviroESCA NAP-XPS equipped with a monochromatic Al Kα X-ray source (1486.71 eV) operating at 42 W, which is separated from the analysis chamber by a SiN window. The samples were illuminated with a ~0.3 mm-diameter beam footprint. The hair samples were mounted by twisting each of them into a single strand and affixing the ends to a large SEM stub with an adhesive putty. Spectra were collected at ambient temperature in 7 mbar of nitrogen with a hemispherical Phoibos NAP 150 analyzer operating in a small-area mode with a source−analyzer angle of 55° and a 1D delay-line detector. The gas purity was cross-checked using a mass spectrometer. Under NAP conditions in the EnviroESCA instrument, electrically insulating sample surfaces are charge-neutralized through ionization of gas phase atoms by energetic electrons emitted from the sample and, to a lesser extent, the X-ray beam itself. Survey spectra were collected in one scan with a step size of 1 eV, a pass energy of 100 eV, and a dwell time of 0.1 s. High-resolution C 1s, N 1s, O 1s and S 2p core-level spectra were collected with a step size of 0.1 eV, a dwell time of 100 ms per data point, and a pass energy of 50 eV. Spectra were calibrated for intensity using an instrument transmission function.

XPS fitting. XPS spectra were referenced to the peak maximum of the aliphatic C 1s component at a binding energy of 285 eV[3]. S 2p (S 2p1/2 and S 2p3/2) peaks were fitted to the Gaussian-Lorentzian (30/70) line shapes and Shirley background. Quantification from survey spectra were carried out using O 1s, N 1s, C 1s, S 2s, Si 2p (contaminant) core-level peaks and were calibrated for intensity using an instrument transmission function. Data analysis was performed using the CasaXPS package[4].

4.2. All survey scans – ultra-high vacuum XPS

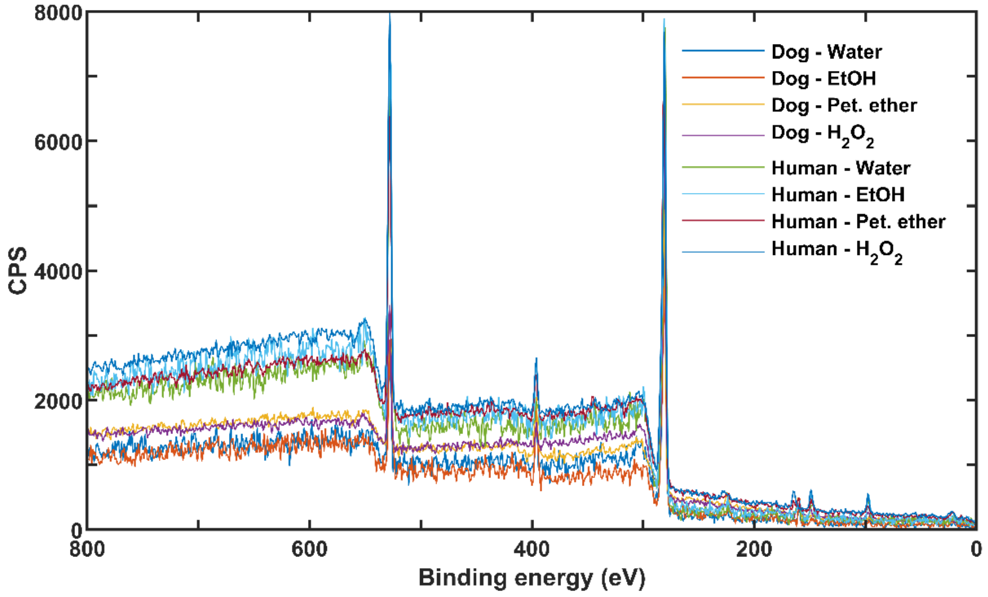

Figure S15. Survey scan for all samples using ultra-high vacuum XPS.

4.3. All samples – ultra-high vacuum XPS sulfur scans

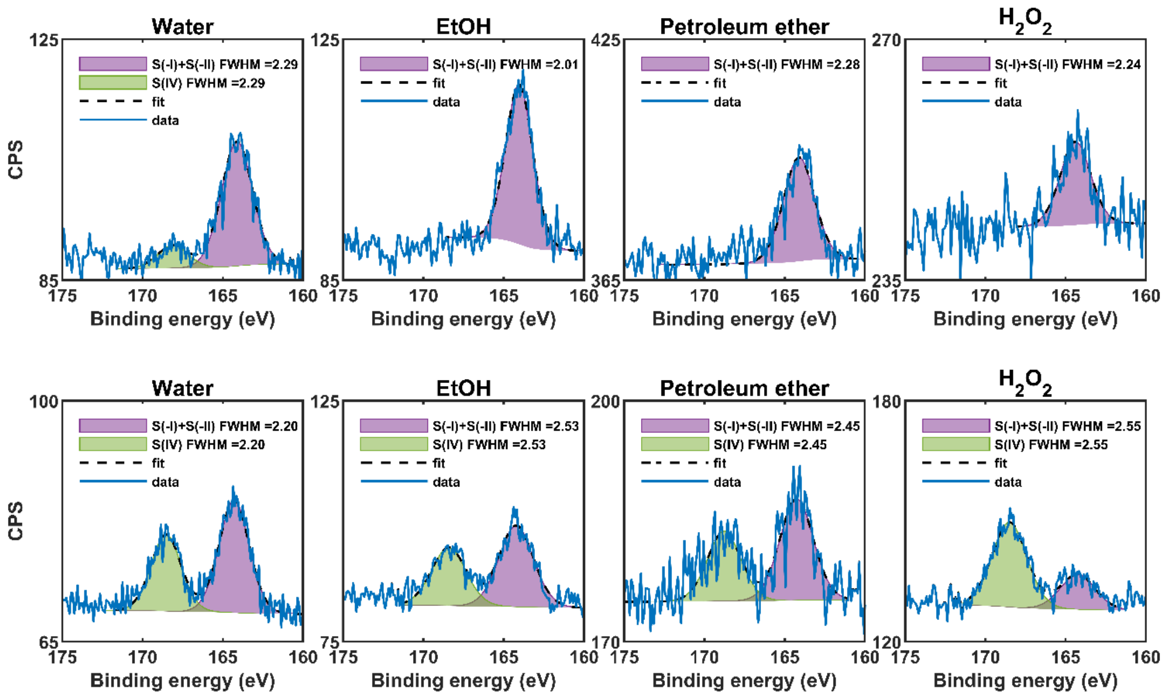

Figure S16. High-resolution S 2p scans for all samples (dog top and human bottom) for ultra-high vacuum XPS.

4.4. Petroleum ether treatment prior to hydrogen peroxide treatment – environmental XPS sulfur scans

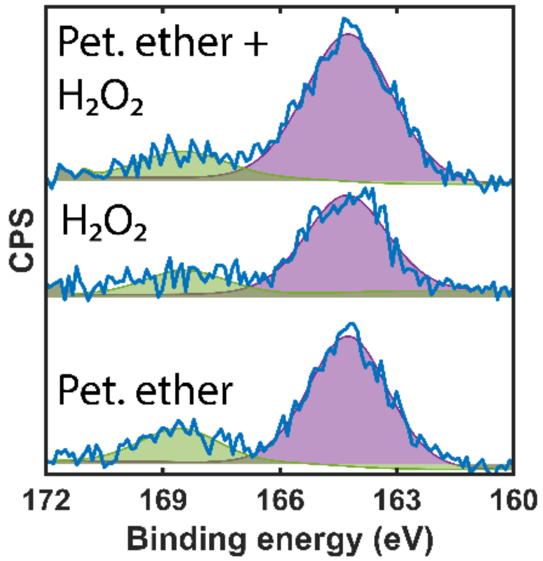

Figure S17. High-resolution S 2p scans for Petroleum ether, H_2O_2, and petroleum ether prior to H_2O_2 treatment for environmental XPS.

5. SICM surface charge mapping

5.1. Method

SICM works by measuring the ion current flowing between a quasi-reference counter electrode (QRCE) inside a nanopipette and a QRCE placed in the solution bathing a sample (Figure S18a) [5]. In hopping mode, the nanopipette is translated towards the sample with a small bias applied (here +50 mV vs bath QRCE) until the ion current is observed to decrease, as access to the nanopipette pore is hindered by the sample. The displacement required to reach a certain decrease in current (here 3%) is recorded and the nanopipette is then retracted and moved to a new location and the process repeated to build up a map of the sample topography and current (Figure S18b and c).

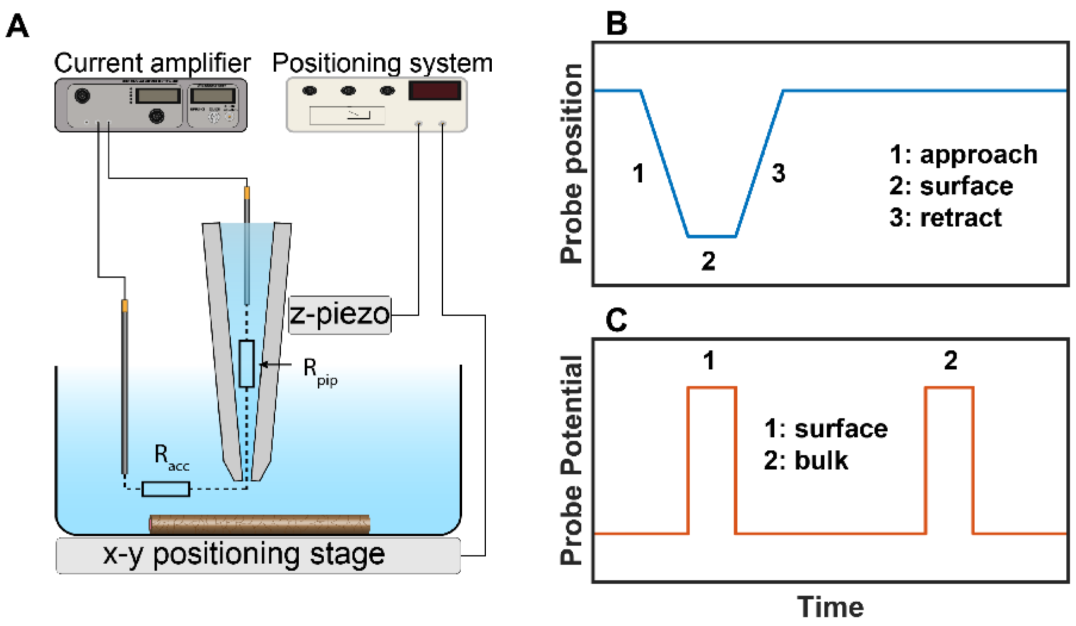

Figure S18. SICM methodology. a) Experimental setup and resistive contributions to the measured ionic current. b) Probe translation at one pixel. c) Tip potential versus time at one pixel.

5.2. Experimental

SICM SICM measurements were carried out using an in-house built instrument running on a LabVIEW 2016 (National Instruments, USA) interface to implement the Warwick Electrochemical Scanned Probe Microscopy (WECSPM) software. The instrument was equipped with a 300 µm x-y-piezo: Nano-Bio300 (MadCityLabs, USA), 38 µm z-piezo: P-753.2 LISA (PI, Germany), a home-build current follower and brick wall filter, all mounted on an Axiovert 40 CFL microscope (Zeiss, Germany). The current sampling rate was 2.56 ms, and the steady-state current was found from the last data point in an *i-t* trace. The approach time was 2 µm sec^{-1} and the approach and pulse potentials were 50 mV and –400 mV, respectively. The electrolyte conditions were 45 mM KNO_3 and 5 mM KCl. Nanopipettes were fabricated from borosilicate glass capillaries (BF120-69-10, Sutter instruments, USA) using a P-2000 laser puller (Sutter instruments, USA) with the following protocol: HEAT=350, FIL=3, VEL=30, DEL=220, PULL=0, then HEAT=350, FIL=3, VEL=40, DEL=180, PULL=120.

5.3. SICM data

5.3.1 SICM data – dog hair – ethanol treated

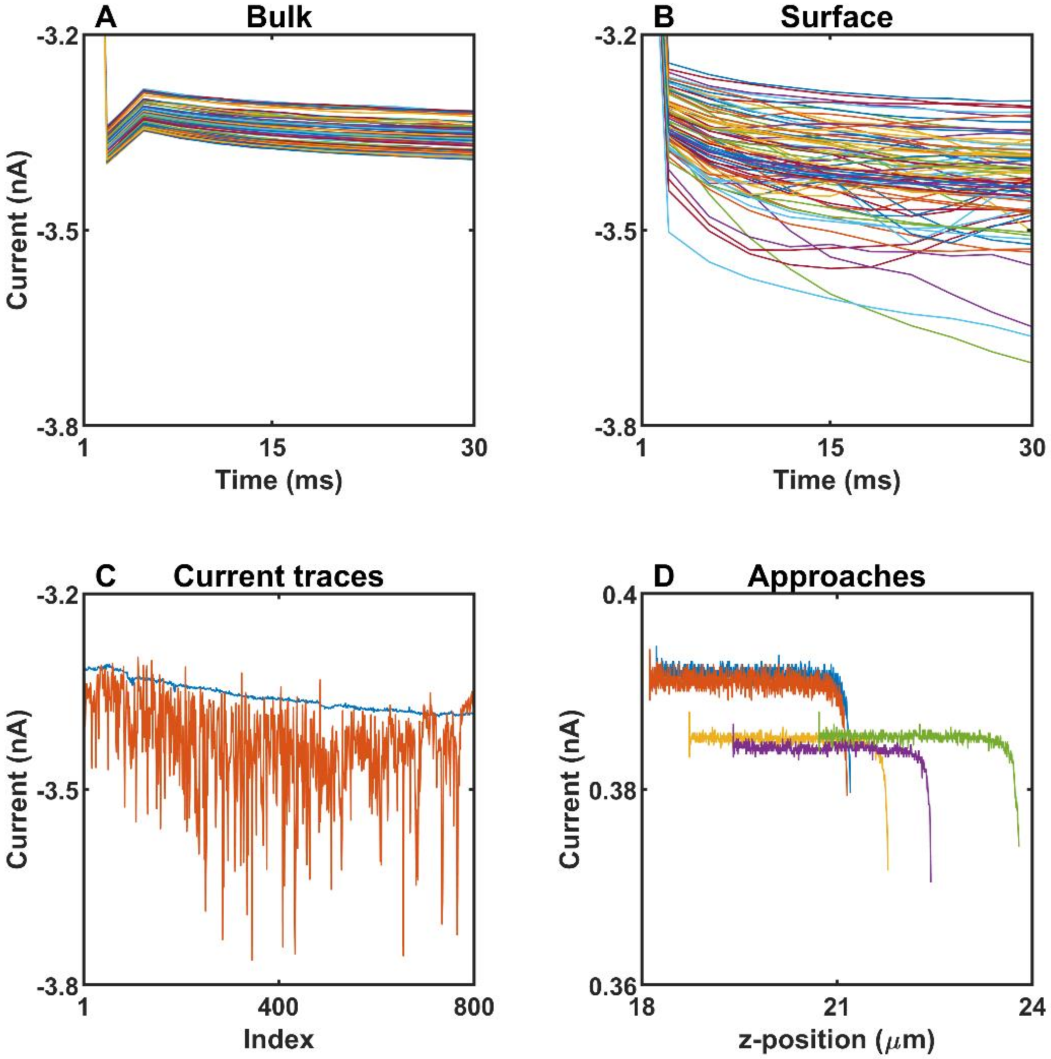

Figure S19. Dog, long scan. (A/B) Bulk and surface i-t traces, respectively. Current averages and sampled over 2.56 ms (first point reads as 0 due to current overload). (C) Bulk and surface current at 30 ms as a function of time (pixel number). (D) Approach curves.

5.3.2 SICM data – dog hair – hydrogen peroxide treated

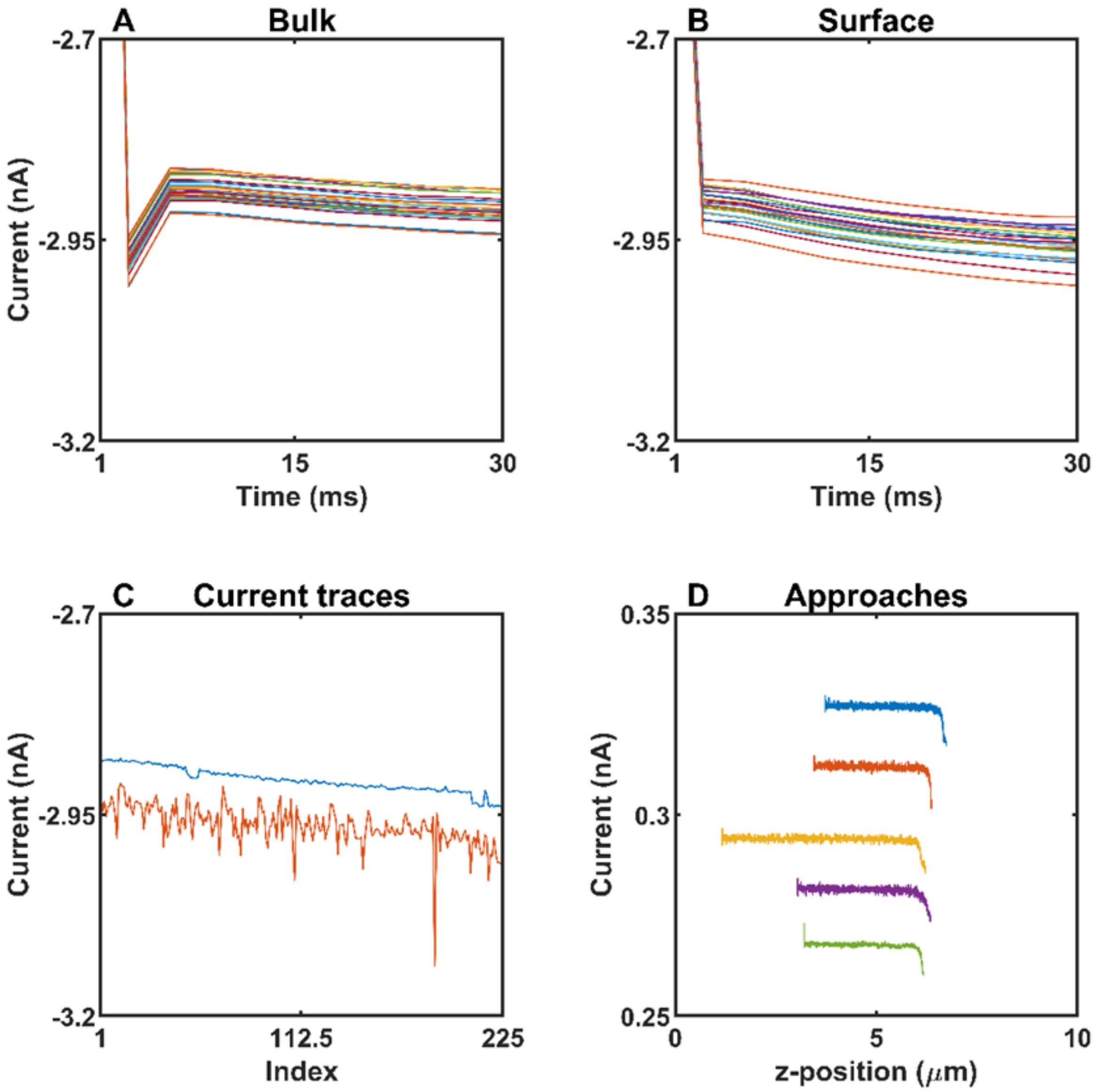

Figure S20. Dog (H_2O_2 treated), long scan. A/B) Bulk and surface i-t traces, respectively. Current averages and sampled over 2.56 ms (first point reads as 0 due to current overload). C) Bulk and surface current at 30 ms as a function of time (pixel number). D) Approach curves.

5.3.3 SICM data – human hair – ethanol treated

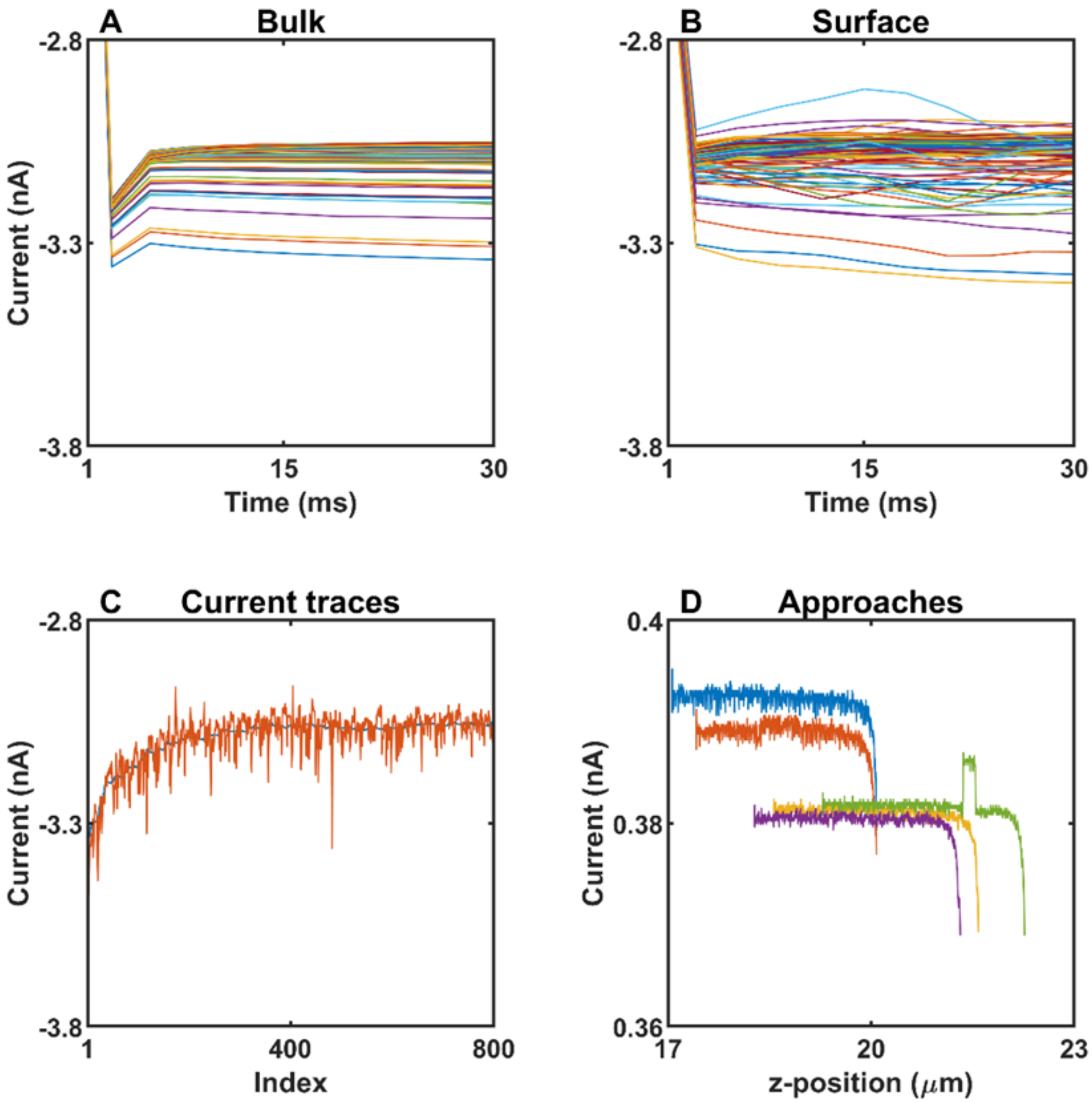

Figure S21. Human (EtOH treated), long scan. A/B) Bulk and surface i-t traces, respectively. Current averages and sampled over 2.56 ms (first point reads as 0 due to current overload). C) Bulk and surface current at 30 ms as a function of time (pixel number). D) Approach curves.

5.3.4 SICM data – dog hair – ethanol treated (3D map)

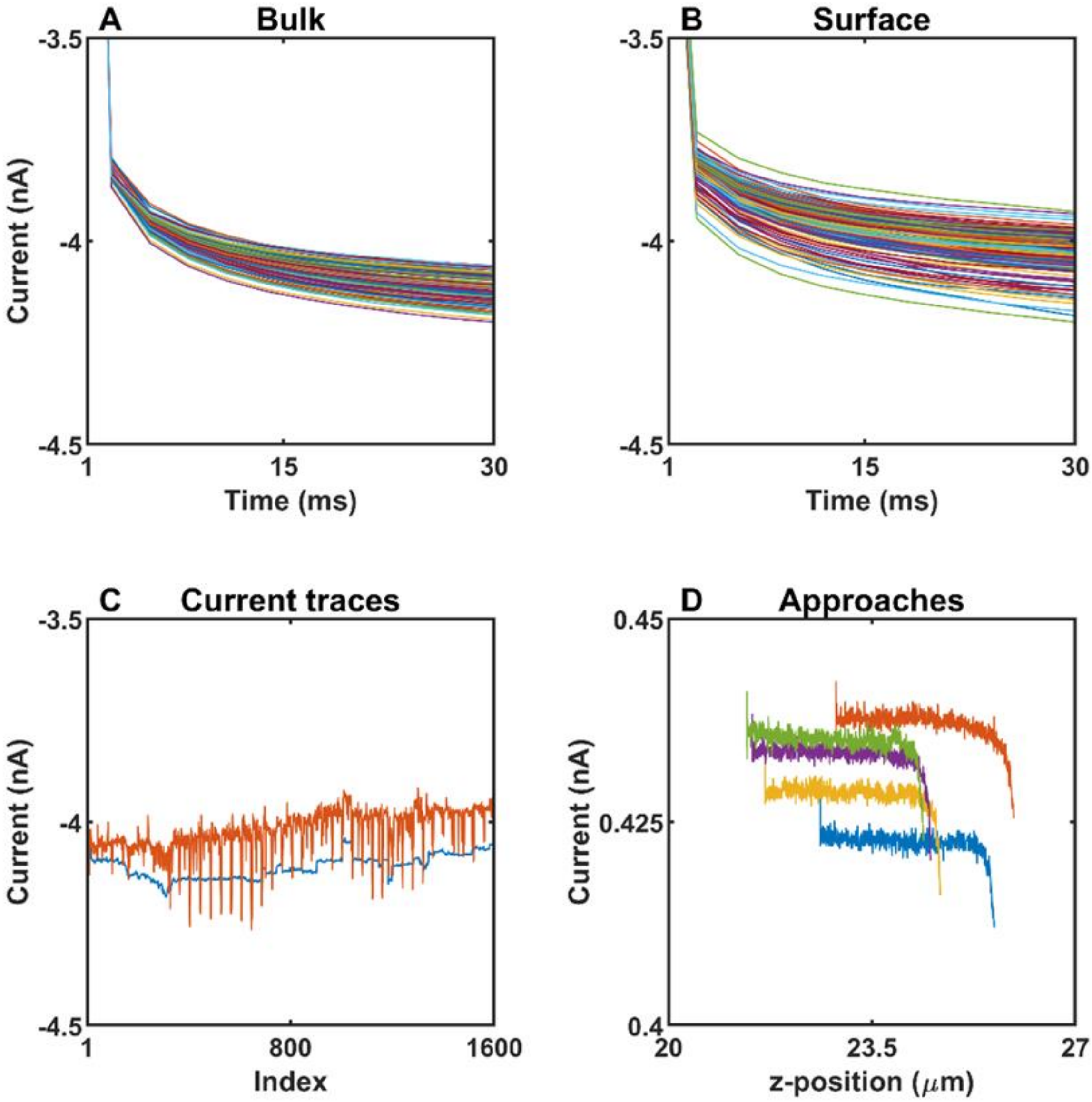

Figure S22. Dog 3D image. (A/B) Bulk and surface i-t traces, respectively. Current averages and sampled over 2.56 ms (first point reads as 0 due to current overload). (C) Bulk and surface current at 30 ms as a function of time (pixel number). (D) Approach curves.

6. Simulation framework for evaluating ionic current

6.1. Simulation method

By modelling the nanopipette near and far from the surface, it is possible to simulate the normalised current expected as a function of wall surface charge, substrate surface charge and distance from the surface, and therefore estimate these parameters for the experimental system. The methodology generally consists of:

1. Accurately measure the nanopipette tip geometry with (scanning) transmission electron microscopy (STEM)
2. Simulate current-voltage curves at different wall surface charges for accurate geometries to estimate the surface charge
3. With the best fit surface charge, simulate the normalised current-distance at the approach voltage used to determine the approach distance
4. At the approach distance, and with the fitted wall surface charge, simulate the normalised current at a range of sample surface charges

6.2. Simulation model

Ion current in SICM is simulated by numerical solution of the Nernst-Planck equation with convection, the Poisson and the Navier-Stokes equations based on the geometry found by STEM (Figure S23)[6–8].

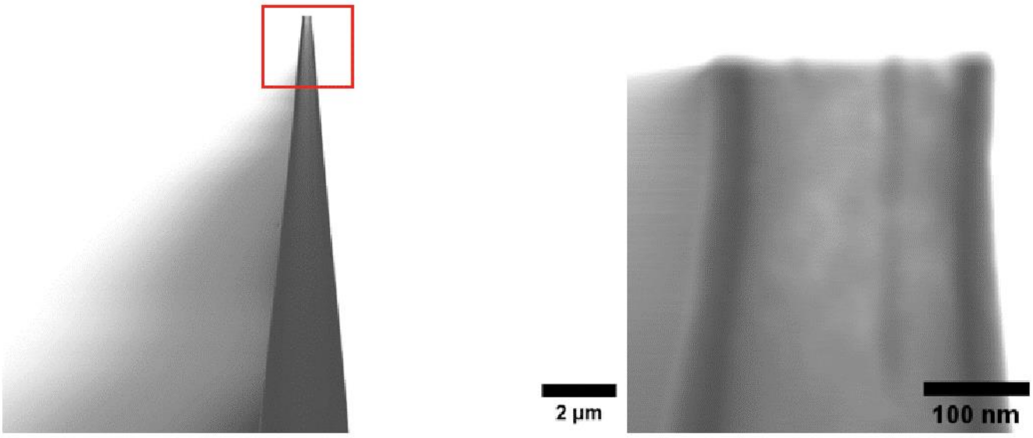

Figure S23. Scanning transmission electron microscope images of a typical nanopipette used in SICM surface charge mapping.

Boundary conditions used here are described in Table S2 and Figure S24. Ion diffusion coefficients were taken from the CRC Handbook[9]. The equations are solved using COMSOL Multiphysics (v6.0) and further details can be found in the model report included as Supporting Information 2.

Boundary	Poisson	Nernst-Planck	Navier-Stokes
B1	Pipette potential	50 mM K^+<br>45 mM NO_3^-<br>5 mM Cl^-	Zero pressure
B2	Pipette wall surface charge density (-28 mC m^{-2})	No flux	No slip
B3	No charge	No flux	No slip
B4	Ground (0 V)	50 mM K^+<br>45 mM NO_3^-<br>5 mM Cl^-	Zero pressure
B5	Substrate surface charge	No flux	No slip

Table S2. Description of boundary conditions used in simulations.

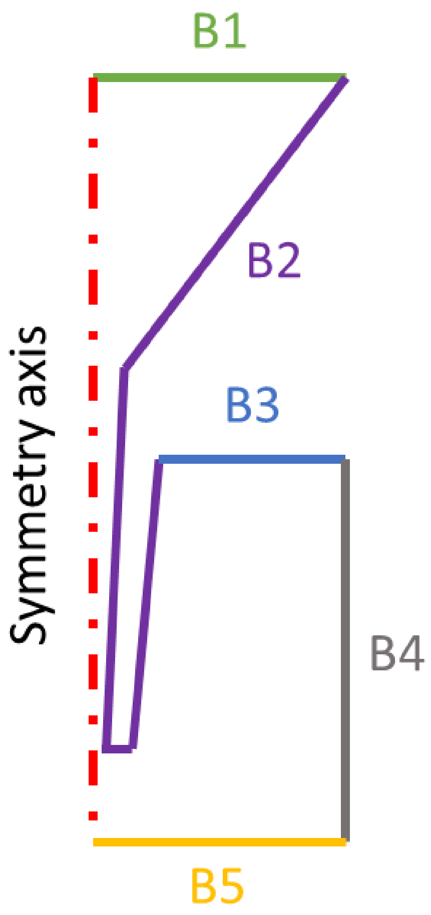

Figure S24. Schematic of the axis-symmetric geometry used in simulations. B1 and B4 represent the semi-infinite bulk of the pipette and the bath, respectively. B2 represents the nanopipette walls. B3 represents the solution surface. B5 represents the hair surface.

To determine the surface charge of the nanopipette, the current-voltage relationship was simulated for a surface charge parametric sweep as shown in **Figure S25**. The pipette surface charge was here estimated to be -28 mC m^{-2}.

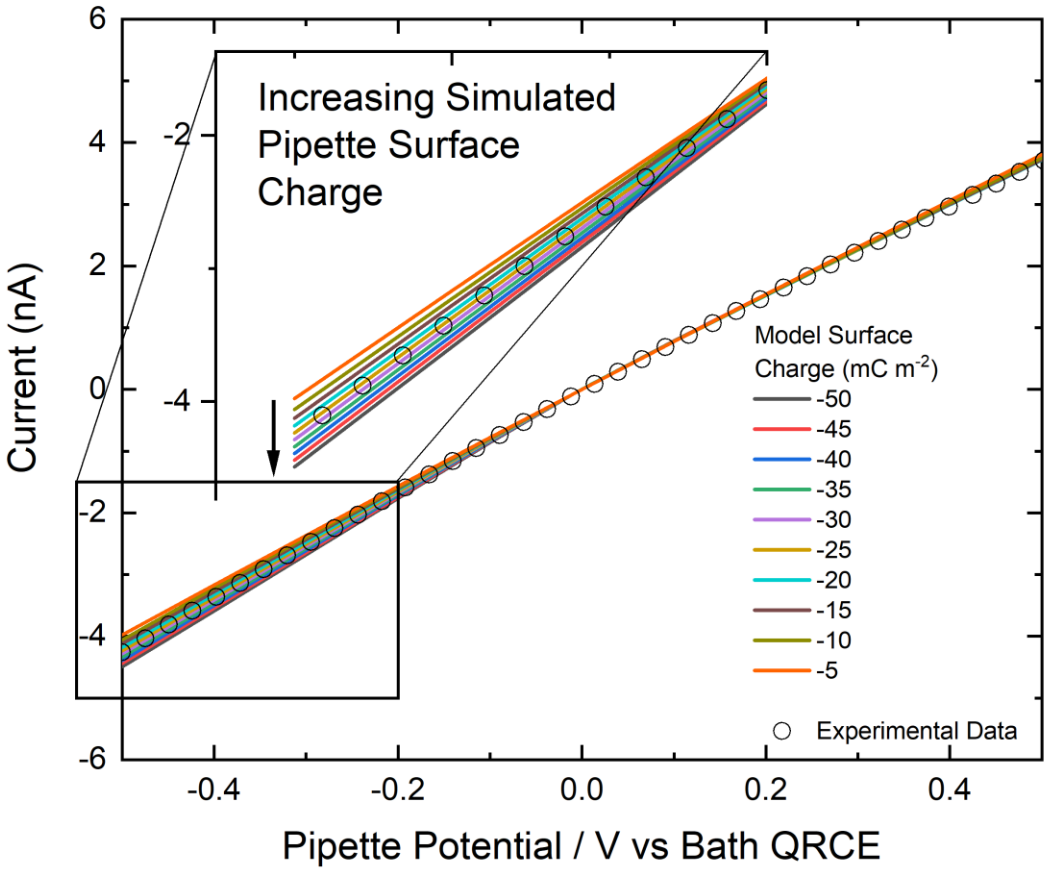

Figure S25. Comparison of simulated (lines) and experimental (points) steady state current-voltage curves. The simulations used the pipette geometry shown in Figures S23 and S24 and were carried out for pipette wall surface charges between -50 mC m^{-2} and -5 mC m^{-2}.

With the geometry and surface charge of the pipettes used had been determined, the current-distance relationship can be determined from a new simulation to gauge the distance corresponding to a 3% current drop, which was used experimentally. The tip-substrate distance was then determined to be 30 nm during potential pulses at the surface (Figure S26).

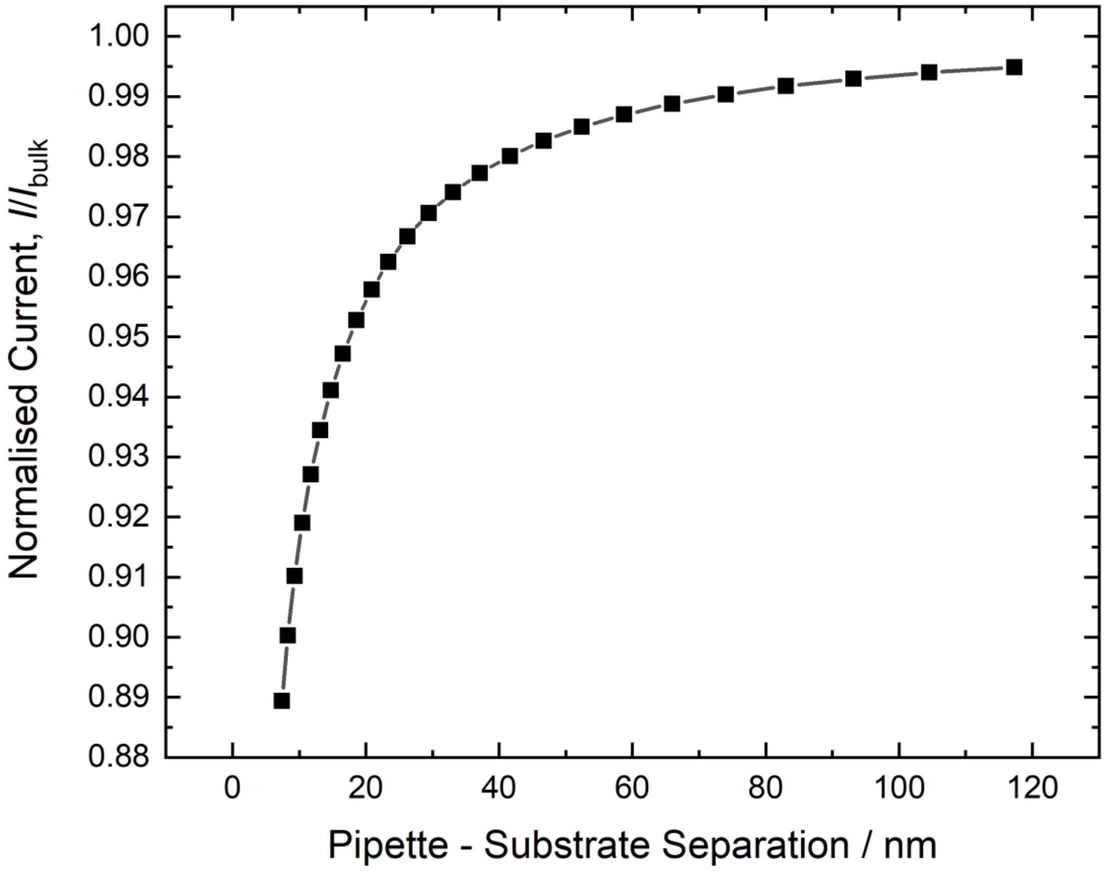

Figure S26. Simulated approach curve. The current is normalised to the bulk current, taken to be that at a pipette-surface separation of 1 µm. Simulations were carried out with a pipette potential of +0.05 V vs Bath QRCE and a pipette wall charge of -28 mC m^{-2}.

The normalized current as a function of substrate surface charge is then simulated to generate the calibration curve used to quantify surface charge from the experimental SICM data (**Figure S27**).

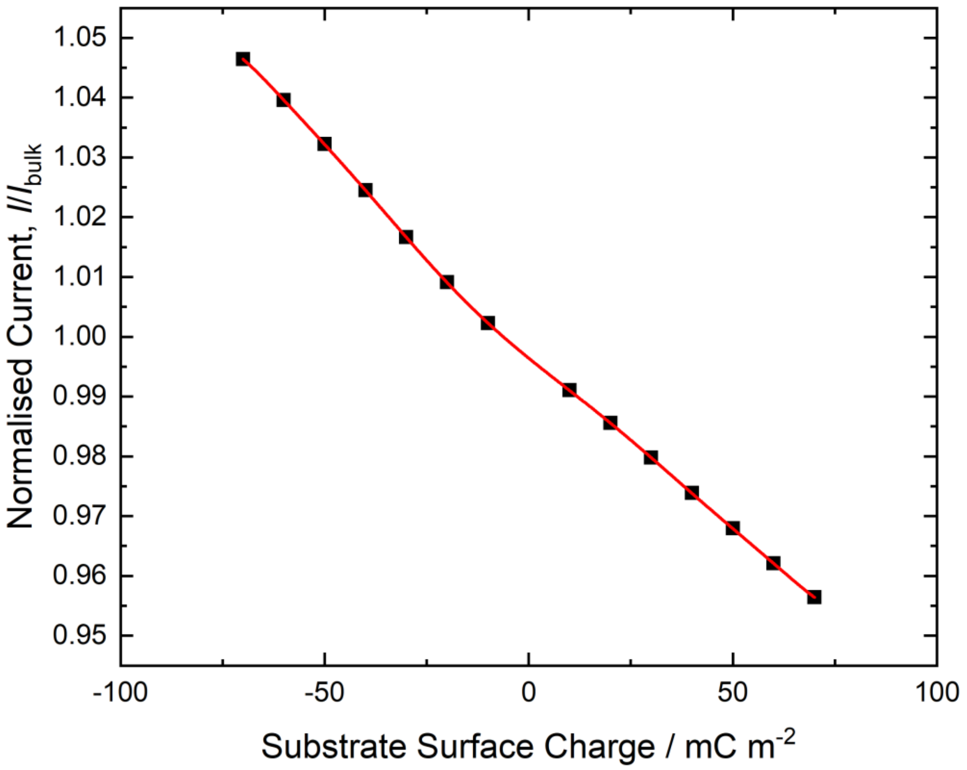

Figure S27. The normalised current as a function of substrate surface charge. The pipette potential was -0.4 V vs Bath QRCE. An optimised wall surface charge of -28 mC m^{-2} and a pipette-substrate approach distance of 30 nm was used. The bulk current was simulated at an approach distance of 1.48 µm. The red line shows a fit to a ninth-order polynomial which was used to convert the experimental data from normalised current to surface charge.

7. References

1 Linnikov, O. D.; Rodina, I. V.; Grigorov. I. G.; Polyakov, E. V. Kinetics and Mechanism of Spontaneous Crystallization of Potassium Nitrate from Its Supersaturated Aqueous Solutions *Cryst. Struct. Theory Appl.* **2013**, *02*, 16-27.

2 Cedeno, R.; Grossier, R.; Candoni, N.; Levernier, N.; Flood, A.; Veesler, S. CNT Effective Interfacial Energy and Pre-Exponential Kinetic Factor from Measured NaCl Crystal Nucleation Time Distributions in Contracting Microdroplets. **2023**, (Preprint) arXiv:2301.11088 submitted: Jan 2023

3 Biesinger, M. B. Accessing the robustness of adventitious carbon for charge referencing (correction) purposes in XPS analysis: Insights from a multi-user facility data review *Appl. Surf. Sci.*, **2022**, *597*, 153681.

4 Fairley, N.; Fernandez, V. ; Richard-Plouet, M.; Guillot-Deudon, C.; Walton, J.; Smith, E.; Flahaut, D.; Greiner, M.; Biesinger, M.; Tougaard, S.; Morgan D.; Baltrusaitis, J. Systematic and collaborative approach to problem solving using X-ray photoelectron spectroscopy *Applied Surface Science Advances*, **2021**, *5*, 100112.

5 Hansma, P. K.; Drake, B.; Marti, O.; Gould, S. A. C.; Prater, C. B.; The scanning ion-conductance microscope *Science,* **1989**, *243*, 641–643.

6 Perry, D.; Momotenko, D.; Lazenby, R. A.; Kang, M.; Unwin, P. R.; Characterization of nanopipettes *Anal. Chem.* **2016**, *88*, 5523–5530.

7 Sa, N.; Lan, W. J.; Shi, W.; Baker, L. A.; Rectification of ion current in nanopipettes by external substrates *ACS Nano,* **2013**, *7*, 11272–11282.

8 Maddar, F. M.; Perry, D.; Brooks, R.; Page, A.; Unwin, P. R. Nanoscale surface charge visualization of human hair *Anal. Chem.,* **2019**, *91*, 4632–4639.

9 David R. Lide, *CRC Handbook of Chemistry and Physics: Vol. 85*, CRC press, 2004.

SurfaceChargeSimulation report

Report date	Mar 2, 2023, 4:50:50 PM

Contents

1. Global Definitions 3
1.1. Parameters 3
1.2. Geometry Parts 6
1.3. Shared Properties 9
2. Model 1 10
2.1. Definitions 10
2.2. Geometry 18
2.3. Materials 20
2.4. Transport of Diluted Species 24
2.5. Electrostatics 52
2.6. Laminar Flow 1 75
2.7. Fast Mesh (Charge mapping, simple delivery) 97

1 Global Definitions

Date	Mar 2, 2023, 4:49:29 PM

GLOBAL SETTINGS

Version	COMSOL Multiphysics 6.0 (Build: 405)
Unit system	SI

USED PRODUCTS

COMSOL Multiphysics
Chemical Reaction Engineering Module

COMPUTER INFORMATION

CPU	Intel64 Family 6 Model 85 Stepping 4, 2 sockets, 8 cores
Operating system	Windows 10

1.1 PARAMETERS

1.1.1 Basic

BASIC

**Name**	**Expression**	**Value**	**Description**
cb	50[mmol/L]	50 mol/m³	Bulk Concentration
d	21*lR0	1.554E−6 m	probe-substrate distances parameter
dcalc	d - dd	7.4001E−8 m	
dd	1.4800181124567361E-6[m]	1.48E−6 m	
epsrH2O	78	78	relative permittivity of water at 25 C
mf	50	50	Mesh factor - controls number of points in each geometry length
numBL	20	20	
PoreSurfaceCharge	-0.028[C/m^2]	−0.028 C/m²	
SigmaSubstrate	-0.06999999999999999[C/m^2]	−0.07 C/m²	
T	25[degC]	298.15 K	
VApp	-0.05[V]	−0.05 V	
VPulse	-0.4[V]	−0.4 V	

1.1.2 Pipette Geometry Basic

PIPETTE GEOMETRY BASIC

Name	Expression	Value	Description
IR0	74.0009056228368[nm]	7.4001E−8 m	
IR50	1.022*IR0	7.5629E−8 m	
IR100	1.022*IR0	7.5629E−8 m	
IR200	1.111*IR0	8.2215E−8 m	
IR500	1.328*IR0	9.8273E−8 m	
IR1000	1.7*IR0	1.258E−7 m	
IR2000	2.467*IR0	1.8256E−7 m	
IR5000	4.306*IR0	3.1865E−7 m	
IR10000	5.571*IR0	4.1226E−7 m	not measured
OR0	1.461*IR0	1.0812E−7 m	
OR50	1.492*IR0	1.1041E−7 m	
OR100	1.498*IR0	1.1085E−7 m	
OR200	1.55*IR0	1.147E−7 m	
OR500	1.95*IR0	1.443E−7 m	
OR1000	2.4*IR0	1.776E−7 m	
OR2000	3.428*IR0	2.5368E−7 m	
OR5000	5.444*IR0	4.0286E−7 m	
OR10000	1.491*IR10000	6.1468E−7 m	not measured

1.1.3 Pipette Geometry Advanced

PIPETTE GEOMETRY ADVANCED

Name	Expression	Value	Description
ang_in	0.06[rad]	0.06 rad	
ang_in_2	ang_in	0.06 rad	
ang_o	0.08[rad]	0.08 rad	pipette semiangle
ang_o_2	ang_in_2 + 3[deg]	0.11236 rad	
Bh	Bw	1E−4 m	solution box height
Bw	100[um]	1E−4 m	solution box width
IR50000	IR10000 + ((50[um] - 10[um])*tan(ang_in))	2.8151E−6 m	
IR100000	IR50000 + ((100[um] - 50[um])*tan(ang_in))	5.8187E−6 m	
IRlp	IR50000 + ((lp - 50[um])*tan(ang_in_2))	3.5855E−5 m	

Name	Expression	Value	Description
OR50000	OR10000 + ((50[um] - 10[um])*tan(ang_o))	3.8215E−6 m	
OR100000	OR50000 + ((100[um] - 50[um])*tan(ang_o))	7.8301E−6 m	
ORlp	OR50000 + ((lp - 50[um])*tan(ang_o_2))	6.5881E−5 m	
IRtaper	0.35[mm]	3.5E−4 m	
taper	1[mm]	0.001 m	
lin	500[um]	5E−4 m	
lp	600[um]	6E−4 m	
lp_h	100.5[um]	1.005E−4 m	
ChargeBoundSub	max(15[um], NP_r + 1[um])	1.5E−5 m	
ChargeBoundSub1	OR0*1.5	1.6217E−7 m	
NP_r	50[nm]	5E−8 m	
fil_r	5[nm]	5E−9 m	

1.1.4 Speciation

SPECIATION

Name	Expression	Value	Description
DK	1.96e-5[cm^2/s]	1.96E−9 m^2/s	CRC handbook @ 25 degC
DCl	2.05e-5[cm^2/s]	2.05E−9 m^2/s	CRC handbook @ 25 degC
DNO3	1.902e-5[cm^2/s]	1.902E−9 m^2/s	

1.2 GEOMETRY PARTS

1.2.1 Pipette_Taper_BoxS

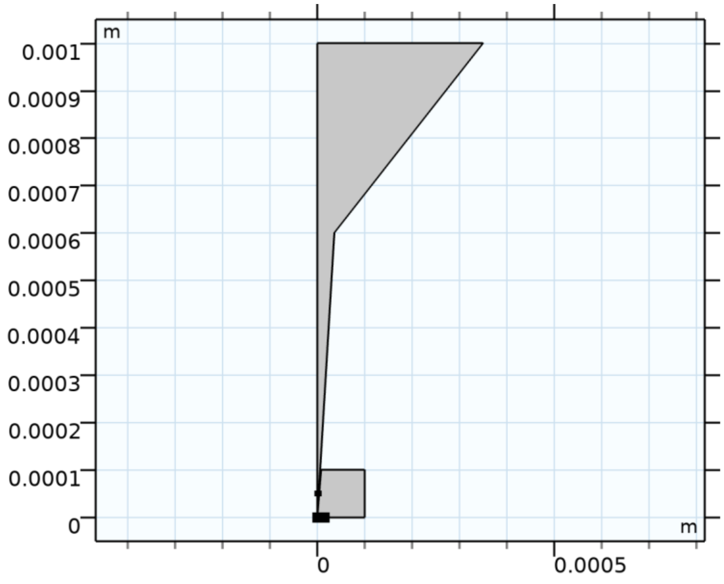

Pipette_Taper_BoxS

UNITS

Length unit	m
Angular unit	deg

GEOMETRY STATISTICS

Description	Value
Space dimension	2

PARAMETERS 1

Name	Expression	Value	Description
Bw_p	Bw	1E−4 m	solution box width
d_p	d	1.554E−6 m	probe-substrate distances parameter
IR0_p	IR0	7.4001E−8 m	
IR100_p	IR100	7.5629E−8 m	
IR50_p	IR50	7.5629E−8 m	
IR200_p	IR200	8.2215E−8 m	
IR500_p	IR500	9.8273E−8 m	
IR1000_p	IR1000	1.258E−7 m	
IR2000_p	IR2000	1.8256E−7 m	
IR5000_p	IR5000	3.1865E−7 m	

Name	Expression	Value	Description
IR10000_p	IR10000	4.1226E−7 m	
IR50000_p	IR50000	2.8151E−6 m	
IR100000_p	IR100000	5.8187E−6 m	
IRlp_p	IRlp	3.5855E−5 m	
IRtaper_p	IRtaper	3.5E−4 m	
lp_p	lp	6E−4 m	
OR0_p	OR0	1.0812E−7 m	
OR50_p	OR50	1.1041E−7 m	
OR100_p	OR100	1.1085E−7 m	
OR200_p	OR200	1.147E−7 m	
OR500_p	OR500	1.443E−7 m	
OR1000_p	OR1000	1.776E−7 m	
OR2000_p	OR2000	2.5368E−7 m	
OR5000_p	OR5000	4.0286E−7 m	
OR10000_p	OR10000	6.1468E−7 m	
OR50000_p	OR50000	3.8215E−6 m	
OR100000_p	OR100000	7.8301E−6 m	
taper_p	taper	0.001 m	
ChargeBoundSub_p	ChargeBoundSub	1.5E−5 m	
ChargeBoundSub1_p	ChargeBoundSub1	1.6217E−7 m	
fil_r_p	fil_r	5E−9 m	
ORlp_p	ORlp	6.5881E−5 m	

1.2.1.1 Inside Pipette (b1)

POLYGON SEGMENTS

Description	Value
Control points	{{IR50_p, 0, 0, 0, 0, 0, 0, 0, 0, 0, 0, 0, 0, IRtaper_p*0.8, IRtaper_p, IRlp_p, IR100000_p, IR50000_p, IR10000_p, IR5000_p, IR2000_p, IR1000_p, IR500_p, IR200_p, IR100_p, IR50_p}, {50[nm] + d_p, 50[nm] + d_p, 100[nm] + d_p, 200[nm] + d_p, 500[nm] + d_p, 1000[nm] + d_p, 2000[nm] + d_p, 5000[nm] + d_p, 10000[nm] + d_p, 50000[nm] + d_p, 100000[nm] + d_p, lp_p + d_p, taper_p + d_p, taper_p + d_p, taper_p + d_p, lp_p + d_p, 100[um] + d_p, 50[um] + d_p, 10[um] + d_p, 5[um] + d_p, 2[um] + d_p, 1[um] + d_p, 500[nm] + d_p, 200[nm] + d_p, 100[nm] + d_p, 50[nm] + d_p}}
Degree	{1, 1, 1, 1, 1, 1, 1, 1, 1, 1, 1, 1, 1, 1, 1, 1, 1, 1, 1, 1, 1, 1, 1, 1, 1}
Weights	{1, 1, 1, 1, 1, 1, 1, 1, 1, 1, 1, 1, 1, 1, 1, 1, 1, 1, 1, 1, 1, 1, 1, 1, 1, 1, 1, 1, 1, 1, 1, 1, 1, 1, 1, 1, 1, 1, 1, 1, 1, 1, 1, 1, 1, 1, 1, 1, 1, 1}
Type	Solid

1.2.1.2 Outside Domain (b2)

POLYGON SEGMENTS

Description	Value
Control points	{{OR100000_p, Bw_p, Bw_p, 0, 0, 0, IR50_p, IR0_p, OR0_p, OR50_p, OR100_p, OR200_p, OR500_p, OR1000_p, OR2000_p, OR5000_p, OR10000_p, OR50000_p, OR100000_p, OR100000_p}, {100[um] + d_p, 100[um] + d_p, 0, 0, 0[nm] + d_p, 50[nm] + d_p, 50[nm] + d_p, 0[nm] + d_p, 0[nm] + d_p, 50[nm] + d_p, 100[nm] + d_p, 200[nm] + d_p, 500[nm] + d_p, 1000[nm] + d_p, 2000[nm] + d_p, 5000[nm] + d_p, 10000[nm] + d_p, 50000[nm] + d_p, 100000[nm] + d_p, 100[um] + d_p}}
Degree	{1, 1, 1, 1, 1, 1, 1, 1, 1, 1, 1, 1, 1, 1, 1, 1, 1, 1, 1}
Weights	{1, 1, 1, 1, 1, 1, 1, 1, 1, 1, 1, 1, 1, 1, 1, 1, 1, 1, 1, 1, 1, 1, 1, 1, 1, 1, 1, 1, 1, 1, 1, 1, 1, 1, 1, 1, 1, 1}
Type	Solid

1.2.1.3 Charge Boundary Substrate (pt1)

POINT

Description	Value
Point coordinate	{1.4999999999999999E-5, 0}

1.2.1.4 Charge Boundary Substrate 1 (pt2)

POINT

Description	Value
Point coordinate	{1.6217298467244686E-7, 0}

1.2.1.5 Bézier Polygon 8 (b3)

POLYGON SEGMENTS

Description	Value
Control points	{{0, IR50000_p}, {50[um] + d_p, 50[um] + d_p}}
Degree	1
Weights	{1, 1}
Type	Solid

1.2.1.6 ToFiletEnd (boxsel31)

GEOMETRIC ENTITY LEVEL

Description	Value
Level	Point

OUTPUT ENTITIES

Description	Value
Include entity if	Entity intersects box

1.2.1.7 Fillet end (fil1)

SETTINGS

Description	Value
Radius	fil_r_p

1.2.1.8 ToFiletlp (boxsel34)

GEOMETRIC ENTITY LEVEL

Description	Value
Level	Point

OUTPUT ENTITIES

Description	Value
Include entity if	Entity intersects box

1.2.1.9 Fillet lp (fil2)

SETTINGS

Description	Value
Radius	500[nm]

1.3 SHARED PROPERTIES

1.3.1 Common model inputs 1

Tag	cminpt

2 Model 1

Date	Jul 15, 2014, 7:15:30 PM

SETTINGS

Description	Value
Unit system	Same as global system (SI)
Geometry shape function	Automatic
Avoid inverted elements by curving interior domain elements	Off

SPATIAL FRAME COORDINATES

First	Second	Third
r	phi	z

MATERIAL FRAME COORDINATES

First	Second	Third
R	PHI	Z

GEOMETRY FRAME COORDINATES

First	Second	Third
Rg	PHIg	Zg

MESH FRAME COORDINATES

First	Second	Third
Rm	PHIm	Zm

2.1 DEFINITIONS

2.1.1 Functions

2.1.1.1 Step 1

Function name	step1
Function type	Step

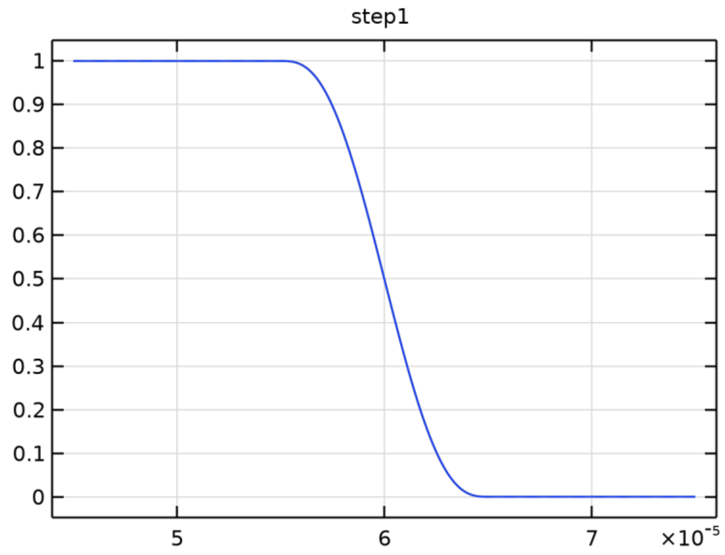

Step 1

PARAMETERS

Description	Value
Location	6e-5
From	1
To	0

SMOOTHING

Description	Value
Size of transition zone	1e-5

2.1.1.2 Step 2

Function name	step2
Function type	Step

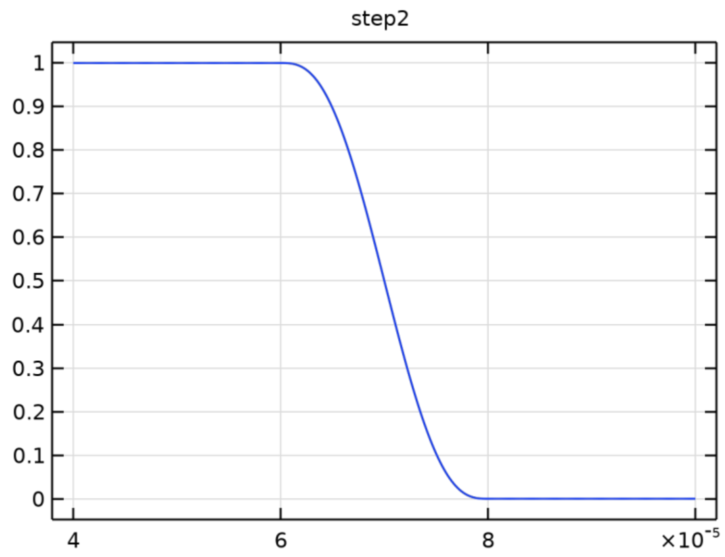

Step 2

PARAMETERS

Description	Value
Location	7e-5
From	1
To	0

SMOOTHING

Description	Value
Size of transition zone	2e-5

2.1.1.3 Step 3

Function name	step3
Function type	Step

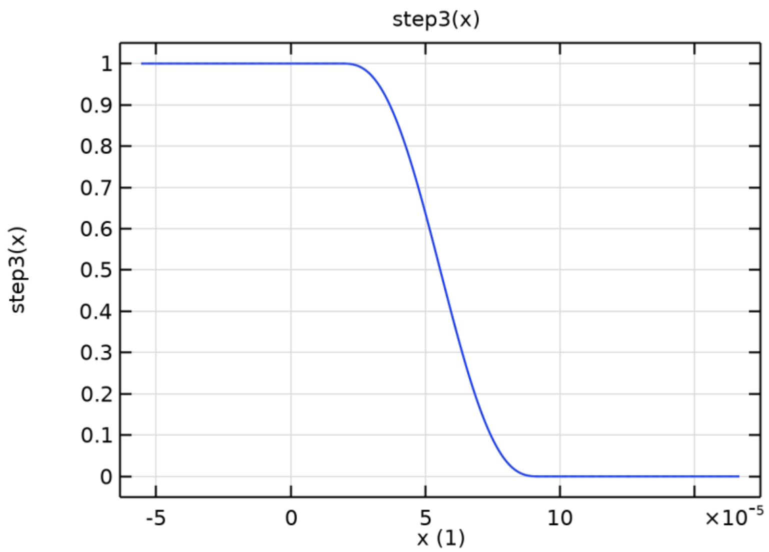

Step 3

PARAMETERS

Description	Value
Location	750*lR0/1[m]
From	1
To	0

SMOOTHING

Description	Value
Size of transition zone	1000*lR0/1[m]

2.1.2 Probes

2.1.2.1 Tip Potential

Probe type	Boundary probe

SELECTION

Geometric entity level	Boundary
Selection	Geometry geom1: Dimension 1: Boundary 17

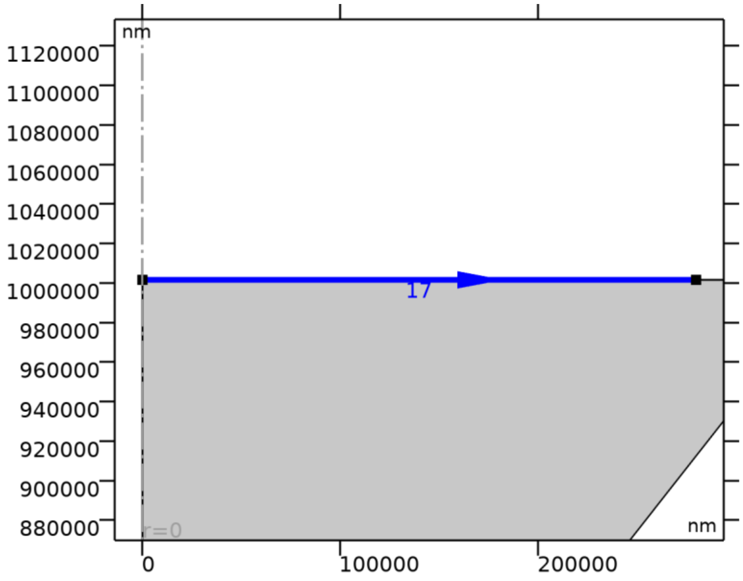

Selection

EXPRESSION

Description	Value
Expression	V
Table and plot unit	V
Description	Electric potential

TABLE AND WINDOW SETTINGS

Description	Value
Plot window	Probe Plot 6

2.1.2.2 Bulk Potential

Probe type	Boundary probe

SELECTION

Geometric entity level	Boundary
Name	Box Boundary (Part Instance 1)
Selection	Named geom1_pi1_boxsel27: Geometry geom1: Dimension 1: Boundary 44

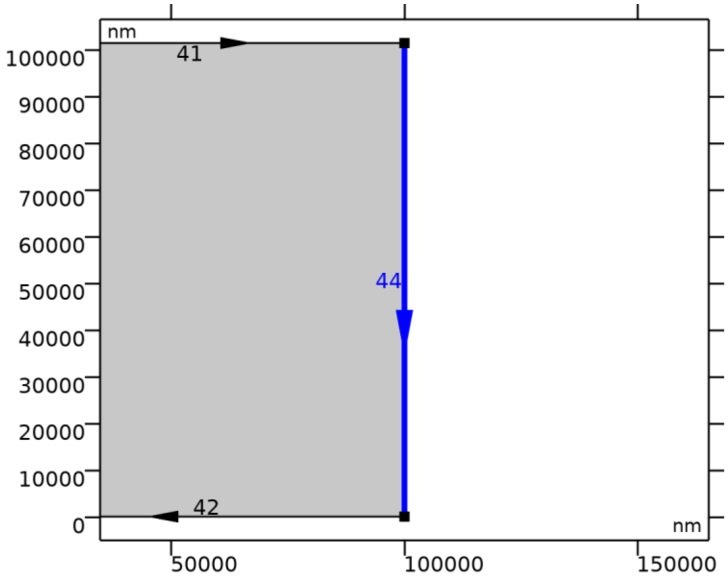

Selection

EXPRESSION

Description	Value
Expression	V
Table and plot unit	V
Description	Electric potential

TABLE AND WINDOW SETTINGS

Description	Value
Plot window	

2.1.2.3 DC Mid Boundary

Probe type	Boundary probe

SELECTION

Geometric entity level	Boundary
Selection	Geometry geom1: Dimension 1: Boundary 14

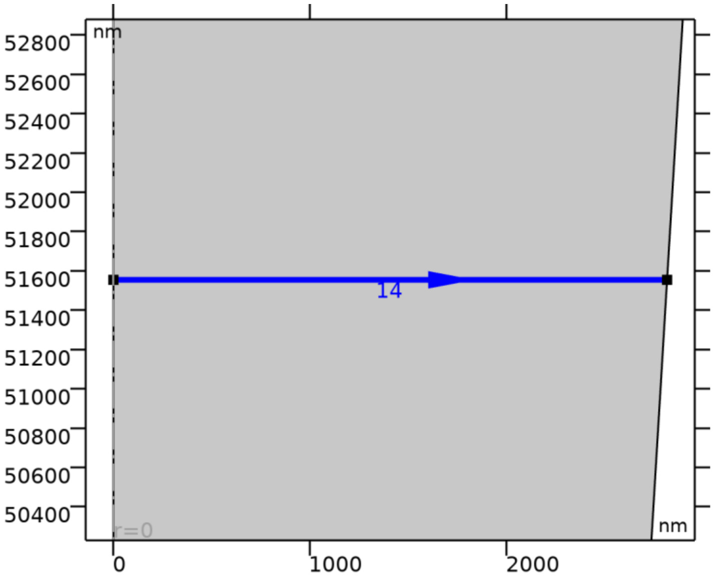

Selection

PROBE TYPE

Description	Value
Type	Integral

EXPRESSION

Description	Value
Expression	F_const*(chds.bndFlux_cK - chds.bndFlux_cNO3 - chds.bndFlux_cCl)*2*pi*r
Table and plot unit	nA
Description	Current (internal)

TABLE AND WINDOW SETTINGS

Description	Value
Plot window	

2.1.2.4 DC Bottom Boundary

Probe type	Boundary probe

SELECTION

Geometric entity level	Boundary
Selection	Geometry geom1: Dimension 1: Boundary 5

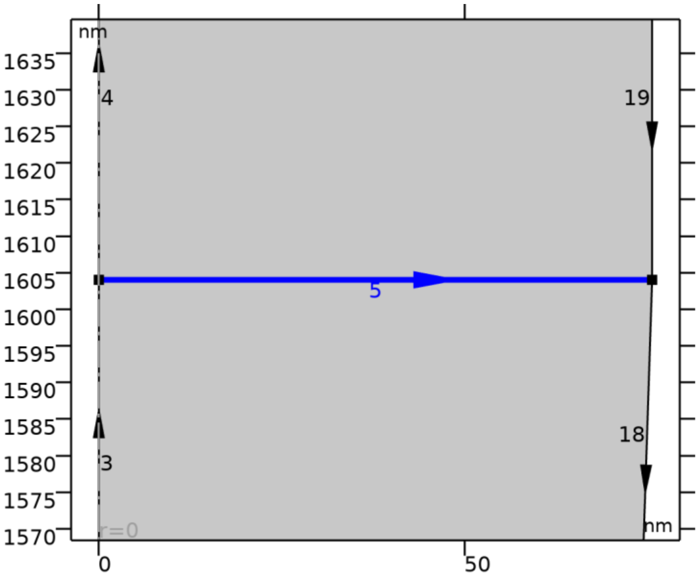

Selection

PROBE TYPE

Description	Value
Type	Integral

EXPRESSION

Description	Value
Expression	F_const*(chds.bndFlux_cK - chds.bndFlux_cNO3 - chds.bndFlux_cCl)
Table and plot unit	nA
Description	Current (internal)

TABLE AND WINDOW SETTINGS

Description	Value
Plot window	

2.1.3 Coordinate Systems

2.1.3.1 Boundary System 1

Coordinate system type	Boundary system
Tag	sys1

COORDINATE NAMES

First	Second	Third
t1	to	n

2.2 GEOMETRY

Pore with glass of infinite width

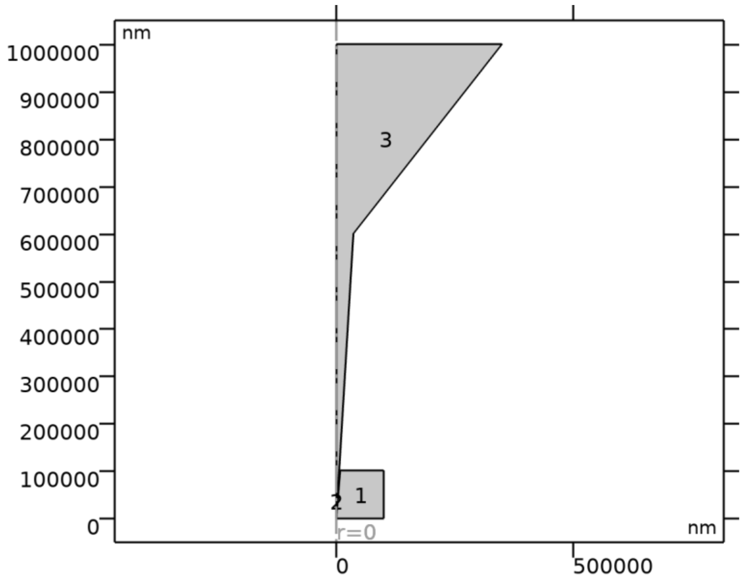

Geometry

UNITS

Length unit	nm
Angular unit	deg

GEOMETRY STATISTICS

Description	Value
Space dimension	2
Number of domains	3
Number of boundaries	48
Number of vertices	46

2.2.1 Rectangle 1 (r1)

POSITION

Description	Value
Position	{0, 0}

SIZE

Description	Value
Width	Bw
Height	dcalc

2.2.2 Part Instance 1 (pi1)

PART

Description	Value
Part	Pipette_Taper_BoxS

INPUT PARAMETERS

Name	Expression	Value	Description
Bw_p	Bw	99999.99999999999 [nm]	solution box width
d_p	d	1554.0190180795726 [nm]	probe-substrate distances parameter
IR0_p	IR0	74.0009056228368 [nm]	
IR100_p	IR100	75.6289255465392 [nm]	
IR50_p	IR50	75.6289255465392 [nm]	
IR200_p	IR200	82.21500614697167 [nm]	
IR500_p	IR500	98.27320266712726 [nm]	
IR1000_p	IR1000	125.80153955882253 [nm]	
IR2000_p	IR2000	182.56023417153838 [nm]	
IR5000_p	IR5000	318.6478996119352 [nm]	
IR10000_p	IR10000	412.25904522482375 [nm]	
IR50000_p	IR50000	2815.1431984767146 [nm]	
IR100000_p	IR100000	5818.748390041579 [nm]	
IRlp_p	IRlp	35854.800305690216 [nm]	
IRtaper_p	IRtaper	349999.99999999994 [nm]	
lp_p	lp	599999.9999999999 [nm]	
OR0_p	OR0	108.11532311496455 [nm]	
OR50_p	OR50	110.4093511892725 [nm]	
OR100_p	OR100	110.85335662300952 [nm]	
OR200_p	OR200	114.70140371539703 [nm]	
OR500_p	OR500	144.30176596453174 [nm]	
OR1000_p	OR1000	177.6021734948083 [nm]	
OR2000_p	OR2000	253.6751044750845 [nm]	
OR5000_p	OR5000	402.86093021072344 [nm]	
OR10000_p	OR10000	614.6782364302122 [nm]	
OR50000_p	OR50000	3821.5224247531132 [nm]	
OR100000_p	OR100000	7830.0776601567395 [nm]	
taper_p	taper	999999.9999999999 [nm]	
ChargeBoundSub_p	ChargeBoundSub	14999.999999999996 [nm]	

Name	Expression	Value	Description
ChargeBoundSub1_p	ChargeBoundSub1	162.17298467244683 [nm]	
fil_r_p	fil_r	4.999999999999999 [nm]	
ORlp_p	ORlp	65880.83609627905 [nm]	

POSITION AND ORIENTATION OF OUTPUT

Description	Value
Displacement	{0, 0}

2.2.3 2 IR0 (pt1)

POINT

Description	Value
Point coordinate	{148.0018112456736, 74.00090562283671}

2.2.4 15um (pt2)

POINT

Description	Value
Point coordinate	{14999.999999999998, 74.00090562283671}

2.3 MATERIALS

2.3.1 Water

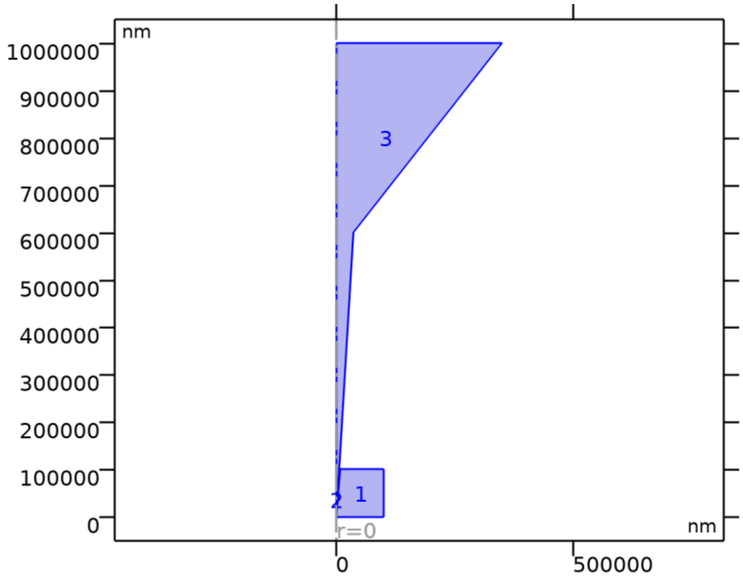

Water

SELECTION

Geometric entity level	Domain
Selection	Geometry geom1: Dimension 2: All domains

MATERIAL PARAMETERS

Name	Value	Unit
Dynamic viscosity	eta(T[1/K])[Pa*s]	Pa·s
Density	rho(T[1/K])[kg/m^3]	kg/m^3
Relative permittivity	epsrH2O	1

BASIC

Description	Value
Dynamic viscosity	eta(T[1/K])[Pa*s]
Ratio of specific heats	1.0
Electrical conductivity	{{5.5e-6[S/m], 0, 0}, {0, 5.5e-6[S/m], 0}, {0, 0, 5.5e-6[S/m]}}
Heat capacity at constant pressure	Cp(T[1/K])[J/(kg*K)]
Density	rho(T[1/K])[kg/m^3]
Thermal conductivity	{{k(T[1/K])[W/(m*K)], 0, 0}, {0, k(T[1/K])[W/(m*K)], 0}, {0, 0, k(T[1/K])[W/(m*K)]}}
Speed of sound	cs(T[1/K])[m/s]
Relative permittivity	{{epsrH2O, 0, 0}, {0, epsrH2O, 0}, {0, 0, epsrH2O}}

FUNCTIONS

Function name	Type
eta	Piecewise
Cp	Piecewise
rho	Piecewise
k	Piecewise
cs	Interpolation

2.3.1.1 Piecewise

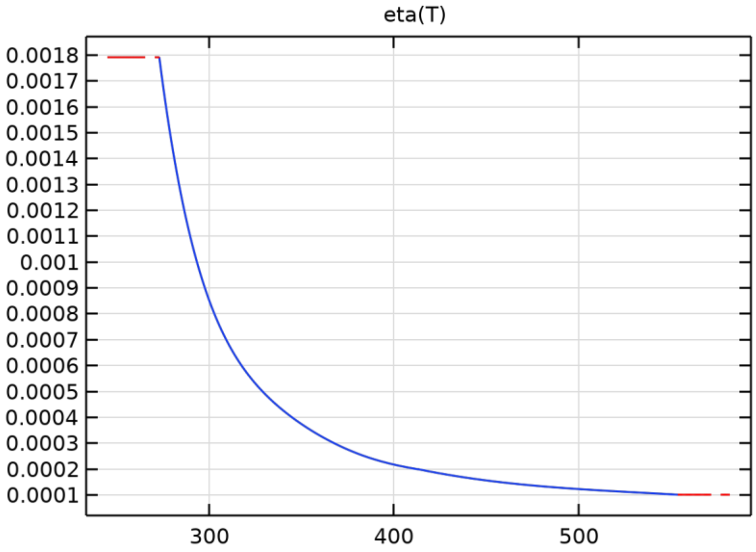

eta

2.3.1.2 Piecewise 2

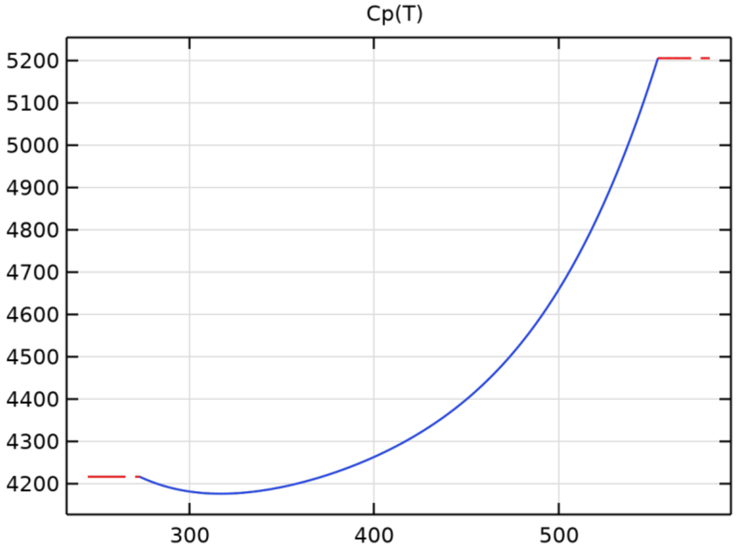

Cp

2.3.1.3 Piecewise 3

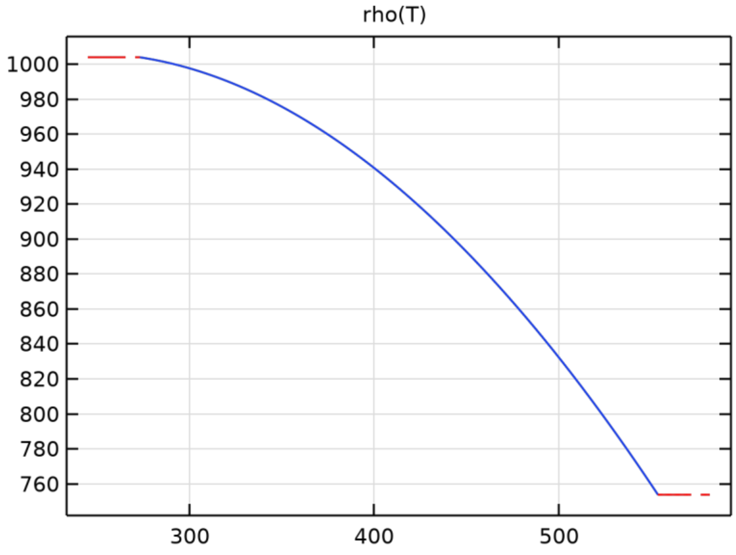

rho

2.3.1.4 Piecewise 4

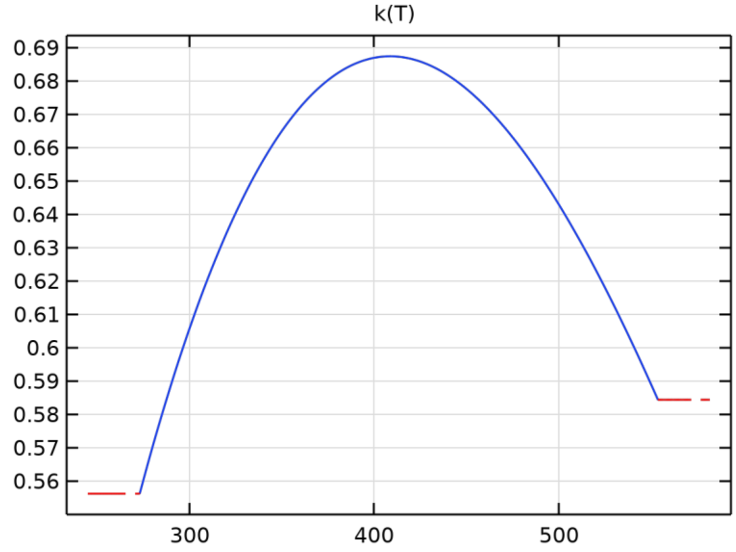

k

2.3.1.5 Interpolation

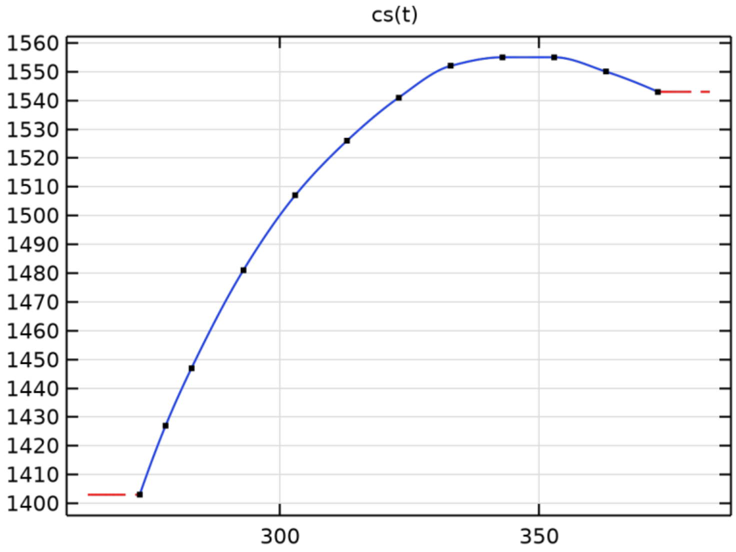

cs

2.4 TRANSPORT OF DILUTED SPECIES

USED PRODUCTS

COMSOL Multiphysics
Chemical Reaction Engineering Module

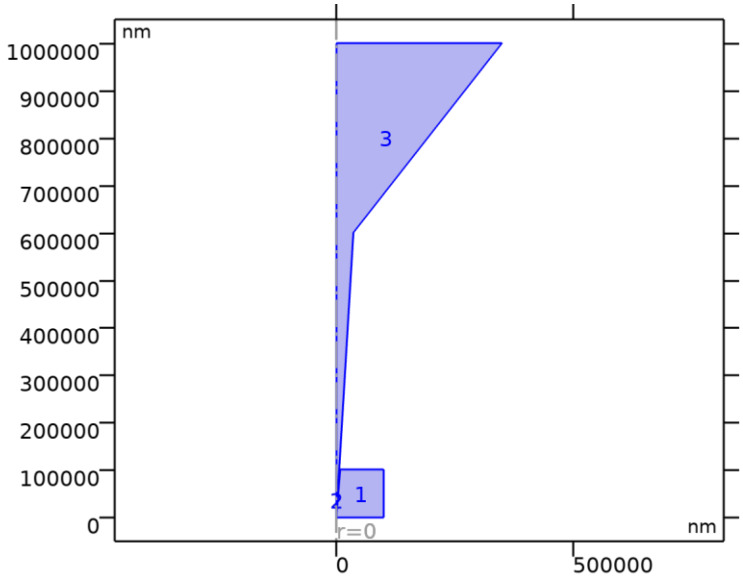

Transport of Diluted Species

SELECTION

Geometric entity level	Domain

Selection	Geometry geom1: Dimension 2: All domains

EQUATIONS

$$\nabla \cdot (\mathbf{J}_i + \mathbf{u} c_i) = R_i$$

$$\mathbf{J}_i = -D_i \nabla c_i - z_i u_{m,i} F c_i \nabla V$$

2.4.1 Interface Settings

2.4.1.1 Discretization

SETTINGS

Description	Value
Concentration	Quadratic

SETTINGS

Description	Value
Equation form	Study controlled

2.4.1.2 Advanced Settings

SETTINGS

Description	Value
Convective term	Conservative form

2.4.1.3 Transport Mechanisms

SETTINGS

Description	Value
Convection	On
Migration in electric field	On
Mass transfer in porous media	Off

2.4.2 Variables

Name	Expression	Unit	Description	Selection	Details
chds.d	1	1	Out-of-plane geometry extension	Global	
chds.nr	nr	1	Normal vector, r component	Boundaries 5, 14	
chds.nphi	0	1	Normal vector, phi component	Boundaries 5, 14	
chds.nz	nz	1	Normal vector, z component	Boundaries 5, 14	

Name	Expression	Unit	Description	Selection	Details
chds.nr	dnr	1	Normal vector, r component	Boundaries 1–4, 6–13, 15–48	
chds.nphi	0	1	Normal vector, phi component	Boundaries 1–4, 6–13, 15–48	
chds.nz	dnz	1	Normal vector, z component	Boundaries 1–4, 6–13, 15–48	
chds.nrmesh	nrmesh	1	Normal vector (mesh), r component	Boundaries 5, 14	
chds.nphimesh	0	1	Normal vector (mesh), phi component	Boundaries 5, 14	
chds.nzmesh	nzmesh	1	Normal vector (mesh), z component	Boundaries 5, 14	
chds.nrmesh	dnrmesh	1	Normal vector (mesh), r component	Boundaries 1–4, 6–13, 15–48	
chds.nphimesh	0	1	Normal vector (mesh), phi component	Boundaries 1–4, 6–13, 15–48	
chds.nzmesh	dnzmesh	1	Normal vector (mesh), z component	Boundaries 1–4, 6–13, 15–48	
chds.nrc	root.nrc/chds.ncLen	1	Normal vector, r component	Boundaries 1–48	
chds.nphic	0	1	Normal vector, phi component	Boundaries 1–48	
chds.nzc	root.nzc/chds.ncLen	1	Normal vector, z component	Boundaries 1–48	
chds.ncLen	sqrt(root.nrc^2+root.nzc^2+eps)	1	Help variable	Boundaries 1–48	
chds.cbf_cK	0	mol/(m²·s)	Convective boundary flux	Boundaries 1–48	
chds.u	0	m/s	Velocity field, r component	Domains 1–3	
chds.v	0	m/s	Velocity field, phi component	Domains 1–3	

Name	Expression	Unit	Description	Selection	Details
chds.w	0	m/s	Velocity field, z component	Domains 1–3	
chds.cbf_cNO3	0	mol/(m²·s)	Convective boundary flux	Boundaries 1–48	
chds.cbf_cCl	0	mol/(m²·s)	Convective boundary flux	Boundaries 1–48	
chds.R_cK	0	mol/(m³·s)	Total rate expression	Domains 1–3	+ operation
chds.cP_cK	0	mol/kg	Concentration species adsorbed to the solid	Domains 1–3	+ operation
chds.cP_cK	0	mol/kg	Concentration species adsorbed to the solid	Boundaries 1–48	+ operation
chds.KP_cK	0	m³/kg	Adsorption isotherm, first concentration derivative	Domains 1–3	+ operation
chds.KP_cK	0	m³/kg	Adsorption isotherm, first concentration derivative	Boundaries 1–48	+ operation
chds.Rads_cK	0	mol/(m³·s)	Total adsorption rate	Domains 1–3	+ operation
chds.DiT_cK	0	m²/s	Turbulent diffusivity	Domains 1–3	
chds.cVar_cK	cK	mol/m³	Species	Boundaries 1–48	
chds.R_cNO3	0	mol/(m³·s)	Total rate expression	Domains 1–3	+ operation
chds.cP_cNO3	0	mol/kg	Concentration species adsorbed to the solid	Domains 1–3	+ operation
chds.cP_cNO3	0	mol/kg	Concentration species adsorbed to the solid	Boundaries 1–48	+ operation
chds.KP_cNO3	0	m³/kg	Adsorption isotherm, first concentration derivative	Domains 1–3	+ operation
chds.KP_cNO3	0	m³/kg	Adsorption isotherm, first	Boundaries 1–48	+ operation

Name	Expression	Unit	Description	Selection	Details
			concentration derivative		
chds.Rads_cNO3	0	mol/(m³·s)	Total adsorption rate	Domains 1–3	+ operation
chds.DiT_cNO3	0	m²/s	Turbulent diffusivity	Domains 1–3	
chds.cVar_cNO3	cNO3	mol/m³	Species	Boundaries 1–48	
chds.R_cCl	0	mol/(m³·s)	Total rate expression	Domains 1–3	+ operation
chds.cP_cCl	0	mol/kg	Concentration species adsorbed to the solid	Domains 1–3	+ operation
chds.cP_cCl	0	mol/kg	Concentration species adsorbed to the solid	Boundaries 1–48	+ operation
chds.KP_cCl	0	m³/kg	Adsorption isotherm, first concentration derivative	Domains 1–3	+ operation
chds.KP_cCl	0	m³/kg	Adsorption isotherm, first concentration derivative	Boundaries 1–48	+ operation
chds.Rads_cCl	0	mol/(m³·s)	Total adsorption rate	Domains 1–3	+ operation
chds.DiT_cCl	0	m²/s	Turbulent diffusivity	Domains 1–3	
chds.cVar_cCl	cCl	mol/m³	Species	Boundaries 1–48	
chds.poro	1	1	Porosity	Domains 1–3	
chds.theta_g	0	1	Gas volume fraction	Domains 1–3	
chds.theta_l	1	1	Liquid volume fraction	Domains 1–3	
chds.theta	chds.poro	1	Mobile fluid volume fraction	Domains 1–3	

2.4.3 Convection, Diffusion, and Migration

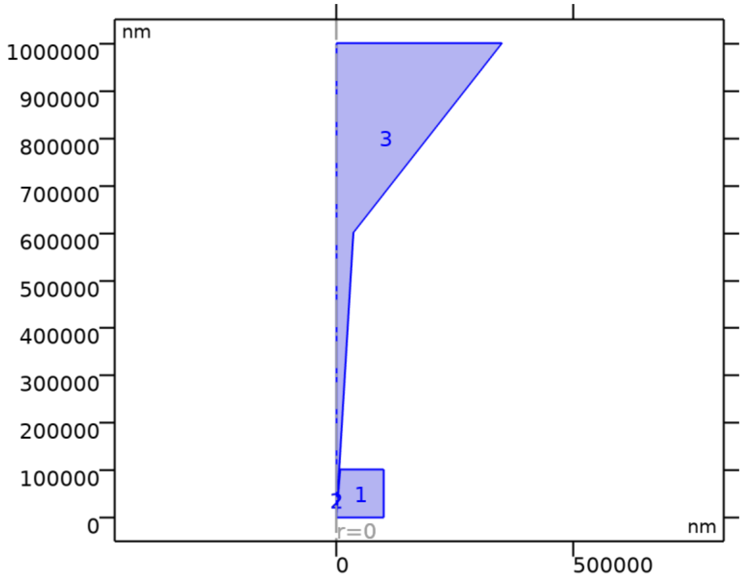

Convection, Diffusion, and Migration

SELECTION

Geometric entity level	Domain
Selection	Geometry geom1: Dimension 2: All domains

EQUATIONS

$$\nabla \cdot (\mathbf{J}_i + \mathbf{u}c_i) = R_i$$

$$\mathbf{J}_i = -D_i \nabla c_i - z_i u_{m,i} F c_i \nabla V$$

2.4.3.1 Convection

SETTINGS

**Description**	**Value**
Velocity field	Velocity field (spf2)

2.4.3.2 Diffusion

SETTINGS

**Description**	**Value**
Source	Material
Material	Water (mat1)
Diffusion coefficient	User defined
Diffusion coefficient	{{DK, 0, 0}, {0, DK, 0}, {0, 0, DK}}
Diffusion coefficient	User defined
Diffusion coefficient	{{DNO3, 0, 0}, {0, DNO3, 0}, {0, 0, DNO3}}

Description	Value
Diffusion coefficient	User defined
Diffusion coefficient	{{DCl, 0, 0}, {0, DCl, 0}, {0, 0, DCl}}

2.4.3.3 Migration in Electric Field

SETTINGS

Description	Value
Electric potential	Electric potential (es)
Mobility	Nernst - Einstein relation
Charge number	{1, -1, -1}

2.4.3.4 Coordinate System Selection

SETTINGS

Description	Value
Coordinate system	Global coordinate system

2.4.3.5 Model Input

SETTINGS

Description	Value
Temperature	User defined
Temperature	T

2.4.3.6 Variables

Name	Expression	Unit	Description	Selection	Details
domflux.cKr	2*(chds.dflux_cKr+chds.cflux_cKr+chds.mflux_cKr)*pi*r*chds.d	mol/(m·s)	Domain flux, r component	Domains 1–3	
domflux.cKz	2*(chds.dflux_cKz+chds.cflux_cKz+chds.mflux_cKz)*pi*r*chds.d	mol/(m·s)	Domain flux, z component	Domains 1–3	
domflux.cNO3r	2*(chds.dflux_cNO3r+chds.cflux_cNO3r+chds.mflux_cNO3r)*pi*r*chds.d	mol/(m·s)	Domain flux, r component	Domains 1–3	
domflux.cNO3z	2*(chds.dflux_cNO3z+chds.cflux_cNO3z+chds.mflux_cNO3z)*pi*r*chds.d	mol/(m·s)	Domain flux, z component	Domains 1–3	

Name	Expression	Unit	Description	Selection	Details
domflux.cClr	2*(chds.dflux_cClr+chds.cflux_cClr+chds.mflux_cClr)*pi*r*chds.d	mol/(m·s)	Domain flux, r component	Domains 1–3	
domflux.cClz	2*(chds.dflux_cClz+chds.cflux_cClz+chds.mflux_cClz)*pi*r*chds.d	mol/(m·s)	Domain flux, z component	Domains 1–3	
chds.ndflux_cK	chds.dflux_cKr*chds.nrc+chds.dflux_cKphi*chds.nphic+chds.dflux_cKz*chds.nzc	mol/(m²·s)	Normal diffusive flux	Boundaries 2, 5, 14, 17–48	
chds.ncflux_cK	chds.cflux_cKr*chds.nrc+chds.cflux_cKphi*chds.nphic+chds.cflux_cKz*chds.nzc	mol/(m²·s)	Normal convective flux	Boundaries 2, 5, 14, 17–48	
chds.nmflux_cK	chds.mflux_cKr*chds.nrc+chds.mflux_cKphi*chds.nphic+chds.mflux_cKz*chds.nzc	mol/(m²·s)	Normal electrophoretic flux	Boundaries 2, 5, 14, 17–48	
chds.ntflux_cK	chds.bndFlux_cK	mol/(m²·s)	Normal total flux	Boundaries 2, 5, 14, 17–48	
chds.ndflux_cNO3	chds.dflux_cNO3r*chds.nrc+chds.dflux_cNO3phi*chds.nphic+chds.dflux_cNO3z*chds.nzc	mol/(m²·s)	Normal diffusive flux	Boundaries 2, 5, 14, 17–48	
chds.ncflux_cNO3	chds.cflux_cNO3r*chds.nrc+chds.cflux_cNO3phi*chds.nphic+chds.cflux_cNO3z*chds.nzc	mol/(m²·s)	Normal convective flux	Boundaries 2, 5, 14, 17–48	
chds.nmflux_cNO3	chds.mflux_cNO3r*chds.nrc+chds.mflux_cNO3phi*chds.nphic+chds.mflux_cNO3z*chds.nzc	mol/(m²·s)	Normal electrophoretic flux	Boundaries 2, 5, 14, 17–48	
chds.ntflux_cNO3	chds.bndFlux_cNO3	mol/(m²·s)	Normal total flux	Boundaries 2, 5, 14, 17–48	
chds.ndflux_cCl	chds.dflux_cClr*chds.nrc+chds.dflux_cClphi*chds.nphic+ch	mol/(m²·s)	Normal diffusive flux	Boundaries 2, 5, 14, 17–48	

Name	Expression	Unit	Description	Selection	Details
	ds.dflux_cClz*chds.nzc				
chds.ncflux_cCl	chds.cflux_cClr*chds.nrc+chds.cflux_cClphi*chds.nphic+chds.cflux_cClz*chds.nzc	mol/(m²·s)	Normal convective flux	Boundaries 2, 5, 14, 17–48	
chds.nmflux_cCl	chds.mflux_cClr*chds.nrc+chds.mflux_cClphi*chds.nphic+chds.mflux_cClz*chds.nzc	mol/(m²·s)	Normal electrophoretic flux	Boundaries 2, 5, 14, 17–48	
chds.ntflux_cCl	chds.bndFlux_cCl	mol/(m²·s)	Normal total flux	Boundaries 2, 5, 14, 17–48	
chds.u	model.input.u1	m/s	Velocity field, r component	Domains 1–3	Meta
chds.v	model.input.u2	m/s	Velocity field, phi component	Domains 1–3	Meta
chds.w	model.input.u3	m/s	Velocity field, z component	Domains 1–3	Meta
chds.bndFlux_cK	0.25*(uflux_spatial(cK)-dflux_spatial(cK))/(pi*r*chds.d)	mol/(m²·s)	Boundary flux	Boundaries 5, 14	Meta
chds.bndFlux_cK	-dflux_spatial(cK)/chds.d	mol/(m²·s)	Boundary flux	Boundaries 1, 3–4, 6–13, 15–16	
chds.bndFlux_cK	-0.5*dflux_spatial(cK)/(pi*r*chds.d)	mol/(m²·s)	Boundary flux	Boundaries 2, 17–48	Meta
chds.bndFlux_cNO3	0.25*(uflux_spatial(cNO3)-dflux_spatial(cNO3))/(pi*r*chds.d)	mol/(m²·s)	Boundary flux	Boundaries 5, 14	Meta
chds.bndFlux_cNO3	-dflux_spatial(cNO3)/chds.d	mol/(m²·s)	Boundary flux	Boundaries 1, 3–4, 6–13, 15–16	
chds.bndFlux_cNO3	-0.5*dflux_spatial(cNO3)/(pi*r*chds.d)	mol/(m²·s)	Boundary flux	Boundaries 2, 17–48	Meta
chds.bndFlux_cCl	0.25*(uflux_spatial(cCl)-	mol/(m²·s)	Boundary flux	Boundaries 5, 14	Meta

Name	Expression	Unit	Description	Selection	Details
	dflux_spatial(cCl))/(pi*r*chds.d)				
chds.bndFlux_cCl	-dflux_spatial(cCl)/chds.d	mol/(m²·s)	Boundary flux	Boundaries 1, 3–4, 6–13, 15–16	
chds.bndFlux_cCl	-0.5*dflux_spatial(cCl)/(pi*r*chds.d)	mol/(m²·s)	Boundary flux	Boundaries 2, 17–48	Meta
chds.DF_cKrr	DK	m²/s	Fluid diffusion coefficient, rr component	Domains 1–3	
chds.DF_cKphir	0	m²/s	Fluid diffusion coefficient, phir component	Domains 1–3	
chds.DF_cKzr	0	m²/s	Fluid diffusion coefficient, zr component	Domains 1–3	
chds.DF_cKrphi	0	m²/s	Fluid diffusion coefficient, rphi component	Domains 1–3	
chds.DF_cKphiphi	DK	m²/s	Fluid diffusion coefficient, phiphi component	Domains 1–3	
chds.DF_cKzphi	0	m²/s	Fluid diffusion coefficient, zphi component	Domains 1–3	
chds.DF_cKrz	0	m²/s	Fluid diffusion coefficient, rz component	Domains 1–3	
chds.DF_cKphiz	0	m²/s	Fluid diffusion coefficient, phiz component	Domains 1–3	
chds.DF_cKzz	DK	m²/s	Fluid diffusion coefficient, zz component	Domains 1–3	
chds.D_cKrr	chds.DF_cKrr+chds.DiT_cK	m²/s	Diffusion coefficient, rr component	Domains 1–3	
chds.D_cKphir	chds.DF_cKphir	m²/s	Diffusion coefficient, phir component	Domains 1–3	

Name	Expression	Unit	Description	Selection	Details
chds.D_cKzr	chds.DF_cKzr	m^2/s	Diffusion coefficient, zr component	Domains 1–3	
chds.D_cKrphi	chds.DF_cKrphi	m^2/s	Diffusion coefficient, rphi component	Domains 1–3	
chds.D_cKphiphi	chds.DF_cKphiphi+chds.DiT_cK	m^2/s	Diffusion coefficient, phiphi component	Domains 1–3	
chds.D_cKzphi	chds.DF_cKzphi	m^2/s	Diffusion coefficient, zphi component	Domains 1–3	
chds.D_cKrz	chds.DF_cKrz	m^2/s	Diffusion coefficient, rz component	Domains 1–3	
chds.D_cKphiz	chds.DF_cKphiz	m^2/s	Diffusion coefficient, phiz component	Domains 1–3	
chds.D_cKzz	chds.DF_cKzz+chds.DiT_cK	m^2/s	Diffusion coefficient, zz component	Domains 1–3	
chds.DF_cNO3rr	DNO3	m^2/s	Fluid diffusion coefficient, rr component	Domains 1–3	
chds.DF_cNO3phir	0	m^2/s	Fluid diffusion coefficient, phir component	Domains 1–3	
chds.DF_cNO3zr	0	m^2/s	Fluid diffusion coefficient, zr component	Domains 1–3	
chds.DF_cNO3rphi	0	m^2/s	Fluid diffusion coefficient, rphi component	Domains 1–3	
chds.DF_cNO3phiphi	DNO3	m^2/s	Fluid diffusion coefficient, phiphi component	Domains 1–3	
chds.DF_cNO3zphi	0	m^2/s	Fluid diffusion coefficient, zphi component	Domains 1–3	

Name	Expression	Unit	Description	Selection	Details
chds.DF_cNO3rz	0	m²/s	Fluid diffusion coefficient, rz component	Domains 1–3	
chds.DF_cNO3phiz	0	m²/s	Fluid diffusion coefficient, phiz component	Domains 1–3	
chds.DF_cNO3zz	DNO3	m²/s	Fluid diffusion coefficient, zz component	Domains 1–3	
chds.D_cNO3rr	chds.DF_cNO3rr+chds.DiT_cNO3	m²/s	Diffusion coefficient, rr component	Domains 1–3	
chds.D_cNO3phir	chds.DF_cNO3phir	m²/s	Diffusion coefficient, phir component	Domains 1–3	
chds.D_cNO3zr	chds.DF_cNO3zr	m²/s	Diffusion coefficient, zr component	Domains 1–3	
chds.D_cNO3rphi	chds.DF_cNO3rphi	m²/s	Diffusion coefficient, rphi component	Domains 1–3	
chds.D_cNO3phiphi	chds.DF_cNO3phiphi+chds.DiT_cNO3	m²/s	Diffusion coefficient, phiphi component	Domains 1–3	
chds.D_cNO3zphi	chds.DF_cNO3zphi	m²/s	Diffusion coefficient, zphi component	Domains 1–3	
chds.D_cNO3rz	chds.DF_cNO3rz	m²/s	Diffusion coefficient, rz component	Domains 1–3	
chds.D_cNO3phiz	chds.DF_cNO3phiz	m²/s	Diffusion coefficient, phiz component	Domains 1–3	
chds.D_cNO3zz	chds.DF_cNO3zz+chds.DiT_cNO3	m²/s	Diffusion coefficient, zz component	Domains 1–3	
chds.DF_cClrr	DCl	m²/s	Fluid diffusion coefficient, rr component	Domains 1–3	
chds.DF_cClphir	0	m²/s	Fluid diffusion coefficient, phir component	Domains 1–3	

Name	Expression	Unit	Description	Selection	Details
chds.DF_cClzr	0	m²/s	Fluid diffusion coefficient, zr component	Domains 1–3	
chds.DF_cClrphi	0	m²/s	Fluid diffusion coefficient, rphi component	Domains 1–3	
chds.DF_cClphiphi	DCl	m²/s	Fluid diffusion coefficient, phiphi component	Domains 1–3	
chds.DF_cClzphi	0	m²/s	Fluid diffusion coefficient, zphi component	Domains 1–3	
chds.DF_cClrz	0	m²/s	Fluid diffusion coefficient, rz component	Domains 1–3	
chds.DF_cClphiz	0	m²/s	Fluid diffusion coefficient, phiz component	Domains 1–3	
chds.DF_cClzz	DCl	m²/s	Fluid diffusion coefficient, zz component	Domains 1–3	
chds.D_cClrr	chds.DF_cClrr+chds.DiT_cCl	m²/s	Diffusion coefficient, rr component	Domains 1–3	
chds.D_cClphir	chds.DF_cClphir	m²/s	Diffusion coefficient, phir component	Domains 1–3	
chds.D_cClzr	chds.DF_cClzr	m²/s	Diffusion coefficient, zr component	Domains 1–3	
chds.D_cClrphi	chds.DF_cClrphi	m²/s	Diffusion coefficient, rphi component	Domains 1–3	
chds.D_cClphiphi	chds.DF_cClphiphi+chds.DiT_cCl	m²/s	Diffusion coefficient, phiphi component	Domains 1–3	
chds.D_cClzphi	chds.DF_cClzphi	m²/s	Diffusion coefficient, zphi component	Domains 1–3	

Name	Expression	Unit	Description	Selection	Details
chds.D_cClrz	chds.DF_cClrz	m²/s	Diffusion coefficient, rz component	Domains 1–3	
chds.D_cClphiz	chds.DF_cClphiz	m²/s	Diffusion coefficient, phiz component	Domains 1–3	
chds.D_cClzz	chds.DF_cClzz+chds.DiT_cCl	m²/s	Diffusion coefficient, zz component	Domains 1–3	
chds.Dav_cK	0.5*(chds.D_cKrr+chds.D_cKzz)	m²/s	Average diffusion coefficient	Domains 1–3	
chds.Dav_cNO3	0.5*(chds.D_cNO3rr+chds.D_cNO3zz)	m²/s	Average diffusion coefficient	Domains 1–3	
chds.Dav_cCl	0.5*(chds.D_cClrr+chds.D_cClzz)	m²/s	Average diffusion coefficient	Domains 1–3	
chds.tflux_cKr	chds.dflux_cKr+chds.mflux_cKr+chds.cflux_cKr	mol/(m²·s)	Total flux, r component	Domains 1–3	+ operation
chds.tflux_cKphi	chds.dflux_cKphi+chds.mflux_cKphi+chds.cflux_cKphi	mol/(m²·s)	Total flux, phi component	Domains 1–3	+ operation
chds.tflux_cKz	chds.dflux_cKz+chds.mflux_cKz+chds.cflux_cKz	mol/(m²·s)	Total flux, z component	Domains 1–3	+ operation
chds.dfluxMag_cK	sqrt(chds.dflux_cKr^2+chds.dflux_cKphi^2+chds.dflux_cKz^2)	mol/(m²·s)	Diffusive flux magnitude	Domains 1–3	
chds.tfluxMag_cK	sqrt(chds.tflux_cKr^2+chds.tflux_cKphi^2+chds.tflux_cKz^2)	mol/(m²·s)	Total flux magnitude	Domains 1–3	
chds.dpflux_cKr	0	mol/(m²·s)	Dispersive flux, r component	Domains 1–3	
chds.dpflux_cKphi	0	mol/(m²·s)	Dispersive flux, phi component	Domains 1–3	
chds.dpflux_cKz	0	mol/(m²·s)	Dispersive flux, z component	Domains 1–3	
chds.mflux_cKr	chds.z_cK*F_const*cK*(-chds.um_cKrr*d(chds.V,r)-	mol/(m²·s)	Electrophoretic flux, r component	Domains 1–3	

Name	Expression	Unit	Description	Selection	Details
	chds.um_cKrz*d(chds.V,z))				
chds.mflux_cKphi	chds.z_cK*F_const*cK*(-chds.um_cKphir*d(chds.V,r)-chds.um_cKphiz*d(chds.V,z))	mol/(m²·s)	Electrophoretic flux, phi component	Domains 1–3	
chds.mflux_cKz	chds.z_cK*F_const*cK*(-chds.um_cKzr*d(chds.V,r)-chds.um_cKzz*d(chds.V,z))	mol/(m²·s)	Electrophoretic flux, z component	Domains 1–3	
chds.mfluxMag_cK	sqrt(chds.mflux_cKr^2+chds.mflux_cKphi^2+chds.mflux_cKz^2)	mol/(m²·s)	Electrophoretic flux magnitude	Domains 1–3	
chds.tflux_cNO3r	chds.dflux_cNO3r+chds.mflux_cNO3r+chds.cflux_cNO3r	mol/(m²·s)	Total flux, r component	Domains 1–3	+ operation
chds.tflux_cNO3phi	chds.dflux_cNO3phi+chds.mflux_cNO3phi+chds.cflux_cNO3phi	mol/(m²·s)	Total flux, phi component	Domains 1–3	+ operation
chds.tflux_cNO3z	chds.dflux_cNO3z+chds.mflux_cNO3z+chds.cflux_cNO3z	mol/(m²·s)	Total flux, z component	Domains 1–3	+ operation
chds.dfluxMag_cNO3	sqrt(chds.dflux_cNO3r^2+chds.dflux_cNO3phi^2+chds.dflux_cNO3z^2)	mol/(m²·s)	Diffusive flux magnitude	Domains 1–3	
chds.tfluxMag_cNO3	sqrt(chds.tflux_cNO3r^2+chds.tflux_cNO3phi^2+chds.tflux_cNO3z^2)	mol/(m²·s)	Total flux magnitude	Domains 1–3	
chds.dpflux_cNO3r	0	mol/(m²·s)	Dispersive flux, r component	Domains 1–3	
chds.dpflux_cNO3phi	0	mol/(m²·s)	Dispersive flux, phi component	Domains 1–3	
chds.dpflux_cNO3z	0	mol/(m²·s)	Dispersive flux, z component	Domains 1–3	

Name	Expression	Unit	Description	Selection	Details
chds.mflux_cNO3r	chds.z_cNO3*F_const*cNO3*(-chds.um_cNO3rr*d(chds.V,r)-chds.um_cNO3rz*d(chds.V,z))	mol/(m²·s)	Electrophoretic flux, r component	Domains 1–3	
chds.mflux_cNO3phi	chds.z_cNO3*F_const*cNO3*(-chds.um_cNO3phir*d(chds.V,r)-chds.um_cNO3phiz*d(chds.V,z))	mol/(m²·s)	Electrophoretic flux, phi component	Domains 1–3	
chds.mflux_cNO3z	chds.z_cNO3*F_const*cNO3*(-chds.um_cNO3zr*d(chds.V,r)-chds.um_cNO3zz*d(chds.V,z))	mol/(m²·s)	Electrophoretic flux, z component	Domains 1–3	
chds.mfluxMag_cNO3	sqrt(chds.mflux_cNO3r^2+chds.mflux_cNO3phi^2+chds.mflux_cNO3z^2)	mol/(m²·s)	Electrophoretic flux magnitude	Domains 1–3	
chds.tflux_cClr	chds.dflux_cClr+chds.mflux_cClr+chds.cflux_cClr	mol/(m²·s)	Total flux, r component	Domains 1–3	+ operation
chds.tflux_cClphi	chds.dflux_cClphi+chds.mflux_cClphi+chds.cflux_cClphi	mol/(m²·s)	Total flux, phi component	Domains 1–3	+ operation
chds.tflux_cClz	chds.dflux_cClz+chds.mflux_cClz+chds.cflux_cClz	mol/(m²·s)	Total flux, z component	Domains 1–3	+ operation
chds.dfluxMag_cCl	sqrt(chds.dflux_cClr^2+chds.dflux_cClphi^2+chds.dflux_cClz^2)	mol/(m²·s)	Diffusive flux magnitude	Domains 1–3	
chds.tfluxMag_cCl	sqrt(chds.tflux_cClr^2+chds.tflux_cClphi^2+chds.tflux_cClz^2)	mol/(m²·s)	Total flux magnitude	Domains 1–3	
chds.dpflux_cClr	0	mol/(m²·s)	Dispersive flux, r component	Domains 1–3	
chds.dpflux_cClphi	0	mol/(m²·s)	Dispersive flux, phi component	Domains 1–3	

Name	Expression	Unit	Description	Selection	Details
chds.dpflux_cClz	0	mol/(m²·s)	Dispersive flux, z component	Domains 1–3	
chds.mflux_cClr	chds.z_cCl*F_const*cCl*(-chds.um_cClrr*d(chds.V,r)-chds.um_cClrz*d(chds.V,z))	mol/(m²·s)	Electrophoretic flux, r component	Domains 1–3	
chds.mflux_cClphi	chds.z_cCl*F_const*cCl*(-chds.um_cClphir*d(chds.V,r)-chds.um_cClphiz*d(chds.V,z))	mol/(m²·s)	Electrophoretic flux, phi component	Domains 1–3	
chds.mflux_cClz	chds.z_cCl*F_const*cCl*(-chds.um_cClzr*d(chds.V,r)-chds.um_cClzz*d(chds.V,z))	mol/(m²·s)	Electrophoretic flux, z component	Domains 1–3	
chds.mfluxMag_cCl	sqrt(chds.mflux_cClr^2+chds.mflux_cClphi^2+chds.mflux_cClz^2)	mol/(m²·s)	Electrophoretic flux magnitude	Domains 1–3	
chds.dflux_cKr	-chds.D_cKrr*cKr-chds.D_cKrz*cKz	mol/(m²·s)	Diffusive flux, r component	Domains 1–3	+ operation
chds.dflux_cKphi	-chds.D_cKphir*cKr-chds.D_cKphiz*cKz	mol/(m²·s)	Diffusive flux, phi component	Domains 1–3	+ operation
chds.dflux_cKz	-chds.D_cKzr*cKr-chds.D_cKzz*cKz	mol/(m²·s)	Diffusive flux, z component	Domains 1–3	+ operation
chds.grad_cKr	cKr	mol/m⁴	Concentration gradient, r component	Domains 1–3	
chds.grad_cKphi	0	mol/m⁴	Concentration gradient, phi component	Domains 1–3	
chds.grad_cKz	cKz	mol/m⁴	Concentration gradient, z component	Domains 1–3	
chds.dflux_cNO3r	-chds.D_cNO3rr*cNO3r-	mol/(m²·s)	Diffusive flux, r component	Domains 1–3	+ operation

Name	Expression	Unit	Description	Selection	Details
	chds.D_cNO3rz*cNO3z				
chds.dflux_cNO3phi	-chds.D_cNO3phir*cNO3r-chds.D_cNO3phiz*cNO3z	mol/(m²·s)	Diffusive flux, phi component	Domains 1–3	+ operation
chds.dflux_cNO3z	-chds.D_cNO3zr*cNO3r-chds.D_cNO3zz*cNO3z	mol/(m²·s)	Diffusive flux, z component	Domains 1–3	+ operation
chds.grad_cNO3r	cNO3r	mol/m⁴	Concentration gradient, r component	Domains 1–3	
chds.grad_cNO3phi	0	mol/m⁴	Concentration gradient, phi component	Domains 1–3	
chds.grad_cNO3z	cNO3z	mol/m⁴	Concentration gradient, z component	Domains 1–3	
chds.dflux_cClr	-chds.D_cClrr*cClr-chds.D_cClrz*cClz	mol/(m²·s)	Diffusive flux, r component	Domains 1–3	+ operation
chds.dflux_cClphi	-chds.D_cClphir*cClr-chds.D_cClphiz*cClz	mol/(m²·s)	Diffusive flux, phi component	Domains 1–3	+ operation
chds.dflux_cClz	-chds.D_cClzr*cClr-chds.D_cClzz*cClz	mol/(m²·s)	Diffusive flux, z component	Domains 1–3	+ operation
chds.grad_cClr	cClr	mol/m⁴	Concentration gradient, r component	Domains 1–3	
chds.grad_cClphi	0	mol/m⁴	Concentration gradient, phi component	Domains 1–3	
chds.grad_cClz	cClz	mol/m⁴	Concentration gradient, z component	Domains 1–3	
chds.um_cKrr	chds.D_cKrr/(R_const*chds.T)	s·mol/kg	Mobility, rr component	Domains 1–3	
chds.um_cKphir	chds.D_cKphir/(R_const*chds.T)	s·mol/kg	Mobility, phir component	Domains 1–3	

Name	Expression	Unit	Description	Selection	Details
chds.um_cKzr	chds.D_cKzr/(R_const*chds.T)	s·mol/kg	Mobility, zr component	Domains 1–3	
chds.um_cKrphi	chds.D_cKrphi/(R_const*chds.T)	s·mol/kg	Mobility, rphi component	Domains 1–3	
chds.um_cKphiphi	chds.D_cKphiphi/(R_const*chds.T)	s·mol/kg	Mobility, phiphi component	Domains 1–3	
chds.um_cKzphi	chds.D_cKzphi/(R_const*chds.T)	s·mol/kg	Mobility, zphi component	Domains 1–3	
chds.um_cKrz	chds.D_cKrz/(R_const*chds.T)	s·mol/kg	Mobility, rz component	Domains 1–3	
chds.um_cKphiz	chds.D_cKphiz/(R_const*chds.T)	s·mol/kg	Mobility, phiz component	Domains 1–3	
chds.um_cKzz	chds.D_cKzz/(R_const*chds.T)	s·mol/kg	Mobility, zz component	Domains 1–3	
chds.z_cK	1	1	Charge number	Domains 1–3	
chds.um_cNO3rr	chds.D_cNO3rr/(R_const*chds.T)	s·mol/kg	Mobility, rr component	Domains 1–3	
chds.um_cNO3phir	chds.D_cNO3phir/(R_const*chds.T)	s·mol/kg	Mobility, phir component	Domains 1–3	
chds.um_cNO3zr	chds.D_cNO3zr/(R_const*chds.T)	s·mol/kg	Mobility, zr component	Domains 1–3	
chds.um_cNO3rphi	chds.D_cNO3rphi/(R_const*chds.T)	s·mol/kg	Mobility, rphi component	Domains 1–3	
chds.um_cNO3phiphi	chds.D_cNO3phiphi/(R_const*chds.T)	s·mol/kg	Mobility, phiphi component	Domains 1–3	
chds.um_cNO3zphi	chds.D_cNO3zphi/(R_const*chds.T)	s·mol/kg	Mobility, zphi component	Domains 1–3	
chds.um_cNO3rz	chds.D_cNO3rz/(R_const*chds.T)	s·mol/kg	Mobility, rz component	Domains 1–3	
chds.um_cNO3phiz	chds.D_cNO3phiz/(R_const*chds.T)	s·mol/kg	Mobility, phiz component	Domains 1–3	
chds.um_cNO3zz	chds.D_cNO3zz/(R_const*chds.T)	s·mol/kg	Mobility, zz component	Domains 1–3	
chds.z_cNO3	-1	1	Charge number	Domains 1–3	
chds.um_cClrr	chds.D_cClrr/(R_const*chds.T)	s·mol/kg	Mobility, rr component	Domains 1–3	
chds.um_cClphir	chds.D_cClphir/(R_const*chds.T)	s·mol/kg	Mobility, phir component	Domains 1–3	
chds.um_cClzr	chds.D_cClzr/(R_const*chds.T)	s·mol/kg	Mobility, zr component	Domains 1–3	

Name	Expression	Unit	Description	Selection	Details
chds.um_cClrphi	chds.D_cClrphi/(R_const*chds.T)	s·mol/kg	Mobility, rphi component	Domains 1–3	
chds.um_cClphiphi	chds.D_cClphiphi/(R_const*chds.T)	s·mol/kg	Mobility, phiphi component	Domains 1–3	
chds.um_cClzphi	chds.D_cClzphi/(R_const*chds.T)	s·mol/kg	Mobility, zphi component	Domains 1–3	
chds.um_cClrz	chds.D_cClrz/(R_const*chds.T)	s·mol/kg	Mobility, rz component	Domains 1–3	
chds.um_cClphiz	chds.D_cClphiz/(R_const*chds.T)	s·mol/kg	Mobility, phiz component	Domains 1–3	
chds.um_cClzz	chds.D_cClzz/(R_const*chds.T)	s·mol/kg	Mobility, zz component	Domains 1–3	
chds.z_cCl	-1	1	Charge number	Domains 1–3	
chds.V	model.input.V	V	Electric potential	Domains 1–3	Meta
chds.T	chds.cdm1.minput_temperature	K	Temperature	Domains 1–3	
chds.cflux_cKr	cK*chds.u	mol/(m²·s)	Convective flux, r component	Domains 1–3	
chds.cflux_cKphi	cK*chds.v	mol/(m²·s)	Convective flux, phi component	Domains 1–3	
chds.cflux_cKz	cK*chds.w	mol/(m²·s)	Convective flux, z component	Domains 1–3	
chds.cfluxMag_cK	sqrt(chds.cflux_cKr^2+chds.cflux_cKphi^2+chds.cflux_cKz^2)	mol/(m²·s)	Convective flux magnitude	Domains 1–3	
chds.cflux_cNO3r	cNO3*chds.u	mol/(m²·s)	Convective flux, r component	Domains 1–3	
chds.cflux_cNO3phi	cNO3*chds.v	mol/(m²·s)	Convective flux, phi component	Domains 1–3	
chds.cflux_cNO3z	cNO3*chds.w	mol/(m²·s)	Convective flux, z component	Domains 1–3	
chds.cfluxMag_cNO3	sqrt(chds.cflux_cNO3r^2+chds.cflux_cNO3phi^2+chds.cflux_cNO3z^2)	mol/(m²·s)	Convective flux magnitude	Domains 1–3	
chds.cflux_cClr	cCl*chds.u	mol/(m²·s)	Convective flux, r component	Domains 1–3	
chds.cflux_cClphi	cCl*chds.v	mol/(m²·s)	Convective flux, phi component	Domains 1–3	

Name	Expression	Unit	Description	Selection	Details
chds.cflux_cClz	cCl*chds.w	mol/(m²·s)	Convective flux, z component	Domains 1–3	
chds.cfluxMag_cCl	sqrt(chds.cflux_cClr^2+chds.cflux_cClphi^2+chds.cflux_cClz^2)	mol/(m²·s)	Convective flux magnitude	Domains 1–3	
chds.Res_cK	-chds.D_cKrr*cKrr-chds.D_cKrz*cKrz-chds.D_cKzr*cKzr-chds.D_cKzz*cKzz+d(cK*(chds.u-chds.z_cK*chds.um_cKrr*F_const*d(chds.V,r)-chds.z_cK*chds.um_cKrz*F_const*d(chds.V,z)),r)+if(abs(r)<0.001*h,d(cK*(chds.u-chds.z_cK*chds.um_cKrr*F_const*d(chds.V,r)-chds.z_cK*chds.um_cKrz*F_const*d(chds.V,z)),r),cK*(chds.u-chds.z_cK*chds.um_cKrr*F_const*d(chds.V,r)-chds.z_cK*chds.um_cKrz*F_const*d(chds.V,z))/r)+d(cK*(chds.w-chds.z_cK*chds.um_cKzr*F_const*d(chds.V,r)-chds.z_cK*chds.um_cKzz*F_const*d(chds.V,z)),z)-chds.R_cK	mol/(m³·s)	Equation residual	Domains 1–3	
chds.Rlin_cK	0		Linear source term coefficient	Domains 1–3	
chds.Res_cNO3	-chds.D_cNO3rr*cNO3rr-chds.D_cNO3rz*cN	mol/(m³·s)	Equation residual	Domains 1–3	

Name	Expression	Unit	Description	Selection	Details
	O3rz-chds.D_cNO3zr*cNO3zr-chds.D_cNO3zz*cNO3zz+d(cNO3*(chds.u-chds.z_cNO3*chds.um_cNO3rr*F_const*d(chds.V,r)-chds.z_cNO3*chds.um_cNO3rz*F_const*d(chds.V,z)),r)+if(abs(r)<0.001*h,d(cNO3*(chds.u-chds.z_cNO3*chds.um_cNO3rr*F_const*d(chds.V,r)-chds.z_cNO3*chds.um_cNO3rz*F_const*d(chds.V,z)),r),cNO3*(chds.u-chds.z_cNO3*chds.um_cNO3rr*F_const*d(chds.V,r)-chds.z_cNO3*chds.um_cNO3rz*F_const*d(chds.V,z))/r)+d(cNO3*(chds.w-chds.z_cNO3*chds.um_cNO3zr*F_const*d(chds.V,r)-chds.z_cNO3*chds.um_cNO3zz*F_const*d(chds.V,z)),z)-chds.R_cNO3				
chds.Rlin_cNO3	0		Linear source term coefficient	Domains 1–3	
chds.Res_cCl	-chds.D_cClrr*cClrr-chds.D_cClrz*cClrz-chds.D_cClzr*cClzr-chds.D_cClzz*cClzz+d(cCl*(chds.u-chds.z_cCl*chds.um_cClrr*F_const*d(chds.V,r)-chds.z_cCl*chds.um_cClrz*F_const*d(ch	$mol/(m^3\cdot s)$	Equation residual	Domains 1–3	

Name	Expression	Unit	Description	Selection	Details
	ds.V,z)),r)+if(abs(r)<0.001*h,d(cCl*(chds.u-chds.z_cCl*chds.um_cClrr*F_const*d(chds.V,r)-chds.z_cCl*chds.um_cClrz*F_const*d(chds.V,z)),r),cCl*(chds.u-chds.z_cCl*chds.um_cClrr*F_const*d(chds.V,r)-chds.z_cCl*chds.um_cClrz*F_const*d(chds.V,z))/r)+d(cCl*(chds.w-chds.z_cCl*chds.um_cClzr*F_const*d(chds.V,r)-chds.z_cCl*chds.um_cClzz*F_const*d(chds.V,z)),z)-chds.R_cCl				
chds.Rlin_cCl	0		Linear source term coefficient	Domains 1–3	

2.4.3.7 Shape functions

Name	Shape function	Unit	Description	Shape frame	Selection
cK	Lagrange (Quadratic)	mol/m³	Concentration	Material	Domains 1–3
cNO3	Lagrange (Quadratic)	mol/m³	Concentration	Material	Domains 1–3
cCl	Lagrange (Quadratic)	mol/m³	Concentration	Material	Domains 1–3

2.4.3.8 Weak Expressions

Weak expression	Integration order	Integration frame	Selection
2*(chds.dflux_cKr*test(cKr)+chds.dflux_cKz*test(cKz))*chds.d*pi*r	4	Material	Domains 1–3
2*(chds.dflux_cNO3r*test(cNO3r)+chds.dflux_cNO3z*test(cNO3z))*chds.d*pi*r	4	Material	Domains 1–3
2*(chds.dflux_cClr*test(cClr)+chds.dflux_cClz*test(cClz))*chds.d*pi*r	4	Material	Domains 1–3

Weak expression	Integration order	Integration frame	Selection
2*chds.z_cK*F_const*cK*((-chds.um_cKrr*d(chds.V,r)-chds.um_cKrz*d(chds.V,z))*test(cKr)+(-chds.um_cKzr*d(chds.V,r)-chds.um_cKzz*d(chds.V,z))*test(cKz))*chds.d*pi*r	4	Material	Domains 1–3
2*chds.z_cNO3*F_const*cNO3*((-chds.um_cNO3rr*d(chds.V,r)-chds.um_cNO3rz*d(chds.V,z))*test(cNO3r)+(-chds.um_cNO3zr*d(chds.V,r)-chds.um_cNO3zz*d(chds.V,z))*test(cNO3z))*chds.d*pi*r	4	Material	Domains 1–3
2*chds.z_cCl*F_const*cCl*((-chds.um_cClrr*d(chds.V,r)-chds.um_cClrz*d(chds.V,z))*test(cClr)+(-chds.um_cClzr*d(chds.V,r)-chds.um_cClzz*d(chds.V,z))*test(cClz))*chds.d*pi*r	4	Material	Domains 1–3
2*cK*(chds.u*test(cKr)+chds.w*test(cKz))*(isScalingSystemDomain==0)*chds.d*pi*r	4	Material	Domains 1–3
2*chds.cbf_cK*test(cK)*chds.d*pi*r	4	Material	Boundaries 1–48
2*cNO3*(chds.u*test(cNO3r)+chds.w*test(cNO3z))*(isScalingSystemDomain==0)*chds.d*pi*r	4	Material	Domains 1–3
2*chds.cbf_cNO3*test(cNO3)*chds.d*pi*r	4	Material	Boundaries 1–48
2*cCl*(chds.u*test(cClr)+chds.w*test(cClz))*(isScalingSystemDomain==0)*chds.d*pi*r	4	Material	Domains 1–3
2*chds.cbf_cCl*test(cCl)*chds.d*pi*r	4	Material	Boundaries 1–48
2*chds.streamline*(isScalingSystemDomain==0)*chds.d*pi*r	4	Material	Domains 1–3
2*chds.crosswind*(isScalingSystemDomain==0)*chds.d*pi*r	6	Material	Domains 1–3

2.4.4 Axial Symmetry 1

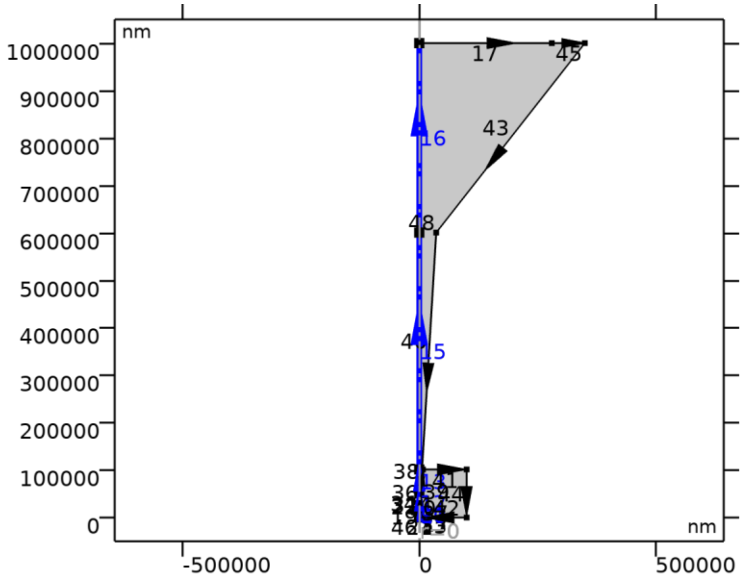

Axial Symmetry 1

SELECTION

Geometric entity level	Boundary
Selection	Geometry geom1: Dimension 1: All boundaries

2.4.5 No Flux 1

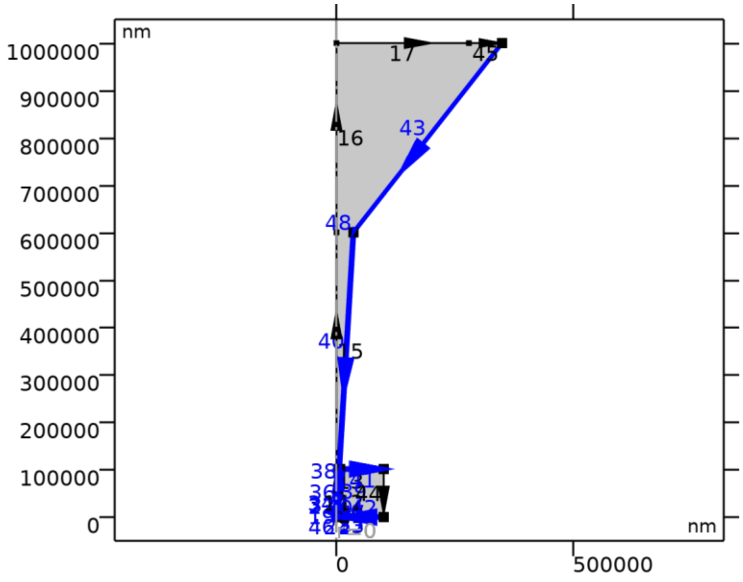

No Flux 1

SELECTION

Geometric entity level	Boundary

Selection	Geometry geom1: Dimension 1: All boundaries

EQUATIONS

$$-\mathbf{n}\cdot\mathbf{J}_i = 0$$

2.4.5.1 Convection

SETTINGS

Description	Value
Include	Off

2.4.5.2 Variables

Name	Expression	Unit	Description	Selection
chds.cbf_cK	cK*(-chds.u*chds.nrmesh-chds.v*chds.nphimesh-chds.w*chds.nzmesh)	mol/(m²·s)	Convective boundary flux	Boundaries 2, 18–43, 46–48
chds.cbf_cNO3	cNO3*(-chds.u*chds.nrmesh-chds.v*chds.nphimesh-chds.w*chds.nzmesh)	mol/(m²·s)	Convective boundary flux	Boundaries 2, 18–43, 46–48
chds.cbf_cCl	cCl*(-chds.u*chds.nrmesh-chds.v*chds.nphimesh-chds.w*chds.nzmesh)	mol/(m²·s)	Convective boundary flux	Boundaries 2, 18–43, 46–48

2.4.6 Initial Values 1

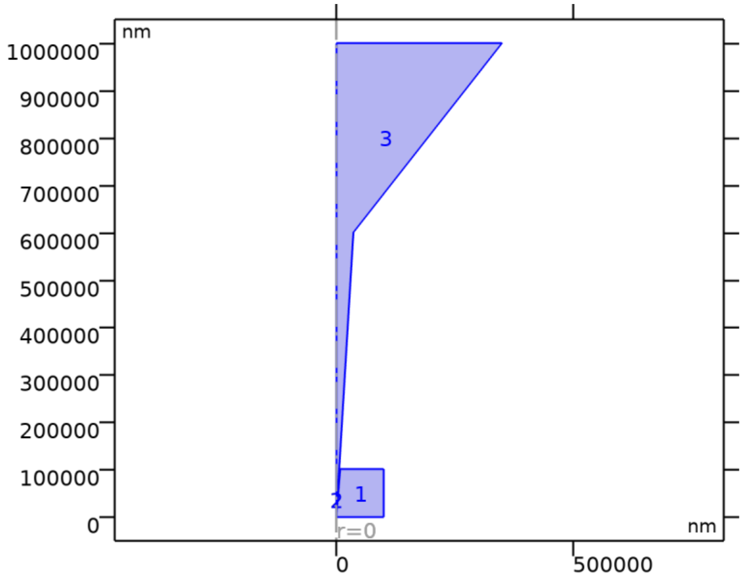

Initial Values 1

SELECTION

Geometric entity level	Domain

Selection	Geometry geom1: Dimension 2: All domains

2.4.6.1 Initial Values

SETTINGS

Description	Value
Concentration	{50[mM], 45[mM], 5[mM]}

2.4.6.2 Variables

Name	Expression	Unit	Description	Selection	Details
chds.c0_cK	50[mM]	mol/m^3	Concentration	Domains 1–3	+ operation
chds.c0_cNO3	45[mM]	mol/m^3	Concentration	Domains 1–3	+ operation
chds.c0_cCl	5[mM]	mol/m^3	Concentration	Domains 1–3	+ operation

2.4.7 Concentration 1

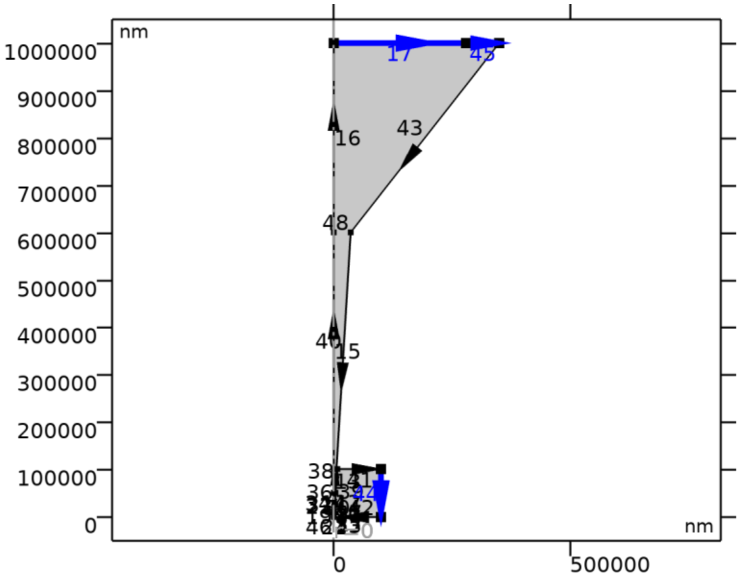

Concentration 1

SELECTION

Geometric entity level	Boundary
Selection	Geometry geom1: Dimension 1: Boundaries 17, 44–45

EQUATIONS

$$c_i = c_{0,i}$$

2.4.7.1 Concentration

SETTINGS

Description	Value
Species cK	On
Species cNO3	On
Species cCl	On
Concentration	{50[mM], 45[mM], 5[mM]}

2.4.7.2 Constraint Settings

SETTINGS

Description	Value
Apply reaction terms on	All physics (symmetric)
Use weak constraints	Off
Constraint method	Elemental

2.4.7.3 Variables

Name	Expression	Unit	Description	Selection	Details
chds.c0_cK	50[mM]	mol/m³	Concentration	Boundaries 17, 44–45	+ operation
chds.c0_cNO3	45[mM]	mol/m³	Concentration	Boundaries 17, 44–45	+ operation
chds.c0_cCl	5[mM]	mol/m³	Concentration	Boundaries 17, 44–45	+ operation
chds.conc1.nmflow_cK	chds.conc1.int(2*chds.ntflux_cK*pi*r)*chds.d	mol/s	Normal molar flow rate	Global	
chds.conc1.nmflow_cNO3	chds.conc1.int(2*chds.ntflux_cNO3*pi*r)*chds.d	mol/s	Normal molar flow rate	Global	
chds.conc1.nmflow_cCl	chds.conc1.int(2*chds.ntflux_cCl*pi*r)*chds.d	mol/s	Normal molar flow rate	Global	

2.4.7.4 Constraints

Constraint	Constraint force	Shape function	Selection	Details
-chds.cVar_cK+chds.c0_cK	test(-chds.cVar_cK+chds.c0_cK)	Lagrange (Quadratic)	Boundaries 17, 44–45	Elemental
-chds.cVar_cNO3+chds.c0_cNO3	test(-chds.cVar_cNO3+chds.c0_cNO3)	Lagrange (Quadratic)	Boundaries 17, 44–45	Elemental

Constraint	Constraint force	Shape function	Selection	Details
-chds.cVar_cCl+chds.c0_cCl	test(-chds.cVar_cCl+chds.c0_cCl)	Lagrange (Quadratic)	Boundaries 17, 44–45	Elemental

2.5 ELECTROSTATICS

USED PRODUCTS

COMSOL Multiphysics

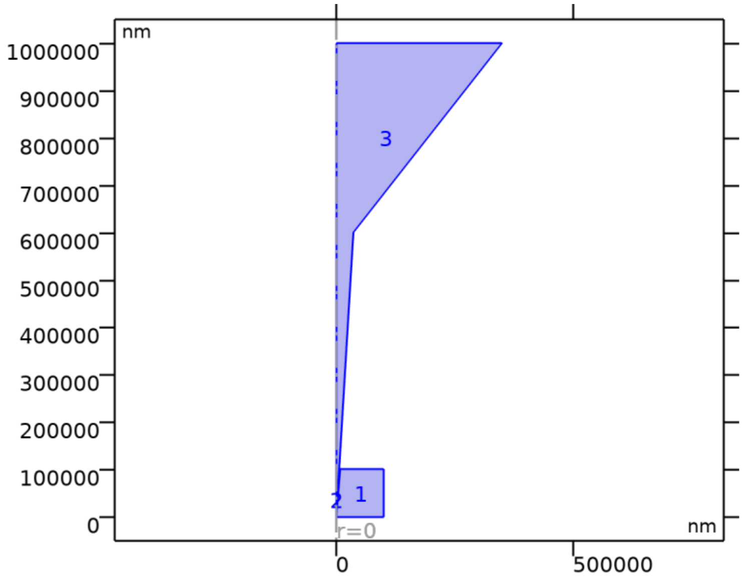

Electrostatics

SELECTION

Geometric entity level	Domain
Selection	Geometry geom1: Dimension 2: All domains

EQUATIONS

$$\nabla \cdot \mathbf{D} = \rho_v$$
$$\mathbf{E} = -\nabla V$$

2.5.1 Interface Settings

2.5.1.1 Discretization

SETTINGS

Description	Value
Electric potential	Quadratic
Value type when using splitting of complex variables	Complex

2.5.1.2 Manual Terminal Sweep Settings

SETTINGS

Description	Value
Use manual terminal sweep	Off
Reference impedance	50[ohm]

2.5.2 Variables

Name	Expression	Unit	Description	Selection	Details
es.d	1	1	Contribution	Domains 1–3	
es.nr	nr		Normal vector, r component	Boundaries 5, 14	
es.nphi	0		Normal vector, phi component	Boundaries 5, 14	
es.nz	nz		Normal vector, z component	Boundaries 5, 14	
es.nr	dnr		Normal vector, r component	Boundaries 1–4, 6–13, 15–48	
es.nphi	0		Normal vector, phi component	Boundaries 1–4, 6–13, 15–48	
es.nz	dnz		Normal vector, z component	Boundaries 1–4, 6–13, 15–48	
es.nmeshr	nrmesh		Mesh normal vector, r component	Boundaries 5, 14	
es.nmeshphi	0		Mesh normal vector, phi component	Boundaries 5, 14	
es.nmeshz	nzmesh		Mesh normal vector, z component	Boundaries 5, 14	
es.nmeshr	dnrmesh		Mesh normal vector, r component	Boundaries 1–4, 6–13, 15–48	
es.nmeshphi	0		Mesh normal vector, phi component	Boundaries 1–4, 6–13, 15–48	
es.nmeshz	dnzmesh		Mesh normal vector, z component	Boundaries 1–4, 6–13, 15–48	

Name	Expression	Unit	Description	Selection	Details
es.unmeshr	unrmesh		Mesh normal vector, upside, r component	Boundaries 1–48	
es.unmeshphi	0		Mesh normal vector, upside, phi component	Boundaries 1–48	
es.unmeshz	unzmesh		Mesh normal vector, upside, z component	Boundaries 1–48	
es.dnmeshr	dnrmesh		Mesh normal vector, downside, r component	Boundaries 1–48	
es.dnmeshphi	0		Mesh normal vector, downside, phi component	Boundaries 1–48	
es.dnmeshz	dnzmesh		Mesh normal vector, downside, z component	Boundaries 1–48	
es.I_srr	1	1	Spatial identity matrix, material frame, rr component	Domains 1–3	
es.I_sphir	0	1	Spatial identity matrix, material frame, phir component	Domains 1–3	
es.I_szr	0	1	Spatial identity matrix, material frame, zr component	Domains 1–3	
es.I_srphi	0	1	Spatial identity matrix, material frame, rphi component	Domains 1–3	
es.I_sphiphi	1	1	Spatial identity matrix, material frame, phiphi component	Domains 1–3	
es.I_szphi	0	1	Spatial identity matrix, material	Domains 1–3	

Name	Expression	Unit	Description	Selection	Details
			frame, zphi component		
es.I_srz	0	1	Spatial identity matrix, material frame, rz component	Domains 1–3	
es.I_sphiz	0	1	Spatial identity matrix, material frame, phiz component	Domains 1–3	
es.I_szz	1	1	Spatial identity matrix, material frame, zz component	Domains 1–3	
es.unTr	es.unTer	Pa	Maxwell upward surface stress tensor, r component	Boundaries 1–48	
es.unTphi	es.unTephi	Pa	Maxwell upward surface stress tensor, phi component	Boundaries 1–48	
es.unTz	es.unTez	Pa	Maxwell upward surface stress tensor, z component	Boundaries 1–48	
es.dnTr	es.dnTer	Pa	Maxwell downward surface stress tensor, r component	Boundaries 1–48	
es.dnTphi	es.dnTephi	Pa	Maxwell downward surface stress tensor, phi component	Boundaries 1–48	
es.dnTz	es.dnTez	Pa	Maxwell downward surface stress tensor, z component	Boundaries 1–48	
es.unr	unr		Normal vector up direction, r component	Boundaries 1–48	

Name	Expression	Unit	Description	Selection	Details
es.unphi	0		Normal vector up direction, phi component	Boundaries 1–48	
es.unz	unz		Normal vector up direction, z component	Boundaries 1–48	
es.dnr	dnr		Normal vector down direction, r component	Boundaries 1–48	
es.dnphi	0		Normal vector down direction, phi component	Boundaries 1–48	
es.dnz	dnz		Normal vector down direction, z component	Boundaries 1–48	
es.unTer	-0.5*es.dnr*(real(up(es.Dr))*real(up(es.Er))+real(up(es.Dphi))*real(up(es.Ephi))+real(up(es.Dz))*real(up(es.Ez)))+real(up(es.Dr))*(real(up(es.Er))*es.dnr+real(up(es.Ephi))*es.dnphi+real(up(es.Ez))*es.dnz)	Pa	Maxwell upward electric surface stress tensor, r component	Boundaries 5, 14	
es.unTephi	-0.5*es.dnphi*(real(up(es.Dr))*real(up(es.Er))+real(up(es.Dphi))*real(up(es.Ephi))+real(up(es.Dz))*real(up(es.Ez)))+real(up(es.Dphi))*(real(up(es.Er))*es.dnr+real(up(es.Ephi))*es.dnphi+real(up(es.Ez))*es.dnz)	Pa	Maxwell upward electric surface stress tensor, phi component	Boundaries 5, 14	
es.unTez	-0.5*es.dnz*(real(up(es.Dr))*real(up(es.Er))+real(up(es.Dphi))*real(up(es.Ephi))+real(up(es.Dz))*real(up(es.Ez)))+real(up(es.Dz))*(real(up(es.Er))*es.dnr+re	Pa	Maxwell upward electric surface stress tensor, z component	Boundaries 5, 14	

Name	Expression	Unit	Description	Selection	Details
	al(up(es.Ephi))*es.dnphi+real(up(es.Ez))*es.dnz)				
es.unTer	0	Pa	Maxwell upward electric surface stress tensor, r component	Boundaries 1–4, 6–13, 15–48	
es.unTephi	0	Pa	Maxwell upward electric surface stress tensor, phi component	Boundaries 1–4, 6–13, 15–48	
es.unTez	0	Pa	Maxwell upward electric surface stress tensor, z component	Boundaries 1–4, 6–13, 15–48	
es.dnTer	-0.5*es.unr*(real(down(es.Dr))*real(down(es.Er))+real(down(es.Dphi))*real(down(es.Ephi))+real(down(es.Dz))*real(down(es.Ez)))+real(down(es.Dr))*(real(down(es.Er))*es.unr+real(down(es.Ephi))*es.unphi+real(down(es.Ez))*es.unz)	Pa	Maxwell downward electric surface stress tensor, r component	Boundaries 1–48	
es.dnTephi	-0.5*es.unphi*(real(down(es.Dr))*real(down(es.Er))+real(down(es.Dphi))*real(down(es.Ephi))+real(down(es.Dz))*real(down(es.Ez)))+real(down(es.Dphi))*(real(down(es.Er))*es.unr+real(down(es.Ephi))*es.unphi+real(down(es.Ez))*es.unz)	Pa	Maxwell downward electric surface stress tensor, phi component	Boundaries 1–48	
es.dnTez	-0.5*es.unz*(real(down(es.Dr))*real(down(es.Er))+real(down(es.Dphi))*real(down(es.Ephi))+real(down(es.Dz))*	Pa	Maxwell downward electric surface stress tensor, z component	Boundaries 1–48	

Name	Expression	Unit	Description	Selection	Details
	real(down(es.Ez)))+real(down(es.Dz))*(real(down(es.Er))*es.unr+real(down(es.Ephi))*es.unphi+real(down(es.Ez))*es.unz)				
es.intWe	es.int_We(es.d*es.dWe)	J	Total electric energy	Global	+ operation
es.zref	50[ohm]	Ω	Reference impedance	Global	

2.5.3 Charge Conservation 1

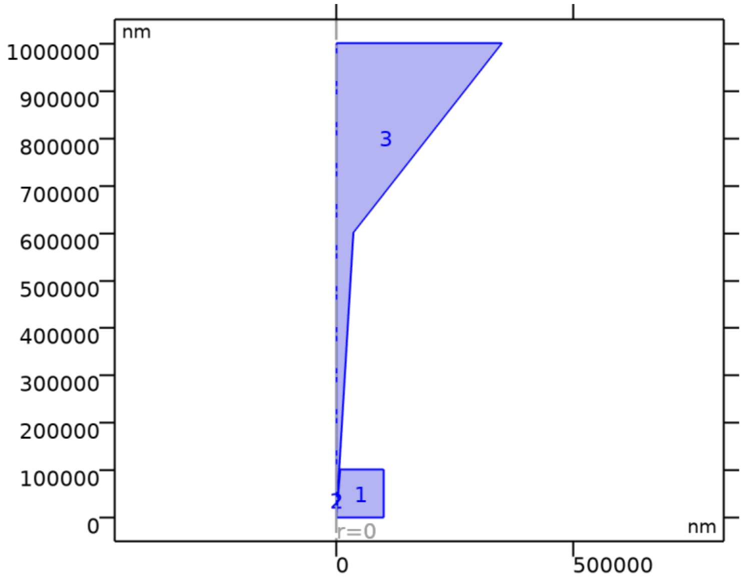

Charge Conservation 1

SELECTION

Geometric entity level	Domain
Selection	Geometry geom1: Dimension 2: All domains

EQUATIONS

$$\mathbf{E} = -\nabla V$$
$$\nabla \cdot (\epsilon_0 \epsilon_r \mathbf{E}) = \rho_v$$

2.5.3.1 Constitutive Relation D-E

SETTINGS

Description	Value
Dielectric model	Relative permittivity

Description	Value
Relative permittivity	From material

2.5.3.2 Coordinate System Selection

SETTINGS

Description	Value
Coordinate system	Global coordinate system

2.5.3.3 Model Input

SETTINGS

Description	Value
Temperature	User defined
Temperature	293.15[K]

PROPERTIES FROM MATERIAL

Property	Material	Property group
Relative permittivity	Water	Basic

2.5.3.4 Variables

Name	Expression	Unit	Description	Selection	Details
es.nD	0	C/m^2	Surface charge density	Boundaries 1–48	+ operation
es.epsilonrrr	material.epsilonr11	1	Relative permittivity, rr component	Domains 1–3	Meta
es.epsilonrphir	material.epsilonr21	1	Relative permittivity, phir component	Domains 1–3	Meta
es.epsilonrzr	material.epsilonr31	1	Relative permittivity, zr component	Domains 1–3	Meta
es.epsilonrrphi	material.epsilonr12	1	Relative permittivity, rphi component	Domains 1–3	Meta
es.epsilonrphiphi	material.epsilonr22	1	Relative permittivity, phiphi component	Domains 1–3	Meta
es.epsilonrzphi	material.epsilonr32	1	Relative permittivity, zphi component	Domains 1–3	Meta

Name	Expression	Unit	Description	Selection	Details
es.epsilonrrz	material.epsilonr13	1	Relative permittivity, rz component	Domains 1–3	Meta
es.epsilonrphiz	material.epsilonr23	1	Relative permittivity, phiz component	Domains 1–3	Meta
es.epsilonrzz	material.epsilonr33	1	Relative permittivity, zz component	Domains 1–3	Meta
es.epsilonr_iso	material.epsilonr_iso	1	Relative permittivity, isotropic value	Domains 1–3	Meta
es.Drr	0	C/m²	Remanent electric displacement, r component	Domains 1–3	
es.Drphi	0	C/m²	Remanent electric displacement, phi component	Domains 1–3	
es.Drz	0	C/m²	Remanent electric displacement, z component	Domains 1–3	
es.Dr	epsilon0_const*es.I_srr*es.Er+epsilon0_const*es.I_srphi*es.Ephi+epsilon0_const*es.I_srz*es.Ez+es.Pr+es.Per	C/m²	Electric displacement field, r component	Domains 1–3	
es.Dphi	epsilon0_const*es.I_sphir*es.Er+epsilon0_const*es.I_sphiphi*es.Ephi+epsilon0_const*es.I_sphiz*es.Ez+es.Pphi+es.Pephi	C/m²	Electric displacement field, phi component	Domains 1–3	
es.Dz	epsilon0_const*es.I_szr*es.Er+epsilon0_const*es.I_szphi*es.Ephi+epsilon0_const*es.I_szz*es.Ez+es.Pz+es.Pez	C/m²	Electric displacement field, z component	Domains 1–3	

Name	Expression	Unit	Description	Selection	Details
es.Pr	epsilon0_const*(es.chirr*es.Er+es.chirphi*es.Ephi+es.chirz*es.Ez)	C/m²	Polarization, r component	Domains 1–3	
es.Pphi	epsilon0_const*(es.chiphir*es.Er+es.chiphiphi*es.Ephi+es.chiphiz*es.Ez)	C/m²	Polarization, phi component	Domains 1–3	
es.Pz	epsilon0_const*(es.chizr*es.Er+es.chizphi*es.Ephi+es.chizz*es.Ez)	C/m²	Polarization, z component	Domains 1–3	
es.normD	sqrt(realdot(es.Dr,es.Dr)+realdot(es.Dphi,es.Dphi)+realdot(es.Dz,es.Dz))	C/m²	Electric displacement field norm	Domains 1–3	
es.normP	sqrt(realdot(es.Pr,es.Pr)+realdot(es.Pphi,es.Pphi)+realdot(es.Pz,es.Pz))	C/m²	Polarization norm	Domains 1–3	
es.Per	0	C/m²	Polarization contribution, r component	Domains 1–3	+ operation
es.Pephi	0	C/m²	Polarization contribution, phi component	Domains 1–3	+ operation
es.Pez	0	C/m²	Polarization contribution, z component	Domains 1–3	+ operation
es.chirr	-1+es.epsilonrrr	1	Electric susceptibility, rr component	Domains 1–3	
es.chiphir	es.epsilonrphir	1	Electric susceptibility, phir component	Domains 1–3	
es.chizr	es.epsilonrzr	1	Electric susceptibility, zr component	Domains 1–3	
es.chirphi	es.epsilonrrphi	1	Electric susceptibility, rphi component	Domains 1–3	

Name	Expression	Unit	Description	Selection	Details
es.chiphiphi	-1+es.epsilonrphiphi	1	Electric susceptibility, phiphi component	Domains 1–3	
es.chizphi	es.epsilonrzphi	1	Electric susceptibility, zphi component	Domains 1–3	
es.chirz	es.epsilonrrz	1	Electric susceptibility, rz component	Domains 1–3	
es.chiphiz	es.epsilonrphiz	1	Electric susceptibility, phiz component	Domains 1–3	
es.chizz	-1+es.epsilonrzz	1	Electric susceptibility, zz component	Domains 1–3	
es.Er	-Vr	V/m	Electric field, r component	Domains 1–3	
es.Ephi	0	V/m	Electric field, phi component	Domains 1–3	
es.Ez	-Vz	V/m	Electric field, z component	Domains 1–3	
es.tEr	-VTr	V/m	Tangential electric field, r component	Boundaries 1–48	
es.tEphi	0	V/m	Tangential electric field, phi component	Boundaries 1–48	
es.tEz	-VTz	V/m	Tangential electric field, z component	Boundaries 1–48	
es.normE	sqrt(realdot(es.Er,es.Er)+realdot(es.Ephi,es.Ephi)+realdot(es.Ez,es.Ez))	V/m	Electric field norm	Domains 1–3	
es.Jr	2*es.Jdr	A/m²	Current density, r component	Domains 1–3	+ operation
es.Jphi	2*es.Jdphi	A/m²	Current density, phi component	Domains 1–3	+ operation
es.Jz	2*es.Jdz	A/m²	Current density, z component	Domains 1–3	+ operation

Name	Expression	Unit	Description	Selection	Details
es.Jdr	0	A/m²	Displacement current density, r component	Domains 1–3	
es.Jdphi	0	A/m²	Displacement current density, phi component	Domains 1–3	
es.Jdz	0	A/m²	Displacement current density, z component	Domains 1–3	
es.normJ	sqrt(realdot(es.Jr,es.Jr)+realdot(es.Jphi,es.Jphi)+realdot(es.Jz,es.Jz))	A/m²	Current density norm	Domains 1–3	
es.ccn1.nJ	es.unr*down(es.Jr)+es.unphi*down(es.Jphi)+es.unz*down(es.Jz)	A/m²	Inward current density	Boundaries 1–4, 6–13, 15–48	
es.W	es.We	J/m³	Energy density	Domains 1–3	+ operation
es.dWe	2*es.We*pi*r	J/m²	Integrand for total electric energy	Domains 1–3	Meta
es.We	0.5*epsilon0_const*(((es.I_srr+es.chirr)*es.Er+(es.I_srphi+es.chirphi)*es.Ephi+(es.I_srz+es.chirz)*es.Ez)*es.Er+((es.I_sphir+es.chiphir)*es.Er+(es.I_sphiphi+es.chiphiphi)*es.Ephi+(es.I_sphiz+es.chiphiz)*es.Ez)*es.Ephi+((es.I_szr+es.chizr)*es.Er+(es.I_szphi+es.chizphi)*es.Ephi+(es.I_szz+es.chizz)*es.Ez)*es.Ez)	J/m³	Electric energy density	Domains 1–3	

2.5.3.5 Shape functions

Name	Shape function	Unit	Description	Shape frame	Selection
V	Lagrange (Quadratic)	V	Electric potential	Material	Domains 1–3

2.5.3.6 Weak Expressions

Weak expression	Integration order	Integration frame	Selection
-2*(es.Dr*test(Vr)+es.Dz*test(Vz))*es.d*pi*r	4	Material	Domains 1–3

2.5.4 Axial Symmetry 1

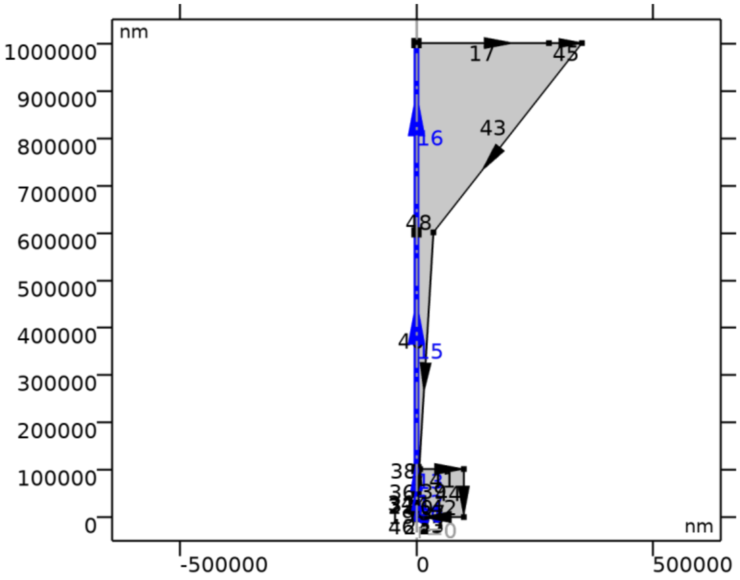

Axial Symmetry 1

SELECTION

Geometric entity level	Boundary
Selection	Geometry geom1: Dimension 1: All boundaries

2.5.5 Zero Charge 1

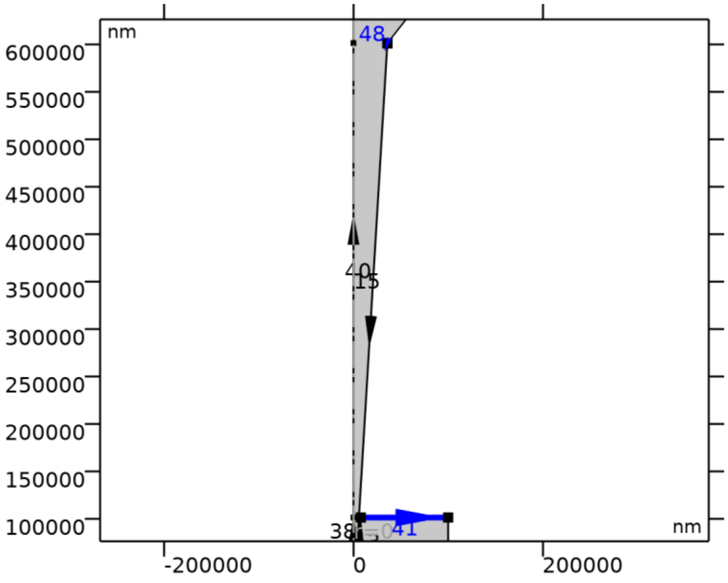

Zero Charge 1

SELECTION

Geometric entity level	Boundary
Selection	Geometry geom1: Dimension 1: All boundaries

EQUATIONS

$$\mathbf{n}\cdot\mathbf{D}=0$$

2.5.5.1 Variables

Name	Expression	Unit	Description	Selection	Details
es.nD	0	C/m^2	Surface charge density	Boundaries 41, 48	+ operation

2.5.5.2 Shape functions

Name	Shape function	Unit	Description	Shape frame	Selection	Details
V	Lagrange (Quadratic)	V	Electric potential	Material	No boundaries	Slit

2.5.6 Initial Values 1

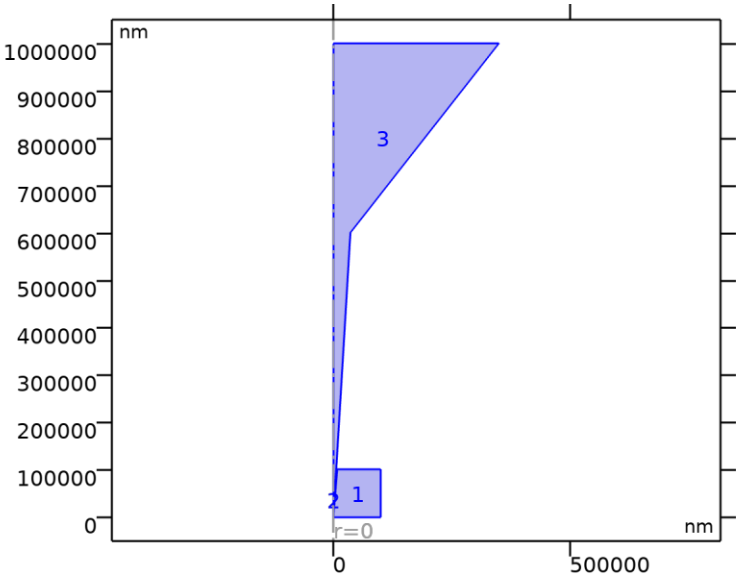

Initial Values 1

SELECTION

Geometric entity level	Domain
Selection	Geometry geom1: Dimension 2: All domains

SETTINGS

Description	Value
Electric potential	0

2.5.7 Ground (Tip QCRE)

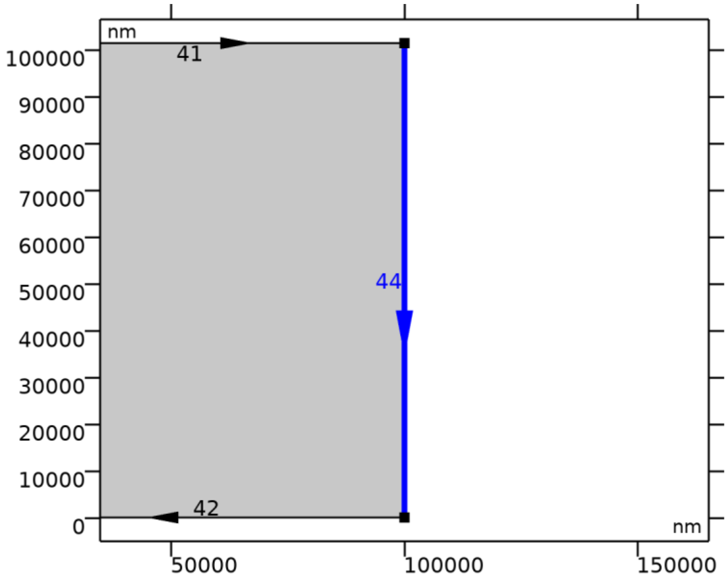

Ground (Tip QCRE)

SELECTION

Geometric entity level	Boundary
Selection	Geometry geom1: Dimension 1: Boundary 44

EQUATIONS

$$V=0$$

2.5.7.1 Constraint Settings

SETTINGS

Description	Value
Apply reaction terms on	All physics (symmetric)
Use weak constraints	Off
Constraint method	Elemental

2.5.7.2 Variables

Name	Expression	Unit	Description	Selection	Details
es.nD	es.unr*down(es.Dr)+es.unphi*down(es.Dphi)+es.unz*down(es.Dz)	C/m^2	Surface charge density	Boundary 44	+ operation

2.5.7.3 Constraints

Constraint	Constraint force	Shape function	Selection	Details
-V	test(-V)	Lagrange (Quadratic)	Boundary 44	Elemental

2.5.8 Electric Potential (Tip QRCE) VPulse

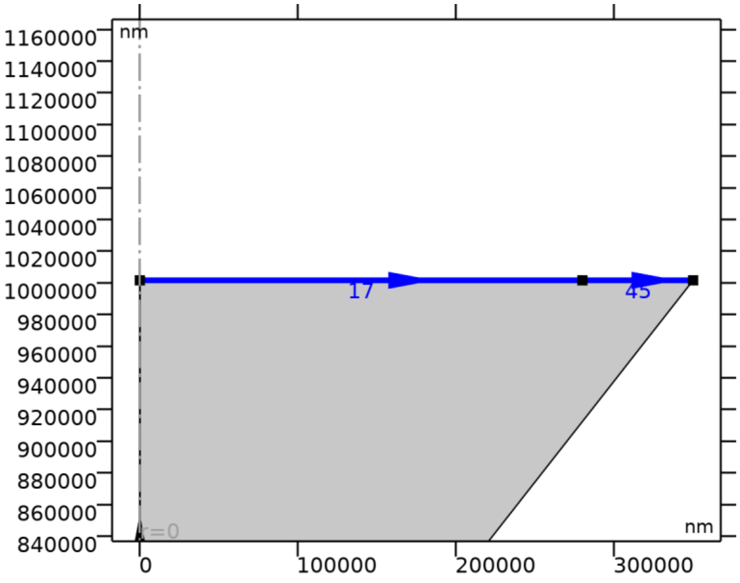

Electric Potential (Tip QRCE) VPulse

SELECTION

Geometric entity level	Boundary
Selection	Geometry geom1: Dimension 1: Boundaries 17, 45

EQUATIONS

$$V = V_0$$

2.5.8.1 Electric Potential

SETTINGS

Description	Value
Electric potential	VPulse

2.5.8.2 Constraint Settings

SETTINGS

Description	Value
Apply reaction terms on	All physics (symmetric)
Use weak constraints	Off
Constraint method	Elemental

2.5.8.3 Variables

Name	Expression	Unit	Description	Selection	Details
es.nD	es.unr*down(es.Dr)+es.unphi*down(es.Dphi)+es.unz*down(es.Dz)	C/m²	Surface charge density	Boundaries 17, 45	+ operation
es.V0	VPulse	V	Electric potential	Boundaries 17, 45	

2.5.8.4 Constraints

Constraint	Constraint force	Shape function	Selection	Details
es.V0-V	test(es.V0-V)	Lagrange (Quadratic)	Boundaries 17, 45	Elemental

2.5.9 Surface Charge - Pipette Wall

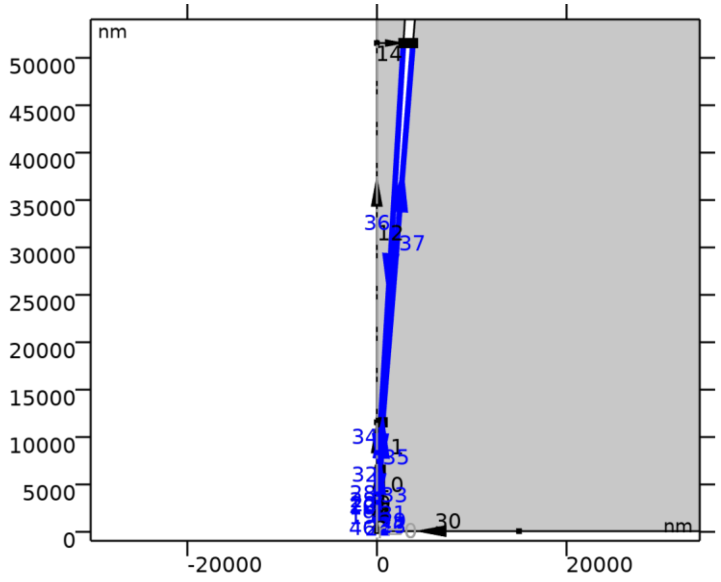

Surface Charge - Pipette Wall

SELECTION

Geometric entity level	Boundary
Selection	Geometry geom1: Dimension 1: Boundaries 18–29, 31–37, 46–47

EQUATIONS

$$\mathbf{n} \cdot (\mathbf{D}_1 - \mathbf{D}_2) = \rho_s$$

2.5.9.1 Surface Charge Density

SETTINGS

Description	Value
Surface charge density	PoreSurfaceCharge

2.5.9.2 Coordinate System Selection

SETTINGS

Description	Value
Coordinate system	Global coordinate system

2.5.9.3 Variables

Name	Expression	Unit	Description	Selection	Details
es.nD	es.sfcd1.rhoqs	C/m^2	Surface charge density	Boundaries 18–29, 31–37, 46–47	+ operation
es.sfcd1.rhoqs	PoreSurfaceCharge	C/m^2	Surface charge density	Boundaries 18–29, 31–37, 46–47	

2.5.9.4 Weak Expressions

Weak expression	Integration order	Integration frame	Selection
-2*es.sfcd1.rhoqs*test(V)*es.d*pi*r	4	Material	Boundaries 18–29, 31–37, 46–47

2.5.10 Surface Charge - Taper Wall

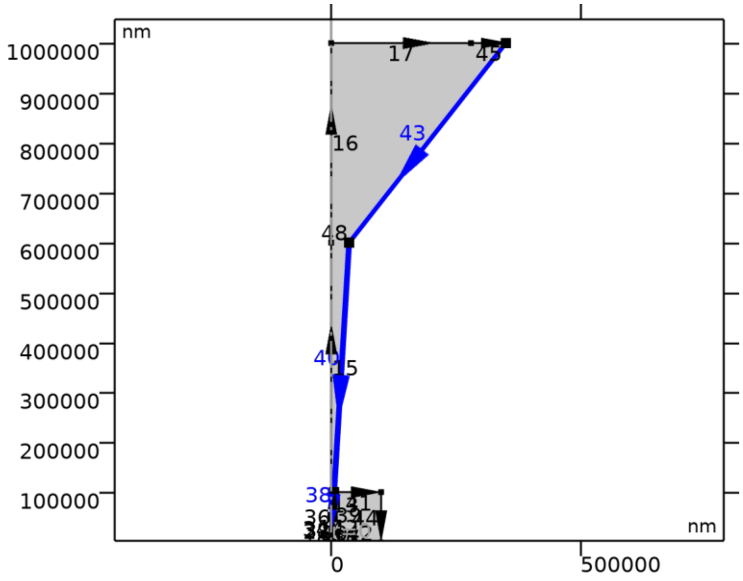

Surface Charge - Taper Wall

SELECTION

Geometric entity level	Boundary
Selection	Geometry geom1: Dimension 1: Boundaries 38, 40, 43

EQUATIONS

$$\mathbf{n}\cdot(\mathbf{D}_1 - \mathbf{D}_2) = \rho_s$$

2.5.10.1 Surface Charge Density

SETTINGS

Description	Value
Surface charge density	PoreSurfaceCharge*step2(z[1/m])

2.5.10.2 Coordinate System Selection

SETTINGS

Description	Value
Coordinate system	Global coordinate system

2.5.10.3 Variables

Name	Expression	Unit	Description	Selection	Details
es.nD	es.sfcd4.rhoqs	C/m²	Surface charge density	Boundaries 38, 40, 43	+ operation
es.sfcd4.rhoqs	PoreSurfaceCharge*step2(z[1/m])	C/m²	Surface charge density	Boundaries 38, 40, 43	

2.5.10.4 Weak Expressions

Weak expression	Integration order	Integration frame	Selection
-2*es.sfcd4.rhoqs*test(V)*es.d*pi*r	4	Material	Boundaries 38, 40, 43

2.5.11 Surface Charge - Outer Wall

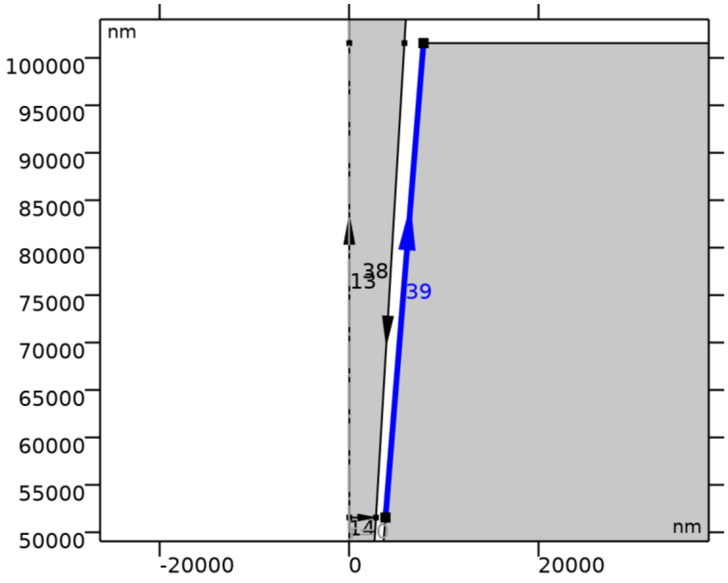

Surface Charge - Outer Wall

SELECTION

Geometric entity level	Boundary
Name	Pipette 100000nm Outer (Part Instance 1)
Selection	Named geom1_pi1_boxsel30: Geometry geom1: Dimension 1: Boundary 39

EQUATIONS

$$\mathbf{n}\cdot(\mathbf{D}_1 - \mathbf{D}_2) = \rho_s$$

2.5.11.1 Surface Charge Density

SETTINGS

Description	Value
Surface charge density	PoreSurfaceCharge*step1(z[1/m])

2.5.11.2 Coordinate System Selection

SETTINGS

Description	Value
Coordinate system	Global coordinate system

2.5.11.3 Variables

Name	Expression	Unit	Description	Selection	Details
es.nD	es.sfcd3.rhoqs	C/m^2	Surface charge density	Boundary 39	+ operation

Name	Expression	Unit	Description	Selection	Details
es.sfcd3.rhoqs	PoreSurfaceCharge*step1(z[1/m])	C/m²	Surface charge density	Boundary 39	

2.5.11.4 Weak Expressions

Weak expression	Integration order	Integration frame	Selection
-2*es.sfcd3.rhoqs*test(V)*es.d*pi*r	4	Material	Boundary 39

2.5.12 Surface Charge - Substrate 1

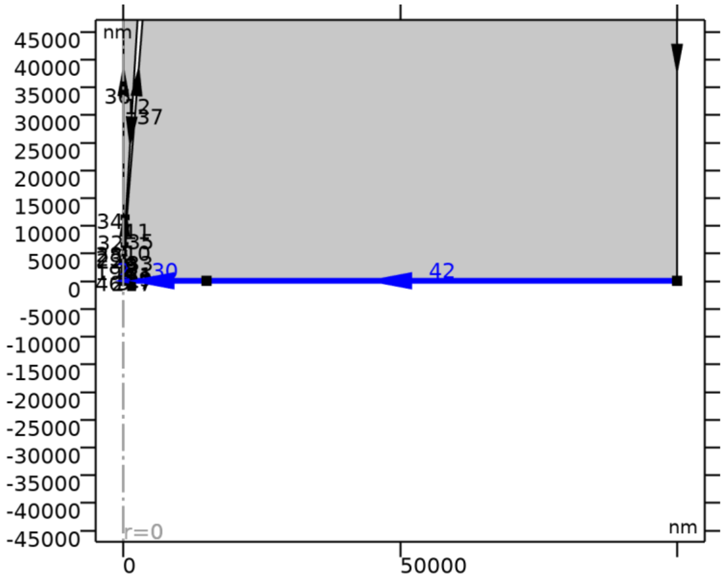

Surface Charge - Substrate 1

SELECTION

Geometric entity level	Boundary
Selection	Geometry geom1: Dimension 1: Boundaries 2, 30, 42

EQUATIONS

$$\mathbf{n} \cdot (\mathbf{D}_1 - \mathbf{D}_2) = \rho_s$$

2.5.12.1 Surface Charge Density

SETTINGS

Description	Value
Surface charge density	SigmaSubstrate*(step3(r/1[m]))

2.5.12.2 Coordinate System Selection

SETTINGS

Description	Value
Coordinate system	Global coordinate system

2.5.12.3 Variables

Name	Expression	Unit	Description	Selection	Details
es.nD	es.sfcd5.rhoqs	C/m²	Surface charge density	Boundaries 2, 30, 42	+ operation
es.sfcd5.rhoqs	SigmaSubstrate*step 3(r/1[m])	C/m²	Surface charge density	Boundaries 2, 30, 42	

2.5.12.4 Weak Expressions

Weak expression	Integration order	Integration frame	Selection
-2*es.sfcd5.rhoqs*test(V)*es.d*pi*r	4	Material	Boundaries 2, 30, 42

2.5.13 Space Charge Density 1

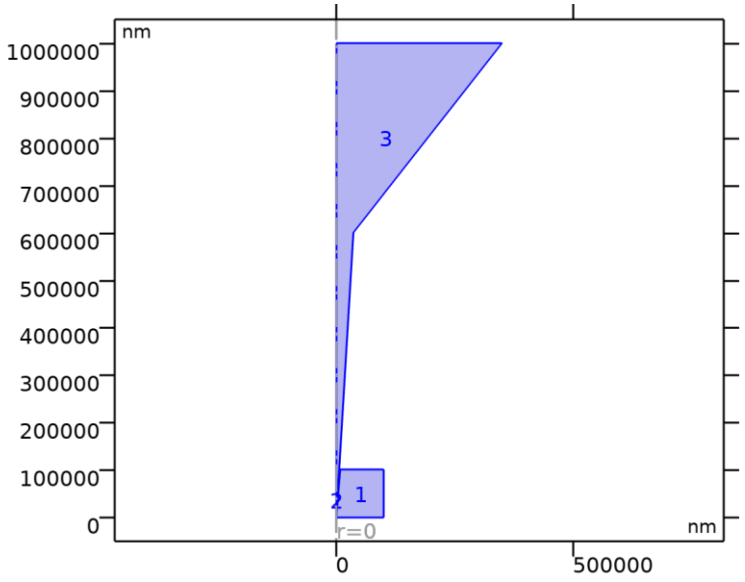

Space Charge Density 1

SELECTION

Geometric entity level	Domain
Selection	Geometry geom1: Dimension 2: All domains

EQUATIONS

$$\nabla \cdot \mathbf{D} = \rho_V$$

2.5.13.1 Coordinate System Selection

SETTINGS

Description	Value
Coordinate system	Global coordinate system

2.5.13.2 Variables

Name	Expression	Unit	Description	Selection	Details
es.scd1.rhoq	F_const*(cK-cNO3-cCl)	C/m³	Space charge density	Domains 1–3	
es.rhoq	es.scd1.rhoq	C/m³	Space charge density	Domains 1–3	+ operation

2.5.13.3 Weak Expressions

Weak expression	Integration order	Integration frame	Selection
-2*es.scd1.rhoq*test(V)*es.d*pi*r	4	Material	Domains 1–3

2.6 LAMINAR FLOW 1

USED PRODUCTS

COMSOL Multiphysics

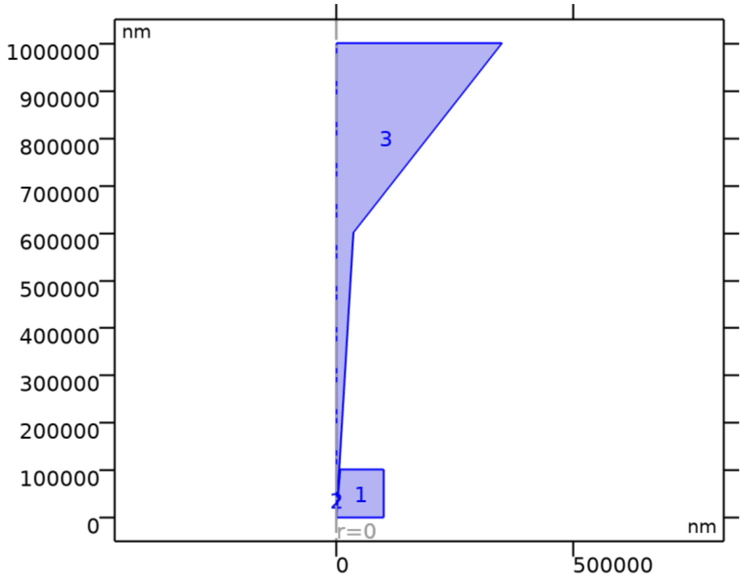

Laminar Flow 1

SELECTION

Geometric entity level	Domain
Selection	Geometry geom1: Dimension 2: All domains

EQUATIONS

$$\rho(\mathbf{u2}\cdot\nabla)\mathbf{u2} = \nabla\cdot[-p2\mathbf{I}+\mathbf{K}]+\mathbf{F}$$
$$\rho\nabla\cdot\mathbf{u2} = 0$$

2.6.1 Interface Settings

2.6.1.1 Discretization

SETTINGS

Description	Value
Discretization of fluids	P1 + P1

SETTINGS

Description	Value
Equation form	Study controlled

2.6.1.2 Physical Model

SETTINGS

Description	Value
Neglect inertial term (Stokes flow)	Off
Compressibility	Incompressible flow
Enable porous media domains	Off
Include gravity	Off
Reference temperature	T
Reference pressure level	1[atm]
Reference temperature	User defined

2.6.1.3 Turbulence

SETTINGS

Description	Value
Turbulence model type	None

2.6.1.4 Advanced Settings

SETTINGS

Description	Value
Use pseudo time stepping for stationary equation form	Off
Use Block Navier-Stokes preconditioner in time dependent studies	Off

2.6.2 Variables

Name	Expression	Unit	Description	Selection	Details
spf2.Tref	model.input.Tref	K	Reference temperature	Global	Meta
spf2.dz	1	m	Thickness	Domains 1–3	
spf2.pref	1[atm]	Pa	Reference pressure level	Domains 1–3	
spf2.pA	p2+spf2.pref	Pa	Absolute pressure	Domains 1–3	
spf2.hasWF	0		Help variable	Boundaries 1–4, 6–13, 15–48	
spf2.hasWF_u	0		Help variable	Boundaries 5, 14	
spf2.hasWF_d	0		Help variable	Boundaries 5, 14	
spf2.dt_CFL	1/max(spf2.maxop(sqrt(emetric(u2,w2))),eps)	s	Time step, CFL=1	Global	
spf2.Qvd_tot	spf2.intop(2*spf2.Qvd*pi*r)	W	Total viscous dissipation	Global	
spf2.K_stressr	spf2.K_stress_tensorrr*spf2.nrmesh+spf2.K_stress_tensorrphi*spf2.nphimesh+spf2.K_stress_tensorrz*spf2.nzmesh	N/m²	Viscous force, exterior boundaries, r component	Boundaries 1–4, 6–13, 15–48	
spf2.K_stressphi	spf2.K_stress_tensorphir*spf2.nrmesh+spf2.K_stress_tensorphiphi*spf2.nphimesh+spf2.K_stress_tensorphiz*spf2.nzmesh	N/m²	Viscous force, exterior boundaries, phi component	Boundaries 1–4, 6–13, 15–48	
spf2.K_stressz	spf2.K_stress_tensorzr*spf2.nrmesh+spf2.K_stress_tensorzphi*spf2.nphimesh+spf2.K_stress_tensorzz*spf2.nzmesh	N/m²	Viscous force, exterior boundaries, z component	Boundaries 1–4, 6–13, 15–48	
spf2.T_stressr	spf2.T_stress_tensorrr*spf2.nrmesh+spf2.T_stress_tensorrphi*spf2.nphimesh+spf2.T_st	N/m²	Total traction, exterior boundaries, r component	Boundaries 1–4, 6–13, 15–48	

Name	Expression	Unit	Description	Selection	Details
	ress_tensorrz*spf2.nzmesh				
spf2.T_stressphi	spf2.T_stress_tensorphir*spf2.nrmesh+spf2.T_stress_tensorphiphi*spf2.nphimesh+spf2.T_stress_tensorphiz*spf2.nzmesh	N/m²	Total traction, exterior boundaries, phi component	Boundaries 1–4, 6–13, 15–48	
spf2.T_stressz	spf2.T_stress_tensorzr*spf2.nrmesh+spf2.T_stress_tensorzphi*spf2.nphimesh+spf2.T_stress_tensorzz*spf2.nzmesh	N/m²	Total traction, exterior boundaries, z component	Boundaries 1–4, 6–13, 15–48	
spf2.K_stress_dr	down(spf2.K_stress_tensorrr)*spf2.nrmesh+down(spf2.K_stress_tensorrphi)*spf2.nphimesh+down(spf2.K_stress_tensorrz)*spf2.nzmesh	N/m²	Viscous force, interior boundaries, downside, r component	Boundaries 5, 14	
spf2.K_stress_dphi	down(spf2.K_stress_tensorphir)*spf2.nrmesh+down(spf2.K_stress_tensorphiphi)*spf2.nphimesh+down(spf2.K_stress_tensorphiz)*spf2.nzmesh	N/m²	Viscous force, interior boundaries, downside, phi component	Boundaries 5, 14	
spf2.K_stress_dz	down(spf2.K_stress_tensorzr)*spf2.nrmesh+down(spf2.K_stress_tensorzphi)*spf2.nphimesh+down(spf2.K_stress_tensorzz)*spf2.nzmesh	N/m²	Viscous force, interior boundaries, downside, z component	Boundaries 5, 14	
spf2.K_stress_dr	down(spf2.K_stress_tensorrr)*spf2.dnrmesh+down(spf2.K_stress_tensorrphi)*spf2.dnphimesh+down(spf2.K_stress_tensorrz)*spf2.dnzmesh	N/m²	Viscous force, interior boundaries, downside, r component	Boundaries 1–4, 6–13, 15–48	
spf2.K_stress_dphi	down(spf2.K_stress_tensorphir)*spf2.dnrmesh+down(spf2.K_stres	N/m²	Viscous force, interior boundaries,	Boundaries 1–4, 6–13, 15–48	

Name	Expression	Unit	Description	Selection	Details
	s_tensorphiphi)*spf2.dnphimesh+down(spf2.K_stress_tensorphiz)*spf2.dnzmesh		downside, phi component		
spf2.K_stress_dz	down(spf2.K_stress_tensorzr)*spf2.dnrmesh+down(spf2.K_stress_tensorzphi)*spf2.dnphimesh+down(spf2.K_stress_tensorzz)*spf2.dnzmesh	N/m²	Viscous force, interior boundaries, downside, z component	Boundaries 1–4, 6–13, 15–48	
spf2.K_stress_ur	-up(spf2.K_stress_tensorrr)*spf2.nrmesh-up(spf2.K_stress_tensorrphi)*spf2.nphimesh-up(spf2.K_stress_tensorrz)*spf2.nzmesh	N/m²	Viscous force, interior boundaries, upside, r component	Boundaries 5, 14	
spf2.K_stress_uphi	-up(spf2.K_stress_tensorphir)*spf2.nrmesh-up(spf2.K_stress_tensorphiphi)*spf2.nphimesh-up(spf2.K_stress_tensorphiz)*spf2.nzmesh	N/m²	Viscous force, interior boundaries, upside, phi component	Boundaries 5, 14	
spf2.K_stress_uz	-up(spf2.K_stress_tensorzr)*spf2.nrmesh-up(spf2.K_stress_tensorzphi)*spf2.nphimesh-up(spf2.K_stress_tensorzz)*spf2.nzmesh	N/m²	Viscous force, interior boundaries, upside, z component	Boundaries 5, 14	
spf2.T_stress_dr	down(spf2.T_stress_tensorrr)*spf2.nrmesh+down(spf2.T_stress_tensorrphi)*spf2.nphimesh+down(spf2.T_stress_tensorrz)*spf2.nzmesh	N/m²	Total traction, interior boundaries, downside, r component	Boundaries 5, 14	
spf2.T_stress_dphi	down(spf2.T_stress_tensorphir)*spf2.nrmesh+down(spf2.T_stress	N/m²	Total traction, interior boundaries,	Boundaries 5, 14	

Name	Expression	Unit	Description	Selection	Details
	_tensorphiphi)*spf2.nphimesh+down(spf2.T_stress_tensorphiz)*spf2.nzmesh		downside, phi component		
spf2.T_stress_dz	down(spf2.T_stress_tensorzr)*spf2.nrmesh+down(spf2.T_stress_tensorzphi)*spf2.nphimesh+down(spf2.T_stress_tensorzz)*spf2.nzmesh	N/m²	Total traction, interior boundaries, downside, z component	Boundaries 5, 14	
spf2.T_stress_dr	down(spf2.T_stress_tensorrr)*spf2.dnrmesh+down(spf2.T_stress_tensorrphi)*spf2.dnphimesh+down(spf2.T_stress_tensorrz)*spf2.dnzmesh	N/m²	Total traction, interior boundaries, downside, r component	Boundaries 1–4, 6–13, 15–48	
spf2.T_stress_dphi	down(spf2.T_stress_tensorphir)*spf2.dnrmesh+down(spf2.T_stress_tensorphiphi)*spf2.dnphimesh+down(spf2.T_stress_tensorphiz)*spf2.dnzmesh	N/m²	Total traction, interior boundaries, downside, phi component	Boundaries 1–4, 6–13, 15–48	
spf2.T_stress_dz	down(spf2.T_stress_tensorzr)*spf2.dnrmesh+down(spf2.T_stress_tensorzphi)*spf2.dnphimesh+down(spf2.T_stress_tensorzz)*spf2.dnzmesh	N/m²	Total traction, interior boundaries, downside, z component	Boundaries 1–4, 6–13, 15–48	
spf2.T_stress_ur	-up(spf2.T_stress_tensorrr)*spf2.nrmesh-up(spf2.T_stress_tensorrphi)*spf2.nphimesh-up(spf2.T_stress_tensorrz)*spf2.nzmesh	N/m²	Total traction, interior boundaries, upside, r component	Boundaries 5, 14	
spf2.T_stress_uphi	-up(spf2.T_stress_tensorphir)*spf2.nrmesh-up(spf2.T_stress_tensorphiphi)*spf2.nphim	N/m²	Total traction, interior boundaries, upside, phi component	Boundaries 5, 14	

Name	Expression	Unit	Description	Selection	Details
	esh-up(spf2.T_stress_tensorphiz)*spf2.nzmesh				
spf2.T_stress_uz	-up(spf2.T_stress_tensorzr)*spf2.nrmesh-up(spf2.T_stress_tensorzphi)*spf2.nphimesh-up(spf2.T_stress_tensorzz)*spf2.nzmesh	N/m²	Total traction, interior boundaries, upside, z component	Boundaries 5, 14	
spf2.usePseudoTimeStepping	0	1	Help variable	Global	
spf2.nr	nr	1	Normal vector, r component	Boundaries 5, 14	
spf2.nphi	0	1	Normal vector, phi component	Boundaries 5, 14	
spf2.nz	nz	1	Normal vector, z component	Boundaries 5, 14	
spf2.nr	dnr	1	Normal vector, r component	Boundaries 1–4, 6–13, 15–48	
spf2.nphi	0	1	Normal vector, phi component	Boundaries 1–4, 6–13, 15–48	
spf2.nz	dnz	1	Normal vector, z component	Boundaries 1–4, 6–13, 15–48	
spf2.nrmesh	nrmesh	1	Normal vector, r component	Boundaries 5, 14	
spf2.nphimesh	0	1	Normal vector, phi component	Boundaries 5, 14	
spf2.nzmesh	nzmesh	1	Normal vector, z component	Boundaries 5, 14	
spf2.nrmesh	dnrmesh	1	Normal vector, r component	Boundaries 1–4, 6–13, 15–48	
spf2.nphimesh	0	1	Normal vector, phi component	Boundaries 1–4, 6–13, 15–48	

Name	Expression	Unit	Description	Selection	Details
spf2.nzmesh	dnzmesh	1	Normal vector, z component	Boundaries 1–4, 6–13, 15–48	

2.6.3 Fluid Properties 1

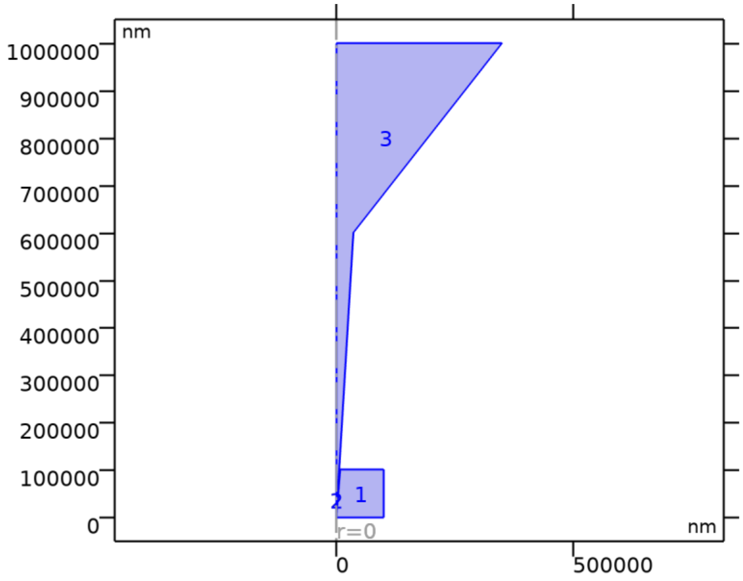

Fluid Properties 1

SELECTION

Geometric entity level	Domain
Selection	Geometry geom1: Dimension 2: All domains

EQUATIONS

$$\rho(\mathbf{u2}\cdot\nabla)\mathbf{u2}=\nabla\cdot[-p2\mathbf{I}+\mathbf{K}]+\mathbf{F}$$
$$\rho\nabla\cdot\mathbf{u2}=0$$
$$\mathbf{K}=\mu\left(\nabla\mathbf{u2}+(\nabla\mathbf{u2})^T\right)$$

2.6.3.1 Fluid Properties

SETTINGS

Description	Value
Density	From material
	Newtonian
Dynamic viscosity	From material

2.6.3.2 Model Input

SETTINGS

Description	Value
Temperature	User defined
Temperature	T

PROPERTIES FROM MATERIAL

Property	Material	Property group
Density	Water	Basic
Dynamic viscosity	Water	Basic

2.6.3.3 Variables

Name	Expression	Unit	Description	Selection	Details
spf2.Fr	0	N/m³	Volume force, r component	Domains 1–3	+ operation
spf2.Fphi	0	N/m³	Volume force, phi component	Domains 1–3	+ operation
spf2.Fz	0	N/m³	Volume force, z component	Domains 1–3	+ operation
spf2.mu	material.mu	Pa·s	Dynamic viscosity	Domains 1–3	Meta
spf2.rho	subst(material.rho,spf2.fp1.minput_temperature,spf2.Trho,spf2.fp1.minput_pressure,spf2.prho)	kg/m³	Density	Domains 1–3	Meta
spf2.Trho	spf2.Tref	K	Temperature for density evaluation	Domains 1–3	
spf2.prho	spf2.pref	Pa	Pressure for the evaluation of density	Domains 1–3	
spf2.rhoref	subst(material.rho,spf2.fp1.minput_temperature,spf2.Tref,spf2.fp1.minput_pressure,spf2.pref)	kg/m³	Reference density	Domains 1–3	Meta
spf2.mumat	material.mu	Pa·s	Dynamic viscosity	Domains 1–3	Meta
spf2.srijrr	u2r	1/s	Strain rate tensor, rr component	Domains 1–3	

Name	Expression	Unit	Description	Selection	Details
spf2.srijphir	0	1/s	Strain rate tensor, phir component	Domains 1–3	
spf2.srijzr	0.5*(w2r+u2z)	1/s	Strain rate tensor, zr component	Domains 1–3	
spf2.srijrphi	0	1/s	Strain rate tensor, rphi component	Domains 1–3	
spf2.srijphiphi	if(abs(r)<0.001*h, u2r,u2/r)	1/s	Strain rate tensor, phiphi component	Domains 1–3	
spf2.srijzphi	0	1/s	Strain rate tensor, zphi component	Domains 1–3	
spf2.srijrz	0.5*(u2z+w2r)	1/s	Strain rate tensor, rz component	Domains 1–3	
spf2.srijphiz	0	1/s	Strain rate tensor, phiz component	Domains 1–3	
spf2.srijzz	w2z	1/s	Strain rate tensor, zz component	Domains 1–3	
spf2.rrijrr	0	1/s	Rotation rate tensor, rr component	Domains 1–3	
spf2.rrijphir	0	1/s	Rotation rate tensor, phir component	Domains 1–3	
spf2.rrijzr	0.5*(w2r-u2z)	1/s	Rotation rate tensor, zr component	Domains 1–3	
spf2.rrijrphi	0	1/s	Rotation rate tensor, rphi component	Domains 1–3	
spf2.rrijphiphi	0	1/s	Rotation rate tensor, phiphi component	Domains 1–3	
spf2.rrijzphi	0	1/s	Rotation rate tensor, zphi component	Domains 1–3	

Name	Expression	Unit	Description	Selection	Details
spf2.rrijrz	0.5*(u2z-w2r)	1/s	Rotation rate tensor, rz component	Domains 1–3	
spf2.rrijphiz	0	1/s	Rotation rate tensor, phiz component	Domains 1–3	
spf2.rrijzz	0	1/s	Rotation rate tensor, zz component	Domains 1–3	
spf2.sr	sqrt(2*spf2.srijrr^2+2*spf2.srijrphi^2+2*spf2.srijrz^2+2*spf2.srijphir^2+2*spf2.srijphiphi^2+2*spf2.srijphiz^2+2*spf2.srijzr^2+2*spf2.srijzphi^2+2*spf2.srijzz^2+eps)	1/s	Shear rate	Domains 1–3	
spf2.rr	sqrt(2*spf2.rrijrr^2+2*spf2.rrijrphi^2+2*spf2.rrijrz^2+2*spf2.rrijphir^2+2*spf2.rrijphiphi^2+2*spf2.rrijphiz^2+2*spf2.rrijzr^2+2*spf2.rrijzphi^2+2*spf2.rrijzz^2+eps)	1/s	Rotation rate	Domains 1–3	
spf2.divu	u2r+if(abs(r)<0.001*h,u2r,u2/r)+w2z	1/s	Divergence of velocity field	Domains 1–3	
spf2.U	sqrt(u2^2+w2^2)	m/s	Velocity magnitude	Domains 1–3	
spf2.vorticityr	0	1/s	Vorticity field, r component	Domains 1–3	
spf2.vorticityphi	-w2r+u2z	1/s	Vorticity field, phi component	Domains 1–3	
spf2.vorticityz	0	1/s	Vorticity field, z component	Domains 1–3	
spf2.vort_magn	sqrt(spf2.vorticityr^2+spf2.vorticityphi^2+spf2.vorticityz^2)	1/s	Vorticity magnitude	Domains 1–3	

Name	Expression	Unit	Description	Selection	Details
spf2.cellRe	0.25*spf2.rho*sqrt(emetric(u2,w2)/emetric2)/spf2.mu	1	Cell Reynolds number	Domains 1–3	
spf2.nu	spf2.mu/spf2.rho	m²/s	Kinematic viscosity	Domains 1–3	
spf2.betaT	0	1/Pa	Isothermal compressibility coefficient	Domains 1–3	
spf2.Qm	0	kg/(m³·s)	Source term	Domains 1–3	+ operation
spf2.Fgtotr	0	N/m³	Gravity force, r component	Domains 1–3	+ operation
spf2.Fgtotphi	0	N/m³	Gravity force, phi component	Domains 1–3	+ operation
spf2.Fgtotz	0	N/m³	Gravity force, z component	Domains 1–3	+ operation
spf2.mu_eff	spf2.mu+spf2.muT	Pa·s	Effective dynamic viscosity	Domains 1–3	
spf2.muT	0	Pa·s	Turbulent dynamic viscosity	Domains 1–3	
spf2.T_stress_tensorrr	spf2.K_stress_tensorrr-p2	N/m²	Total stress tensor, rr component	Domains 1–3	+ operation
spf2.T_stress_tensorphir	spf2.K_stress_tensorphir	N/m²	Total stress tensor, phir component	Domains 1–3	+ operation
spf2.T_stress_tensorzr	spf2.K_stress_tensorzr	N/m²	Total stress tensor, zr component	Domains 1–3	+ operation
spf2.T_stress_tensorrphi	spf2.K_stress_tensorrphi	N/m²	Total stress tensor, rphi component	Domains 1–3	+ operation
spf2.T_stress_tensorphiphi	spf2.K_stress_tensorphiphi-p2	N/m²	Total stress tensor, phiphi component	Domains 1–3	+ operation
spf2.T_stress_tensorzphi	spf2.K_stress_tensorzphi	N/m²	Total stress tensor, zphi component	Domains 1–3	+ operation

Name	Expression	Unit	Description	Selection	Details
spf2.T_stress_tensorrz	spf2.K_stress_tensorrz	N/m²	Total stress tensor, rz component	Domains 1–3	+ operation
spf2.T_stress_tensorphiz	spf2.K_stress_tensorphiz	N/m²	Total stress tensor, phiz component	Domains 1–3	+ operation
spf2.T_stress_tensorzz	spf2.K_stress_tensorzz-p2	N/m²	Total stress tensor, zz component	Domains 1–3	+ operation
spf2.K_stress_tensorrr	2*spf2.mu_eff*u2r	N/m²	Viscous stress tensor, rr component	Domains 1–3	+ operation
spf2.K_stress_tensorphir	0	N/m²	Viscous stress tensor, phir component	Domains 1–3	+ operation
spf2.K_stress_tensorzr	spf2.mu_eff*(w2r+u2z)	N/m²	Viscous stress tensor, zr component	Domains 1–3	+ operation
spf2.K_stress_tensorrphi	0	N/m²	Viscous stress tensor, rphi component	Domains 1–3	+ operation
spf2.K_stress_tensorphiphi	2*spf2.mu_eff*if(abs(r)<0.001*h,u2r,u2/r)	N/m²	Viscous stress tensor, phiphi component	Domains 1–3	+ operation
spf2.K_stress_tensorzphi	0	N/m²	Viscous stress tensor, zphi component	Domains 1–3	+ operation
spf2.K_stress_tensorrz	spf2.mu_eff*(u2z+w2r)	N/m²	Viscous stress tensor, rz component	Domains 1–3	+ operation
spf2.K_stress_tensorphiz	0	N/m²	Viscous stress tensor, phiz component	Domains 1–3	+ operation
spf2.K_stress_tensorzz	2*spf2.mu_eff*w2z	N/m²	Viscous stress tensor, zz component	Domains 1–3	+ operation
spf2.K_stress_tensor_testrr	2*spf2.mu_eff*test(u2r)	N/m²	Viscous stress tensor test, rr component	Domains 1–3	+ operation
spf2.K_stress_tensor_testphir	0	N/m²	Viscous stress tensor test, phir component	Domains 1–3	+ operation

Name	Expression	Unit	Description	Selection	Details
spf2.K_stress_tensor_testzr	spf2.mu_eff*(test(w2r)+test(u2z))	N/m^2	Viscous stress tensor test, zr component	Domains 1–3	+ operation
spf2.K_stress_tensor_testrphi	0	N/m^2	Viscous stress tensor test, rphi component	Domains 1–3	+ operation
spf2.K_stress_tensor_testphiphi	2*spf2.mu_eff*if(abs(r)<0.001*h,test(u2r),test(u2)/r)	N/m^2	Viscous stress tensor test, phiphi component	Domains 1–3	+ operation
spf2.K_stress_tensor_testzphi	0	N/m^2	Viscous stress tensor test, zphi component	Domains 1–3	+ operation
spf2.K_stress_tensor_testrz	spf2.mu_eff*(test(u2z)+test(w2r))	N/m^2	Viscous stress tensor test, rz component	Domains 1–3	+ operation
spf2.K_stress_tensor_testphiz	0	N/m^2	Viscous stress tensor test, phiz component	Domains 1–3	+ operation
spf2.K_stress_tensor_testzz	2*spf2.mu_eff*test(w2z)	N/m^2	Viscous stress tensor test, zz component	Domains 1–3	+ operation
spf2.upwind_helpr	u2	m/s	Upwind term, r component	Domains 1–3	+ operation
spf2.upwind_helpphi	0	m/s	Upwind term, phi component	Domains 1–3	+ operation
spf2.upwind_helpz	w2	m/s	Upwind term, z component	Domains 1–3	+ operation
spf2.continuityEquation	spf2.rho*spf2.divu	$kg/(m^3\cdot s)$	Continuity equation	Domains 1–3	
spf2.contCoeff	spf2.rho	kg/m^3	Continuity equation	Domains 1–3	
spf2.tau_vdrr	2*spf2.mu*spf2.srijrr	Pa	Viscous stress tensor, rr component	Domains 1–3	+ operation
spf2.tau_vdphir	2*spf2.mu*spf2.srijphir	Pa	Viscous stress tensor, phir component	Domains 1–3	+ operation
spf2.tau_vdzr	2*spf2.mu*spf2.srijzr	Pa	Viscous stress tensor, zr component	Domains 1–3	+ operation

Name	Expression	Unit	Description	Selection	Details
spf2.tau_vdrphi	2*spf2.mu*spf2.srijrphi	Pa	Viscous stress tensor, rphi component	Domains 1–3	+ operation
spf2.tau_vdphiphi	2*spf2.mu*spf2.srijphiphi	Pa	Viscous stress tensor, phiphi component	Domains 1–3	+ operation
spf2.tau_vdzphi	2*spf2.mu*spf2.srijzphi	Pa	Viscous stress tensor, zphi component	Domains 1–3	+ operation
spf2.tau_vdrz	2*spf2.mu*spf2.srijrz	Pa	Viscous stress tensor, rz component	Domains 1–3	+ operation
spf2.tau_vdphiz	2*spf2.mu*spf2.srijphiz	Pa	Viscous stress tensor, phiz component	Domains 1–3	+ operation
spf2.tau_vdzz	2*spf2.mu*spf2.srijzz	Pa	Viscous stress tensor, zz component	Domains 1–3	+ operation
spf2.Qvd	spf2.tau_vdrr*u2r+spf2.tau_vdrz*u2z+spf2.tau_vdphiphi*if(abs(r)<0.001*h,u2r,u2/r)+spf2.tau_vdzr*w2r+spf2.tau_vdzz*w2z	W/m³	Viscous dissipation	Domains 1–3	+ operation
spf2.epsilon_p	1	1	Porosity	Domains 1–3	
spf2.Fst_tensorrr	0	N/m²	Surface tension force, rr component	Domains 1–3	+ operation
spf2.Fst_tensorphir	0	N/m²	Surface tension force, phir component	Domains 1–3	+ operation
spf2.Fst_tensorzr	0	N/m²	Surface tension force, zr component	Domains 1–3	+ operation
spf2.Fst_tensorrphi	0	N/m²	Surface tension force, rphi component	Domains 1–3	+ operation
spf2.Fst_tensorphiphi	0	N/m²	Surface tension force, phiphi component	Domains 1–3	+ operation

Name	Expression	Unit	Description	Selection	Details
spf2.Fst_tensorzphi	0	N/m²	Surface tension force, zphi component	Domains 1–3	+ operation
spf2.Fst_tensorrz	0	N/m²	Surface tension force, rz component	Domains 1–3	+ operation
spf2.Fst_tensorphiz	0	N/m²	Surface tension force, phiz component	Domains 1–3	+ operation
spf2.Fst_tensorzz	0	N/m²	Surface tension force, zz component	Domains 1–3	+ operation
spf2.res_u	if(spf2.isFluidHasBeenSolved==0, 0,p2r+spf2.rho*u2*u2r+spf2.rho*w2*u2z-(d(2*u2r,r)+if(abs(r)<0.001*h,d(2*u2r,r),2*u2r/r)+d(u2z+w2r,z)-2*if(abs(r)<0.001*h,u2r,u2/r)/r)*spf2.mu-spf2.Fr)	N/m³	Equation residual	Domains 1–3	
spf2.res_v	if(spf2.isFluidHasBeenSolved==0, 0,-spf2.Fphi)	N/m³	Equation residual	Domains 1–3	
spf2.res_w	if(spf2.isFluidHasBeenSolved==0, 0,spf2.rho*u2*w2r+p2z+spf2.rho*w2*w2z-(d(w2r+u2z,r)+if(abs(r)<0.001*h,d(w2r+u2z,r),(w2r+u2z)/r)+d(2*w2z,z))*spf2.mu-spf2.Fz)	N/m³	Equation residual	Domains 1–3	
spf2.res_p	spf2.rho*spf2.divu	kg/(m³·s)	Pressure equation residual	Domains 1–3	

2.6.3.4 Shape functions

Name	Shape function	Unit	Description	Shape frame	Selection
u2	Lagrange (Linear)	m/s	Velocity field, r component	Material	Domains 1–3
w2	Lagrange (Linear)	m/s	Velocity field, z component	Material	Domains 1–3
p2	Lagrange (Linear)	Pa	Pressure	Material	Domains 1–3

2.6.4 Initial Values 1

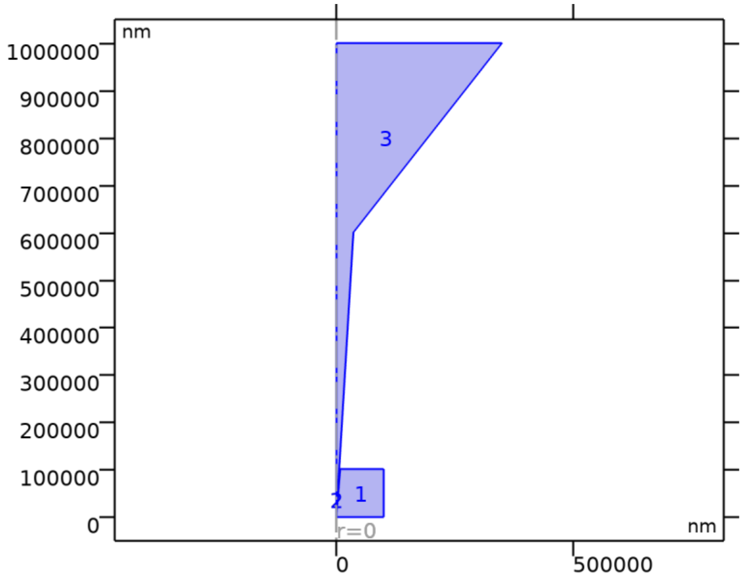

Initial Values 1

SELECTION

Geometric entity level	Domain
Selection	Geometry geom1: Dimension 2: All domains

2.6.4.1 Initial Values

SETTINGS

Description	Value
Velocity field, r component	0
Velocity field, phi component	0
Velocity field, z component	0
Pressure	0

2.6.4.2 Coordinate System Selection

SETTINGS

Description	Value
Coordinate system	Global coordinate system

2.6.4.3 Variables

Name	Expression	Unit	Description	Selection
spf2.u_initr	0	m/s	Velocity field, r component	Domains 1–3
spf2.u_initphi	0	m/s	Velocity field, phi component	Domains 1–3
spf2.u_initz	0	m/s	Velocity field, z component	Domains 1–3
spf2.p_init	0	Pa	Pressure	Domains 1–3

2.6.5 Axial Symmetry 1

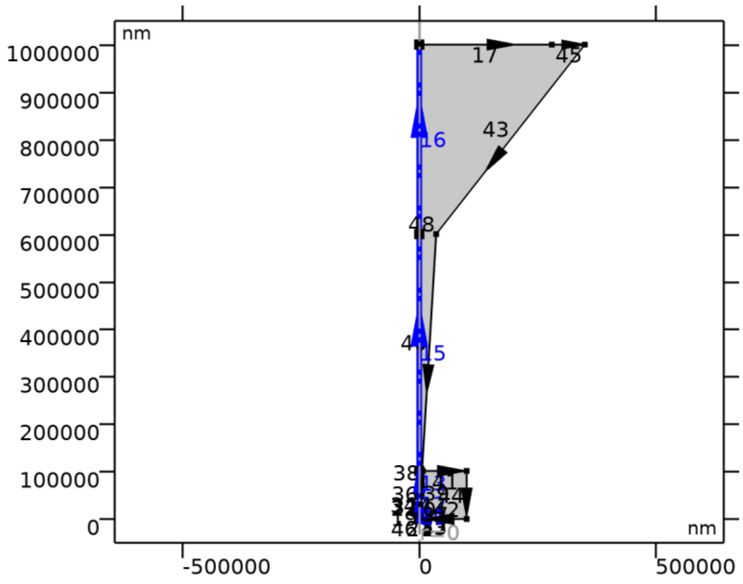

Axial Symmetry 1

SELECTION

Geometric entity level	Boundary
Selection	Geometry geom1: Dimension 1: All boundaries

2.6.5.1 Constraint Settings

SETTINGS

Description	Value
Apply reaction terms on	All physics (symmetric)
Constraint method	Elemental

2.6.6 Wall 1

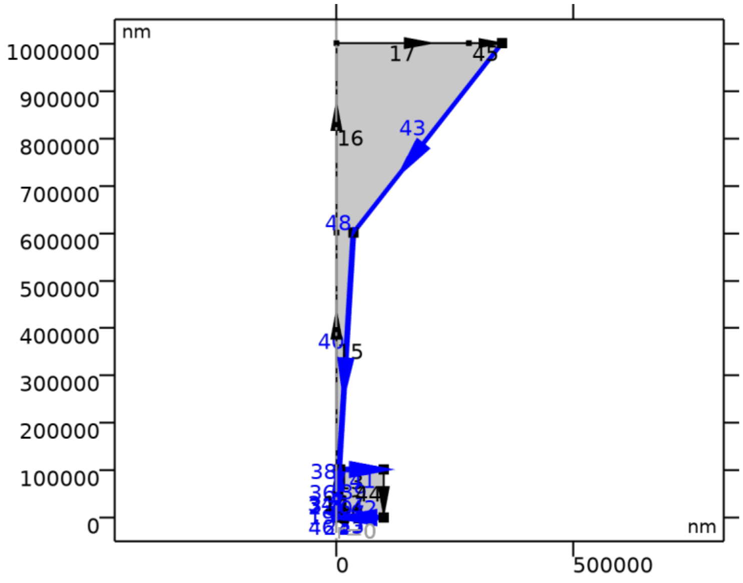

Wall 1

SELECTION

Geometric entity level	Boundary
Selection	Geometry geom1: Dimension 1: All boundaries

EQUATIONS

$$\mathbf{u2} = \mathbf{0}$$

2.6.6.1 Boundary Condition

SETTINGS

Description	Value
Wall condition	No slip

2.6.6.2 Wall Movement

SETTINGS

Description	Value
Translational velocity	Automatic from frame
Sliding wall	Off

2.6.6.3 Constraint Settings

SETTINGS

Description	Value
Constraints	Default

Description	Value
Apply reaction terms on	Individual dependent variables
Constraint method	Elemental

2.6.6.4 Variables

Name	Expression	Unit	Description	Selection	Details
spf2.ubndr	spf2.utrr+spf2.usr	m/s	Velocity at boundary, r component	Boundaries 2, 18–43, 46–48	
spf2.ubndphi	spf2.utrphi+spf2.usphi	m/s	Velocity at boundary, phi component	Boundaries 2, 18–43, 46–48	
spf2.ubndz	spf2.utrz+spf2.usz	m/s	Velocity at boundary, z component	Boundaries 2, 18–43, 46–48	
spf2.usr	0	m/s	Velocity of sliding wall, r component	Boundaries 2, 18–43, 46–48	
spf2.usphi	0	m/s	Velocity of sliding wall, phi component	Boundaries 2, 18–43, 46–48	
spf2.usz	0	m/s	Velocity of sliding wall, z component	Boundaries 2, 18–43, 46–48	
spf2.utrr	0	m/s	Velocity of moving wall, r component	Boundaries 2, 18–43, 46–48	
spf2.utrphi	0	m/s	Velocity of moving wall, phi component	Boundaries 2, 18–43, 46–48	
spf2.utrz	0	m/s	Velocity of moving wall, z component	Boundaries 2, 18–43, 46–48	
spf2.uLeakager	0	m/s	Leakage velocity, r component	Boundaries 2, 18–43, 46–48	+ operation
spf2.uLeakagephi	0	m/s	Leakage velocity, phi component	Boundaries 2, 18–43, 46–48	+ operation

Name	Expression	Unit	Description	Selection	Details
spf2.uLeakagez	0	m/s	Leakage velocity, z component	Boundaries 2, 18–43, 46–48	+ operation
spf2.noSlipWall	1	1	Help variable	Boundaries 2, 18–43, 46–48	

2.6.7 Volume Force 1

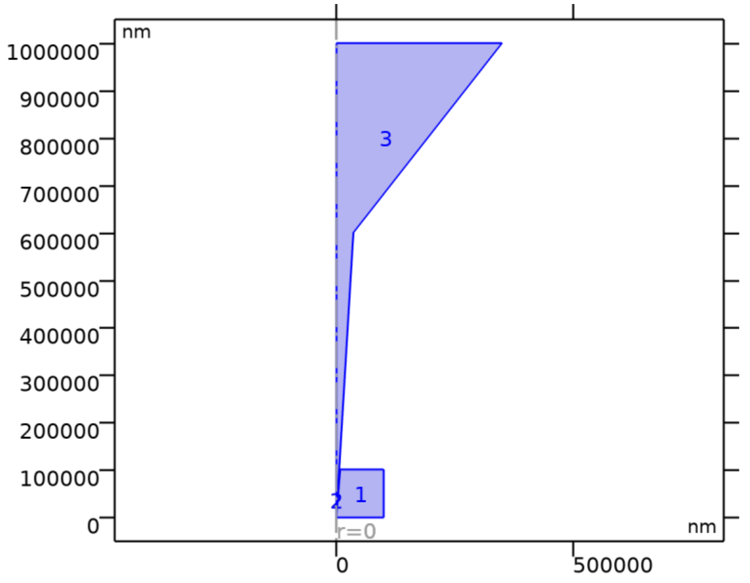

Volume Force 1

SELECTION

Geometric entity level	Domain
Selection	Geometry geom1: Dimension 2: All domains

EQUATIONS

$$\rho(\mathbf{u2}\cdot\nabla)\mathbf{u2} = \nabla\cdot[-p2\mathbf{I}+\mathbf{K}]+\mathbf{F}$$

2.6.7.1 Variables

Name	Expression	Unit	Description	Selection	Details
spf2.Fr	es.Er*F_const*(cK -cNO3-cCl)	N/m^3	Volume force, r component	Domains 1–3	+ operation
spf2.Fphi	0	N/m^3	Volume force, phi component	Domains 1–3	+ operation
spf2.Fz	es.Ez*F_const*(cK -cNO3-cCl)	N/m^3	Volume force, z component	Domains 1–3	+ operation

2.6.8 Boundary Stress 1

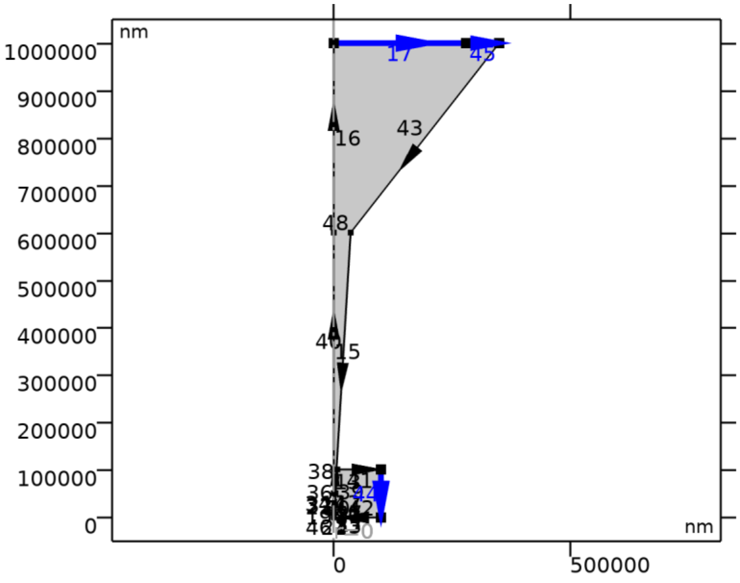

Boundary Stress 1

SELECTION

Geometric entity level	Boundary
Selection	Geometry geom1: Dimension 1: Boundaries 17, 44–45

EQUATIONS

$$[-p2\mathbf{I}+\mathbf{K}]\mathbf{n}=\mathbf{F}$$

2.6.8.1 Boundary Condition

SETTINGS

Description	Value
Boundary condition	General stress
Stress, r component	0
Stress, phi component	0
Stress, z component	0

2.6.8.2 Constraint Settings

SETTINGS

Description	Value
Use weak constraints	Off
Constraint method	Elemental

2.6.8.3 Variables

Name	Expression	Unit	Description	Selection
spf2.Fbndr	0	N/m^2	Stress, r component	Boundaries 17, 44–45
spf2.Fbndphi	0	N/m^2	Stress, phi component	Boundaries 17, 44–45
spf2.Fbndz	0	N/m^2	Stress, z component	Boundaries 17, 44–45
spf2.bs1.volumeFlowRate	spf2.bs1.intop(2*(u2*spf2.nrmesh+w2*spf2.nzmesh)*pi*r)	m^3/s	Outward volume flow rate across feature selection	Global
spf2.bs1.massFlowRate	spf2.bs1.intop(2*spf2.rho*(u2*spf2.nrmesh+w2*spf2.nzmesh)*pi*r)	kg/s	Outward mass flow rate across feature selection	Global
spf2.bs1.pAverage	spf2.bs1.intop(2*p2*pi*r)/max(spf2.bs1.intop(2*pi*r),1000*eps)	Pa	Pressure average over feature selection	Global

2.7 FAST MESH (CHARGE MAPPING, SIMPLE DELIVERY)

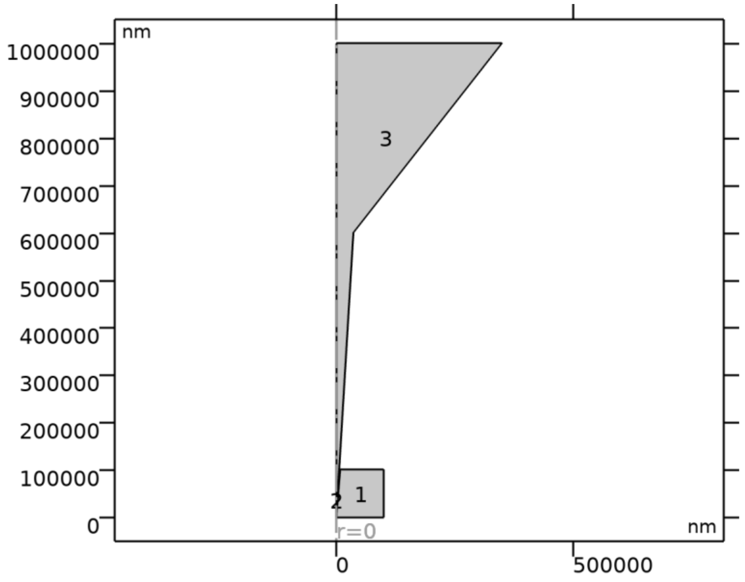

Fast Mesh (Charge mapping, simple delivery)

MESH STATISTICS

Description	Value
Status	Empty mesh
Mesh vertices	0

2.7.1 Size (size)

SETTINGS

Description	Value
Maximum element size	Bw/25
Minimum element size	0.5[nm]
Curvature factor	0.3
Resolution of narrow regions	10
Maximum element growth rate	1.2
Custom element size	Custom

2.7.2 Size 13 (size13)

SELECTION

Geometric entity level	Domain
Selection	Geometry geom1: Dimension 2: Domain 1

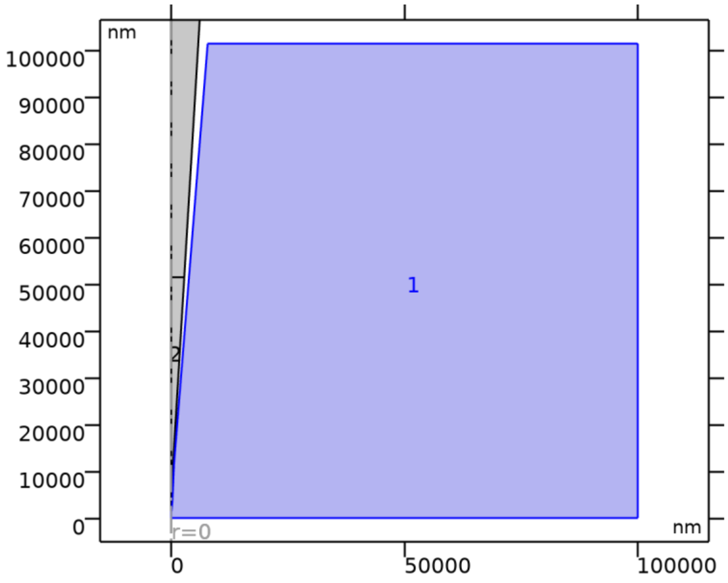

Size 13

SETTINGS

Description	Value
Maximum element size	Bw/25
Minimum element size	0.5[nm]

Description	Value
Minimum element size	Off
Curvature factor	0.3
Resolution of narrow regions	10
Resolution of narrow regions	Off
Custom element size	Custom

2.7.3 PipetteMesh (edg1)

SELECTION

Geometric entity level	Boundary
Selection	Geometry geom1: Dimension 1: Boundaries 3–13, 18–29, 31–39

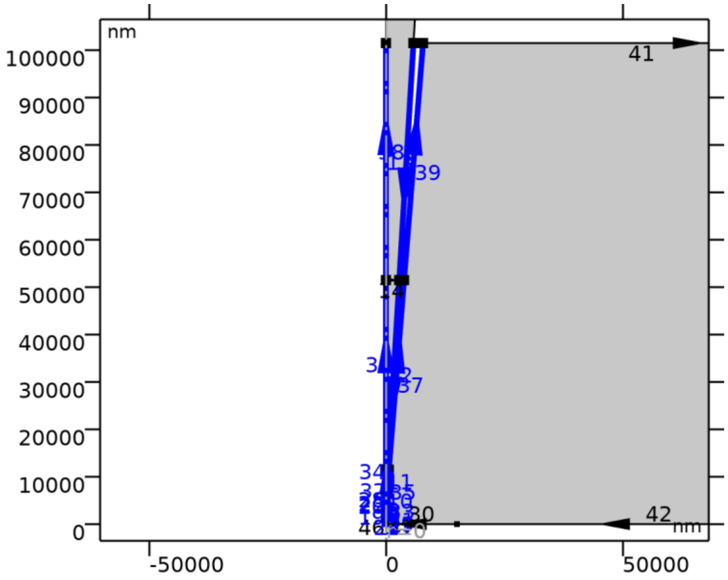

PipetteMesh

SETTINGS

Description	Value
Maximum element depth to process	3
Last build time	0
Built with	COMSOL 6.0.0.405 (win64)|2023 - 02 - 24T18:40:51.124745100

2.7.3.1 EndMesh (size13)

SELECTION

Geometric entity level	Boundary
Name	PipetteEndOnly (Part Instance 1)

Selection	Named geom1_pi1_boxsel32: Geometry geom1: Dimension 1: Boundary 21

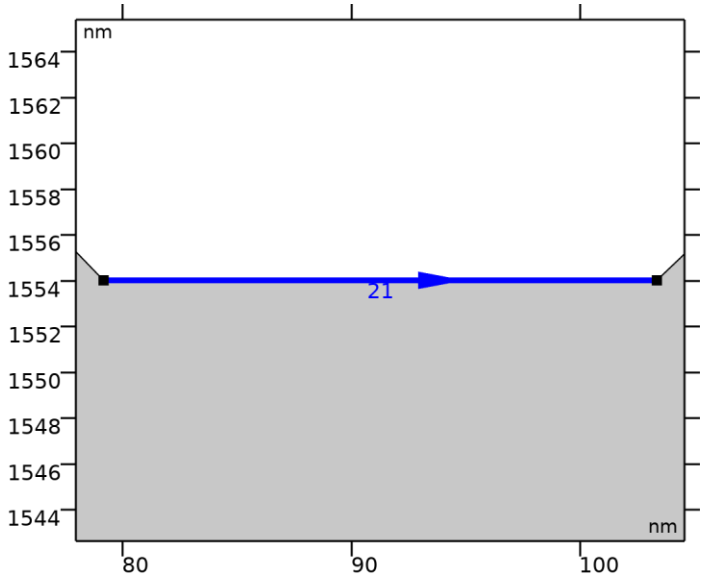

EndMesh

SETTINGS

Description	Value
Maximum element size	(OR0 - IR0)/mf
Minimum element size	(OR0 - IR0)/5
Minimum element size	Off
Curvature factor	0.3
Curvature factor	Off
Maximum element growth rate	1.05
Custom element size	Custom

2.7.3.2 Mesh0-50nm (size1)

SELECTION

Geometric entity level	Boundary
Name	BoundaryAndPipette 50nm (Part Instance 1)
Selection	Named geom1_pi1_boxsel3: Geometry geom1: Dimension 1: Boundaries 3, 18, 24

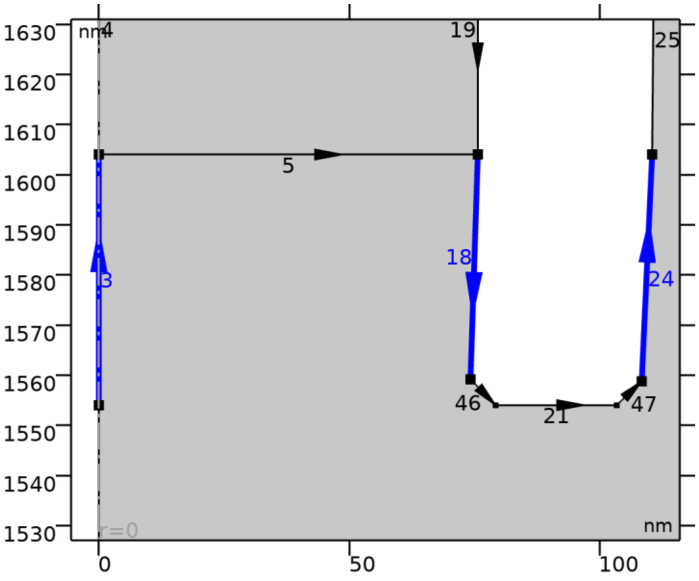

Mesh0-50nm

SETTINGS

Description	Value
Maximum element size	IR50/mf
Minimum element size	IR50/30
Curvature factor	0.3
Curvature factor	Off
Maximum element growth rate	Off
Custom element size	Custom

2.7.3.3 Mesh50-100nm (size15)

SELECTION

Geometric entity level	Boundary
Name	BoundaryAndPipette 100nm (Part Instance 1)
Selection	Named geom1_pi1_boxsel4: Geometry geom1: Dimension 1: Boundaries 4, 19, 25

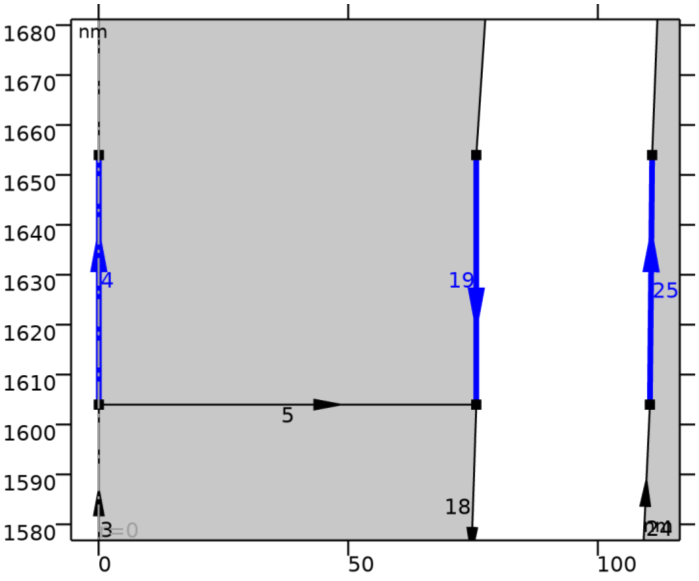

Mesh50-100nm

SETTINGS

Description	Value
Maximum element size	IR100/mf
Minimum element size	IR100/20
Curvature factor	0.3
Curvature factor	Off
Maximum element growth rate	1.05
Custom element size	Custom

2.7.3.4 Mesh100-200nm (size4)

SELECTION

Geometric entity level	Boundary
Name	BoundaryAndPipette 200nm (Part Instance 1)
Selection	Named geom1_pi1_boxsel5: Geometry geom1: Dimension 1: Boundaries 6, 20, 26

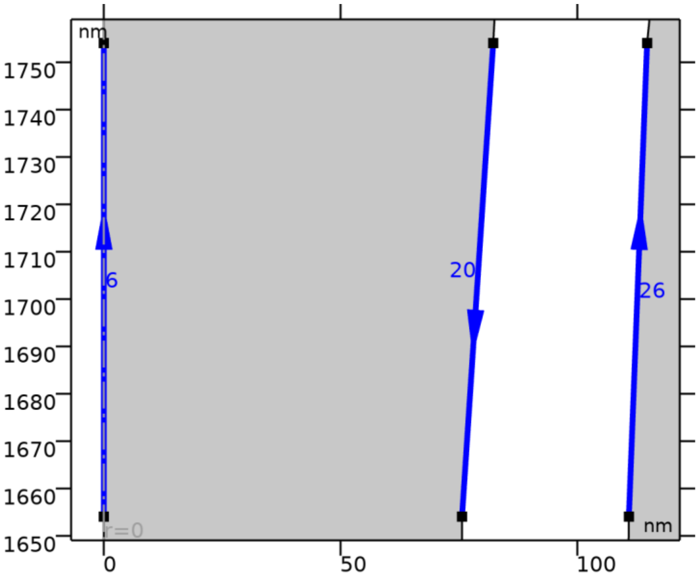

Mesh100-200nm

SETTINGS

Description	Value
Maximum element size	IR200/mf
Minimum element size	IR100/20
Curvature factor	0.3
Curvature factor	Off
Maximum element growth rate	1.08
Custom element size	Custom

2.7.3.5 Mesh200-500nm (size16)

SELECTION

Geometric entity level	Boundary
Name	BoundaryAndPipette 500nm (Part Instance 1)
Selection	Named geom1_pi1_boxsel2: Geometry geom1: Dimension 1: Boundaries 7, 22, 27

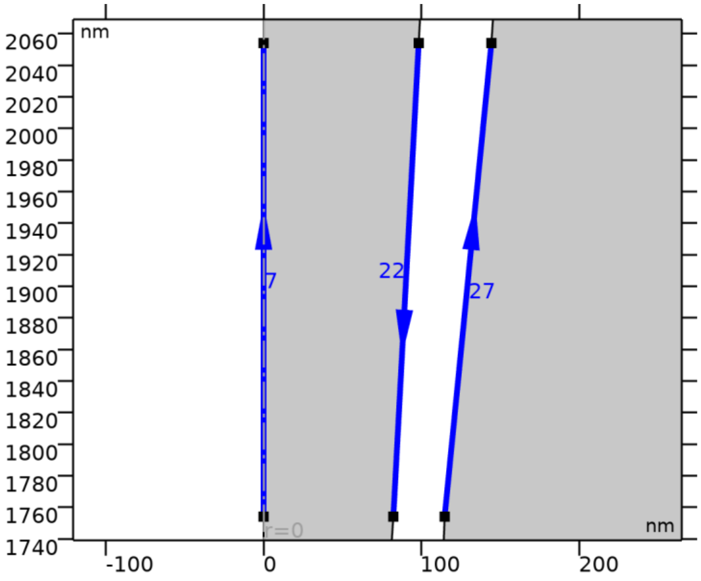

Mesh200-500nm

SETTINGS

Description	Value
Maximum element size	IR500/mf
Minimum element size	IR100/20
Curvature factor	0.3
Curvature factor	Off
Maximum element growth rate	1.08
Custom element size	Custom

2.7.3.6 Mesh500-1000nm (size5)

SELECTION

Geometric entity level	Boundary
Name	BoundaryAndPipette 1000nm (Part Instance 1)
Selection	Named geom1_pi1_boxsel6: Geometry geom1: Dimension 1: Boundaries 8, 23, 29

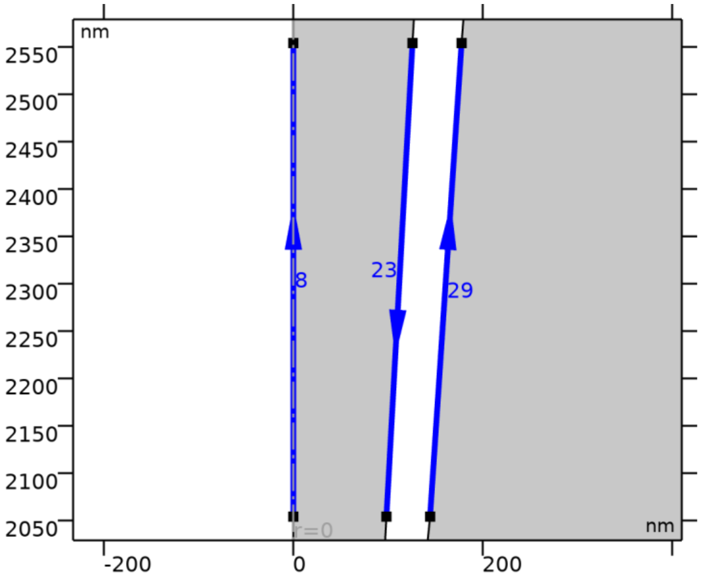

Mesh500-1000nm

SETTINGS

Description	Value
Maximum element size	IR2000/mf
Minimum element size	IR500/mf
Curvature factor	0.3
Curvature factor	Off
Custom element size	Custom

2.7.3.7 Mesh1000-2000nm (size17)

SELECTION

Geometric entity level	Boundary
Name	BoundaryAndPipette 2000nm (Part Instance 1)
Selection	Named geom1_pi1_boxsel7: Geometry geom1: Dimension 1: Boundaries 9, 28, 31

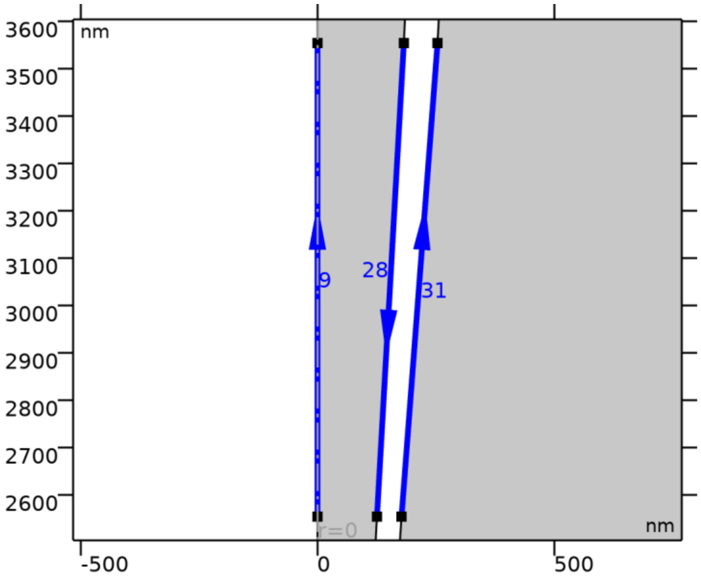

Mesh1000-2000nm

SETTINGS

Description	Value
Maximum element size	IR2000/mf
Minimum element size	IR500/mf
Curvature factor	0.3
Curvature factor	Off
Custom element size	Custom

2.7.3.8 Mesh2000-5000nm (size7)

SELECTION

Geometric entity level	Boundary
Name	BoundaryAndPipette 5000nm (Part Instance 1)
Selection	Named geom1_pi1_boxsel8: Geometry geom1: Dimension 1: Boundaries 10, 32–33

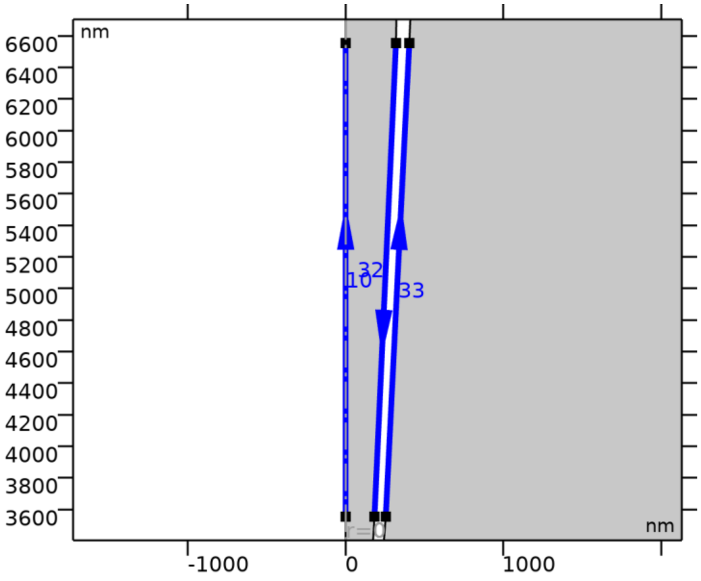

Mesh2000-5000nm

SETTINGS

Description	Value
Maximum element size	IR5000/mf
Minimum element size	IR2000/mf
Curvature factor	0.3
Curvature factor	Off
Custom element size	Custom

2.7.3.9 Mesh5000-10000nm (size8)

SELECTION

Geometric entity level	Boundary
Name	BoundaryAndPipette 10000nm (Part Instance 1)
Selection	Named geom1_pi1_boxsel9: Geometry geom1: Dimension 1: Boundaries 11, 34–35

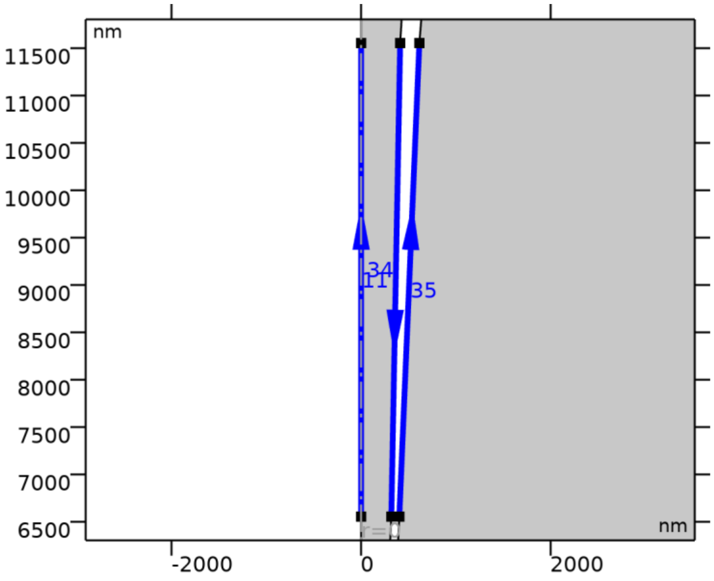

Mesh5000-10000nm

SETTINGS

Description	Value
Maximum element size	IR10000/mf
Minimum element size	IR5000/mf
Curvature factor	0.3
Curvature factor	Off
Custom element size	Custom

2.7.3.10 Mesh10000-50000nm (size9)

SELECTION

Geometric entity level	Boundary
Name	BoundaryAndPipette 50000nm (Part Instance 1)
Selection	Named geom1_pi1_boxsel10: Geometry geom1: Dimension 1: Boundaries 12, 36–37

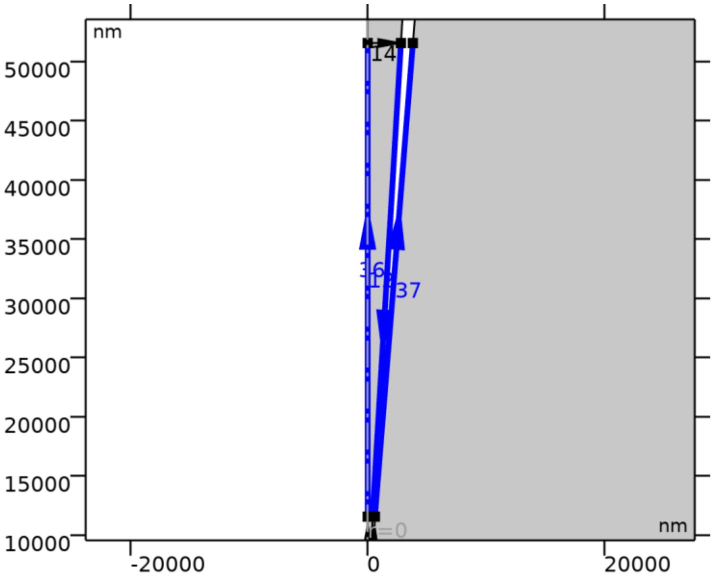

Mesh10000-50000nm

SETTINGS

Description	Value
Maximum element size	IR50000/mf
Minimum element size	IR10000/mf
Curvature factor	0.3
Curvature factor	Off
Custom element size	Custom

2.7.3.11 Mesh50000-100000nm (size10)

SELECTION

Geometric entity level	Boundary
Name	BoundaryAndPipette 100000nm (Part Instance 1)
Selection	Named geom1_pi1_boxsel11: Geometry geom1: Dimension 1: Boundaries 13, 38–39

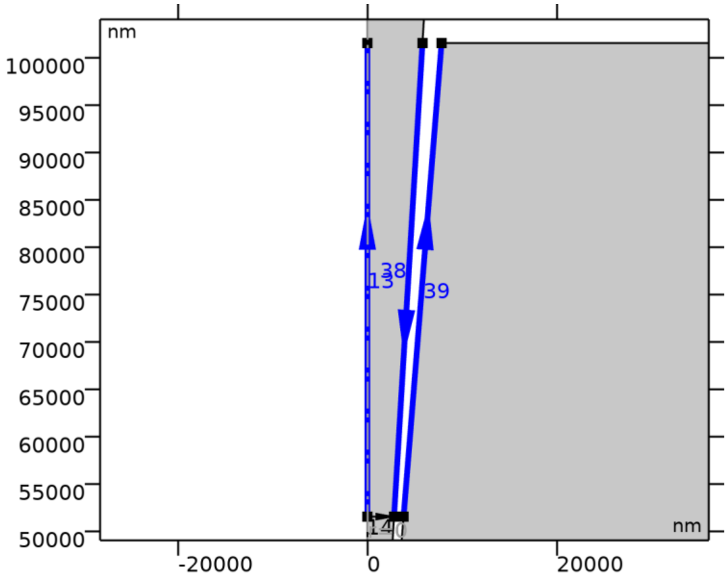

Mesh50000-100000nm

SETTINGS

Description	Value
Maximum element size	IR100000/mf
Minimum element size	IR50000/mf
Curvature factor	0.3
Curvature factor	Off
Maximum element growth rate	1.05
Custom element size	Custom

2.7.3.12 Mesh100000nm-lp (size11)

SELECTION

Geometric entity level	Boundary
Name	BoundaryAndPipette lp (Part Instance 1)
Selection	Named geom1_pi1_boxsel12: Geometry geom1: Dimension 1: Boundaries 15, 40, 48

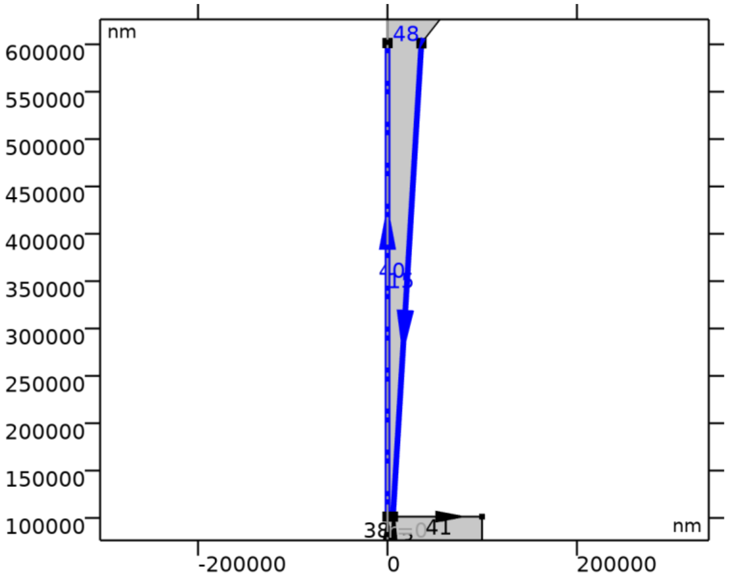

Mesh100000nm-lp

SETTINGS

Description	Value
Maximum element size	IRlp/mf
Minimum element size	IR100000/mf
Curvature factor	0.3
Curvature factor	Off
Custom element size	Custom

2.7.3.13 Meshlp-taper symm (size19)

SELECTION

Geometric entity level	Boundary
Name	BoundaryAndPipette taper (Part Instance 1)
Selection	Named geom1_pi1_boxsel13: Geometry geom1: Dimension 1: Boundaries 16, 43

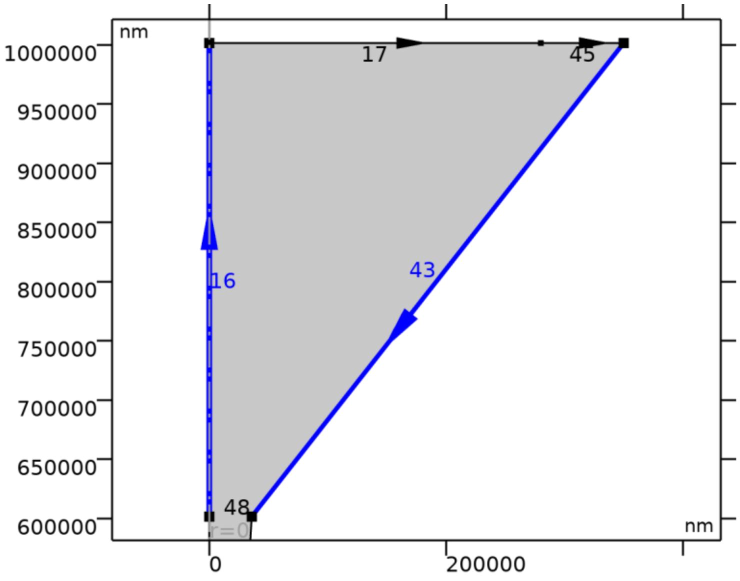

Meshlp-taper symm

SETTINGS

Description	Value
Maximum element size	lRtaper/mf
Minimum element size	lRlp/mf
Curvature factor	0.3
Curvature factor	Off
Maximum element growth rate	1.02
Custom element size	Custom

2.7.3.14 Meshlp-taper (size14)

SELECTION

Geometric entity level	Boundary
Name	Pipette taper BL6 (Part Instance 1)
Selection	Named geom1_pi1_boxsel26: Geometry geom1: Dimension 1: Boundary 43

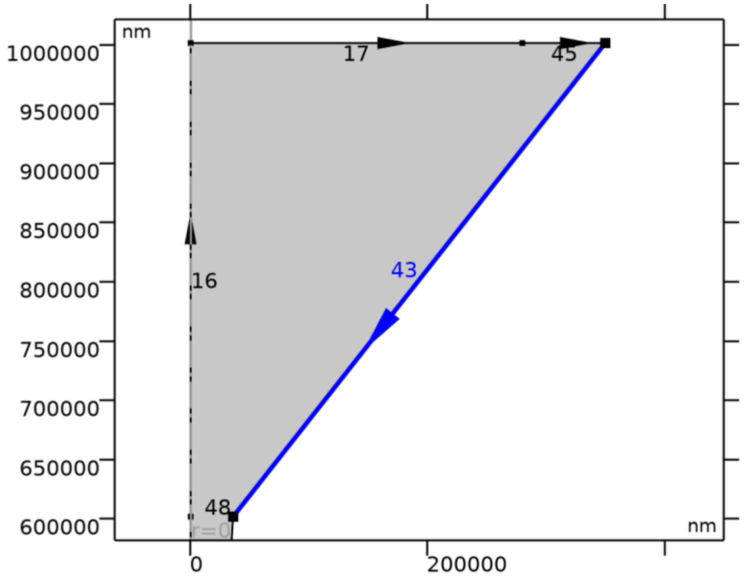

Meshlp-taper

SETTINGS

Description	Value
Maximum element size	IRtaper/mf
Minimum element size	IRlp/mf
Curvature factor	0.3
Curvature factor	Off
Maximum element growth rate	1.01
Custom element size	Custom

2.7.4 Substrate (edg2)

SELECTION

Geometric entity level	Boundary
Selection	Geometry geom1: Dimension 1: Boundaries 2, 30, 42

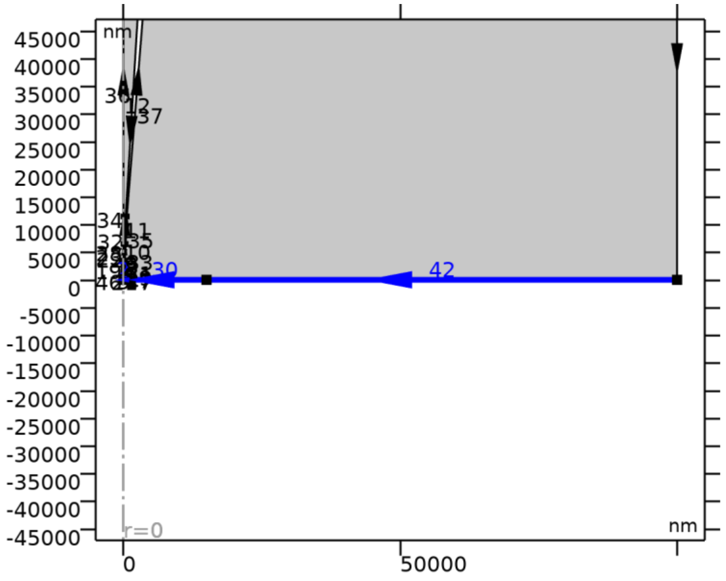

Substrate

SETTINGS

Description	Value
Maximum element depth to process	3
Last build time	0
Built with	COMSOL 6.0.0.405 (win64)|2023 - 02 - 24T18:40:51.144739800

2.7.4.1 SubNearMesh (size6)

SELECTION

Geometric entity level	Boundary
Selection	Geometry geom1: Dimension 1: Boundary 2

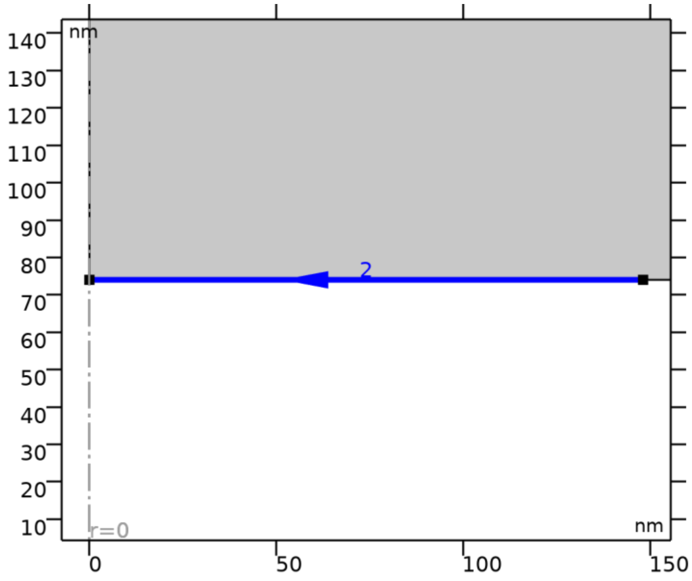

SubNearMesh

SETTINGS

Description	Value
Maximum element size	IR0/50
Minimum element size	5[nm]
Minimum element size	Off
Curvature factor	0.3
Curvature factor	Off
Resolution of narrow regions	Off
Maximum element growth rate	1.05
Maximum element growth rate	Off
Custom element size	Custom

2.7.4.2 SubFarMesh (size15)

SELECTION

Geometric entity level	Boundary
Selection	Geometry geom1: Dimension 1: Boundary 30

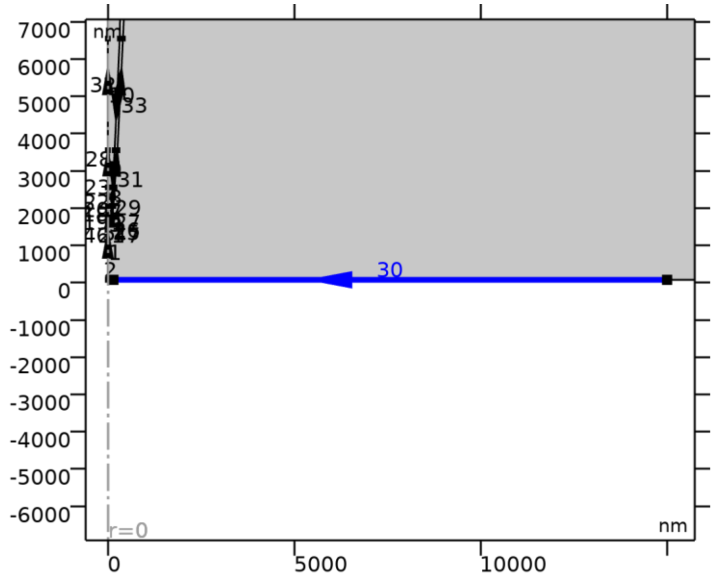

SubFarMesh

SETTINGS

Description	Value
Maximum element size	50[nm]
Minimum element size	9.0E-7
Minimum element size	Off
Curvature factor	0.3
Curvature factor	Off
Resolution of narrow regions	Off
Maximum element growth rate	1.02
Custom element size	Custom

2.7.4.3 SubInactMesh (size16)

SELECTION

Geometric entity level	Boundary
Selection	Geometry geom1: Dimension 1: Boundary 42

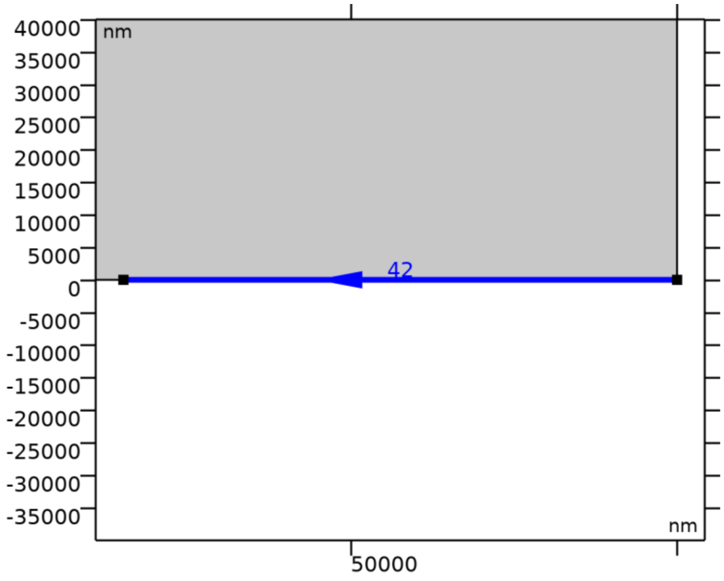

SubInactMesh

SETTINGS

Description	Value
Maximum element size	200[nm]
Minimum element size	5[nm]
Minimum element size	Off
Curvature factor	0.3
Curvature factor	Off
Resolution of narrow regions	Off
Maximum element growth rate	1.01
Maximum element growth rate	Off
Custom element size	Custom

2.7.5 Boundary Layers 1 (bl1)

SELECTION

Geometric entity level	Domain
Selection	Geometry geom1: Dimension 2: All domains

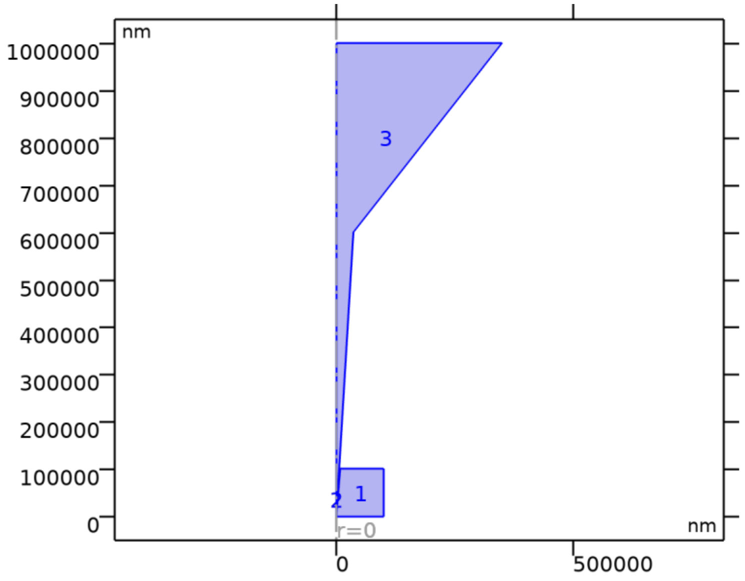

Boundary Layers 1

SETTINGS

Description	Value
Maximum angle per split	30
Maximum layer decrement	1
Maximum element depth to process	3
Last build time	32
Built with	COMSOL 6.0.0.405 (win64)|2023 - 02 - 24T18:41:23.900745300

2.7.5.1 Boundary Layer BL1 (blp)

SELECTION

Geometric entity level	Boundary
Name	BL1 (Part Instance 1)
Selection	Named geom1_pi1_csel3_bnd: Geometry geom1: Dimension 1: Boundaries 21, 46–47

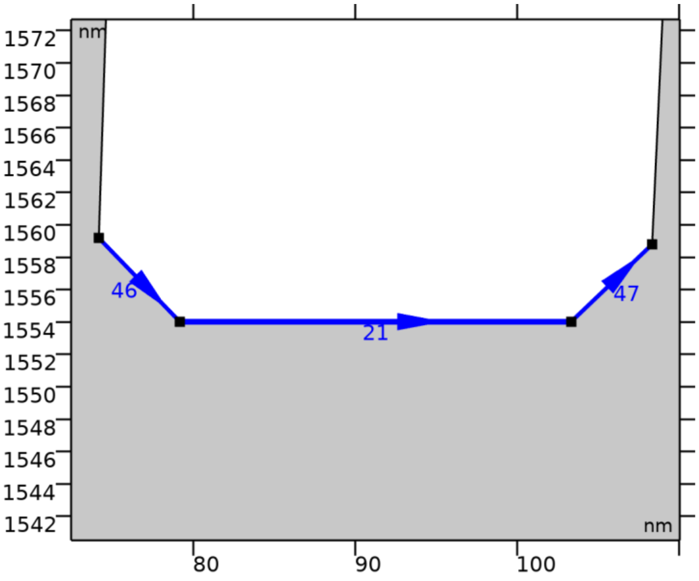

Boundary Layer BL1

SETTINGS

Description	Value
Number of layers	numBL
Stretching factor	1.03
Thickness specification	First layer
Thickness	0.2[nm]

2.7.5.2 Boundary Layer BL2 (blp9)

SELECTION

Geometric entity level	Boundary
Name	BL2 (Part Instance 1)
Selection	Named geom1_pi1_csel4_bnd: Geometry geom1: Dimension 1: Boundaries 18–19, 24–25

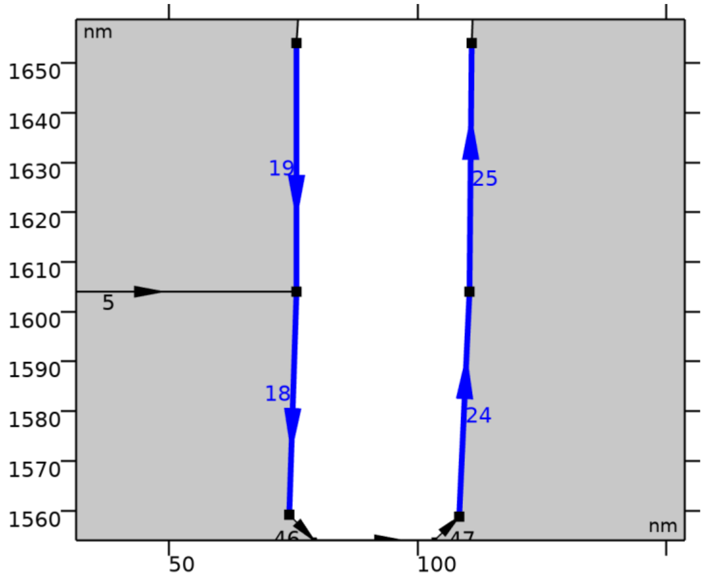

Boundary Layer BL2

SETTINGS

Description	Value
Number of layers	numBL
Stretching factor	1.04
Thickness specification	First layer
Thickness	0.2[nm]

2.7.5.3 Boundary Layer BL3 (blp8)

SELECTION

Geometric entity level	Boundary
Name	BL3 (Part Instance 1)
Selection	Named geom1_pi1_csel5_bnd: Geometry geom1: Dimension 1: Boundaries 20, 26

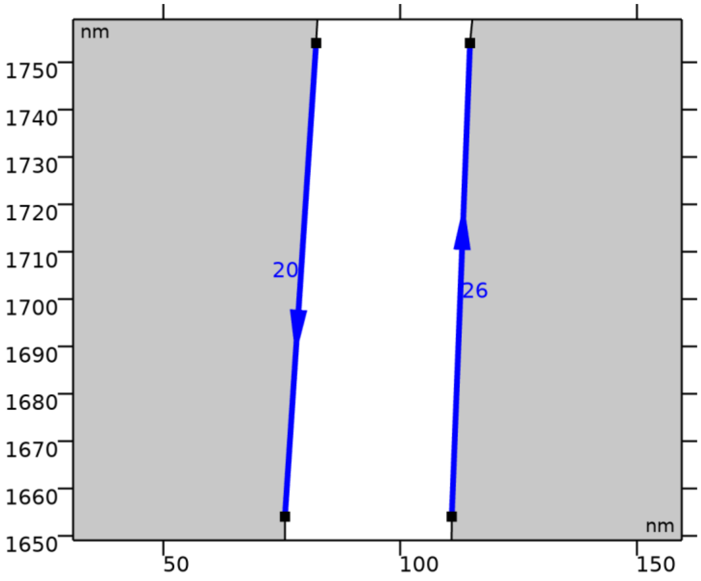

Boundary Layer BL3

SETTINGS

Description	Value
Number of layers	numBL
Stretching factor	1.055
Thickness specification	First layer
Thickness	0.2[nm]

2.7.5.4 Boundary Layer BL4 (blp2)

SELECTION

Geometric entity level	Boundary
Name	BL4 (Part Instance 1)
Selection	Named geom1_pi1_csel6_bnd: Geometry geom1: Dimension 1: Boundaries 22, 27

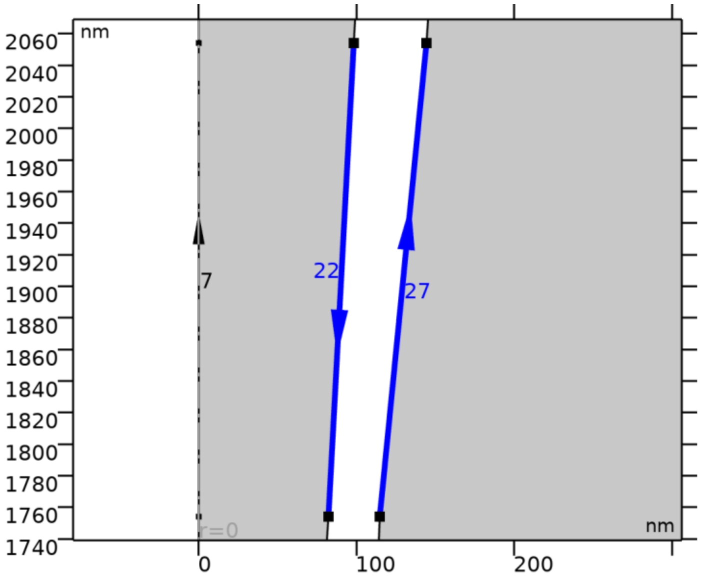

Boundary Layer BL4

SETTINGS

Description	Value
Number of layers	numBL
Stretching factor	1.07
Thickness specification	First layer
Thickness	0.2[nm]

2.7.5.5 Boundary Layer BL5 (blp3)

SELECTION

Geometric entity level	Boundary
Name	BL5 (Part Instance 1)
Selection	Named geom1_pi1_csel7_bnd: Geometry geom1: Dimension 1: Boundaries 23, 29

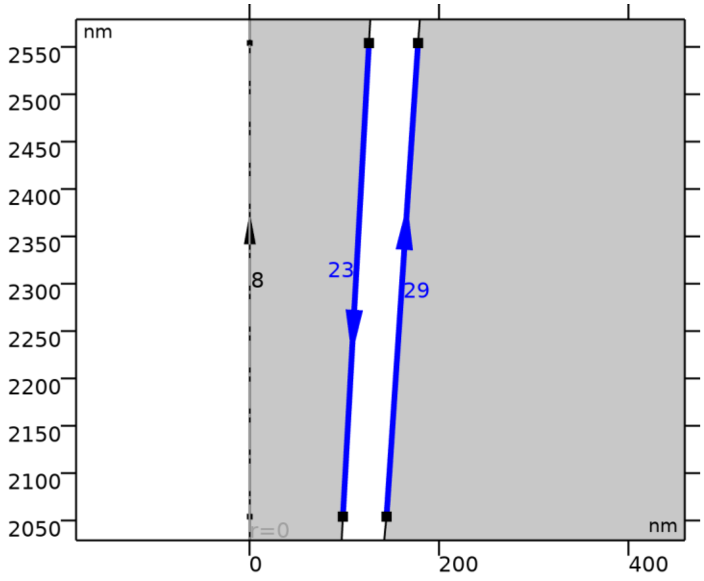

Boundary Layer BL5

SETTINGS

Description	Value
Number of layers	numBL
Stretching factor	1.1
Thickness specification	First layer
Thickness	0.2[nm]

2.7.5.6 Boundary Layer BL6 (blp10)

SELECTION

Geometric entity level	Boundary
Name	BL6 (Part Instance 1)
Selection	Named geom1_pi1_csel8_bnd: Geometry geom1: Dimension 1: Boundaries 28, 31–40, 43, 48

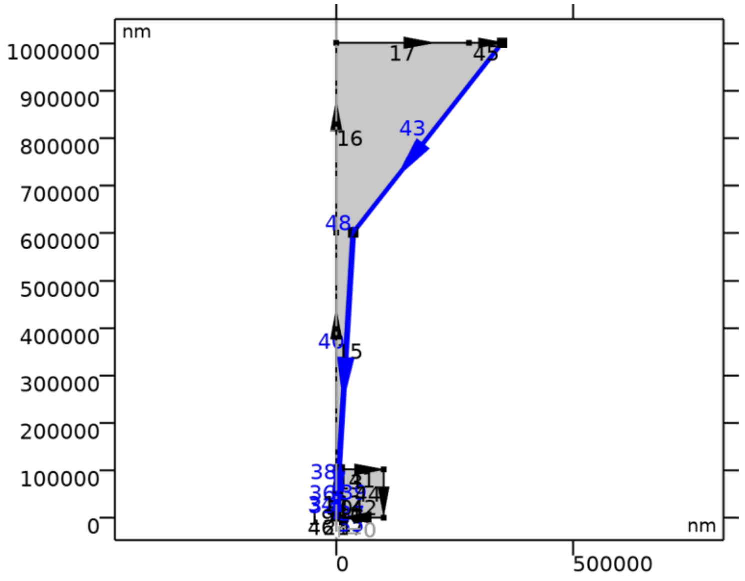

Boundary Layer BL6

SETTINGS

Description	Value
Number of layers	numBL
Stretching factor	1.12
Thickness specification	First layer
Thickness	0.2[nm]

2.7.6 Boundary Layers 2 (bl2)

SELECTION

Geometric entity level	Domain
Selection	Geometry geom1: Dimension 2: Domains 1, 3

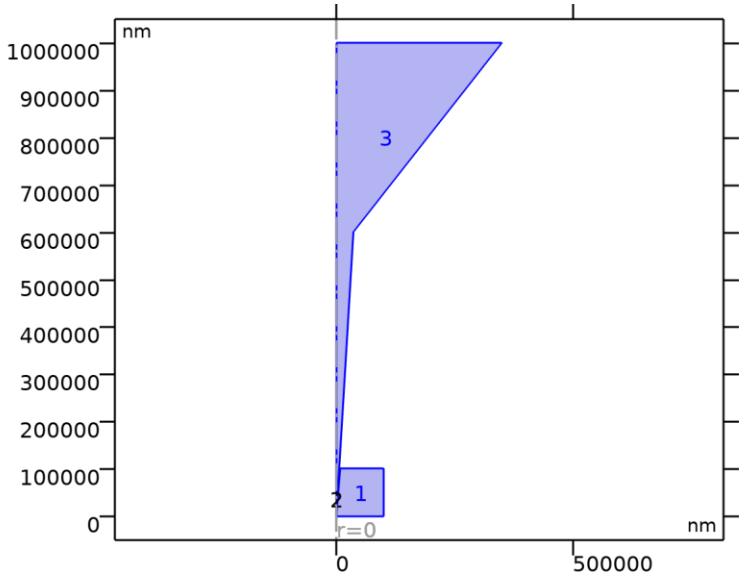

Boundary Layers 2

SETTINGS

Description	Value
Maximum angle per split	30
Maximum layer decrement	1
Maximum element depth to process	3
Last build time	2
Built with	COMSOL 6.0.0.405 (win64)|2023 - 02 - 24T18:41:26.724272200

2.7.6.1 Boundary Layer SubActiveNear (blp1)

SELECTION

Geometric entity level	Boundary
Selection	Geometry geom1: Dimension 1: Boundary 2

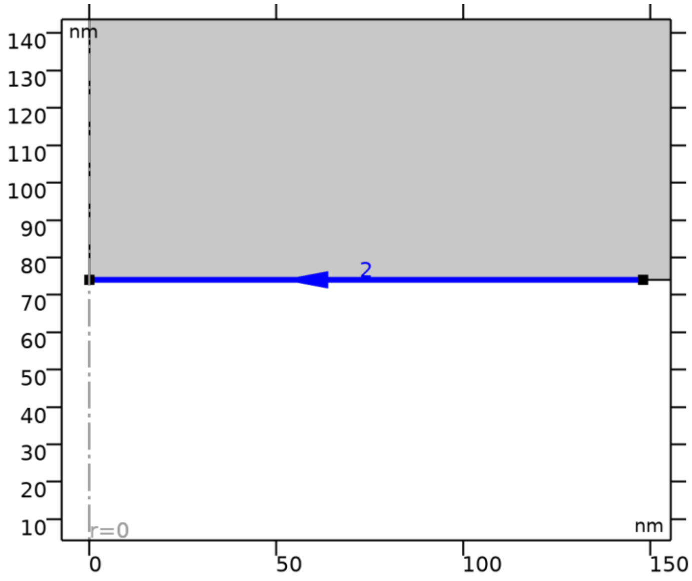

Boundary Layer SubActiveNear

SETTINGS

Description	Value
Number of layers	numBL
Stretching factor	if(dd>15[nm], 1.05, 1)

2.7.6.2 Boundary Layer SubActiveFar (blp6)

SELECTION

Geometric entity level	Boundary
Selection	Geometry geom1: Dimension 1: Boundary 30

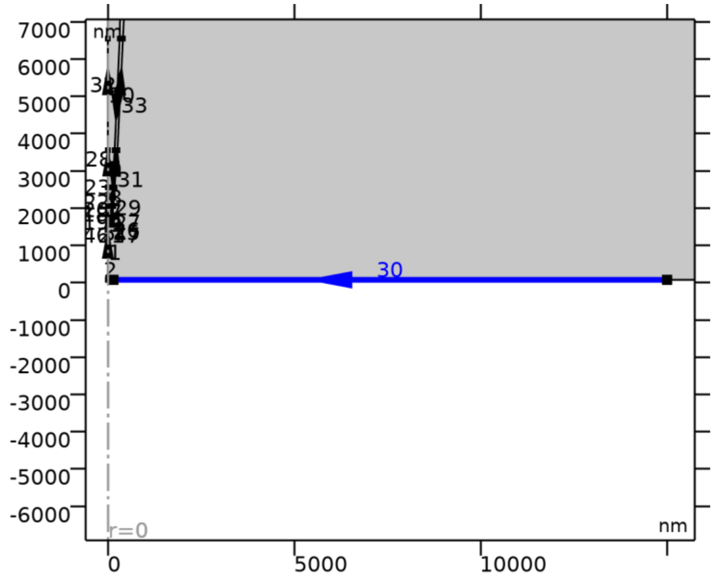

Boundary Layer SubActiveFar

SETTINGS

Description	Value
Number of layers	numBL
Stretching factor	1.06

2.7.6.3 Boundary Layer SubInactive (blp7)

SELECTION

Geometric entity level	Boundary
Selection	Geometry geom1: Dimension 1: Boundary 42

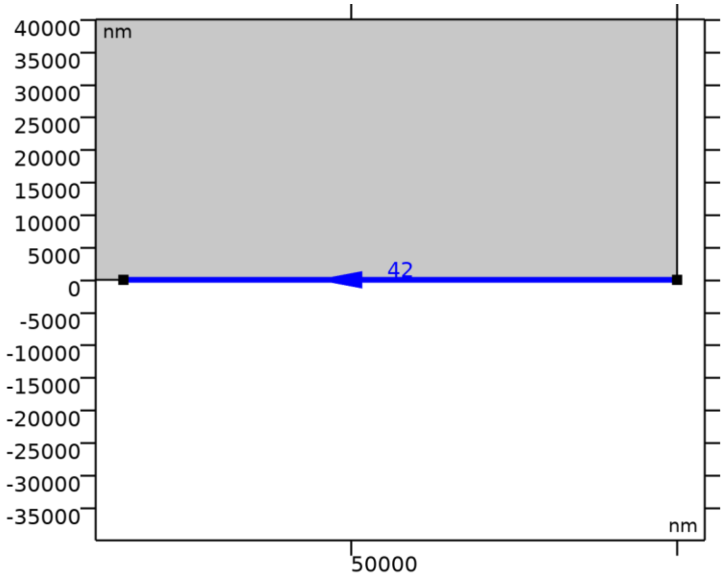

Boundary Layer SubInactive

SETTINGS

Description	Value
Number of layers	numBL
Stretching factor	1.1

2.7.7 MeshTop (size12)

SELECTION

Geometric entity level	Boundary
Name	Top Boundary (Part Instance 1)
Selection	Named geom1_pi1_boxsel28: Geometry geom1: Dimension 1: Boundary 17

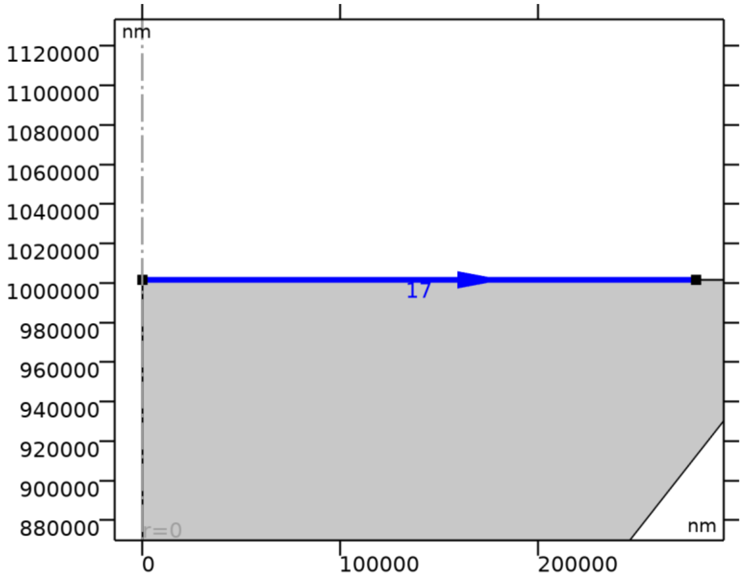

MeshTop

SETTINGS

Description	Value
Maximum element size	IRlp/10
Maximum element size	Off
Minimum element size	9.03E-8
Minimum element size	Off
Curvature factor	0.3
Curvature factor	Off
Maximum element growth rate	1
Custom element size	Custom
